# Supplementary material for: Genome-wide determination of on-target and off-target characteristics for RNA-guided DNA methylation by dCas9 methyltransferases
Source: Gigascience. 2018 Feb 19;7(3):giy011. doi: 10.1093/gigascience/giy011 (PMC5888497; doi:10.1093/gigascience/giy011)
Supplement: GIGA-D-17-00040_Original_Submission.pdf [file giy011_giga-d-17-00040_original_submission.pdf]

GigaScience

Genome wide determination of on-target and off-target characteristics for RNA-guided DNA Methylation by dCas9 methyltransferases (CRISPRme)

--Manuscript Draft--

**Manuscript Number:**	GIGA-D-17-00040
**Full Title:**	Genome wide determination of on-target and off-target characteristics for RNA-guided DNA Methylation by dCas9 methyltransferases (CRISPRme)
**Article Type:**	Research

Funding Information:

Teknologi og Produktion, Det Frie Forskningsråd (DFF–1337-00128)	Prof. Yonglun Luo
Teknologi og Produktion, Det Frie Forskningsråd (DFF-1335-00763A)	Prof. Yonglun Luo
Innovation Fund Denmark (BrainStem)	Prof. Yonglun Luo
Lundbeckfonden (R173-2014-1105)	Prof. Yonglun Luo
Lundbeckfonden (R151-2013-14439)	Prof. Lars Bolund
Lundbeckfonden (R219-2016-1375)	Dr. Lin Lin
Toyota Foundation (JP)	Prof. Anders Lade Nielsen
Lundbeckfonden	Prof. Anders Lade Nielsen

Abstract:

Background
Fusion of active protein domains to the nuclease-deficient clustered regularly interspaced short palindromic repeat (CRISPR) associated protein 9 (dCas9) has been used for epigenome editing, but the specificities of these engineered proteins have still not been fully investigated.

Findings
In this study, we generated a CRISPR-guided DNA methyltransferases (CRISPRme) by fusing the catalytic domain of DNMT3A or DNMT3B to the C terminus of the dCas9 protein from S. pyogenes and validated its on-target and global off-target characteristics. Using targeted quantitative bisulfite pyrosequencing, we prove that CRISPRme can efficiently methylate the CpG dinucleotides flanking its target sites in genomic loci (uPA and TGFBR3) in human cells (HEK293T) with de novo CpG-methylation levels exceeding 70% for some target sites. Furthermore, we conducted whole genome bisulfite sequencing to address the specificity of dCas9 methyltransferases. Whole genome bisulfite sequencing revealed that although CRISPRme did not cause global methylation changes, a substantial amount of off-target differentially methylated regions were identified in cells overexpressing CRISPRme and gRNAs. These off-target differentially methylated regions were predominantly found in promoter regions, 5' un-translated regions, CpG islands, and DNase I hypersensitivity sites. Through chromatin immunoprecipitation with massive parallel DNA sequencing analysis, we further revealed that these off-target DMRs were weakly correlated with dCas9 off-target binding sites. Using qPCR, fluorescent reporter cells and RNA sequencing, we also found that CRISPRme can mediate transient inhibition of gene expression. However, this effect appears to result from dCas9-mediated interference with transcription rather than de novo DNA methylation of the promoter investigated.

Conclusion
Our results prove that CRISPRme allows for efficient RNA-guided methylation of endogenous CpGs. Still there is significant off-target methylation indicating that further improvements of the specificity of CRISPR-dCas9 based DNA methylation modifiers are required.

**Corresponding Author:**	Yonglun Luo

Powered by Editorial Manager® and ProduXion Manager® from Aries Systems Corporation

	Aarhus Universitet Health<br>Aarhus, DENMARK
**Corresponding Author Secondary Information:**	
**Corresponding Author's Institution:**	Aarhus Universitet Health
**Corresponding Author's Secondary Institution:**	
**First Author:**	Lin Lin
**First Author Secondary Information:**	
**Order of Authors:**	Lin Lin
	Yong Liu
	Jingrong Huang
	Fengping Xu
	Tina Fuglsang Daugaard
	Trine Skov Petersen
	Bettina Hansen
	Lingfei ye
	Qing Zhou
	Fang Fang
	Shengting Li
	Lasse Fløe
	Kristopher Torp Jensen
	Ellen Shrock
	Huanming Yang
	Jian Wang
	Xun Xu
	Lars Bolund
	Anders Lade Nielsen
	Yonglun Luo
**Order of Authors Secondary Information:**	
**Opposed Reviewers:**	
**Additional Information:**	
**Question**	**Response**
Are you submitting this manuscript to a special series or article collection?	No
**Experimental design and statistics**<br><br>Full details of the experimental design and statistical methods used should be given in the Methods section, as detailed in our Minimum Standards Reporting Checklist. Information essential to interpreting the data presented should be made available in the figure legends.	Yes

Have you included all the information requested in your manuscript?	
**Resources**<br><br>A description of all resources used, including antibodies, cell lines, animals and software tools, with enough information to allow them to be uniquely identified, should be included in the Methods section. Authors are strongly encouraged to cite Research Resource Identifiers (RRIDs) for antibodies, model organisms and tools, where possible.<br><br>Have you included the information requested as detailed in our Minimum Standards Reporting Checklist?	Yes
**Availability of data and materials**<br><br>All datasets and code on which the conclusions of the paper rely must be either included in your submission or deposited in publicly available repositories (where available and ethically appropriate), referencing such data using a unique identifier in the references and in the "Availability of Data and Materials" section of your manuscript.<br><br>Have you have met the above requirement as detailed in our Minimum Standards Reporting Checklist?	Yes

Manuscript
Click here to download Manuscript CRISPRme_R6 GigaScience.docx

Genome wide determination of on-target and off-target characteristics for RNA-guided DNA Methylation by dCas9 methyltransferases (CRISPRme)

Lin Lin [1, 7, §], Yong Liu [1, §], Jinrong Huang [2, 3, §], Fengping Xu [2, 3, 4, §], Tina Fuglsang Daugaard [1], Trine Skov Petersen [1], Bettina Hansen [1], Lingfei Ye [2], Qing Zhou [2, 3], Fang Fang [2, 3], Shengting Li [1, 2], Lasse Fløe [1], Kristopher Torp Jensen [1], Ellen Shrock [6], Huanming Yang [2, 4], Jian Wang [2, 4], Xun Xu [2, 3, *], Lars Bolund [1, 2, 7], Anders Lade Nielsen [1], Yonglun Luo [1, 7, 8 *]

Initials for authors:
L.L., Y.Liu., F.X., J.H., T.F.D., T.S.P., B.H., L.Y., Q.Z., F.F., S.L., L.F., E.S., H.Y., J.W., X.X., L.B., A.L.N., Y.L.

Affiliations for authors:
1. Department of Biomedicine, Aarhus University, Aarhus, Denmark
2. BGI-Shenzhen, Shenzhen 518083, China
3. China National GeneBank-Shenzhen, BGI-Shenzhen, Shenzhen 518083, China
4. Department of Biology, University of Copenhagen, Copenhagen, Denmark
5. James D. Watson Institute of Genome Sciences, Hangzhou 310058, China
6. Department of Genetics, Harvard Medical School, Boston, MA, USA.
7. Danish Regenerative Engineering Alliance for Medicine (DREAM), Department of Biomedicine, Aarhus University, Aarhus, Denmark
8. BrainStem - Stem Cell Center of Excellence in Neurology, Copenhagen, Denmark
§. These authors contributed equally to the study
*. All correspondence should be addressed to Xun Xu (XUXUN@GENOMICS.CN) and Yonglun Luo (ALUN@BIOMED.AU.DK)

Emails:
Lin Lin: lin.lin@biomed.au.dk
Yong Liu: liuyongbox@gmail.com
Jinrong Huang: huangjinrong@genomics.cn
Fengping Xu: xufengping@genomics.cn
Tina Fuglsang Daugaard: tfm@biomed.au.dk
Trine Skov Petersen: trinesp@biomed.au.dk
Bettina Hansen: bhansen@biomed.au.dk
Lingfei Ye: yelingfei@genomics.cn

Qing Zhou: zhouqing1@genomics.cn
Fang Fang: fangfang@genomics.org.cn
Shengting Li: lishengting@gmail.com
Lasse Fløe: lassefloe@gmail.com
Kristopher Torp Jensen: kristopher.torp@gmail.com
Ellen Shrock: ellen_shrock@g.harvard.edu
Huanming Yang: yanghm@genomics.cn
Jian Wang: wangjian@genomics.cn
Xun Xu: xuxun@genomics.cn
Lars Bolund: bolund@biomed.au.dk
Anders Lade Nielsen: aln@biomed.au.dk
Yonglun Luo: alun@biomed.au.dk

Graphical Abstract

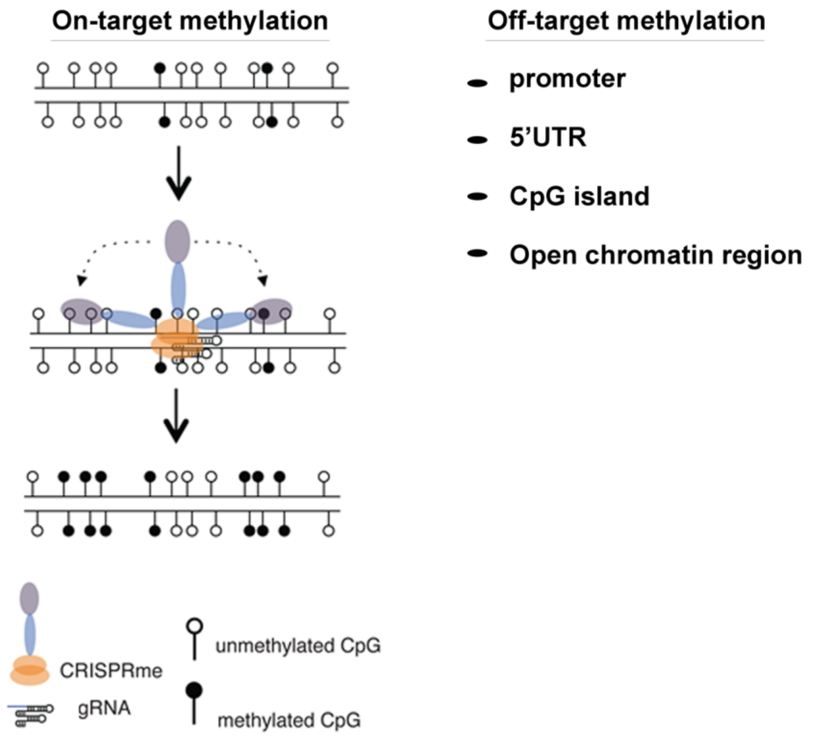

Abstract

Background

Fusion of active protein domains to the nuclease-deficient clustered regularly interspaced short palindromic repeat (CRISPR) associated protein 9 (dCas9) has been used for epigenome editing, but the specificities of these engineered proteins have still not been fully investigated.

Findings

In this study, we generated a CRISPR-guided DNA methyltransferases (CRISPRme) by fusing the catalytic domain of DNMT3A or DNMT3B to the C terminus of the dCas9 protein from *S. pyogenes* and validated its on-target and global off-target characteristics. Using targeted quantitative bisulfite pyrosequencing, we prove that CRISPRme can efficiently methylate the CpG dinucleotides flanking its target sites in genomic loci (*uPA* and *TGFBR3*) in human cells (HEK293T) with *de novo* CpG-methylation levels exceeding 70% for some target sites. Furthermore, we conducted whole genome bisulfite sequencing to address the specificity of dCas9 methyltransferases. Whole genome bisulfite sequencing revealed that although CRISPRme did not cause global methylation changes, a substantial amount of off-target differentially methylated regions were identified in cells overexpressing CRISPRme and gRNAs. These off-target differentially methylated regions were predominantly found in promoter regions, 5' un-translated regions, CpG islands, and DNase I hypersensitivity sites. Through chromatin immunoprecipitation with massive parallel DNA sequencing analysis, we further revealed that these off-target DMRs were weakly correlated with dCas9 off-target binding sites. Using qPCR, fluorescent reporter cells and RNA sequencing, we also found that CRISPRme can mediate transient inhibition of gene expression. However, this effect appears to result from dCas9-mediated interference with transcription rather than *de novo* DNA methylation of the promoter investigated.

Conclusion

Our results prove that CRISPRme allows for efficient RNA-guided methylation of endogenous CpGs. Still there is significant off-target methylation indicating that further improvements of the specificity of CRISPR-dCas9 based DNA methylation modifiers are required.

Key words

DNA methylation – CRISPR – Cas9 – DNMT3A – DNMT3B – dCas9 – specificity – off-targets – genome wide

Background

Owing to its simplicity, efficiency and potential for multiplicity, the type II Clustered Regularly Interspaced Short Palindromic Repeats (CRISPR) and CRISPR-associated protein 9 (Cas9) along with engineered variants have been widely used for genome and epigenome editing across many species [1-5]. The Cas9 protein is guided to a specific genomic locus containing a protospacer adjacent motif (PAM) by a small single guide RNA (gRNA), which contains programmable guide sequences (typically 20 nt) and a conserved scaffold sequence [1]. By introducing double mutations (D10A and H840A) to the SpCas9 protein (dCas9) to inactivate its catalytic activity and fusing functional effectors to the C terminus of dCas9, the applications of CRISPR/Cas9 are expanded to regulation of gene expression (CRISPRa and CRISPRi) [6-8], targeted DNA purification [9], visualization of specific gene regions [10], and acetylation or methylation of chromatin components [11, 12].

Genome-wide studies have revealed fundamental functional roles of DNA methylation as well as associations between aberrant DNA methylation and human diseases including cancer [13, 14]. Methylation of cytosine residues (5mC) in the mammalian genome mainly occurs at CpG dinucleotides and is normally associated with repression of gene expression in promoter regions. Currently, insights into DNA methylation-associated biological processes are largely based on correlative data. Methods have been developed to methylate desired gene loci selectively by fusing programmable DNA binding proteins (zinc finger proteins (ZFs) or transcription-activator-like effectors (TALEs)) to DNA methyltransferases [3-9]. However, the laborious generation of ZFs- and TALEs hampers their broader applications. Due to its simplicity and programmability, engineering *S. pyogenes* dCas9 for targeted DNA methylation by fusing dCas9 to the catalytic domain of mammalian DNA methyltransferases has the potential to provide a tool for easily programmable DNA methylation [15, 16].

Currently, genome-wide characterization of the specificity of dCas9-based epigenetic modifiers is still lacking. To gain more insights into the efficiency and specificity of targeted DNA methylation by CRISPR gRNA-guided dCas9 methyltransferases (hereafter denoted CRISPRme), we used quantitative bisulfite pyrosequencing, whole genome bisulfite sequencing, and ChIP-seq to investigate the characteristics of CRISPRme-mediated DNA methylation in human cells.

Methods

Cell Culture

Human embryonic kidney HEK293T cells (ATCC) were cultured in Dulbecco's modified Eagle's medium (DMEM, Life Technologies), 10% fetal bovine serum (Sigma), 1% penicillin-streptomycin (Sigma), 1X GlutaMAX (Life Technologies) at 37 degree, 5% $CO2$.

CRISPRme plasmids

The dCas9 coding sequence was derived from pHR-SFFV-dCas9-BFP-KRAB (Addgene ID 46911) (a gift from Stanley Qi & Jonathan Weissman). The catalytic domains of DNMT1, DNMT3A and DNMT3B were PCR-amplified from pcDNA3/Myc-DNMT1 (Addgene ID 36939), pcDNA3/Myc-DNMT3A (Addgene ID 35521) and pcDNA3/Myc-DNMT3B1 (Addgene ID 35522) (a gift from Arthur Riggs), respectively. The DNMT3A (E752A) and DNMT3B (E697A) catalytically inactivating mutations were introduced by site-directed mutagenesis. All CRISPRme plasmids described in this study (**Supplementary Table 1)** have been validated by Sanger sequencing and will be publically available through Addgene (https://www.addgene.org/Yonglun_Luo/),

CRISPRme gRNA design

Based on the observation that CRISPRme could efficiently methylate the CpGs flanking the target sites, a web-based gRNA designing tool (***CRISPRme gRNA finder*, http://luolab.au.dk/views/gRNA.cgi)** was developed to facilitate CRISPRme-based gRNA design. All updates regarding the CRISPRme protocol are available on the website (http://luolab.au.dk/). All gRNA sequences are listed in **Supplementary Table 1**.

Transfection and enrichment of CRISPRme expressing cells

Unless stated elsewhere, cells were transfected with gRNAs (total 500 ng) and a CRISPRme expression vector (500 ng) in six-well plates using X-tremeGENE 9 DNA transfection reagent (Roche). For single gRNA or pUC19 control transfections, the amount of plasmid added was equivalent to the total amount of plasmid added for multiple gRNA transfections. For BFP-based enrichment, cells were harvested 48 hours after transfection, and CRISPRme-expressing cells were sorted by FACS. Briefly, transfected cells were harvested by trypsinization, washed twice with 2% FBS-PBS, and re-suspended in 500 µL 2% FBS-PBS. Cells were stained with Propidium Iodide (PI) before sorting. PI negative and BFP positive or negative cells were sorted with a 4 Laser BD Facs Aria III instrument. For antibiotic-based enrichment, 24 hours after transfection, transfected cells were cultured in medium containing blasticidin (10 ug/mL) and puromycin (10 ug/mL) for 48 hours and harvested for gene expression or DNA methylation analysis. All transfections were performed in at least two independent experiments.

Quantitative PCR (qPCR)

Total RNA was extracted from cells with the RNeasy Plus Mini Kit (Qiagen, 74136) according to the manufacturer's instructions and quantified using a Nanodrop 1000 Spectrophotometer. The first strand cDNA was synthesized from 100-500 ng total RNA with the iScript cDNA synthesis kit (Bio-Rad, 170-8891) following the manufacturer's instructions. qPCR was performed in triplicate for each sample, using the Light Cycler 480 SYBR Green I Master mix (Roche Life Science, 04887352001) and a Light Cycler 480 qPCR machine. Each qPCR reaction contained 1 µL cDNA template (5 times diluted), 7.5 µL qPCR Master mix (2X), and 5 pmol of each qPCR primer in a total volume of 15 µL. The following qPCR program was used for *uPA, TGFBR3*, *CDKN2A* and *GAPDH*: 1 cycle at 95 °C for 5 min; 45 cycles at 95 °C for 10s, 57 °C for 10s, and 72 °C for 10s during which the fluorescence signal was measured. The final product was subjected to melting curve analysis. Primers for qPCR are listed in **Supplementary Table 1**. Relative gene expression was calculated using the $2^{-\Delta\Delta CT}$ method by first normalizing to the internal control *GAPDH* (ΔCT) and then calibrating to the transfection control pUC19 (ΔΔCT) [35].

DNA methylation analysis by bisulfite pyrosequencing with PyroMark Q24

Genomic DNA was extracted using the DNeasy Blood & Tissue Kit (Qiagen, 69506) according to the manufacturer's instructions. A total of 200 ng of genomic DNA was bisulfite treated using the EpiTect Bisulfite Kit (Qiagen, 59104) according to the manufacturer's instructions. This converts unmethylated cytosines to uracils. The bisulfite converted DNA was eluted with 20 µL elution buffer provided by the kit. Bisulfite PCR reactions for all genes described in this study were performed in a 25 µl volume containing 0.15 µl Hotstar Taq polymerase (5U/µl) (New England Biolabs, M0495L), 2.5 µl 10xStandard buffer, 0.5 µl of 10 mM dNTPs, 1.0 µl of each primer (10 µM) and 1.5 µl bisulfite converted genomic DNA. PCR was performed under the following conditions: 95 ˚C for 5 min followed by 45 cycles of 94 ˚C for 30 sec, 58 ˚C for 1 min, and 72 ˚C for 45 sec, and, finally, by 72 ˚C for 7 min. 4 µL PCR product was checked by gel electrophoresis. Pyrosequencing was performed with the PyroMark Q24 Advanced Reagents (Qiagen, 970922) using 20 µL PCR product from the bisulfite treated DNA and 20 µL sequencing primer (0.375 µM) according to the PyroMark Q24 CpG protocol. The general degree of cytosine methylation was determined by pyrosequencing of the bisulfite converted genomic DNA, using the PyroMark Q24 Advanced system (Qiagen).

DNA methylation analysis by bisulfite Sanger sequencing

Bisulfite converted DNA was used as template for PCR amplifications with the BS specific PCR primers listed in **Supplementary Table 1**, using the DreamTaq DNA Polymerase (Life Technologies, EP0701). PCR products were gel purified, sub-cloned in a TA-cloning vector (Life Technologies, 450030) and transformed into chemically competent *E.coli* cells. Cell clones were

manually picked, sub-cultured in 250 ul LB medium overnight, lysed, subjected to Sanger sequencing and analyzed by BISMA [36].

Fluorescence reporter cell assay

Five stable fluorescence reporter cell clones were established by randomly inserting various copies of the CMV promoter-driven mCherry expression cassette into HEK293T (pLV-mCherry was a gift from Pantelis Tsoulfas, 36084). Cells were transfected separately with each CRISPRme expression vector (50 ng) and gRNAs (total 50 ng) in 24-well plates. One-third of the transfected cells were seeded to a new plate every 2-3 days and the remainder used for flow cytometry analysis. Median mCherry intensity was measured with the BD LSRFortessa™ cell analyzer (FACS CORE facility, Aarhus University). Identical instrument settings and control beads were applied during the time course experiment to ensure valid comparison across different time points. 20,000 events were recorded for each sample. Flow cytometry data was analyzed using the Flowjo software.

Immunostaining

48 hours after transfection, cells were fixed with freshly-made 4% PFA for 15 min at room temperature, followed by three washes with DPBS. Cells were permeabilized in 0.3% Triton X-100 DPBS for 10 min and blocked in 5% goat serum-DPBS for 30 min. Cells were incubated with a primary rabbit anti-HA-tag antibody (C29F4, Cell Signaling 3724, 1:1000) overnight, followed by secondary antibody staining with Alexa Fluor 555 donkey anti-rabbit IgG (A-31572, Life technologies) at room temperature for 2 hours. Images were obtained with a confocal microscope (LSM710, Carl Zeiss).

Southern blot analysis

Genomic DNA (15 µg) from the fluorescence reporter cells was digested with *EcoRI* restriction enzyme overnight and then analyzed by gel electrophoresis with vacuum blotting. Primers for generating the mCherry probe are listed in **Supplementary Table 1**. Probe labelling was performed using the Prime-It II Random Primer Labeling Kit according to the manufacturer's instructions. Pre-hybridization and hybridization steps were carried out at 42 °C. Excess probe was washed from the membrane with SSC buffer, and the hybridization pattern was visualized on X-ray film by autoradiography.

RNA sequencing

Integrity and quantity of extracted RNA was evaluated with an Agilent 2100 Bioanalyzer according to the manufacturer's instructions. After DNase I treatment, mRNA was isolated with Oligo (dT) magnetic beads. Fragmentation buffer was added to generate short fragments of

mRNA. cDNA was synthesized using the mRNA fragments as templates, resolved with EB buffer for end repair and ligated with adaptors. After size selection and purification by agarose gel electrophoresis, cDNA with sizes of approximately 240 bp were used for PCR amplification (12 cycles) and library construction. Libraries were sequenced on an Ion Proton platform (>30 million reads per sample). Sequencing reads that contained low quality, adaptor, and/or short read sequences (length < 30nt) were filtered out before mapping. tmap was used to align the clean reads to the hg19 UCSC RefSeq (RNA sequences, GRCh37). No more than 3 mismatches were allowed in the alignment. Gene expression levels were calculated by transforming uniquely mapped transcript reads to TPM (transcript per million) [37]. Differentially expressed genes were defined as genes with a Benjamini and Hochberg–adjusted P value (FDR) ≤ 0.001 and fold change ≥ 2.

ChIP-seq

HEK293T cells were transfected with dCas9-BFP-DNMT3A and five *uPA* gRNAs. 48 hours after transfection, transfected cells were subjected to ChIP with a commercially available kit ChIP-IT Express Enzymatic (53009-AF, ActivMotif, distributed by Nordic Biolabs) and an anti-HA tag antibody (C29F4, Cell Signaling) according to the manufacturer's instructions. Next generation sequencing libraries were prepared for Chip and input samples, sequenced with SE50 on Illumina HiSeq2500. Clean reads were mapped to human genome hg19 using SOAP2 with the parameter "-p 4 -v 2 -s 35". Unique mapping reads was sampled randomly and equally (62723057 reads). Peaks were called using MACS with P value 1e-3. Sequence motifs enriched within 70 bp of peak summits were identified using MEME-ChIP.

WGBS library preparation and sequencing

Genomic DNA was fragmented by sonication to a mean size of 250bp using a Bioruptor (Diagenode, Belgium). Bisulphite conversion was conducted with the EZ DNA Methylation-Gold kit (ZYMO). WGBS libraries were sequenced on Illumina HiSeq X Ten. After filtering out adaptor and low-quality reads, a total of 953.7Gb 150bp paired-end clean data was generated. An average of 106Gb clean data was obtained for each sample. Clean reads were aligned to the human reference genome (hg19) by BSMAP(v2.74) with the parameter "-u -v 5 -z 33 -p 6 -n 0 -w 20 -s 16 -r 0 -f 10 -L 140" [38]. Only the CpG sites with read depths >=4 were taken into consideration for DNA methylation level calculation. The 48502 bp lambda DNA was used as an extra reference for calculating the bisulphite conversion rate. Nearly complete (>99%) bisulfite conversion was documented in all libraries.

Identification of differentially methylated regions (DMRs) resulted from CRISPRme

The bioconductor package DSS was used to identify DMRs with the parameter "delta >=0.1, pvalue <= 0.01, CpG sites >= 3, DMR lenth >= 10 bp, smoothing window 100 bp". Since expressing high amount of dCas9-BFP-DNMT3A and either uPA or TGFBR3 gRNAs caused the highest de novo on-target methylation, we reasoned that the authentic off-target DMRs should be detected in these two comparisons. We first compared group 1 (dCas9-BFP-DNMT3A (500 ng) + uPA gRNAs (500 ng)) or group 3 (dCas9-BFP-DNMT3A (500 ng) + TGFBR3 gRNAs (500 ng)) to group 9 (pUC19 control) (Supplementary Fig 11a). Next, among the DMRs found by DSS, only hypermethylated DMRs were selected. Hypomethylated DMRs were filtered as these are most likely stochastic DMRs resulted from *in vitro* cell cultivation and manipulations.

Based on the *de novo* methylation of *uPA* and *TGFBR3* (**Fig. 7**) by CRISPRme, we reasoned that the authentic DMRs found between group 1 and group 9 should have mean methylation levels as: group 9 (pUC19) =< group 5 (dCas9-DNMT-3A only (500 ng)) =< group 7 (dCas9-DNMT-3A (50 ng) + uPA gRNAs (50 ng)) =< group 1 (dCas9-DNMT-3A (500 ng) + uPA gRNAs (500 ng)). Similarly, the authentic DMRs found between group 3 and group 9 should have a mean methylation level as: group 9 (pUC19) =< group 5 (dCas9-DNMT-3A only (500 ng)) =< group 8 (dCas9-DNMT-3A (50 ng) + TGFBR3 gRNAs (50 ng)) =< group 2 (dCas9-DNMT-3A (500 ng) + TGFBR3 gRNAs (500 ng)). We applied this methylation level-based filtering criteria to further remove potential stochastic DMRs. The remaining DMRs were subjected to all analyses as described in this study.

Analysis of 5nt-SEED-NGG motif density

The 5nt-SEED-NGG density was calculated by counting the frequency of the sequence containing the 5 nt SEED sequences preceding a NGG site on either DNA strand. The PAM density was calculated by counting the frequency of PAM sites (NGG) on either DNA strand. The median density with standard deviation is shown in the plots in fig 8, 9 and supplementary figure 12. Fisher’s exact test was conducted to compare densities between different sequence datasets.

Statistics

All values in this study were presented as mean ± standard deviation. The one-way Analysis of Variance (ANOVA) with Bonferroni multiple testing, linear regression, Wilcoxon matched-pairs signed-rank test, Fisher’s exact test and Benjamini and Hochberg–adjusted P value were used for statistical analysis. A p-value < 0.05 was considered statistically significant.

Results

Generation and validation of on- and off-target DNA methylation by dCas9 methyltransferases (CRISPRme)

In mammalian cells, DNA methylation is established by *de novo* DNA methyltransferases (DNMT3A and DNMT3B), and maintained upon replication by DNMT1 [17]. Using a similar approach as Vojta *et al.* and McDonald *et al.* [15, 16]., we generated dCas9 methyltransferases by fusing the catalytic domain of DNMT1, DNMT3A, or DNMT3B to the C-terminal end of dCas9, linked by a triple tandem repeated flexible linker (3XG4S, Gly-Gly-Gly-Gly-Ser) and a blue fluorescent protein (BFP) (**Fig. 1a and Supplementary Fig. 1a**). Expression of the fusion proteins were validated by BFP-based Fluorescence Activated Cell Sorting (FACS) (**Supplementary Fig. 1b**) and immunofluorescence staining using anti-HA tag antibody (**Supplementary Fig. 1c**).

To validate whether CRISPRme can methylate endogenous CpGs, the dCas9 methyltransferases were first targeted to the *uPA* promoter in HEK293T cells by co-expression with five gRNAs (*uPA* gRNA T1 to T5, **Fig. 1b**). The *uPA* promoter was chosen because it contains a dense CpG island that is hypomethylated in human cancer cells [18]. Cells were transfected with expression vectors for all five *uPA* gRNAs and an expression vector for dCas9-BFP-DNMT1, dCas9-BFP-DNMT3A, dCas9-BFP-DNMT3B, or dCas9-BFP-EGFP. 48 hours after transfection, cells were sorted into BFP negative (BFPn) and BFP positive (BFPp) populations by FACS. Control cells were transfected with an equivalent amount of pUC19 vector, subjected to the same FACS procedure, and BFPn cells were collected. By bisulfite pyrosequencing [19] the percentage of methylated CpGs (mCpGs) at individual CpG sites was quantified for the *uPA* promoter (uPA-MR1 and uPA-MR2 genomic regions) (**Fig. 1c**). Compared to the pUC19 transfected control cells, cells expressing *uPA* gRNAs and either dCas9-BFP-DNMT3A or dCas9-BFP-DNMT3B, but not dCas9-BFP-DNMT1 or dCas9-BFP-EGFP, had significantly higher mCpG levels (P value < 0.01, ANOVA test). This is consistent with previous reports showing that the C-terminal catalytic domains of DNMT3A and DNMT3B, but not DNMT1, are active [20, 21]. The most efficiently *de novo* methylated CpGs were located 10-50 bp upstream and downstream of the gRNA target sites. CpGs located in the gRNA binding sites were not methylated by CRISPRme, most likely because CRISPR/dCas9 binding blocks the interaction of the methyltransferase domain with the CpGs (**Fig. 1c**). *De novo* methylation by dCas9-BFP-DNMT3A and gRNAs was further validated by bisulfite Sanger sequencing (**Supplementary Fig. 1d**). We repeated this experiment three times and identified similar *de novo* methylation efficiencies of the *uPA* promoter by CRISPRme each time.

Since high frequency off-target mutagenesis has been observed in previous applications of CRISPR-Cas9 [22], we investigated the specificity of dCas9-BFP-DNMT3A and dCas9-BFP-DNMT3B. For this purpose, we repeated the *uPA* promoter methylation experiment including two additional controls: (1) cells expressing either dCas9-BFP-DNMT3A or dCas9-BFP-DNMT3B together with three scrambled gRNAs (gRNAs targeting the CMV promoter), and (2) cells expressing dCas9-BFP-DNMT3A or dCas9-BFP-DNMT3B only. Bisulfite pyrosequencing results further confirmed the *de novo* methylation of *uPA* promoter by CRISPRme and *uPA* gRNAs (**Supplementary Fig. 2a**). However, CRISPRme also caused *de novo* methylation of the *uPA* promoter with scrambled gRNAs, but to a lower extent compared with the result obtained for *uPA* gRNAs. A slight increase, although not significant, of % mCpG levels was also observed in cells expressing dCas9-BFP-DNMT3A or dCas9-BFP-DNMT3B only (**Supplementary Fig. 2a**). Taken together, this indicates the presence of non gRNA-targeted methylation by CRISPRme.

To further assess the specificity of CRISPRme, we investigated two genomic regions with sequence similarity to the *uPA* gRNA target sites: *SH2D3C* (3 gRNA mismatches) and *FAM221A* (3 gRNA mismatches) as well as the *GAPDH* promoter (9 gRNA mismatches). We did not observe significant changes in CpG methylation at the examined *SH2D3C* and *FAM221A* genomic sites in cells expressing CRISPRme relative to pUC19 control cells (**Supplementary Fig. 2b, c**). Surprisingly, several CpG sites in the *GAPDH* promoter were significantly methylated in cells expressing dCas9-BFP-DNMT3A together with either *uPA* or scrambled (CMV) gRNAs (**Supplementary Fig. 3a, b**). The same was observed, to a lesser extent, in cells expressing dCas9-BFP-DNMT3B (**Supplementary Fig. 3b**). This effect was less prominent in cells expressing dCas9-BFP-DNMT3A without gRNAs, further indicating that unspecific methylation of the *GAPDH* promoter is to some extent dependent on co-expression of gRNAs.

To investigate CRISPRme mediated methylation of another genomic locus, we generated three gRNAs targeting the transforming growth factor beta receptor 3 (*TGFBR3*) promoter. Similar on-target and off-target methylation effects as described above were observed for CRISPRme with *TGFBR3* gRNAs (**Fig. 1d-g; Supplementary Fig. 4, 5**). Our DNA methylation results collectively reveal that fusion of dCas9 to the catalytic domain of DNMT3A or DNMT3B can mediate targeted *de novo* DNA methylation. However, some off-target DNA methylation is also observed and this results from both gRNA-dependent and independent effects.

CRISPRme-mediated *de novo* DNA methylation is mediated by the methyltransferase activity of the DNMT3A and DNMT3B catalytic domains

De novo methylation by CRISPRme could be mediated either by the catalytic activity of the fused DNMT3A or DNMT3B domains, or by the recruitment of additional DNA methylation enzymes to

the binding sites facilitated by protein interactions. To elucidate the mechanism of *de novo* DNA methylation by CRISPRme, we introduced the E752A and E697A catalytically inactivating mutations [23] in the DNMT3A and DNMT3B catalytic domains, respectively. HEK293T cells were transfected with CRISPRme (dCas9-BFP-DNMT3A or dCas9-BFP-DNMT3B) or CRISPRme mutants (dCas9-BFP-DNMT3A (E752A) or dCas9-BFP-DNMT3B (E697A)) together with either *uPA* gRNAs or *TGFBR3* gRNAs. 48 hours after transfection, cells were sorted into four populations based on BFP signal intensity (1. very low: +; 2. low: ++; 3. medium: +++; and 4. high: ++++) (**Fig. 2a**). The populations represent different CRISPRme expression levels and thus allowed us to investigate the effect of CRISPRme expression levels on the efficiency of *de novo* DNA methylation. Bisulfite pyrosequencing analysis of 12 CpG sites in the *uPA* promoter region (**Fig. 2b)** and 14 CpG sites in the *TGFBR3* promoter region (**Fig. 2c**) revealed that only CRISPRme but not CRISPRme catalytic mutants cause *de novo* methylation, suggesting that *de novo* methylation by CRISPRme is mediated by the catalytic activity of the DNMT3A and DNMT3B domains (**Fig. 2b, c; Supplementary Fig. 6a**). In the FACS-sorted cells with very low BFP signal intensity, we observed an intermediate efficacy of *de novo* methylation between that of pUC19 transfected cells and the other BFP groups. The *de novo* methylation levels of CpGs were similar for the examined *uPA* and *TGFBR3* promoter regions between cells expressing low, medium or high levels of CRISPRme (**Fig. 2b, c and Supplementary Fig. 6a**).

Given the observation that CRISPRme can cause unspecific methylation of the *GAPDH* promoter, we next investigated whether controlling the CRISPRme expression level could be used to reduce off-target effects. We therefore analyzed the methylation state of the *GAPDH* promoter in cells FACS sorted according to BFP signal intensity (+, ++, +++, and ++++). Consistent with previous results, co-expression of dCas9-BFP-DNMT3A (**Fig. 3a**) or dCas9-BFP-DNMT3B (**Fig. 3b**) with either *uPA* or *TGFBR3* gRNAs significantly increased *de novo* methylation of *GAPDH* promoter CpGs compared to cells expressing CRISPRme without gRNAs or pUC19. Furthermore, titrating CRISPRme expression levels decreased unspecific methylation of the *GAPDH* promoter (**Fig. 3**). However, since *de novo* methylation of gRNA-targeted genes was also decreased by CRISPRme titration (**Fig. 2**), our results collectively suggest that altering CRISPRme expression levels cannot efficiently reduce unspecific methylation relative to targeted methylation.

To investigate global methylation levels, repetitive *LINE1* elements were investigated as they represent a surrogate marker for global DNA methylation[24]. We measured the *LINE1* 5'UTR methylation by bisulfite pyrosequencing which revealed that expression of dCas9-BFP-DNMT3A and *uPA* gRNAs did not result in significant *LINE1* methylation changes (**Fig. 3c**).

Effects of CRISPRme on gene expression

Methylation of promoter DNA can be correlated with inhibition of gene transcription. To determine whether the CRISPRme-mediated *uPA* and *TGFBR3* promoter methylation could inhibit gene expression, we measured *uPA* and *TGFBR3* mRNA levels by quantitative PCR (qPCR) in HEK293T cells expressing dCas9 fusion proteins (dCas9-BFP-DNMT1, dCas9-BFP-DNMT3A, dCas9-BFP-DNMT3B, or dCas9-BFP-EGFP) either without gRNAs or in combination with gRNAs (**Supplementary Fig. 7**). Compared to pUC19 transfection controls, *uPA* expression was similarly decreased in cells expressing *uPA* gRNAs (T2-T5) and either dCas9-BFP-DNMT3A, dCas9-BFP-DNMT3B, dCas9-BFP-DNMT1, or dCas9-BFP-EGFP (**Supplementary Fig. 7a-c**). The reduced expression thus does not appear to be due to *de novo* DNA methylation by CRISPRme as dCas9-BFP-DNMT1 and dCas9-BFP-EGFP cause decreased levels of *uPA* expression similar to CRISPRme despite not causing *de novo* DNA methylation. Similar results were observed in HEK293T cells when expressing all five *uPA* (**Supplementary Fig. 7d**) or *TGFBR3* (**Supplementary Fig. 7e**) gRNAs together with CRISPRme. These results were further validated in cells expressing CRISPRme or CRISPRme mutants with either *uPA* or *TGFBR3* gRNAs, respectively (**Fig. 4a; Supplementary Fig. 6b**).

To investigate whether longer term inhibition of gene expression can be facilitated by CRISPRme, five HEK293T fluorescent reporter cell clones carrying different copies of a CMV-mCherry expression cassette (**Supplementary Fig. 8a, b**) were generated. The reporter cells were co-transfected with three gRNAs targeting the CMV promoter and each of the dCas9 fusion proteins. As controls, the reporter cells were transfected with pUC19. BFP and mCherry signal were quantified by flow cytometry at 2, 5, 8, and 14 days after transfection. We observed that the number of CRISPRme-expressing cells peaked on day 2 and decreased gradually (**Supplementary Fig. 8c**). Maximal inhibition of mCherry levels were observed on day 5 post transfection (**Supplementary Fig. 8d-h**). Compared to other dCas9 fusion proteins, the dCas-BFP-DNMT3A fusion resulted in the highest and longest inhibition of mCherry expression in the reporter cells (four out of five clones) (**Supplementary Fig. 8d-h**). The transient and prolonged inhibition efficacy varied among the five cell clones. For example, clone 2, which has the lowest copy number of transgene, showed the highest transient and longest inhibition by dCas-BFP-DNMT3A (**Supplementary Fig. 8e)**. However, expression of mCherry was, in all clones, not significantly different from the pUC19 control after two weeks, suggesting that inhibition of gene expression by CRISPRme is not stably maintained.

To investigate whether the inhibition of gene expression is specific to the gRNA targeted genes, we conducted RNA sequencing in HEK293T cells expressing all five *uPA* gRNAs (500 ng) and CRISPRme (500 ng). A large number of genes (> 1000) were differentially expressed (FDR *P*

value < 0.001, fold change > 2) in cells expressing *uPA* gRNAs and either dCas9-BFP-DNMT3A or dCas9-BFP-DNMT3B (**Fig. 4c, d**). However, similar effects on the global transcription profile were observed in cells expressing *uPA* gRNAs with dCas9-BFP-DNMT1 or with dCas9-BFP-EGFP lacking *de novo* DNA methylation activity (**Fig. 4b, e**), suggesting that thebroad non-specific alteration of transcription is not related to the dCas9 methyltransferase activity. Since *uPA* is an important factor in regulating cell proliferation and functions, the large number of differentially expressed genes might be a result of both off-target effects and inhibition of *uPA* expression. Taken together, our results clearly indicate that inhibition of *uPA* and *TGFBR3* expression by CRISPRme and corresponding gRNAs is not primarily due to *de novo* DNA methylation of their promoters. The transcriptional repression could instead be caused by interference with the transcriptional initiation or elongation complexes by a mechanism similar to the one observed for the CRISPR interference (CRISPRi) system [8].

Generation of an alternative version of the CRISPRme system (CRISPRme2.0) with enhanced nuclear entry efficiency

Upon staining of the CRISPRme fusion proteins in HEK293T cells, it is evident that most CRISPRme protein is located in the cytoplasm rather than in the nucleus (**Supplementary Fig. 1c**). This is probably due to the large size of the fusion proteins hindering efficient nuclear entry. We therefore reduced the size of the CRISPRme fusion proteins by removing the BFP domain in order to increase the nuclear entry efficiency of CRISPRme. Furthermore, to enable enrichment of CRISPRme expressing cells, we used a system similar to the CRISPR activation (CRISPRa) system, in which transfected cells can be enriched by antibiotic selection [25].

Five expression vectors were constructed encoding the human-codon-optimized dCas9 protein flanked by two copies of an NLS and conjugated to the DNMT3A or DNMT3B catalytic domains, or DNMT3A (E752A), DNMT3B (E697A) or GFP, as well as a blasticidin resistance domain (**Fig. 5a**). In the system utilized for the experiment, the blasticidin domain is cleaved from the fusion protein upon translation via the self-cleaving 2A peptide. This can be used for drug-based enrichment of transfected cells. To distinguish the two CRISPRme systems, the previously described BFP-based version is denoted CRISPRme 1.0, and the antibiotic-based enrichment system is denoted CRISPRme 2.0.

Firstly, to evaluate the *de novo* DNA methylation efficiency of CRISPRme 2.0, HEK293T cells were transfected with *TGFBR3* gRNAs and either dCas9-DNMT3A or dCas-DNMT3B. Control cells were transfected with (1) pUC19; (2) CRISPRme 2.0 alone; (3) *TGFBR3* gRNAs and either dCas9-DNMT3A (E752A) or dCas-DNMT3B (E697A); (4) *TGFBR3* gRNAs and dCas9-EGFP (**Fig. 5a**). Analysis of the 14 CpG sites of the *TGFBR3* promoter (TGFBR3-MR1) revealed that

expressing *TGFBR3* gRNAs with either dCas9-DNMT3A or dCas9-DNMT3B resulted in *de novo* methylation of the CpGs flanking the T2 gRNA binding site (P < 0.05, ANOVA, **Fig. 5b**). 10-fold and 3-fold increases of *de novo* methylation of the *TGFBR3* promoter were achieved in cells expressing *TGFBR3* gRNAs with either dCas9-DNMT3A or dCas9-DNMT3B, respectively, compared to pUC19 or dCas9-EGFP controls (*P* value < 0.001, ANOVA)(**Fig. 5c**). Similar to the observations using CRISPRme1.0, DNMT3A (E752A) and DNMT3B (E697A) catalytic mutants in the CRISPRme2.0 system lacked *de novo* methylation capacity (**Fig. 5b, c**). However, compared to the CRISPRme1.0 (**Figure 1f**), CRISPRme 2.0 appeared to have lower on-target methylation efficiency and higher unspecific methylation (**Fig. 5b, c**). Analysis of transient and long-term *TGFBR3* expression and promoter methylation in cells expressing CRISPRme 2.0 further proved that inhibition of *TGFBR3* expression was not due to CRISPRme-mediated *TGFBR3* promoter methylation (**Fig. 5d**) and not stably maintained (**Supplementary Fig. 9**) .

Since both CRISPRme 1.0 and CRISPRme 2.0 systems use the same catalytic domain of DNMT3A, we speculated that the increased unspecific methylation by CRISPRme 2.0 was related to increased CRISPRme expression levels and/or nuclear localization efficiency. The CRISPRme 1.0 and CRISPRme 2.0 systems utilize different promoters for expression of the fusion proteins. Furthermore, the BFP tag was not included in the CRISPRme 2.0 system, which might increase nuclear entry efficiency of the fusion protein. Thus, we next investigated the potential role of differential nuclear localization and expression patterns. Using fluorescence imaging to quantify the expression level and nuclear localization of the three EGFP control plasmids: dCas9-BFP-EGFP, dCas9-EGFP, and EF1a-EGFP, our results showed that both expression and nuclear entry levels of dCas9-EGFP is significantly higher (*P* value < 0.05, ANOVA) than dCas9-BFP-EGFP (**Supplementary Fig. 10a**). Furthermore, we analyzed unspecific methylation of the *GAPDH* promoter in cells expressing dCas9-BFP-DNMT3A, dCas9-DNMT3A, or EF1a-DNMT3A (a control plasmid expressing the DNMT3A catalytic domain under transcriptional control of the EF1a promoter). Our results showed that cells expressing dCas9-DMNT3A (CRISPRme 2.0) and EF1a-DNMT3A showed notably higher mCpG levels than cells transfected with pUC19 (mean diff. mCpG level = 11.7% and 38.5%, *P* value =0.012, Wilcoxon matched-pairs signed-rank test), while the difference between cells expressing dCas9-BFP-DNMT3A (CRISPRme1.0) and pUC19 was much smaller (mean diff. mCpG level = 2.3%, *P* value = 0.012, Wilcoxon matched-pairs signed-rank test) (**Supplementary Fig. 10b**). We next measured *LINE1* 5'UTR methylation. There was no significant difference in *LINE1* 5'UTR DNA methylation between cells expressing dCas9-BFP-DNMT3A and dCas9-DNMT3A (**Supplementary Fig. 10c**). However, cells transfected with the expression vector EF1a-DNMT3A had significantly higher *LINE1* methylation levels compared to pUC19 control cells (mean diff. % mCpG = 3.1, P value = 0.028, Wilcoxon matched-pairs signed-rank test) (**Supplementary Fig.**

10c), suggesting that increased CRISPRme expression and nuclear entry is accompanied by increased off-target methylation.

Titration of dCas9-DNMT3A or gRNA transfection quantities decreases both on-target and off-target methylation

Based on the above observation, we investigated whether titration of the amounts of CRISPRme 2.0 plasmids used for transfection could improve DNA methylation specificity. Thus, we transfected HEK293T cells with a varying amount of dCas9-DNMT3A (5, 25, 50, 100, 250, and 500 ng) alone or together with *TGFBR3* gRNAs. Control cells were transfected with pUC19 plasmid in the same concentrations. We analyzed *TGFBR3* (**Fig. 6a, b**) and *GAPDH* (**Fig. 6c, d**) promoter methylation and observed that titration of dCas9-DNMT3A caused a concordant decrease in both *TGFBR3* and *GAPDH* methylation. Quantification of *TGFBR3* expression further revealed that *TGFBR3* expression decreased with increasing dCas9-DNMT3A plasmid amounts and was not related to the methylation level of the investigated *TGFBR3* promoter region (**Fig. 6e**). Next, we tested whether decreasing the gRNA expression level could minimize gRNA-dependent off-target effects. We transfected HEK293T cells with various combinations of the amounts of dCas9-DNMT3A (5, 25, and 50 ng) and *TGFBR3* gRNAs (5, 25, 50, and 500 ng) and analyzed the methylation status of the *TGFBR3* (**Fig. 6f, g**) and *GAPDH* promoters (**Fig. 6h, i**). Bisulfite pyrosequencing results showed that titration of gRNA transfection quantities decreased both on-target (*TGFBR3*) and off-target (*GAPDH*) methylation concordantly (**Fig. 6f-i**). Collectively, our results suggest that increasing the expression of dCas9-DNMT3A and gRNAs results in enhancement of both on-target and off-target DNA methylation.

Genome-wide bisulfite sequencing revealed off-target methylation by CRISPRme

We have consistently observed that expression of CRISPRme alone or together with gRNAs results in off target DNA methylation and therefore performed whole-genome bisulfite sequencing (WGBS) to further characterize off-target effects. Nine groups of HEK293T cells were generated representing cells transfected with pUC19 (control), CRISPRme1.0 only (dCas9-BFP-DNMT3A or dCas9-BFP-DNMT3B), and CRISPRme1.0 with either *uPA* gRNAs or *TGFBR3* gRNAs (**Supplementary Fig. 11a**). Using the Illumina HiSeq X platform, we generated over 100 Gb of clean data for each sample (more than 30X coverage with a 99.5% bisulfite conversion rate). This allowed us to analyze the methylation pattern at single-base pair resolution (**Supplementary Fig. 11a**). Since mainly CpG dinucleotides are subject to methylation in HEK293T cells (**Supplementary Fig. 11b**), all following analyses are based on CpG methylation in the entire genome (approximately 40,000,000 CpG sites). We firstly examined *uPA, TGFBR3* and *GAPDH* promoter methylation as revealed by WGBS in all nine groups. As illustrated in **Fig. 7**, WGBS clearly showed that the *uPA* and *TGFBR3* gRNAs could target dCas9-BFP-DNMT3A and dCas9-

BFP-DNMT3B to the *uPA* and *TGFBR3* loci and efficiently methylate CpGs flanking the gRNA binding sites. Furthermore, our WGBS data revealed that CRISPRme-mediated *de novo* methylation of *TGFBR3* and *GAPDH* promoters occurred in a much broader region surrounding the gRNA binding site than previously established.

Next, we analyzed the global DNA methylation profile in these nine groups of HEK293T cells. Consistent with the LINE1 assay, WGBS revealed that expression of CRISPRme 1.0 alone or together with gRNAs was not associated with global methylation changes (**Fig. 8a, Supplementary Fig. 12a**). Since we have only one replicate per group and stochastic methylations frequently occur in cancer cells during cultivation [26], we firstly used the DSS statistical method, developed by Wu et al [27], to identify the authentic off-target differentially methylated regions (DMRs) caused by expression of CRISPRme with gRNAs. Initially, we compared group 1 (dCas9-BFP-DNMT3A and uPA gRNAs, 500 ng) or group 3 (dCas9-BFP-DNMT3A and TGFBR3 gRNAs, 500 ng) to group 9 (pUC19 control) using DSS. Over 10,000 DMRs were identified (group 1: hypermethylated DMR (hyper-DMR) = 16169, hypomethylated DMR (hypo-DMR) = 11172; group 3: hyper-DMR = 12500, hypo-DMR = 11996). The hypo-DMRs most likely resulted from stochastic methylation and are not included in the following analysis. Secondly, based on the *de novo* methylation efficiency of *uPA* and *TGFBR3*, we applied an extra filtering step to exclude potential stochastic DMRs (described in the methods). Following this filtering, we identified two clusters of hyper-DMRs caused by expressing dCas9-BFP-DNMT3A together with either uPA gRNAs (denoted uPA-DMRs, n = 6236, **supplementary Table 1**) or TGFBR3 gRNAs (denoted TGFBR3-DMRs, n = 4159, **supplementary Table 2**). The majority of the DMRs were < 120 bp containing 3-6 CpGs (**Supplementary Fig. 13**). The average methylation level of these hyper-DMRs increased significantly from pUC19 control cells to cells expressing CRISPRme only, low amounts of CRISPRme and gRNAs, and high amounts of CRISPRme and gRNAs (**Fig. 8b-c, Supplementary Fig. 12b-c**), in a pattern similar to the on-target methylation pattern previously described (**Fig. 7**). Our WGBS result revealed that expression of CRISPRme together with gRNAs can result in substantial off-target methylations throughout the genome.

Characteristics of off-target methylation by CRISPRme

To better understand the pattern of off-target methylation by CRISPRme, we stratified each DMR according to its localization in particular types of genomic regions, including promoters, coding sequences (CDS), introns, 5' un-translated regions (5'UTR), 3' UTR, CpG islands (CGI), CGI shores, Alu sequences, LINE1 sequences, and LINE2 sequences. Our results showed that both uPA-DMRs (**Fig. 8d**) and TGFBR3-DMRs (**Supplementary Fig. 12d**) were predominantly enriched in promoters, 5'UTR and CGI. Consistent with this finding, a metaplot of average

methylation levels for all genes before the DSS call also showed that transcription start site flanking regions (overlapping with promoters and 5'UTR) were hypermethylated in cells expressing CRISPRme and gRNAs (**Supplementary Fig. 14**).

Since dCas9 preferentially binds open chromatin regions [28], we further analyzed DNase I hypersensitivity regions based on ENCODE data from HEK293T cells (GEO#: GSM1008573) and quantified the average methylation level in DNase I hypersensitivity sites (DHS) (as an indication of sites with an open chromatin state). The DHS flanking regions (1 kb upstream and downstream) were used as a control. Compared to cells transfected with pUC19, cells expressing CRISPRme alone or together with gRNAs had significantly higher mCpG levels in the DHS sites (*P* value < 0.05; Wilcoxon matched-pairs signed-rank test) (**Fig. 8e, Supplementary Fig. 12e**). Whereas, the mCpG level was slightly decreased in the DNS site flanking regions (representative of less open chromatin regions with high background methylation levels). Compared to a background control sample containing the same number of randomly selected, similar-sized genomic windows as the DMRs, both uPA-DMRs and TGFBR3-DMRs were significantly enriched in DHS (P value < 1e-300, Fisher's exact test, **Fig. 8f** and **Supplementary Fig. 12f**).

Previous studies have discovered that complimentary base pairing between gRNA guide sequences and the PAM-proximal 5nt region (5ntSEED-PAM) is crucial for off-target binding [28, 29]. We assessed the density of individual gRNA 5ntSEED-PAM sequence (5'-**NNNNN**NGG-3') in the hypermethylated DMRs. For each DMR, we included the 100-bp flanking sequences when calculating the presence of 5ntSEED-PAM sequence density. This is based on the previous observation that CRISPRmeonly methylates CpGs flanking the gRNA binding site. We consistently observed significant enrichment of 5ntSEED-PAM sequences for all gRNAs in these DMRs compared to the background (**Fig. 8g, Supplementary Fig. 12g**).

CRISPRme-mediated hypermethylated DMRs are weakly correlated with off-target binding

To investigate whether CRISPRme-mediated off-target methylation is related to unspecific DNA binding by dCas9 and gRNA, we studied off-target binding sites in HEK293T cells expressing dCas9-BFP-DNMT3A and *uPA* gRNAs using ChIP-seq. 7754 enriched peaks (P value < 0.001, **Supplementary File 15**) were identified by ChIP-seq. These were scattered throughout the genome and mainly enriched in intron, 5'UTR, LINE 2 and DHS genomic regions (**Fig. 9a, b**). Using MEME motif scanning of ChIP peaks [30], we identified the most significant motif GAGAGGGAGNGG (P = 1.0e-318). This motif is identical to the 9-bp sequence immediately upstream of the PAM (NGG) site of *uPA* gRNA T2 (GAGCCGGGCGG**GAGAGGGAG(GGG)**) (**Fig. 9b**), suggesting that T2 is dominant compared to other *uPA* gRNAs in mediating off-target

binding. Analysis of 5ntSEED-PAM sequence density further confirmed that uPA T2 binding sites were over-represented in the ChIP peaks.

We next analyzed the correlation between the ChIP peaks and the uPA-DMRs (including the flanking 100 bp of each DMR). We found that there seems to be an increased overlap between ChIP peaks and uPA-DMR (+ flanking 100 bp) compared to the background control. However, this increase is not statically significant (P = 0.09, Fisher's exact test). Since the average methylation level of all ChIP peak regions exceeds 60% (**Supplementary Fig. 15**), and this may partially explain why there is a low correlation between ChIP peaks and DMRs given potential functional difficulty in further increasing the methylation level. Furthermore, ChIP-seq only identified sites that CRISPRme binds to strongly, whereas CRISPRme could potentially methylate CpGs that lie within the active regions that CRISPRme interrogate momentarily.

Discussion

Our results demonstrate that fusing the catalytic domain of human methyltransferases (DNMT3A and DNMT3B) to dCas9 can be used to program DNA methylation in human cells. Since the dCas9 methyltransferases are targeted to a specific genomic locus simply by a small gRNA, the CRISPRme system is more convenient than ZF- or TALE-based methyltransferases [23, 31, 32]. Recently, Vojta *et al.* and McDonald *et al.* reported that directly fusing the catalytic domain of DNMT3A to dCas9 could be used to induce DNA methylation at specific loci in HEK293T cells [15, 16], a method similar to the CRISPR2.0 system (dCas9-DNMT3A) in our study. Additionaly, Peter *et al*. showed that the dCas9-DNMT3A-DNMT3L fusion can further improve *de novo* methylation efficiency compared to dCas9-DNMT3A [33]. Here we also show that dCas9-DNMT3A can methylate CpGs flanking the gRNA binding sites in genomic loci, further proving the general applicability of dCas9 methyltransferases for targeted DNA methylation in mammalian cells. In addition, our study shows for the first time that the fusion of dCas9 to the catalytic domain of DNMT3B is also capable of inducing specific DNA methylation, although the efficiency is lower than that of DNMT3A. Furthermore, in this study, we have generated the FACS-based CRISPR1.0 system, which provides alternative tools for programmable DNA methylation. Together with the reported systems, the CRISPRme systems reported in this study further broadens the availability and applicability of CRISPR-based reprogramming of DNA methylation. Based on the observation that CRISPRme can efficiently methylate the flanking CpG sites from the gRNA binding site, we developed an open-source web-based gRNA designing tool for CRISPRme gRNAs (http://luolab.au.dk/views/gRNA.cgi).

On the basis of extensive gene-specific and whole-genome bisulfite sequencing (WGBS), we identified novel off-target methylation characteristics that appear to be predominantly enriched in

promoter, 5'UTR, CGI, and open chromatin regions. Since most of these genomic regions are hypomethylated in HEK293T cells (**Fig. 8e, Supplementary Fig. 12e, 13**), it is not surprising that the off-target DMRs were enriched in these regions. While for other genomic regions which already have methylated cytosine, it is not possible for CRISPRme to methylate them further. Our study also revealed the gRNA-dependent off-target methylation, which is consistent with the observations of McDonald *et al.* and Vojta *et al.* [15, 16]. Additionally, we have discovered that even in the absence of gRNAs, expression of the dCas9 methyltransferase construct alone can cause unspecific DNA methylation. Our WGBS results show that CRISPRme can cause some off-target DNA methylation throughout the genome. Our study is the first to characterize the on-target and off-target DNA methylation characteristics by dCas9 methyltransferases on a genome-wide scale with single-base resolution.

As is the case for other CRISPR/dCas9-based epigenome engineering technologies [11, 12], improvement of CRISPRme specificity to minimize the gRNA-dependent and gRNA-independent off-target activity is crucial for future applications of the technology. McDonald, et al., has observed significant reduction in off-target methylation using DOX inducible dCas9-DNMT3A-CD. Consistent with these findings, we found that reducing the CRISPRme and gRNA expression levels, as well as lowering the CRISPRme nuclear entry efficiency, can reduce off-target methylation. However, this approach also reduced on-target methylation levels accordingly, this may not represent a plausible way of increasing the specificity of the system. Thus, new approaches should be developed to reduce off-target methylation to insignificant levels, while maintaining sufficient on-target methylation efficiencies. The results presented in this study highlight the importance of extensive control experiments in subsequent experiments, such as the use of CRISPRme mutants, scrambled gRNAs, or no gRNAs. This is necessary for reliable interpretations of correlations between specific DNA methylation events by CRISPRme and phenotypic effects.

In this study, we have observed that CRISPRme can efficiently inhibit expression of genes in human cells. However, the transient inhibition of gene expression is not directly related to specific methylation by CRISPRme. A previous study reported that targeted DNA methylation by a zinc finger-based methyltransferase is not stably maintained [34]. Our time-course experiments to study the inhibition of gene expression and maintenance of CRISPRme *de novo* DNA methylation demonstrate that the established *de novo* methylation is gradually lost during *in vitro* expansion of the transfected cells. This could be the result of removal of the *de novo* established epigenetic marks, dilution of the CRISPRme expression plasmids, and/or negative selection of the cells expressing CRISPRme. We also realized that, in this study, DNA methylation and gene expression were conducted in cells transiently transfected with CRISPRme expression plasmids,

which would lead to overexpression of the CRISPRme proteins. Thus, future studies should be conducted in cells stably (or conditionally) expressing one copy of CRISPRme to minimize off-target methylation.

Conclusions

The two CRISPRme systems presented here and other dCas9 fusion protein systems described previously [11, 12, 15, 16] provide useful tools for targeted epigenome editing. Continued improvement of the specificities of these systems and combining tools to enable simultaneous modification of multiple histones and DNA loci will enable more precise and stable regulation of gene expression. Such CRISPR gRNA-guided programmable epigenetic modification tools will hopefully have broad research applications to delineate the association between specific epigenetic changes, gene-expression regulation, and phenotypes.

Availability of supporting data

RNA sequencing, WGBS, and ChIP-seq data are available from the publicly available repository (GEO).

RNA-seq: GSE74935

CRISPRme: GSE92311

WGBS: GSE92310

ChIP-seq: GSE92261

Declarations

Competing Interests Statement

The authors declare no competing financial interests.

Author contributions

L.L., L.B. and Y.L., conceived the idea.

H.Y., J.W., L.B., X.X., A.L.N., and Y.L. planned and oversaw the study

L.L., Y.Liu., J.H., F.X., T.F.D., T.S.P., B.H., L.Y., Q.Z., F.F., S.L., K.T.J. L.F., E.S., and Y.L. performed experiments and analyzed the data.

L.L., J.H., and Y.L. prepared the figures.

L.L. and Y.L. drafted the manuscript and all authors revised the manuscript.

Acknowledgements

This work was partially supported by grants from Danish Research Council for Independent Research DFF–1337-00128 (Y.L.), the Sapere Aude Young Research Talent Prize DFF-1335-

00763A (Y.L.), the Innovation Fund Denmark (BrainStem, Y.L.) and the Lundbeck Foundation: R173-2014-1105 (Y.L.); R151-2013-14439 (L.B.); R219-2016-1375 (L.L.) A.L.N. was supported by the Toyota-Foundation and the Lundbeck Foundation. FACS was performed with help from Charlotte Christie Petersen and Anni Skovbo at the FACS Core Facility, Aarhus University, Denmark.

Abbreviations

CRISPRme: CRISPR-guided DNA methyltransferase or dCas9 methyltransferase
CRISPR: Clustered Regularly Interspaced Short Palindromic Repeats
Cas9: CRISPR-associated protein 9
dCas9: Nuclease deficient Cas9 or dead Cas9
FACS: Fluorescence-activated cell sorting
WGBS: whole-genome bisulfite sequencing
CGI: CpG island
UTR: Untranslated region
DHS: DNase I hypersensitivity sites
PAM: Protospacer adjacent motif
gRNA: guide RNA
ZF: zinc finger protein
TALE: transcription-activator-like effectors
qPCR: Quantitative PCR

Reference:

1. Jinek M, Chylinski K, Fonfara I, Hauer M, Doudna JA, Charpentier E: **A programmable dual-RNA-guided DNA endonuclease in adaptive bacterial immunity.** *Science* 2012, **337:**816-821.
2. Mali P, Yang L, Esvelt KM, Aach J, Guell M, Dicarlo JE, Norville JE, Church GM: **RNA-Guided Human Genome Engineering via Cas9.** *Science* 2013.
3. Jinek M, East A, Cheng A, Lin S, Ma E, Doudna J: **RNA-programmed genome editing in human cells.** *elife* 2013, **2:**e00471.
4. Cong L, Ran FA, Cox D, Lin S, Barretto R, Habib N, Hsu PD, Wu X, Jiang W, Marraffini LA, Zhang F: **Multiplex genome engineering using CRISPR/Cas systems.** *Science* 2013, **339:**819-823.
5. Johan Vad-Nielsen LL, Lars Bolund, Anders Lade Nielsen, Yonglun Luo **Golden-Gate assembly of CRISPR gRNA Expression Array for Simultaneously Targeting Multiple Genes.** *Cell Mol Life Sci* 2016.
6. Qi LS, Larson MH, Gilbert LA, Doudna JA, Weissman JS, Arkin AP, Lim WA: **Repurposing CRISPR as an RNA-Guided Platform for Sequence-Specific Control of Gene Expression.** *Cell* 2013, **152:**1173-1183.
7. Cheng AW, Wang H, Yang H, Shi L, Katz Y, Theunissen TW, Rangarajan S, Shivalila CS, Dadon DB, Jaenisch R: **Multiplexed activation of endogenous genes by CRISPR-on, an RNA-guided transcriptional activator system.** *Cell Res* 2013, **23:**1163-1171.
8. Gilbert LA, Larson MH, Morsut L, Liu Z, Brar GA, Torres SE, Stern-Ginossar N, Brandman O, Whitehead EH, Doudna JA, et al: **CRISPR-mediated modular RNA-guided regulation of transcription in eukaryotes.** *Cell* 2013, **154:**442-451.
9. Fujita T, Fujii H: **Efficient isolation of specific genomic regions and identification of associated proteins by engineered DNA-binding molecule-mediated chromatin immunoprecipitation (enChIP) using CRISPR.** *Biochem Biophys Res Commun* 2013, **439:**132-136.
10. Chen B, Gilbert LA, Cimini BA, Schnitzbauer J, Zhang W, Li GW, Park J, Blackburn EH, Weissman JS, Qi LS, Huang B: **Dynamic imaging of genomic loci in living human cells by an optimized CRISPR/Cas system.** *Cell* 2013, **155:**1479-1491.
11. Kearns NA, Pham H, Tabak B, Genga RM, Silverstein NJ, Garber M, Maehr R: **Functional annotation of native enhancers with a Cas9-histone demethylase fusion.** *Nat Methods* 2015, **12:**401-403.
12. Hilton IB, D'Ippolito AM, Vockley CM, Thakore PI, Crawford GE, Reddy TE, Gersbach CA: **Epigenome editing by a CRISPR-Cas9-based acetyltransferase activates genes from promoters and enhancers.** *Nat Biotechnol* 2015, **33:**510-517.
13. Jones PA, Baylin SB: **The fundamental role of epigenetic events in cancer.** *Nat Rev Genet* 2002, **3:**415-428.
14. Brena RM, Costello JF: **Genome-epigenome interactions in cancer.** *Hum Mol Genet* 2007, **16 Spec No 1:**R96-105.

15. Vojta A, Dobrinic P, Tadic V, Bockor L, Korac P, Julg B, Klasic M, Zoldos V: **Repurposing the CRISPR-Cas9 system for targeted DNA methylation.** *Nucleic Acids Res* 2016.

16. McDonald JI, Celik H, Rois LE, Fishberger G, Fowler T, Rees R, Kramer A, Martens A, Edwards JR, Challen GA: **Reprogrammable CRISPR/Cas9-based system for inducing site-specific DNA methylation.** *Biol Open* 2016, **5:**866-874.

17. Law JA, Jacobsen SE: **Establishing, maintaining and modifying DNA methylation patterns in plants and animals.** *Nat Rev Genet* 2010, **11:**204-220.

18. Pakneshan P, Szyf M, Farias-Eisner R, Rabbani SA: **Reversal of the hypomethylation status of urokinase (uPA) promoter blocks breast cancer growth and metastasis.** *J Biol Chem* 2004, **279:**31735-31744.

19. Dejeux E, El abdalaoui H, Gut IG, Tost J: **Identification and quantification of differentially methylated loci by the pyrosequencing technology.** *Methods Mol Biol* 2009, **507:**189-205.

20. Gowher H, Jeltsch A: **Molecular enzymology of the catalytic domains of the Dnmt3a and Dnmt3b DNA methyltransferases.** *J Biol Chem* 2002, **277:**20409-20414.

21. Margot JB, Aguirre-Arteta AM, Di Giacco BV, Pradhan S, Roberts RJ, Cardoso MC, Leonhardt H: **Structure and function of the mouse DNA methyltransferase gene: Dnmt1 shows a tripartite structure.** *J Mol Biol* 2000, **297:**293-300.

22. Fu Y, Foden JA, Khayter C, Maeder ML, Reyon D, Joung JK, Sander JD: **High-frequency off-target mutagenesis induced by CRISPR-Cas nucleases in human cells.** *Nat Biotechnol* 2013.

23. Rivenbark AG, Stolzenburg S, Beltran AS, Yuan X, Rots MG, Strahl BD, Blancafort P: **Epigenetic reprogramming of cancer cells via targeted DNA methylation.** *Epigenetics* 2012, **7:**350-360.

24. Yang AS, Estecio MR, Doshi K, Kondo Y, Tajara EH, Issa JP: **A simple method for estimating global DNA methylation using bisulfite PCR of repetitive DNA elements.** *Nucleic Acids Res* 2004, **32:**e38.

25. Konermann S, Brigham MD, Trevino AE, Joung J, Abudayyeh OO, Barcena C, Hsu PD, Habib N, Gootenberg JS, Nishimasu H, et al: **Genome-scale transcriptional activation by an engineered CRISPR-Cas9 complex.** *Nature* 2015, **517:**583-588.

26. Landan G, Cohen NM, Mukamel Z, Bar A, Molchadsky A, Brosh R, Horn-Saban S, Zalcenstein DA, Goldfinger N, Zundelevich A, et al: **Epigenetic polymorphism and the stochastic formation of differentially methylated regions in normal and cancerous tissues.** *Nat Genet* 2012, **44:**1207-1214.

27. Wu H, Xu T, Feng H, Chen L, Li B, Yao B, Qin Z, Jin P, Conneely KN: **Detection of differentially methylated regions from whole-genome bisulfite sequencing data without replicates.** *Nucleic Acids Res* 2015, **43:**e141.

28. Kuscu C, Arslan S, Singh R, Thorpe J, Adli M: **Genome-wide analysis reveals characteristics of off-target sites bound by the Cas9 endonuclease.** *Nat Biotechnol* 2014, **32:**677-683.

29. Wu X, Scott DA, Kriz AJ, Chiu AC, Hsu PD, Dadon DB, Cheng AW, Trevino AE, Konermann S, Chen S, et al: **Genome-wide binding of the CRISPR endonuclease Cas9 in mammalian cells.** *Nat Biotechnol* 2014, **32:**670-676.
30. Bailey TL, Elkan C: **Fitting a mixture model by expectation maximization to discover motifs in biopolymers.** *Proc Int Conf Intell Syst Mol Biol* 1994, **2:**28-36.
31. Bernstein DL, Le Lay JE, Ruano EG, Kaestner KH: **TALE-mediated epigenetic suppression of CDKN2A increases replication in human fibroblasts.** *J Clin Invest* 2015, **125:**1998-2006.
32. Meister GE, Chandrasegaran S, Ostermeier M: **Heterodimeric DNA methyltransferases as a platform for creating designer zinc finger methyltransferases for targeted DNA methylation in cells.** *Nucleic Acids Res* 2010, **38:**1749-1759.
33. Stepper P, Kungulovski G, Jurkowska RZ, Chandra T, Krueger F, Reinhardt R, Reik W, Jeltsch A, Jurkowski TP: **Efficient targeted DNA methylation with chimeric dCas9-Dnmt3a-Dnmt3L methyltransferase.** *Nucleic Acids Res* 2016.
34. Kungulovski G, Nunna S, Thomas M, Zanger UM, Reinhardt R, Jeltsch A: **Targeted epigenome editing of an endogenous locus with chromatin modifiers is not stably maintained.** *Epigenetics Chromatin* 2015, **8:**12.
35. Livak KJ, Schmittgen TD: **Analysis of relative gene expression data using real-time quantitative PCR and the 2(-Delta Delta C(T)) Method.** *Methods* 2001, **25:**402-408.
36. Rohde C, Zhang Y, Reinhardt R, Jeltsch A: **BISMA--fast and accurate bisulfite sequencing data analysis of individual clones from unique and repetitive sequences.** *BMC Bioinformatics* 2010, **11:**230.
37. Wagner GP, Kin K, Lynch VJ: **Measurement of mRNA abundance using RNA-seq data: RPKM measure is inconsistent among samples.** *Theory Biosci* 2012, **131:**281-285.
38. Xi Y, Li W: **BSMAP: whole genome bisulfite sequence MAPping program.** *BMC Bioinformatics* 2009, **10:**232.

Figure captions

Fig. 1

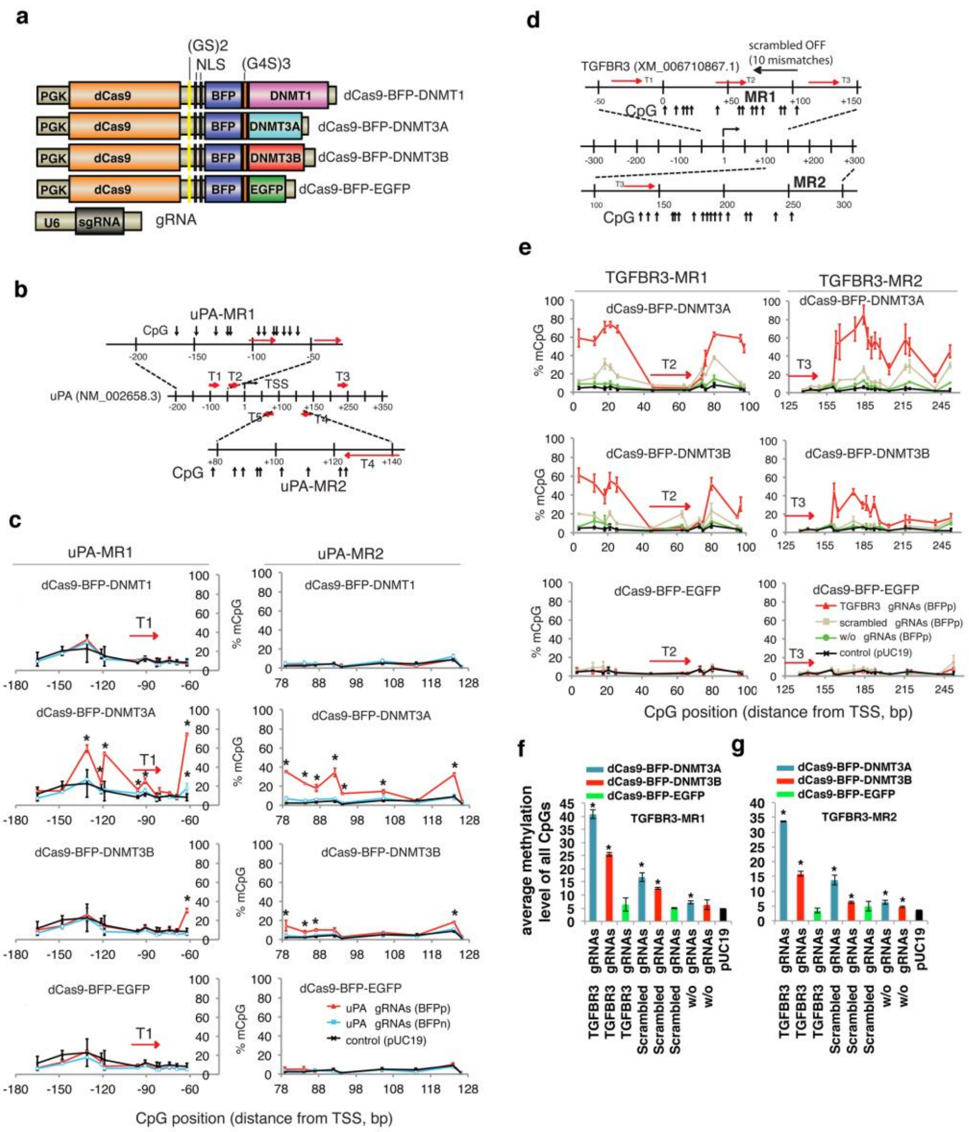

Fig. 1 ***De novo uPA*** **and** ***TGFBR3*** **methylation by RNA-guided dCas9 methyltransferases (CRISPRme1.0)**

(**a**) Schematic illustration of the CRISPRme expression vectors. The catalytically inactive dCas9 was fused to blue fluorescent protein (BFP) and either (i) a methyltransferase catalytic domain (DNMT1, DNMT3A, or DNMT3B), or (ii) green fluorescent protein (EGFP). These constructs are constitutively driven by the PGK promoter. A stretch of Gly and Ser residues (GS or G4S) are used as flexible linkers that connect the fused proteins. NLS denotes a nuclear localization signal. The gRNA chimera is expressed from the human U6 polymerase III promoter. (**b**) Schematic illustration of the *uPA* promoter with CRISPRme gRNA target sites (T1-T5), two *uPA* methylated regions (uPA-MR1, uPA-MR2) and CpGs analyzed by bisulfite pyrosequencing. Transcription

start site (TSS) is annotated according to transcript NM_002658.3 and numbers indicate distances in base pairs. (**c**) Line plots of the percentage of methylated CpGs (mCpG) in uPA-MR1 (left) and uPA-MR2 (right) in the BFP positive (BFPp; red line) and BFP negative (BFPn; light blue line) cells co-transfected with *uPA* gRNAs (T1-T5) and either dCas9-BFP-DNMT1, dCas9-BFP-DNMT3A, dCas9-BFP-DNMT3B, or dCas9-BFP-EGFP. Note that %mCpG in control cells transfected with pUC19 has been re-plotted as a reference (black line). BFPn cells include cells expressing very low level of CRISPRme. Each data point represents mean ± SD ($n = 2$-4). Asterisk (*) indicates statistical significance ($p < 0.05$) compared to the control after Bonferroni correction. (**d**) Schematic illustration of the human *TGFBR3* promoter locus, *TGFBR3* gRNA binding sites (red arrows), potential off-target binding sites (black horizontal arrows) of the scrambled gRNA, and CpG sites. (**e**) Line plots of % mCpG at the *TGFBR3* promoter in cells expressing CRISPRme with (red line) or without (green line) *TGFBR3* gRNAs, or with the scrambled gRNAs (gray line). Note that %mCpG in control cells transfected with pUC19 has been re-plotted as a reference (black line). Each data point represents mean ± SD ($n = 2$-5). (**f-g**) Bar chart of average methylation levels for TGFBR3-MR1 (**f**) and TGFBR3-MR2 (**g**) CpG sites. Values represent mean ± SD ($n = 3$). Asterisk (*) represents P value < 0.05 compared to pUC19 (ANOVA).

Fig 2

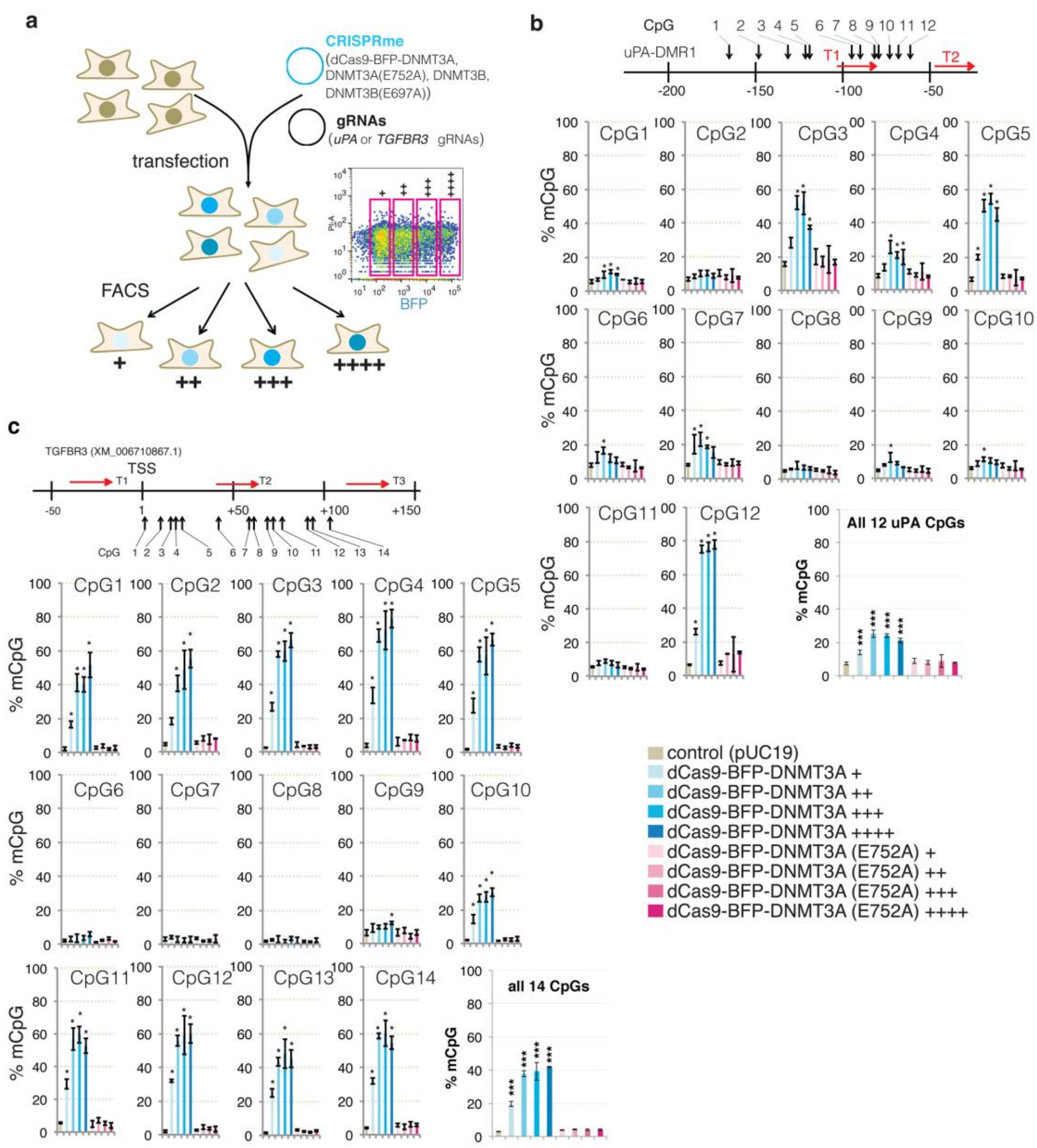

Fig. 2 CRISPRme-mediated DNA methylation is mediated by the methyltransferase catalytic activity

(**a**) Schematic illustration of the experiment. CRISPRme mutations were generated by introducing the E752A and E697A mutations in the DNMT3A and DNMT3B catalytic domains, respectively. CRISPRme-expressing cells were enriched by FACS 48 hours after transfection and sorted according to the BPF signal: +, ++, +++, ++++. Right: Representative FACS plot and gating. (**b-c**) Bar charts indicating % mCpG for individual CpG and average values of all CpG sites in the *uPA* (**b**) and *TGFBR3* (**c**) target regions. The schematic illustrations above the bar graphs show gRNA binding sites and CpG sites analyzed. Value represents mean ± SD (n = 3). Asterisk (*) indicates

statistical significance ($p < 0.05$, ANOVA) compared to the control after Bonferroni correction. Figure legend for bar graphs in (**b**) and (**c**) is presented at bottom-right.

Fig. 3

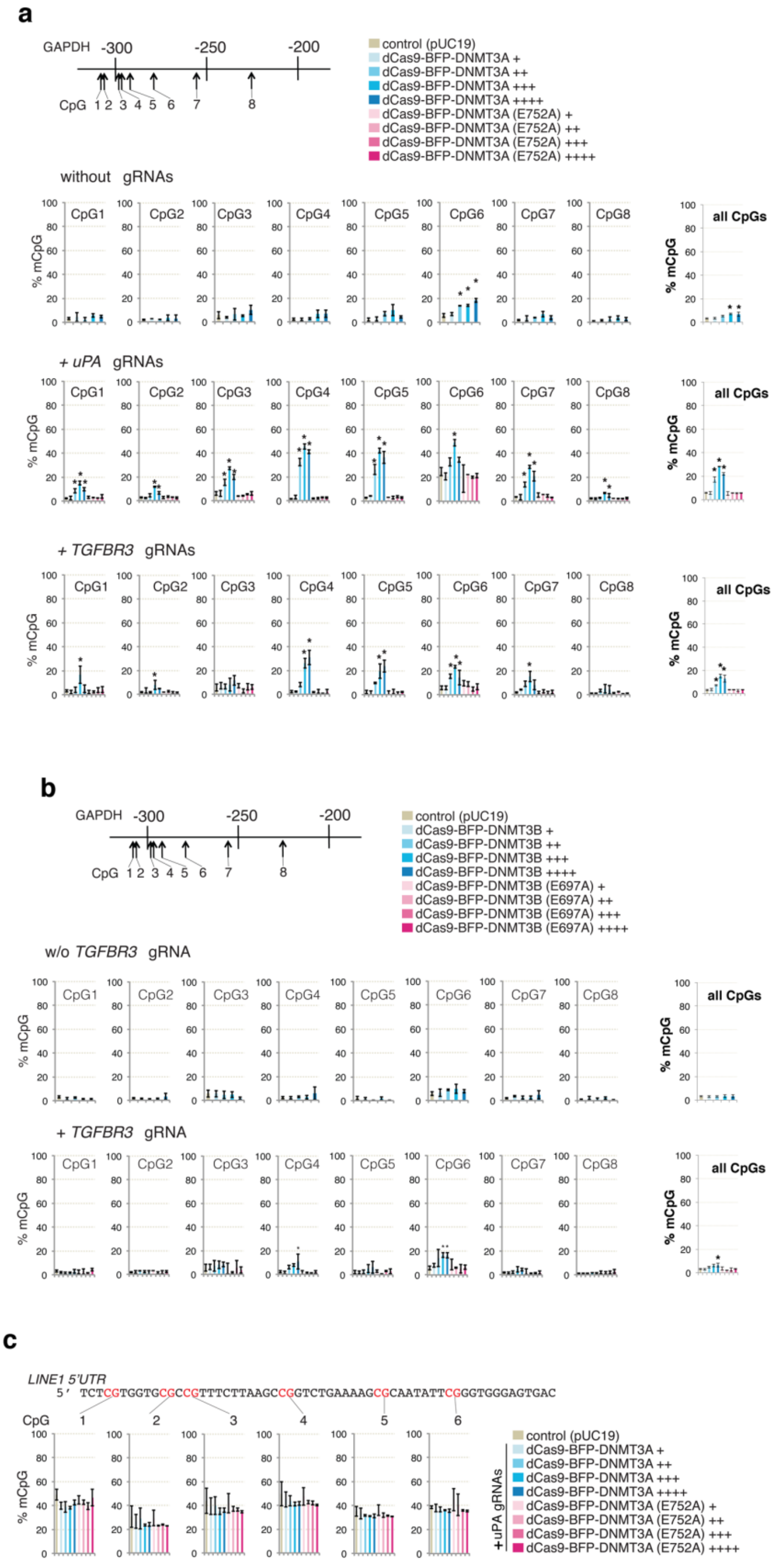
a
GAPDH -300 -250 -200
CpG 1 2 3 4 5 6 7 8
control (pUC19)
dCas9-BFP-DNMT3A +
dCas9-BFP-DNMT3A ++
dCas9-BFP-DNMT3A +++
dCas9-BFP-DNMT3A ++++
dCas9-BFP-DNMT3A (E752A) +
dCas9-BFP-DNMT3A (E752A) ++
dCas9-BFP-DNMT3A (E752A) +++
dCas9-BFP-DNMT3A (E752A) ++++
without gRNAs
+ uPA gRNAs
+ TGFBR3 gRNAs
CpG1 CpG2 CpG3 CpG4 CpG5 CpG6 CpG7 CpG8
all CpGs
% mCpG
b
GAPDH -300 -250 -200
CpG 1 2 3 4 5 6 7 8
control (pUC19)
dCas9-BFP-DNMT3B +
dCas9-BFP-DNMT3B ++
dCas9-BFP-DNMT3B +++
dCas9-BFP-DNMT3B ++++
dCas9-BFP-DNMT3B (E697A) +
dCas9-BFP-DNMT3B (E697A) ++
dCas9-BFP-DNMT3B (E697A) +++
dCas9-BFP-DNMT3B (E697A) ++++
w/o TGFBR3 gRNA
+ TGFBR3 gRNA
c
LINE1 5'UTR
5' TCTCGTGGTGCGCCGTTTCTTAAGCCGGTCTGAAAAGCGCAATATTCGGGTGGGAGTGAC
CpG 1 2 3 4 5 6
+uPA gRNAs
control (pUC19)
dCas9-BFP-DNMT3A +
dCas9-BFP-DNMT3A ++
dCas9-BFP-DNMT3A +++
dCas9-BFP-DNMT3A ++++
dCas9-BFP-DNMT3A (E752A) +
dCas9-BFP-DNMT3A (E752A) ++
dCas9-BFP-DNMT3A (E752A) +++
dCas9-BFP-DNMT3A (E752A) ++++

Fig. 3 Effect of CRISPRme on *GAPDH* and *LINE1* methylation

(a) Bar charts indicating % mCpG at individual CpGs and total % mCpG (8 CpG sites) for the *GAPDH* promoter in cells expressing different levels (BFP signal: +, ++, +++, ++++) of dCas9-BFP-DNMT3A or dCas9-BFP-DNMT3A (E752A) alone or together with either *uPA* or *TGFBR3* gRNAs. **(b)** Bar charts indicating % mCpG in the *GAPDH* promote in cells expressing different levels (BFP signal: +, ++, +++, ++++) of dCas9-BFP-DNMT3B or Cas9-BFP-DNMT3B (E697A) alone or with *TGFBR3* gRNAs. (**c**) *LINE1* 5'UTR methylation in cells expressing *uPA* gRNAs with different levels of either dCas9-BFP-DNMT3A or dCas9-BFP-DNMT3A (E752A). Cells transfected with pUC19 were used as controls. Values represent mean ± SD (n = 3). Asterisks (*) represent P value < 0.05 (ANOVA) compared to pUC19.

Fig 4

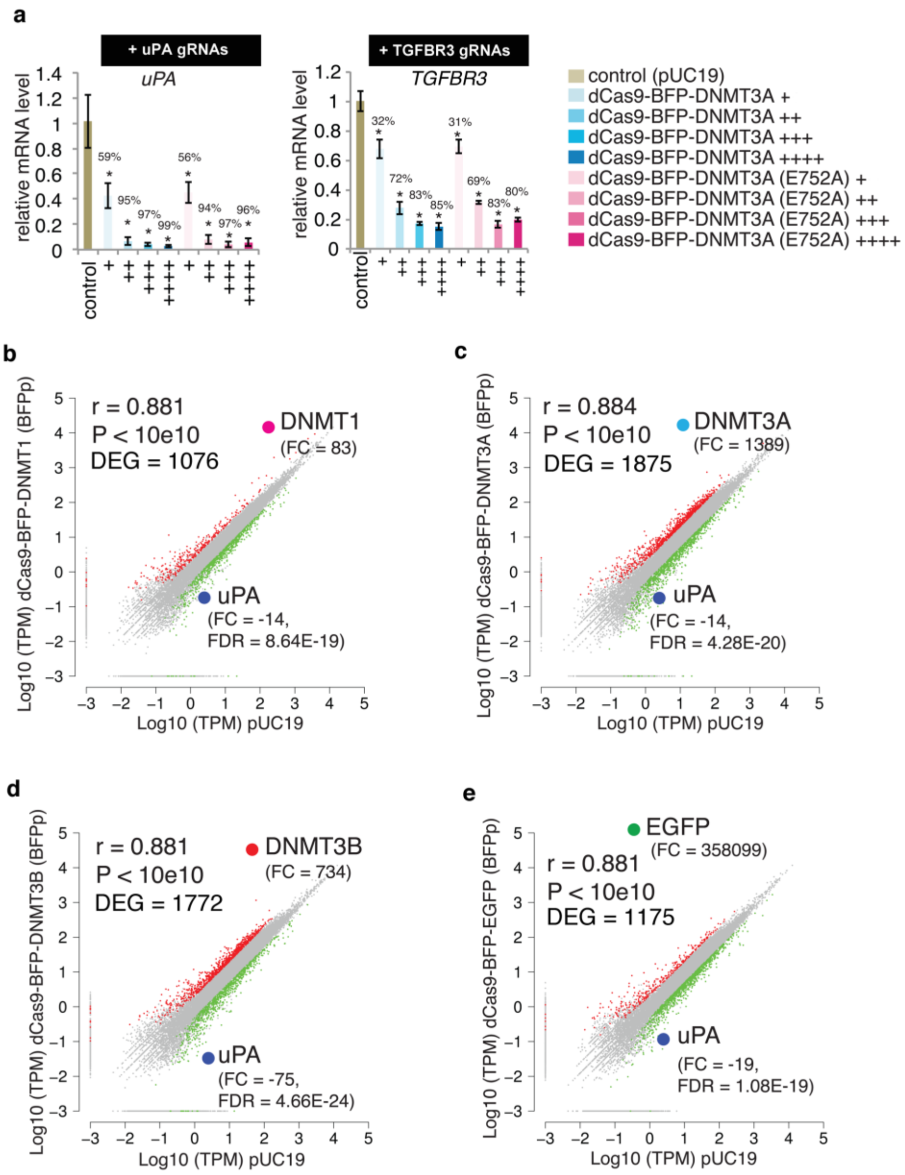

Fig. 4 Effect of CRISPRme on gene expression

(**a**) Relative gene expression levels of *uPA* (**left**) and *TGFBR3* (**right**) in cells expressing different levels of dCas9-BFP-DNMT3A or dCas9-BFP-DNMT3A (E752A), measured by qPCR and quantified as fold change compared to control cells transfected with pUC19. Bar charts depict mean change in mRNA level compared to pUC19 controls. Data represent mean ± SD (n = 3 independent transfections). Mean percentage decrease in mRNA level compared to pUC19 are presented on top of bars. Asterisks (*) represent P value < 0.05 compared to pUC19. (**b-e**) Dot plots of log10 (transcripts per million (TPM)) for all genes expressed in the BFPp cells expressing *uPA* gRNAs (T1-T5) and dCas9-BFP-DNMT1 (**b**), dCas9-BFP-DNMT3A (**c**), dCas9-BFP-DNMT3B (**d**), or dCas9-BFP-EGFP (**e**) plotted against log10 (TPM) in a pUC19 control group.

Differentially expressed genes (DEG) are marked in red (up-regulated) and green (down-regulated) (fold change >= 2, FDR < 0.001). Fold changes compared to the control (pUC19) and FDR p-values for DNMT1, *DNMT3A, DNMT3B, EGFP*, and *uPA* are shown.

Fig 5

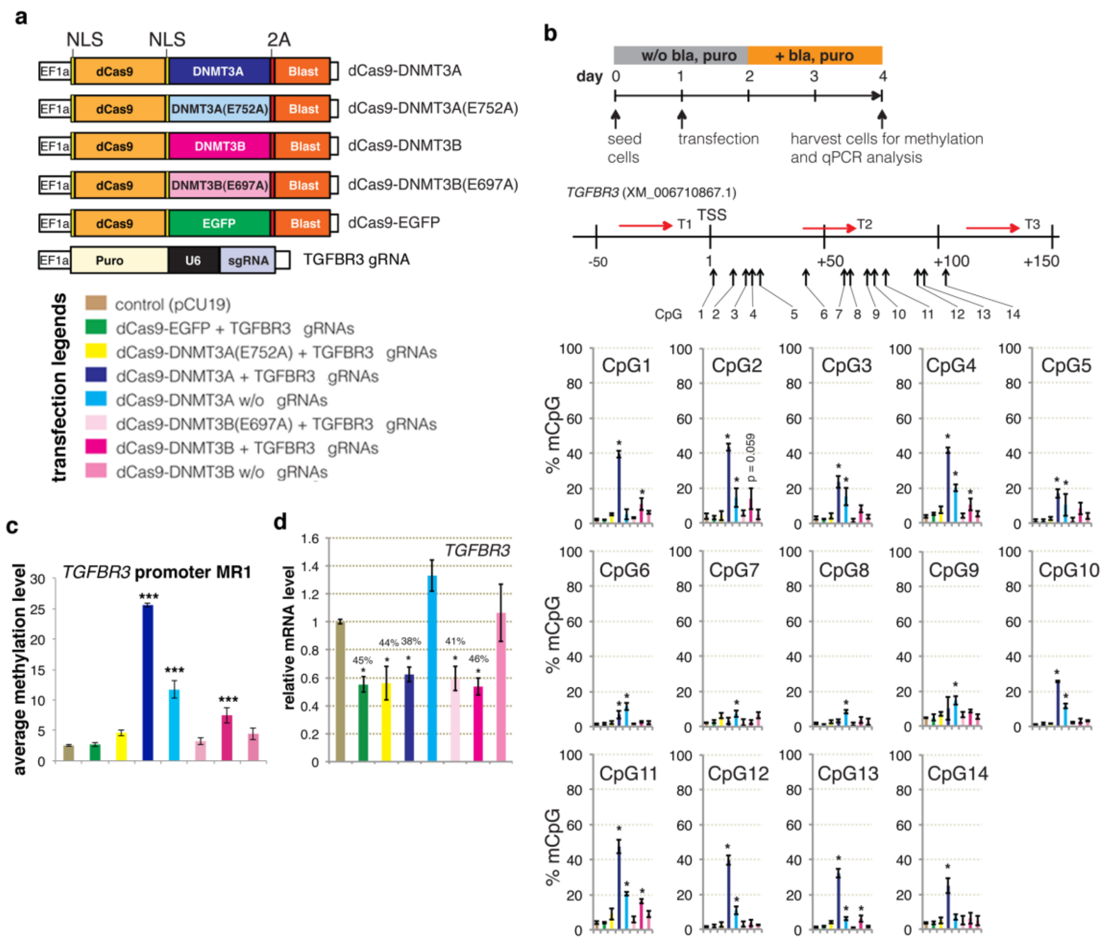

Fig. 5 Generation and validation of CRISPRme 2.0

(**a**) (**top**) Schematic illustration of CRISPRme 2.0 expression vectors encoding dCas9 flanked by nuclear localization signals (NLS) and fused to (i) a DNMT3A or DNMT3B catalytic domain, (ii) a DNMT3A (E752A) or DNMT3B (E697A) catalytically inactivated domain, or (iii) EGFP, and a blasticidin resistance gene. Expression of the fusion proteins was constitutively driven by the elongation factor-1 alpha (EF1a) promoter. (**bottom**) Legends for different transfection groups used in (**b-d**). (**b**) (**top**) Schematic illustration of the experiment and the *TGFBR3* promoter with gRNA target sites and CpGs used for subsequent analysis by pyrosequencing. CRISPRme- and gRNA-expressing cells were enriched by blasticidin (10 ug/mL) and puromycin (10 ug/mL) antibiotic selection. (**bottom**) Bar charts depicting % mCpG for individual *TGFBR3* CpG sites in

cells expressing CRISPRme2.0 or CRISPRme2.0 mutants with or without *TGFBR3* gRNAs. Cells transfected with pUC19 or a dCas9-EGFP fusion were used as negative controls. (**c**) Bar chart illustrating average methylation levels of TGFBR3-MR1. (d) Bar chart illustrating relative TGFBR3 mRNA levels compared to pUC19. % mCpG and relative gene expression values are presented as mean ± SD (n = 3 independent transfections). Asterisks represent P value < 0.05 (*) and < 0.001 (***)(ANOVA). Percentage decrease in gene expression levels compared to pUC19 are presented on top of bars in (**d**).

Fig 6

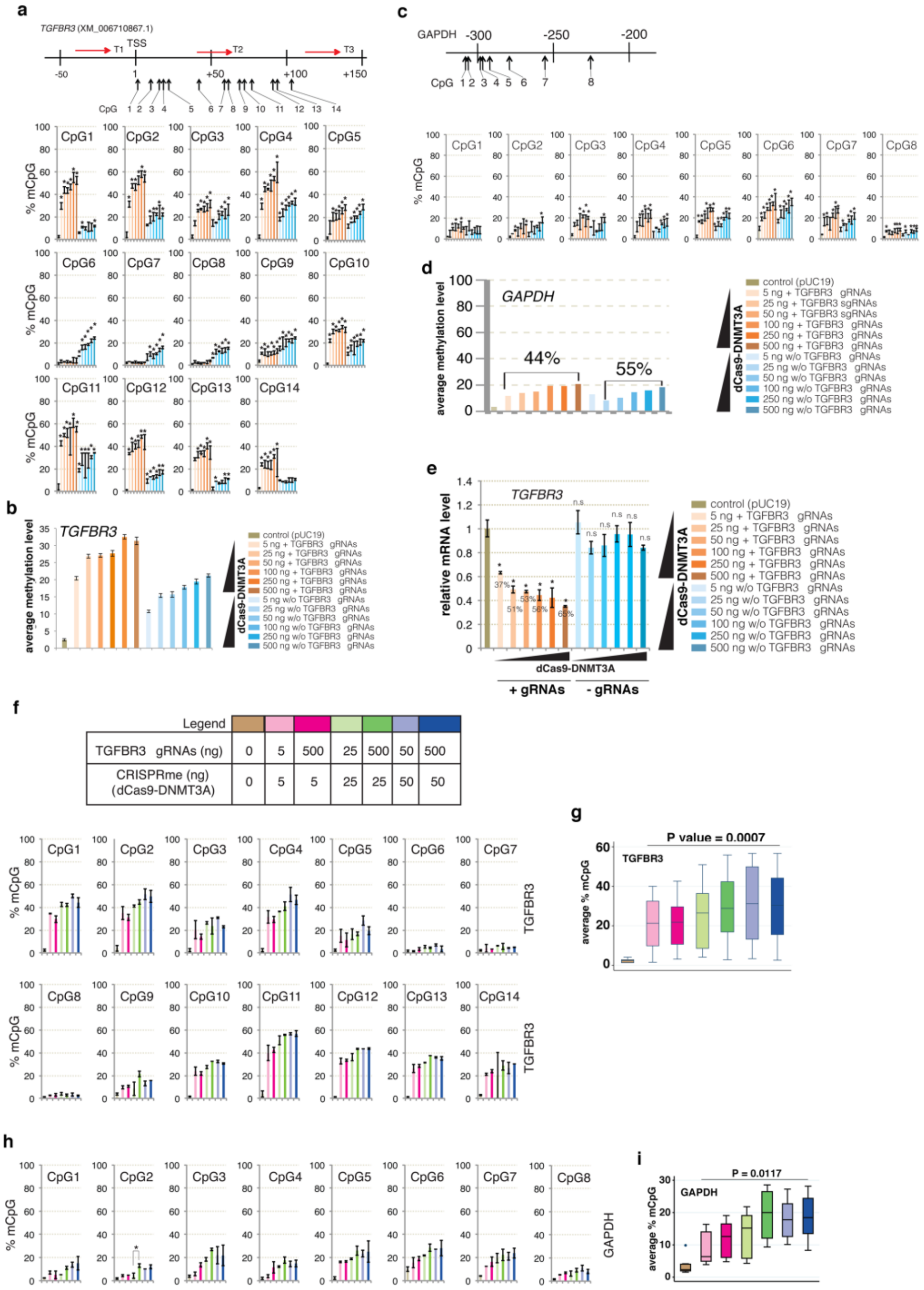

Fig. 6. Effects of titrating the amount of CRISPRme 2.0 and gRNA plasmids on on-target and off-target methylation

(**a-b**) Bar charts illustrating % mCpG levels for the 14 individual *TGFBR3* CpG sites (**a**) and the average methylation level for all 14 CpG sites (**b**) in HEK293T cells expressing various amounts (5, 25, 50, 100, 250, and 500 ng) of the dCas9-DNMT3A plasmid with or without (w/o) *TGFBR3*

gRNAs (500 ng). (**c-d**) Bar charts illustrating % mCpG levels for individual CpG sites (**c**) or average methylation levels (**d**) in the *GAPDH* promoter in cells expressing different levels (5, 25, 50, 100, 250, and 500 ng) of dCas9-DNMT3A with or without *TGFBR3* gRNAs (500 ng). Cells transfected with pUC19 were used as controls. Asterisk (*) indicates statistical significance ($p < 0.05$, ANOVA) after Bonferroni correction. (**e**) Bar charts of relative *TGFBR3* mRNA levels compared to control cells transfected with pUC19. Both CpG methylation and gene expression data represent mean ± SD (n = 3 independent transfections). Asterisk (*) indicates statistical significance (P value < 0.05, ANOVA) after Bonferroni correction. (**f**) (**top**) Transfection legend. HEK293T cells were co-transfected with various amounts of dCas9-DNMT3A (5, 25, and 50 ng) and the *TGFBR3* gRNAs (5, 25, 50, and 500 ng). (**bottom**), bar charts of % mCpG for individual CpG sites in the *TGFBR3* promoter (TGFBR3-DMR1). (**g**) Box plot of the average of % mCpG for all 14 CpG sites in the *TGFBR3* promoter for each experimental group. (**h**) Bar charts illustrating % mCpG for individual CpG sites in the *GAPDH* promoter. (**i**) Box plot of the average % mCpG for all 8 CpG sites in the *GAPDH* promoter for each experimental group. Figures (**f, h**) are plotted as mean ± SD (n = 2, independent transfections). (**g, i**) P values are from Wilcoxon matched-pairs signed-rank tests between the 5 ng and 500 ng experimental groups.

Fig. 7

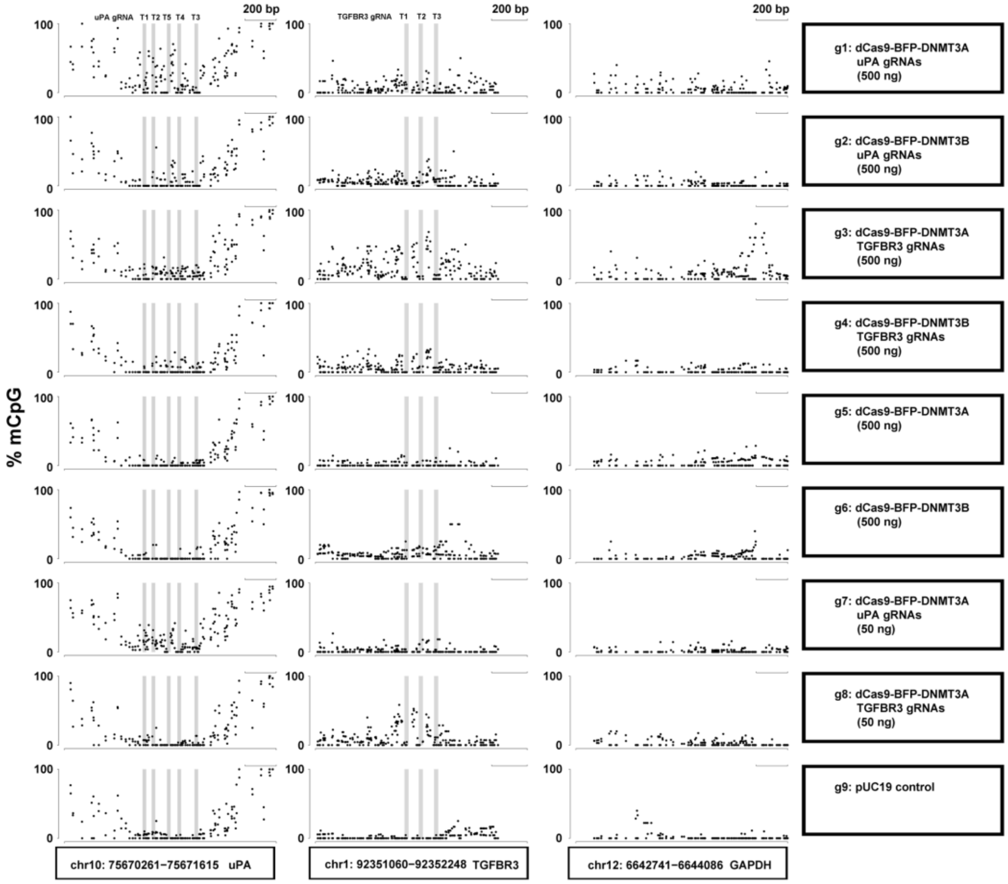

Fig. 7 De novo methylation of *uPA, TGFBR3 and GAPDH* promoters by CRISPRme 1.0. Dot plots of % mCpG for individual CpG sites in the *uPA, TGFBR3 and GAPDH* promoter regions. Each dot represents one CpG site. Right panel indicates the treatment of cells (transfected plasmids). mCpG levels were quantified by WGBS. Scale bar, 200 bp.

Fig. 8

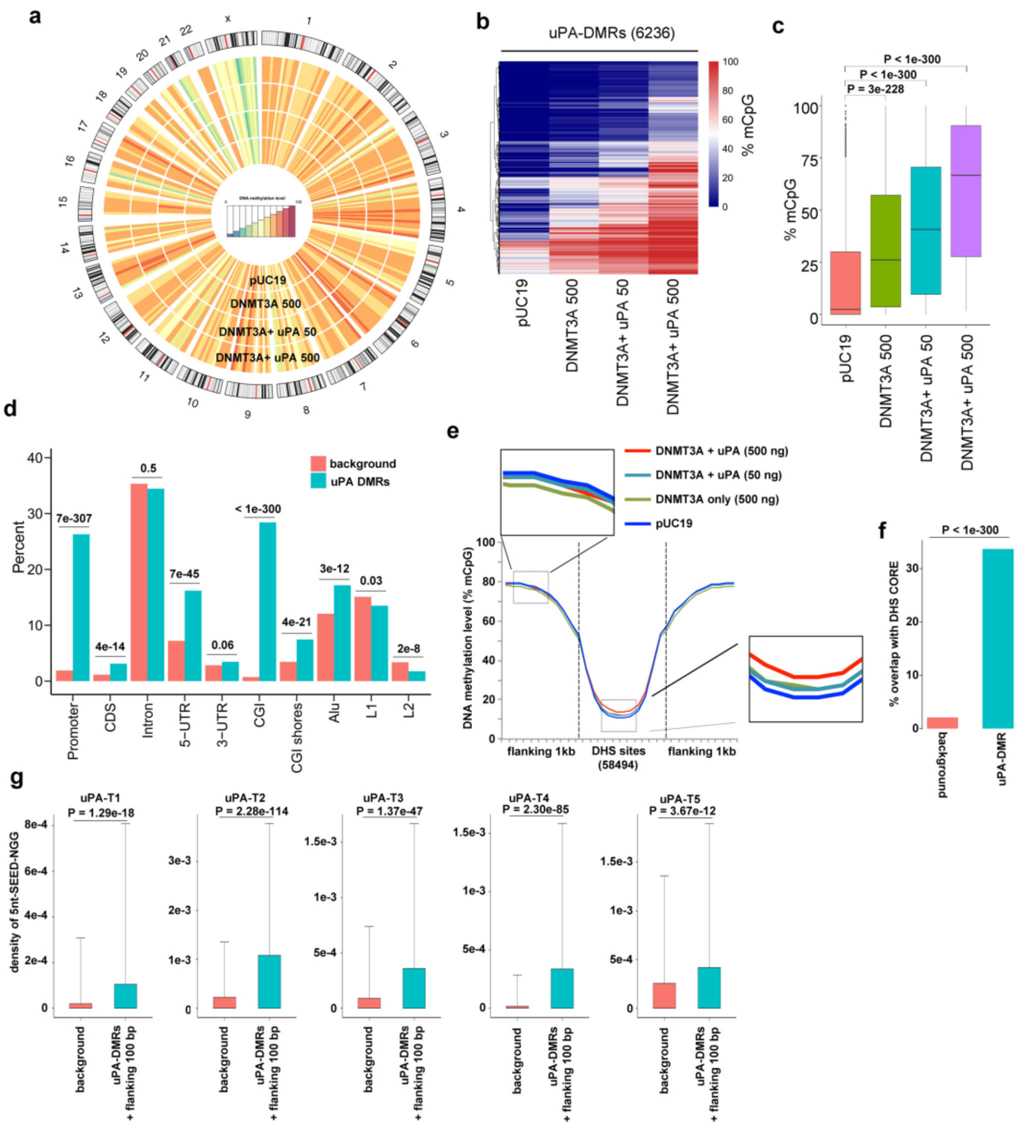

Fig. 8 Effect of overexpressing dCas9-BFP-DNMT3A and uPA gRNAs on global DNA methylation

(**a**) Circular plot of average methylation of 289 10Mb-genomic windows in cells transfected with either pUC19, 500 ng of dCas9-BFP-DNMT3A (DNMT3A 500), 50 ng each of dCas9-BFP-DNMT3A and uPA gRNAs (DNMT3A + uPA 50), or 500 ng each of dCas9-BFP-DNMT3A and uPA gRNAs (DNMT3A + uPA 500). (**b**) Heapmap clustering of the hypermethylated DMRs resulted from expressing high amount of 500 ng each of dCas9-BFP-DNMT3A and uPA gRNAs (DNMT3A + uPA 500) (denoted uPA-DMRs, n = 6236). (**c**) Box plot of uPA-DMRs methylation

levels. P values (t-test) are given above the plot. (**d**) Bar chart illustrating the percentage of the identified uPA-DMRs that fall into the different types of genomic regions indicated. Background represents the percentage of a random sample of the same number of similar sized genomic windows that fall into the categories indicated. Values above bars are P values between background and uPA-DMRs, Fisher's exact test. (**e**) Metaplot of average CpG methylation levels in 58,494 DNase I hypersensitive sites (DHS) and 1 kb upstream and downstream flanking regions. (**f**) Bar chart of % uPA-DMRs falling into DHS core regions. (**g**) Density of 5nt-SEED-NGG for uPA gRNAs (T1 to T5) in background genomic windows and uPA-DMRs + flanking 100 bp. Values represent median density with one standard deviation. P values (t-test) are given above the bar charts.

Fig. 9

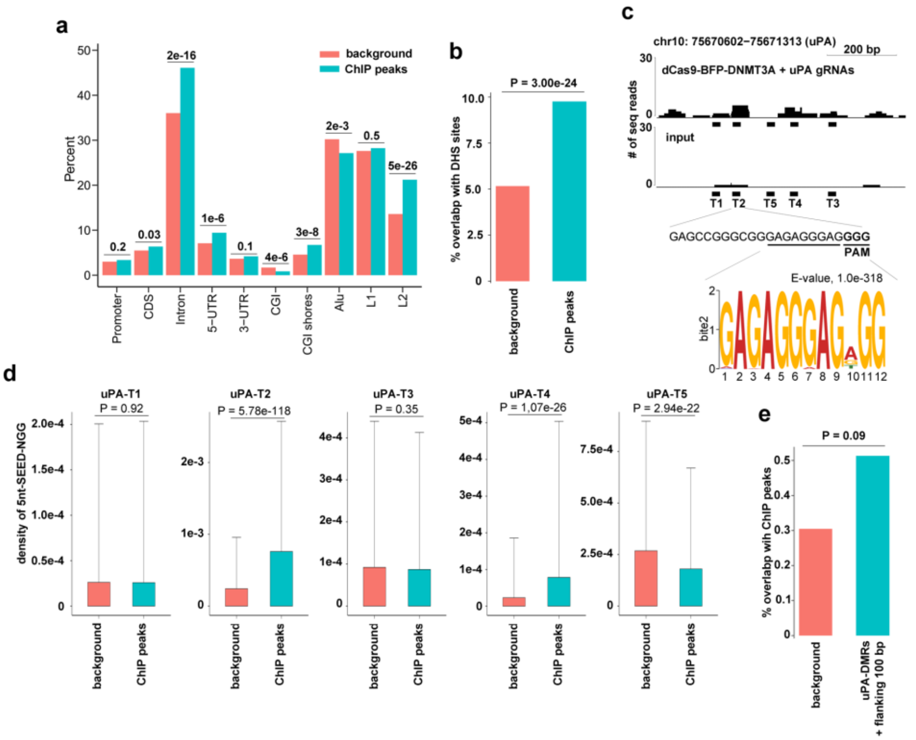

Fig. 9 Correlation between CRISPR off-target binding identified by ChIP-seq and off-target methylation revealed by WGBS

(**a**) Bar chart illustrating the percentage of ChIP peaks from cells expressing dCas9-BFP-DNMT3A and uPA gRNAs or background control regions (random sampling of the same number of similar sized genomic windows as the ChIP peaks) falling into the different types of genomic regions indicated. P-values between background and ChIP peaks indicated above bars, Fisher's exact test. (**b**) Bar chart of % ChIP-peaks falling into DHS core regions. (**c**) Representative plot of

ChIP-seq reads in the uPA promoter, uPA gRNA T2 sequences, and the top motif identified by MEME-ChIP. (**d**) Density of 5nt-SEED-NGG for uPA gRNAs (T1 to T5) ChIP peaks. Background is a random sample of the same number of similar sized genomic windows as ChIP peaks. Values represent median density with one standard deviation. P values are given for the indicated comparisons, t-test. (**e**) Bar plot of % uPA-DMRs overlapping with ChIP peaks. Background is a random sample of the same number of similar sized genomic windows as uPA-DMRs.

Supplementary Figure Legends

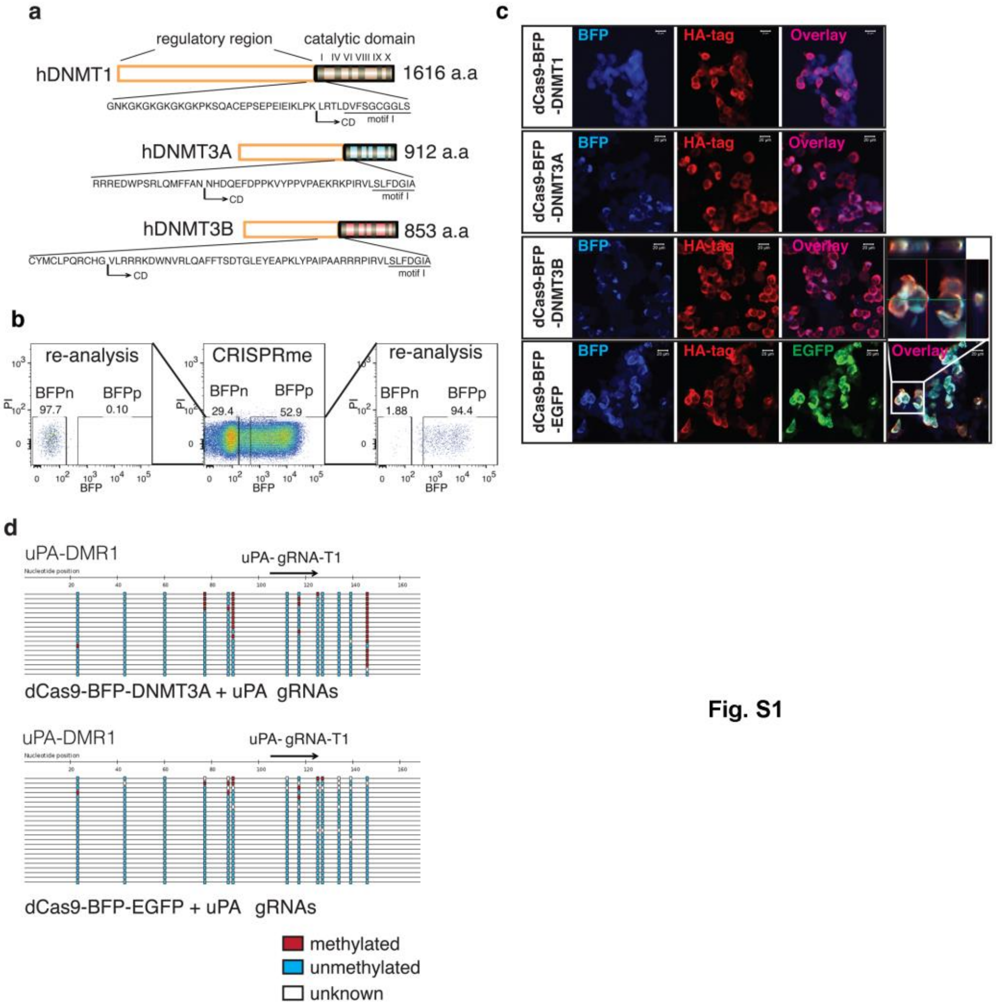

Fig. S1

Supplementary Fig. 1 Validation of CRISPRme expression and *uPA* promoter methylation
(**a**) Schematic overview of the human DNA methyltransferases (DNMT1, DNMT3A and DNMT3B) with the N-terminal regulatory region, a C-terminal catalytic domain (CD), and the cytosine C5-DNA methyltransferase motifs highlighted. The first amino acid (a.a) residue of the C-terminal catalytic domain, which was fused to the dCas9, is indicated by an arrow. (**b**) Representative FACS sorting and Re-analysis of HEK293T cells 48 hours after CRISPRme transfection. Gating for BFP positive (BFPp) and negative (BFPn) cells are indicated. (**c**) Laser scanning microscopy of CRISPRme expression in HEK293T cells, 48 hours after transfection. The BFP signal from the dCas9-BFP-DNMT1 transfected cells was enhanced since the BFP signal from the dCas9-BFP-DNMT1 fusion was initially weaker compared to that from the other three fusion proteins. Scale

bar: 20 µm. (**d**) Validation of RNA-guided *uPA* methylation (uPA-MR1) by dCas9-BFP-DNMT3A using bisulfite Sanger sequencing.

Supplementary Fig. 2

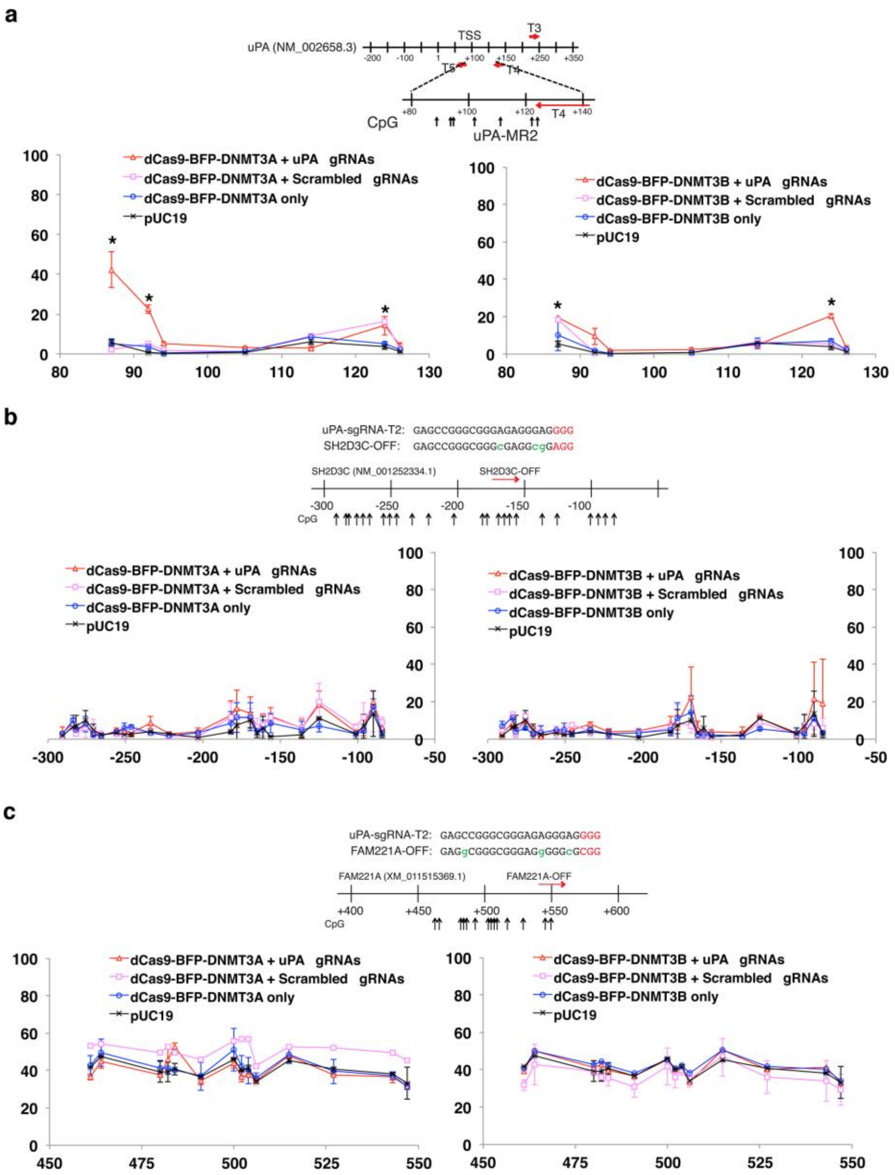

Supplementary Fig. 2 Validation of de novo methylation of uPA by CRISPRme and effect of CRISPRme on two potential off-target sites (*SH2D3C* and *FAM221A*).

(**a**) Line plots of uPA-MR2 methylation in cells transfected with pUC19 (control), dCas9-BFP-DNMT3A or dCas9-BFP-DNMT3A only, and dCas9-BFP-DNMT3A or dCas9-BFP-DNMT3A together with either *uPA* gRNAs or scrambled gRNAs. (**b-c**) Schematic illustration of the *SH2D3C* (**b**) and *FAM221A* (**c**) off-target loci, with off-target sites indicated by red arrows. Sequences of *uPA* gRNA (T2), *SH2D3C*, and *FAM221A* off-target sites are given above, with the PAM (red letters) and mismatches (green letters) indicated. CpGs analyzed are indicated by black arrows; numbers indicate distances (in bp) from the transcription start site (TSS) of the gene (SH2D3C, NM_001252334.1) or (FAM221A, XM_011515369.1). Y-axis represents % mCpG level for each CpG site and X-axis represents distance (in bp) from TSS. The CpG methylation level from the control samples (pUC19 transfection) was re-plotted as a reference. Each data point in the graph represents the mean percentage of CpGs methylated ± SD (n = 2, independent transfections).

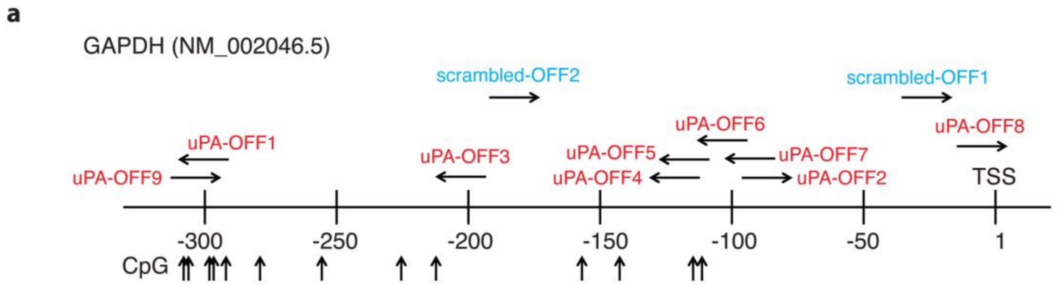

sgRNA	potential OFF sites	strand	guiding site	Sequence (5'-)	Gene	mismatches
uPA_T1	uPA-OFF1	-	chr12:6643261-6643283	GCACCGCTCGCGGCTGCACGCGG	GAPDH	9
uPA_T1	uPA-OFF2	+	chr12:6643477-6643499	GCGTGCCCAGTTGAACCAGGCGG	GAPDH	9
uPA_T2	uPA-OFF3	-	chr12:6643356-6643378	GACACTAGGGAGTCAAGGACGGG	GAPDH	10
uPA_T2	uPA-OFF4	-	chr12:6643442-6643464	GGCCCCGACGTAGGGGGGAAGGG	GAPDH	9
uPA_T2	uPA-OFF5	-	chr12:6643443-6643465	GGGCCCCGACGTAGGGGGGAAGG	GAPDH	10
uPA_T3	uPA-OFF6	-	chr12:6643451-6643473	GAGCGTGTGGGCCCCGACGTAGG	GAPDH	10
uPA_T3	uPA-OFF7	-	chr12:6643471-6643493	GGTTCAACTGGGCACGCACCGAG	GAPDH	10
uPA_T4	uPA-OFF8	+	chr12:6643556-6643578	GCGCACGTAGCTCAGGCCTCAAG	GAPDH	10
uPA_T5	uPA-OFF9	+	chr12:6643258-6643280	AGGCCGCGTGCAGCCGCGAGCGG	GAPDH	10
Scrambed_T1	Scrambled-OFF1	+	chr12:6643534-6643556	CCGGCTACTAGCGGTTTTACGGG	GAPDH	10
Scrambed_T2	Scrambled-OFF2	+	chr12:6643381-6643403	GCTGCCCACAGTCCAGTCCTGGG	GAPDH	10

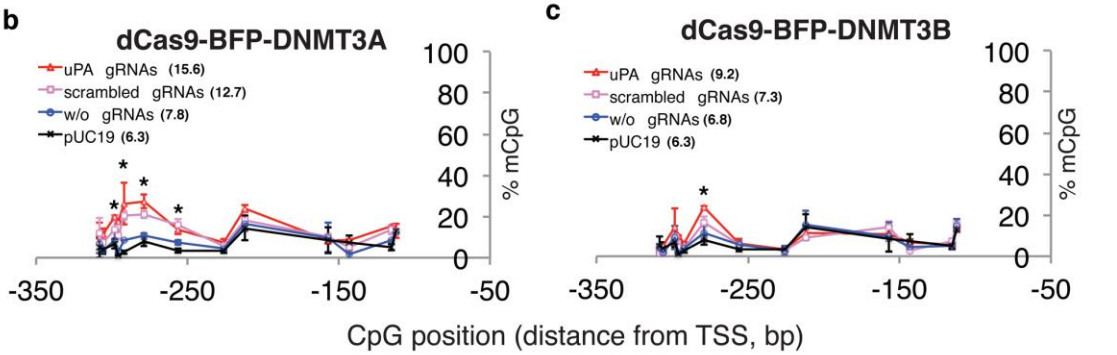

Fig S3

Supplementary Fig. 3 Effects of expressing CRISPRme and *uPA* gRNAs on GAPDH promoter methylation

(**a**) Schematic illustration of the *GAPDH* promoter. Potential off target sites and CpGs analyzed by bisulfite pyrosequencing are indicated. Sequences of the *uPA* or scrambled gRNAs with maximum 10 mismatches are listed. (**b-c**) Line plots *GAPDH* promoter methylation in FACS-sorted BFPp HEK293T cells 48 hours after transfection with dCas9-BFP-DNMT3A (**b**) or dCas9-BFP-DNMT3B (**c**), alone or together with either scrambled or uPA gRNAs. The methylation profiles from the pUC19-transfected samples were re-plotted as reference. Each data point in the

graph represents the mean ± SD (n = 2, independent transfections). Mean methylation levels for all CpGs analyzed are presented next to line legends. Asterisks (*) represent P value < 0.05 compared to pUC19, ANOVA.

Fig S4

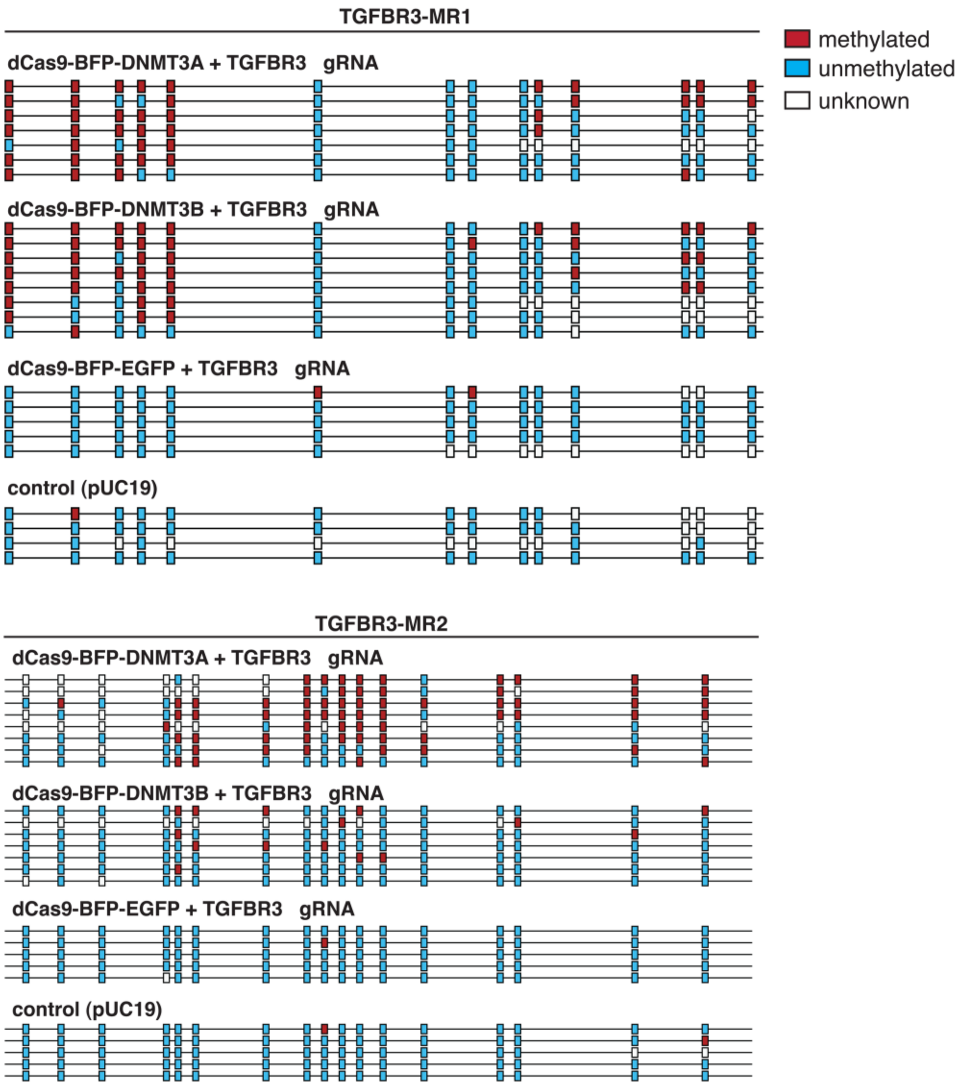

Supplementary Fig. 4 Validation of CRISPRme-mediated *TGFBR3* methylation in HEK293T cells by bisulfite Sanger sequencing

TGFBR3 methylation by CRISPRme was validated by bisulfite Sanger sequencing. CpG methylation status is indicated according to the absolute nucleotide position and color-coded as red, methylated; blue, unmethylated; or white, unknown methylation state based on the sequencing signal.

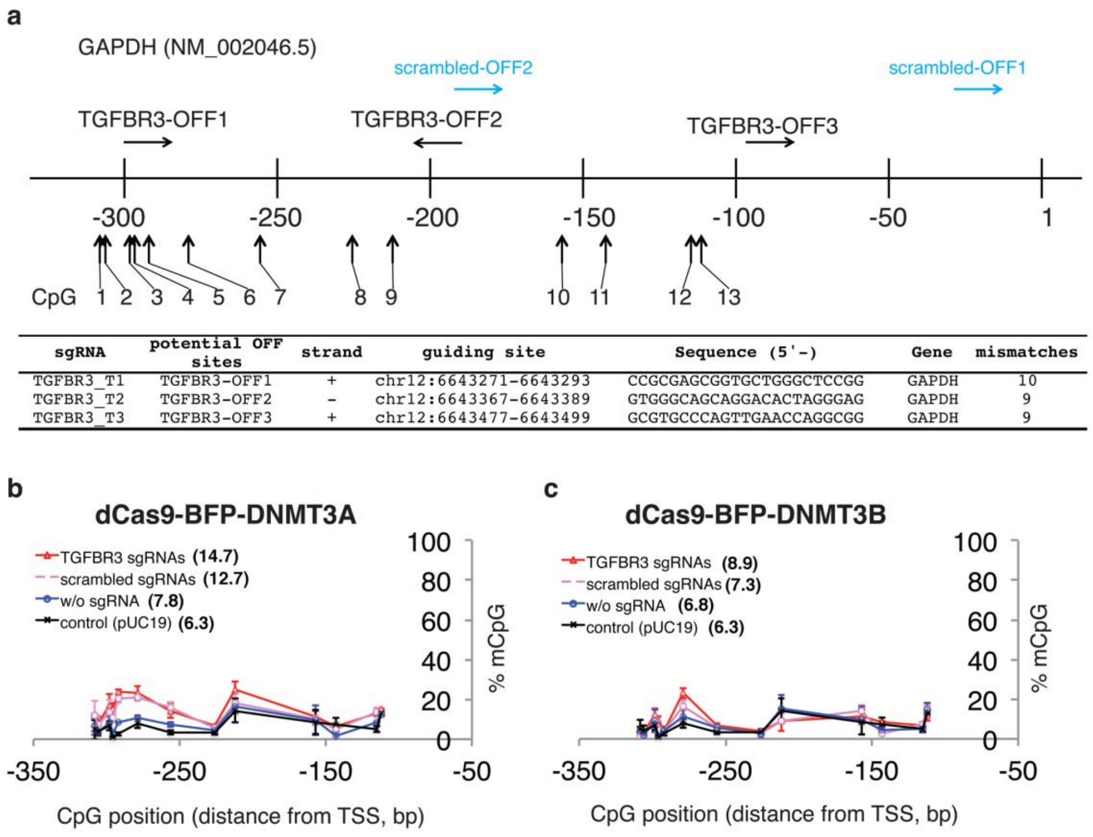

sgRNA	potential OFF sites	strand	guiding site	Sequence (5'-)	Gene	mismatches
TGFBR3_T1	TGFBR3-OFF1	+	chr12:6643271-6643293	CCGCGAGCGGTGCTGGGCTCCGG	GAPDH	10
TGFBR3_T2	TGFBR3-OFF2	-	chr12:6643367-6643389	GTGGGCAGCAGGACACTAGGGAG	GAPDH	9
TGFBR3_T3	TGFBR3-OFF3	+	chr12:6643477-6643499	GCGTGCCCAGTTGAACCAGGCGG	GAPDH	9

Fig S5

Supplementary Fig. 5 Effects of expressing CRISPRme and *TGFBR3* gRNAs on *GAPDH* promoter methylation

(**a**) Schematic illustration of the *GAPDH* promoter, potential off-target sites of *TGFBR3* gRNAs and scrambled gRNAs, and the CpGs analyzed by bisulfite pyrosequencing. Percentage of CpGs methylated within the *GAPDH* promoter was analyzed in the CRISPRme-expressing HEK293T cells. The BFP positive cells were FACS-sorted 48 hours after co-transfection with the *TGFBR3* or scrambled gRNAs and either dCas9-BFP-DNMT3A (**b**) or dCas9-BFP-DNMT3B (**c**). Note that data points for groups with scrambled gRNAs, without (w/o) gRNAs and with pUC19 are re-plotted from **Supplementary Fig. 3** for reference. Each data point in the graph represents the mean ± SD (n = 2, independent transfections). Mean methylation levels for all CpGs analyzed are

presented next to line legends. (**c**) Schematic plot of PAM (NGG) and CpG sites in the *GAPDH* promoter.

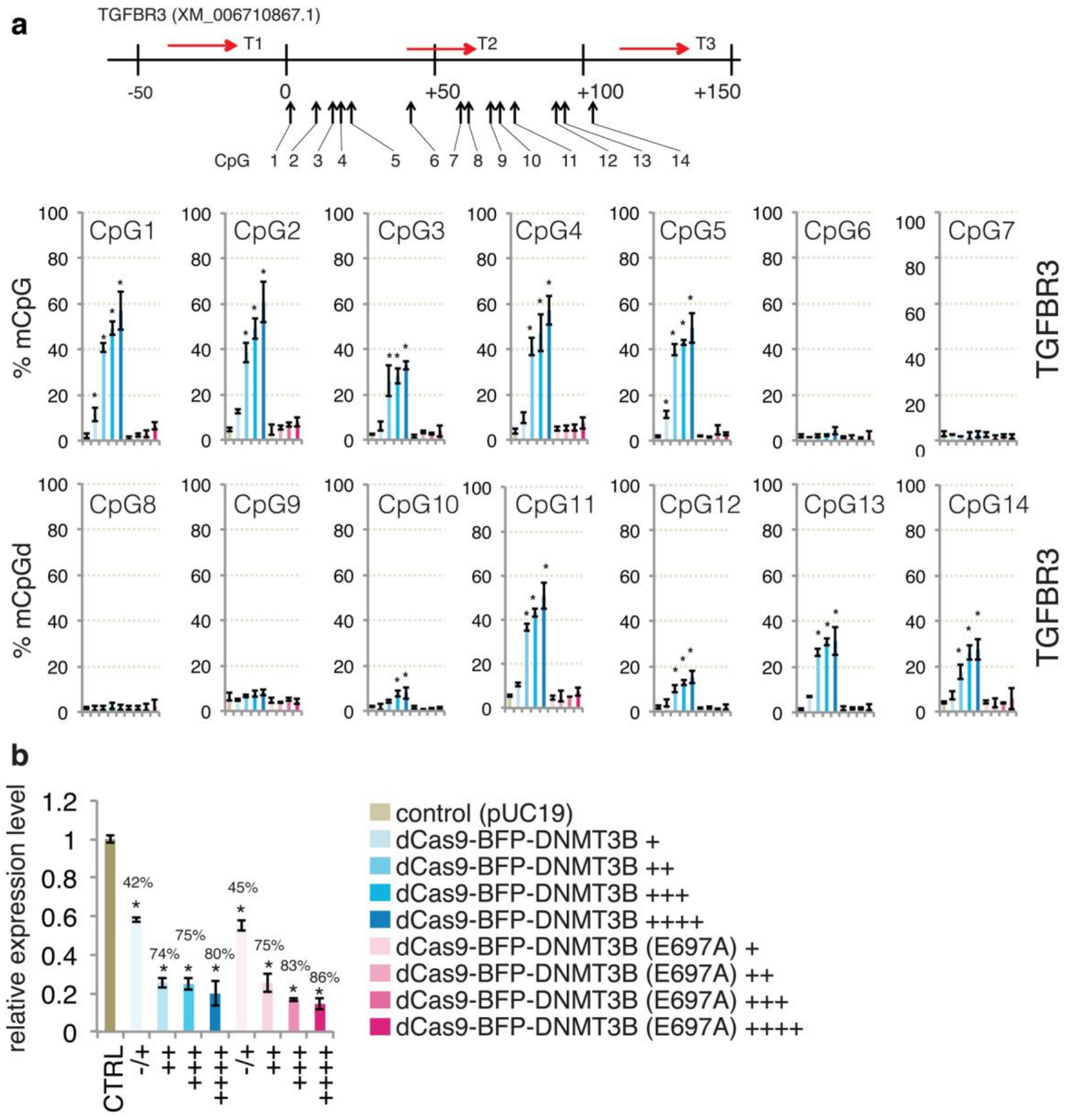

Fig S6

Supplementary Fig. 6 Evaluation of *De novo TGFBR3* methylation and expression inhibition by *TGFBR3 gRNAs together with either* dCas9-BFP-DNMT3B or dCas9-BFP-DNMT3B (E697A).

(**a**) Bar charts of % mCpG level for individual CpG sites of the *TGFBR3* targeted regions in CRISPRme-expressing cells. Cells were enriched by FACS 48 hours after transfection and sorted according to the BPF signal: +, ++, +++, ++++. The schematic illustrations above the bar charts

show gRNA binding sites and CpG sites analyzed. (**b**) Bar chart of relative *TGFBR3* expression (% of pUC19). Both %mCpG and relative gene expression values represent mean ± SD (n = 3, independent transfections). Asterisk (*) indicates statistical significance ($p < 0.05$, ANOVA) compared to the pUC19 control group after Bonferroni correction. Percentage values represent % decrease of *TGFBR3* expression compared to pUC19.

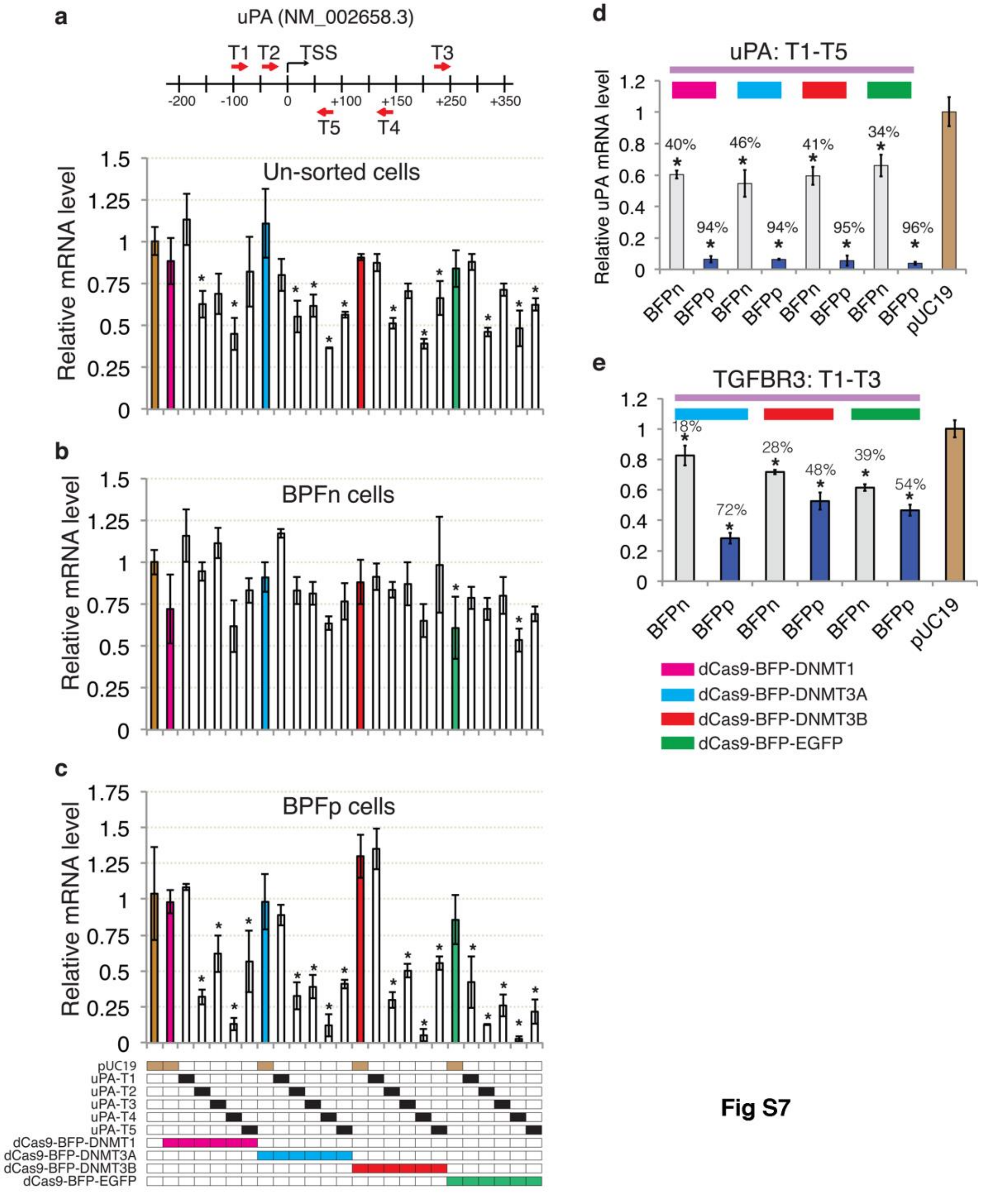

Fig S7

Supplementary Fig. 7 Inhibition of *uPA* and *TGFBR3* expression in HEK293T cells by CRISPRme 1.0 and gRNAs

Relative *uPA* mRNA level in unsorted (**a**), FACS-sorted BFP negative (BFPn, **b**), or BFP positive (BFPp, **c**) HEK293T cells 48 hours after co-transfection of a single *uPA* gRNA and a dCas9 fusion protein. Note that BFPn groups contain cells with weak BFP signals. The transfection

scheme for for the experiment is indicated in the bottom panel of the figure; filled boxes indicate the inclusion of the corresponding plasmid in the transfection. (**d-e**) Relative mRNA levels in cells expressing the corresponding dCas9 fusion protein and either five *uPA* gRNAs (**d**) or three *TGFBR3* gRNAs (**e**). Relative *uPA* and *TGFBR3* mRNA levels were calculated as fold changes compared to the control (pUC19 transfection). Each data point is presented as mean ± SD (n = 3, independent transfections). Asterisk (*) indicates a p-value < 0.05 compared to the control (pUC19).

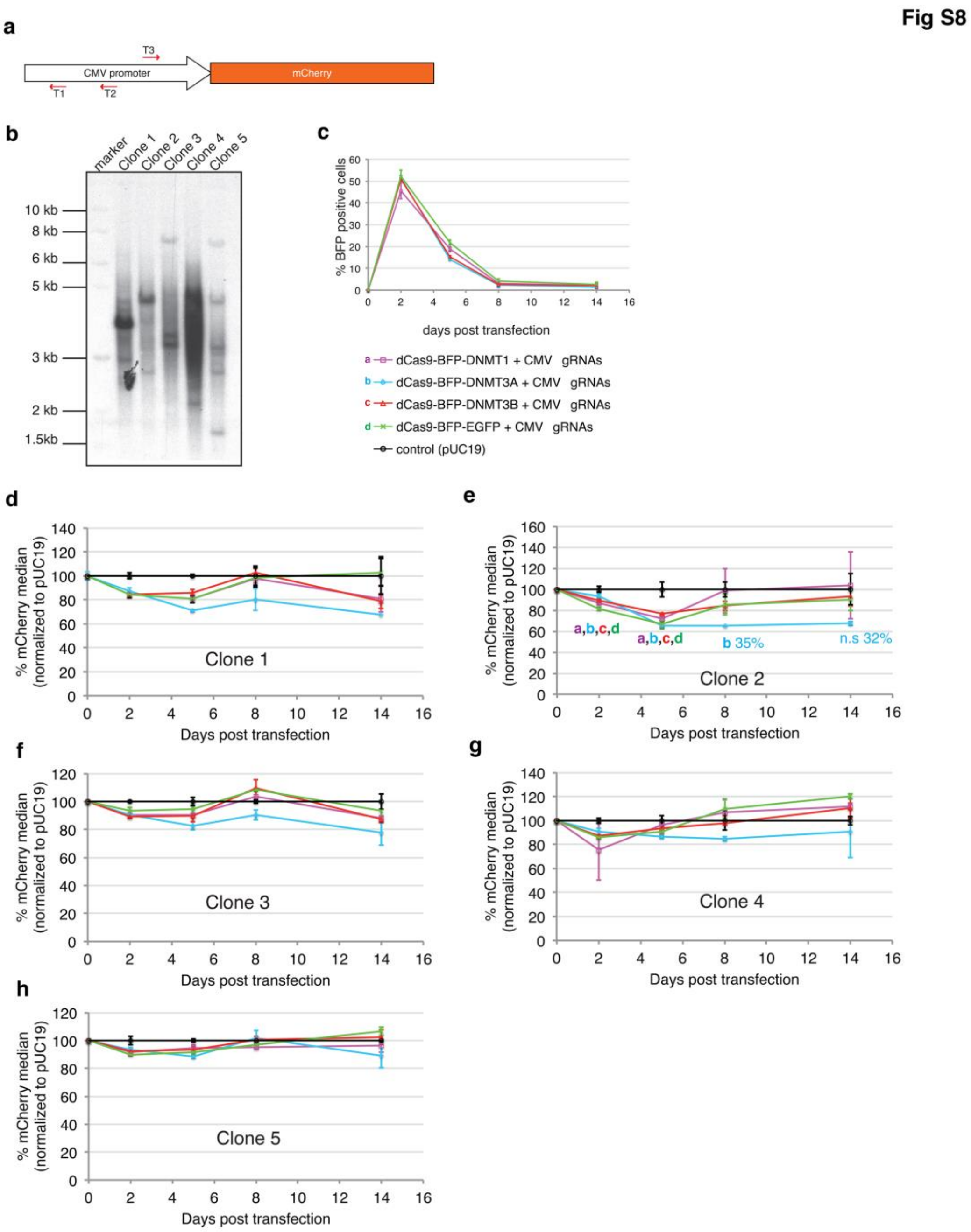

Supplementary Fig. 8 Effects of CRISPRme on mCherry expression in fluorescence reporter cell lines

(**a**) Schematic illustration of the mCherry fluorescence transgene expression cassette. The target sites of the CRISPRme gRNAs within the CMV promoter are indicated by red arrows (5’-3’, targeting sense or antisense strands). (**b**) Southern blot analysis of five cell clones with the transgene cassette randomly and stably integrated into the genome. (**c**) Flow cytometry-based analysis of the percentage of BFP positive cells in the fluorescence reporter cells at 2, 5, 8 and 14

days after transient transfection with CMV gRNAs (T1-T3) and dCas9-BFP-DNMT1, dCas9-BFP-DNMT3A, dCas9-BFP-DNMT3B, or dCas9-BFP-EGFP. (**d-h**) % mCherry fluorescence median intensity in these five clones at day 2, 5, 8, and 14 days following transient transfection with CMV gRNAs (T1-T3) and dCas9-BFP-DNMT1, dCas9-BFP-DNMT3A, dCas9-BFP-DNMT3B, or dCas9-BFP-EGFP. Control cells were transfected with pUC19. Percent inhibition of mCherry expression was calculated by normalizing the median mCherry fluorescence intensity to that from the pUC19 transfected cells at each time point. Figures are plotted using the mean % mCherry median ± SD (n = 3, independent transfections). ANOVA with Bonferroni comparison was performed for cell clone 2. “a”, “b”, “c”, and “d,” indicates a p-value < 0.05 compared to the pUC19 control for the corresponding CRISPRme transfection group.

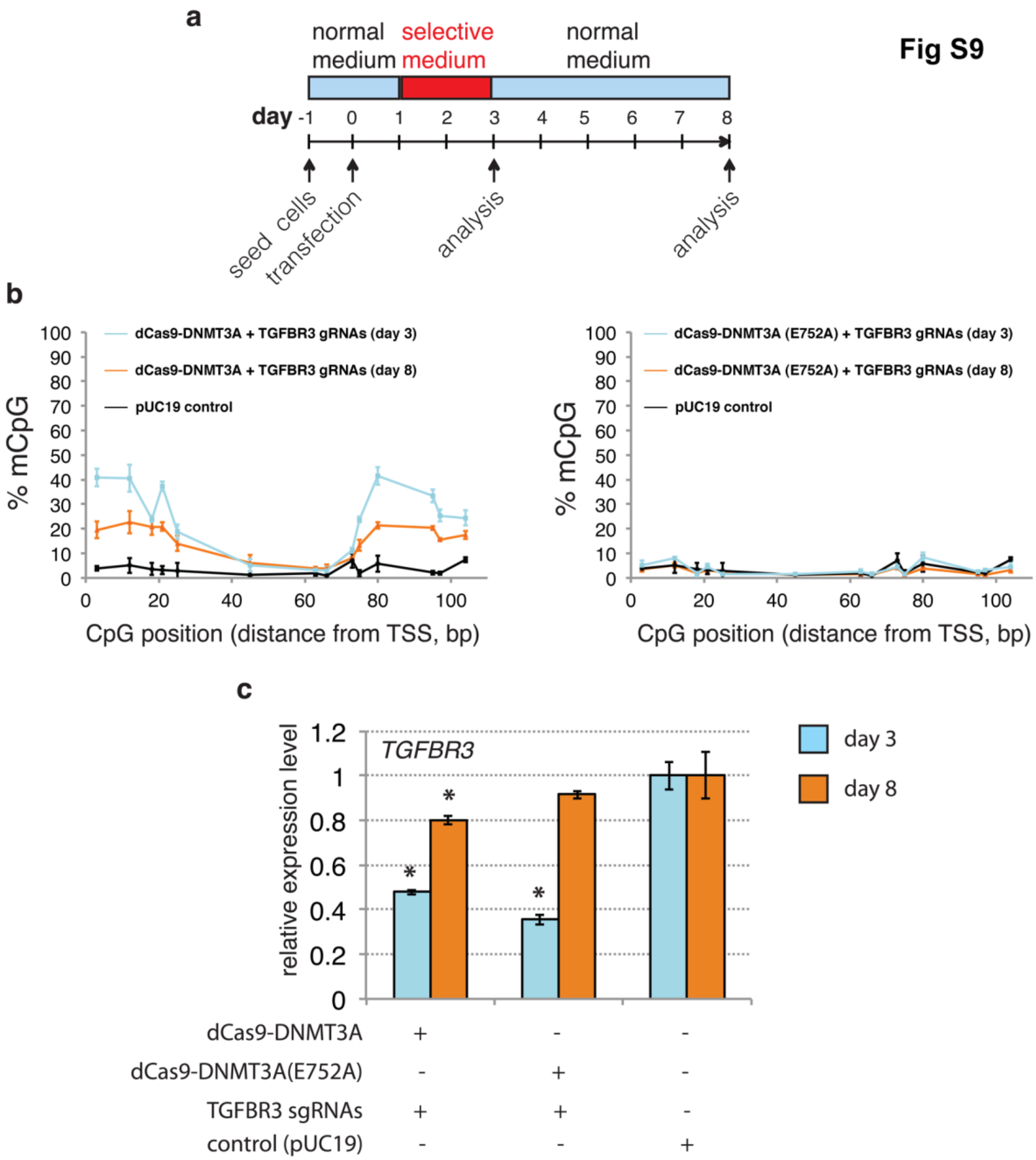

Supplementary Fig. 9 Determination of long-term methylation and inhibition of *TGFBR3* by CRISPRme2.0

(**a**) Schematic representation of the time-course of the experiment. HEK293T cells were transfected with the *TGFBR3* gRNAs and either lenti-dCas9-DNMT3A or lenti-dCas9-DNMT3A (E752A) at day 0, cultured in selective medium from day 1 to day 3, and cultured in non-selective medium from day 3. Control cells were transfected with an identical amount of the pUC19 plasmid. Transfected cells were harvested for methylation analysis of TGFBR3-MR1 using bisulfite pyrosequencing (**b**) and for *TGFBR3* expression analysis (**c**) at day 3 (blue lines or bars)

and day 8 (orange lines or bars). Figures are plotted using mean ± SD (n = 3, independent transfections). Asterisk (*) indicates a p-value < 0.05 compared to the control.

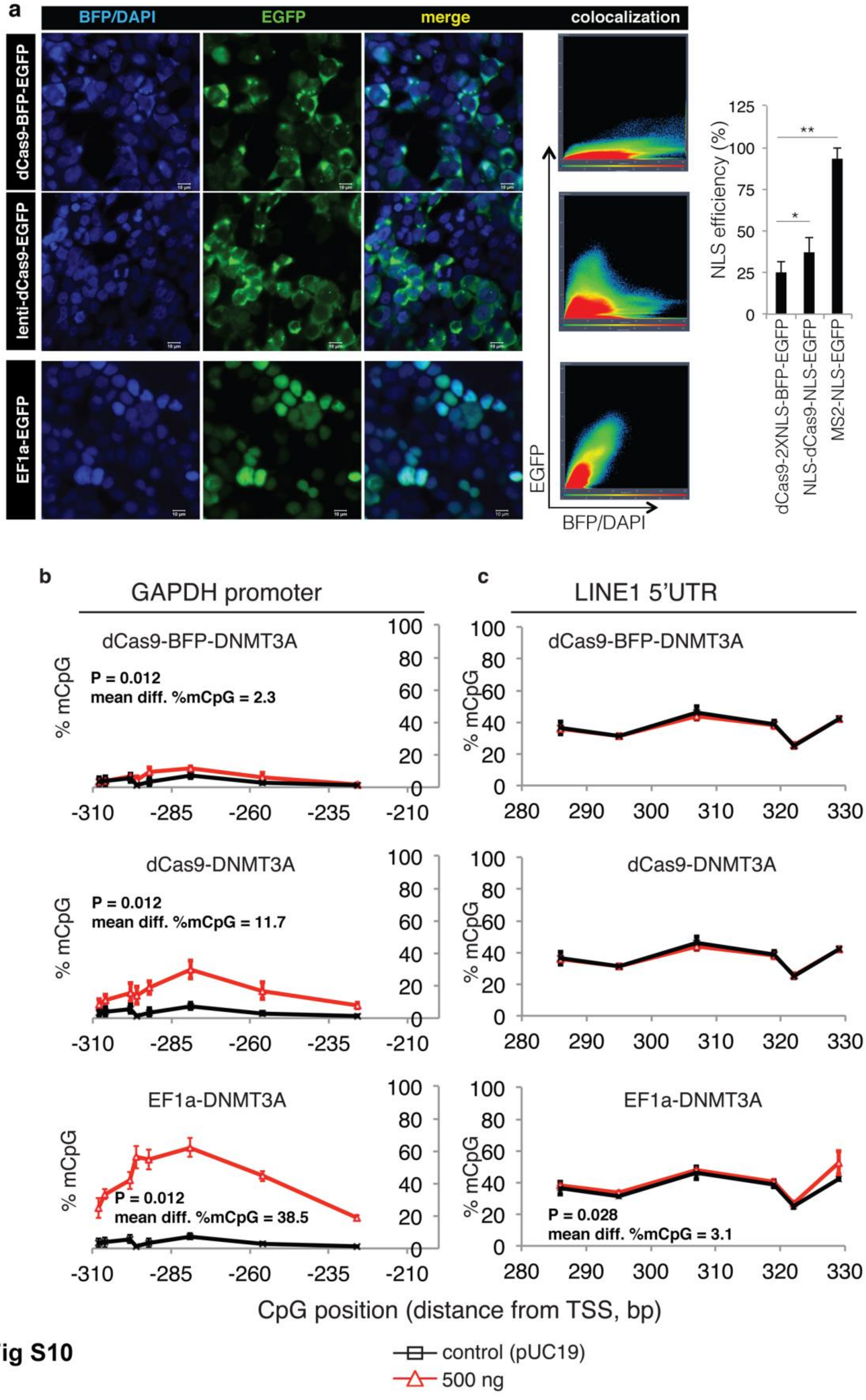
a
BFP/DAPI
EGFP
merge
colocalization
dCas9-BFP-EGFP
lenti-dCas9-EGFP
EF1a-EGFP
EGFP
BFP/DAPI
NLS efficiency (%)
125
100
75
50
25
0
*
**
dCas9-2XNLS-BFP-EGFP
NLS-dCas9-NLS-EGFP
MS2-NLS-EGFP
b
GAPDH promoter
c
LINE1 5'UTR
dCas9-BFP-DNMT3A
P = 0.012
mean diff. %mCpG = 2.3
dCas9-DNMT3A
P = 0.012
mean diff. %mCpG = 11.7
EF1a-DNMT3A
P = 0.012
mean diff. %mCpG = 38.5
P = 0.028
mean diff. %mCpG = 3.1
% mCpG
-310 -285 -260 -235 -210
280 290 300 310 320 330
CpG position (distance from TSS, bp)
control (pUC19)
500 ng

Fig S10

Supplementary Fig. 10 Comparison of dCas9 methyltransferase expression, nuclear entry efficiency, and the effects on specificity

(**a**) Laser scanning fluorescent microscopy of HEK293T cells expressing three EGFP fusion proteins. NLS, nuclear localization signal; BFP, blue fluorescent protein; MS2, the MS2 bacteriophage coat protein. Co-localization of EGFP and BFP/DAPI is observed. Nuclear localization was estimated using Image J. *, p-value < 0.05; **, p-value < 0.01 by ANOVA. (**b-c**) Percentage methylation of *GAPDH* (**b**) and *LINE1* 5'UTR (**c**). Each data point is presented as mean ± SD (n = 3, independent transfections). % mCpG levels were analyzed in cells 48 hours after transfection with dCas9-BFP-DNMT3A, lenti-dCas9-DNMT3A, or EF1a-DNMT3A plasmids (500 ng). Cells transfected with an identical amount of the pUC19 plasmid were used as control and re-plotted for the different treatments as reference. Average difference in % mCpG levels between experimental groups and controls are shown together with Wilcoxon matched-pairs signed-rank test P values.

a

Sample ID	Description	Clean Data Size(bp)	Clean Reads Number	Clean Rate(%)	Mapped Reads	Mapping Rate (%)	Uniquely Mapped Reads	Uniquely Mapping Rate (%)	Bisulfite Conversion Rate (%)
g1	dCas9-BFP-DNMT3A (500 ng) + uPA gRNAs (500 ng)	100,601,409,600	670,676,064	95.06	558316453	83.25	558316453	83.25	99.63
g2	dCas9-BFP-DNMT3B (500 ng) + uPA gRNAs (500 ng)	103,778,694,600	691,857,964	95.64	575422550	83.17	575422550	83.17	99.52
g3	dCas9-BFP-DNMT3A (500 ng) + TGFBR3 gRNAs (500 ng)	117,505,796,100	783,371,974	94.06	651431832	83.16	651431832	83.16	99.65
g4	dCas9-BFP-DNMT3B (500 ng) + TGFBR3 gRNAs (500 ng)	109,001,895,600	726,679,304	90.65	604104613	83.13	604104613	83.13	99.63
g5	dCas9-BFP-DNMT3A (500 ng) only	105,265,839,000	701,772,260	90.36	582484794	83	582484794	83	99.62
g6	dCas9-BFP-DNMT3B (500 ng) only	118,394,522,400	789,296,816	95.6	660303371	83.66	660303371	83.66	99.6
g7	dCas9-BFP-DNMT3A (50 ng) + uPA gRNAs (50 ng)	117,366,362,100	782,442,414	87.18	645181340	82.46	645181340	82.46	99.6
g8	dCas9-BFP-DNMT3A (50 ng) + TGFBR3 gRNAs (50 ng)	101,372,416,500	675,816,110	85.25	561172295	83.04	561172295	83.04	99.49
g9	pUC19 control	80,429,413,800	536,196,092	90.08	444407297	82.88	444407297	82.88	99.46

b

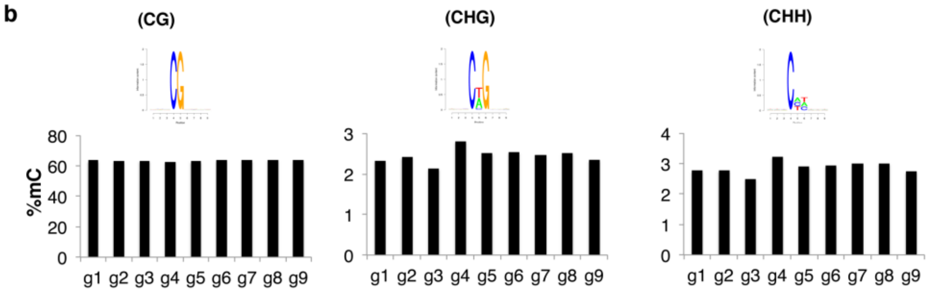

Fig S11

Supplementary Fig. 11 WGBS analysis of cells expressing CRISPRme1.0 and gRNAs

(**a**) Summary of WGBS including clean data, clean reads, clean rate, mapped reads, uniquely mapped reads and rate, and bisulfite conversion rate, for each experimental group and control (pUC19). (**b**) Average percentage of methylated cytosine (% mC) for whole-genome CpG sites, CHG sites, and CHH sites. "H" represents A, C, and T.

Fig. S12

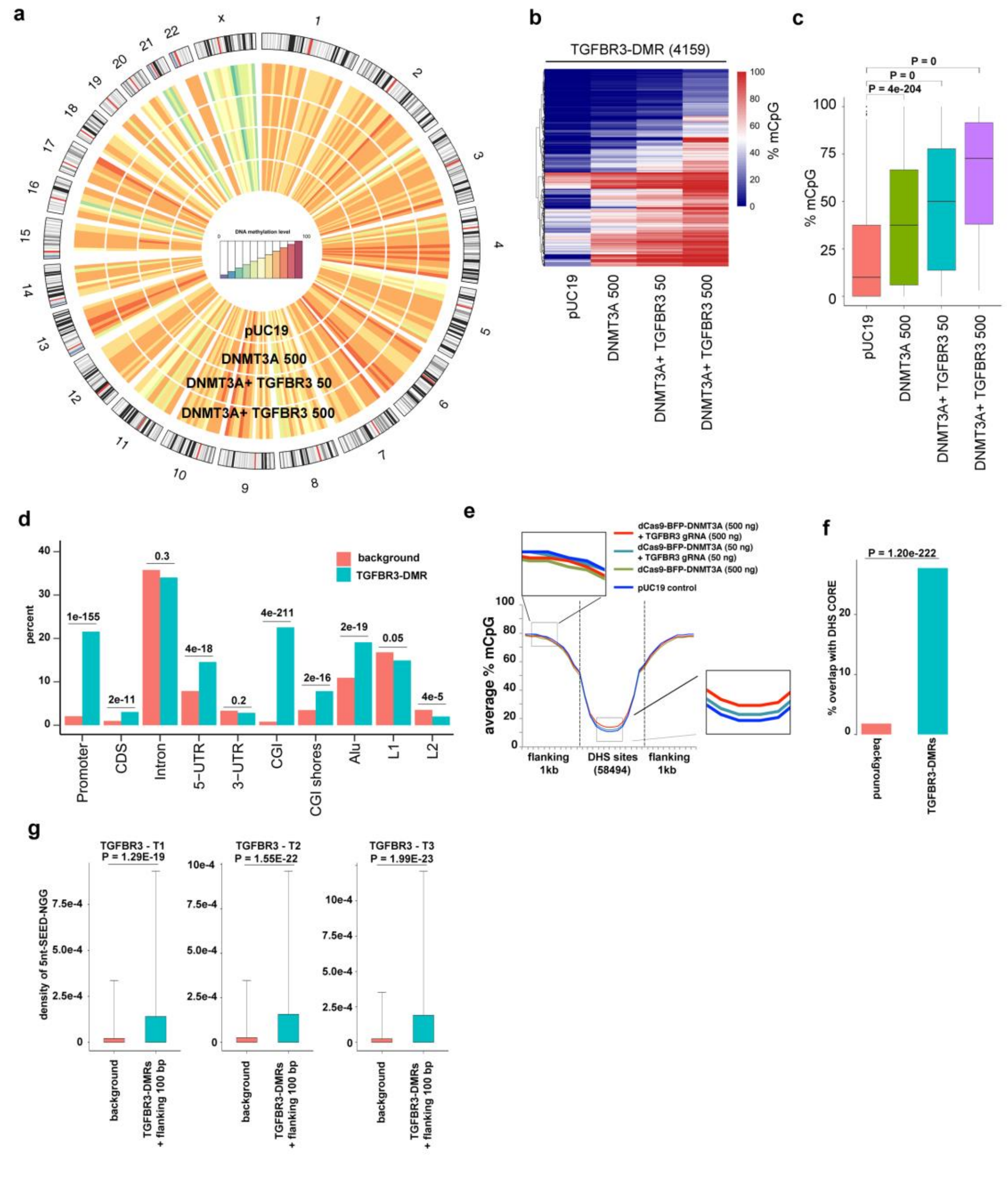

Supplementary Fig. 12 Effect of overexpressing dCas9-BFP-DNMT3A and *TGFBR3* gRNAs on global DNA methylation

(**a**) Circular plot of average methylation of 289 10Mb-genomic windows in cells transfected with either pUC19, 500 ng of dCas9-BFP-DNMT3A (DNMT3A 500), 50 ng each of dCas9-BFP-DNMT3A and *TGFBR3* gRNAs (DNMT3A + *TGFBR3* 50), or 500 ng each of dCas9-BFP-DNMT3A and *TGFBR3* gRNAs (DNMT3A + *TGFBR3* 500). (**b**) Heapmap clustering of the

hypermethylated DMRs resulted from expressing high amount of 500 ng each of dCas9-BFP-DNMT3A and *TGFBR3* gRNAs (DNMT3A + *TGFBR3* 500) (denoted TGFBR3-DMRs, n = 4159). (**c**) Box plot of TGFBR3-DMRs methylation levels. P values (t-test) are given above the plot. (**d**) Bar chart illustrating the percentage of the identified TGFBR3-DMRs that fall into the different types of genomic regions indicated. Background represents the percentage of a random sample of the same number of similar sized genomic windows that fall into the categories indicated. Values above bars are P values between background and TGFBR3-DMRs, Fisher's exact test. (**e**) Metaplot of average CpG methylation levels in 58,494 DNase I hypersensitive sites (DHS) and 1 kb upstream and downstream flanking regions. (**f**) Bar chart of % TGFBR3-DMRs falling into DHS core regions. (**g**) Density of 5nt-SEED-NGG for TGFBR3 gRNAs (T1 to T3) in background genomic windows and TGFBR3A-DMRs + flanking 100 bp. Values represent median density with one standard deviation. P values (t-test) are given above the bar charts.

Fig. S13

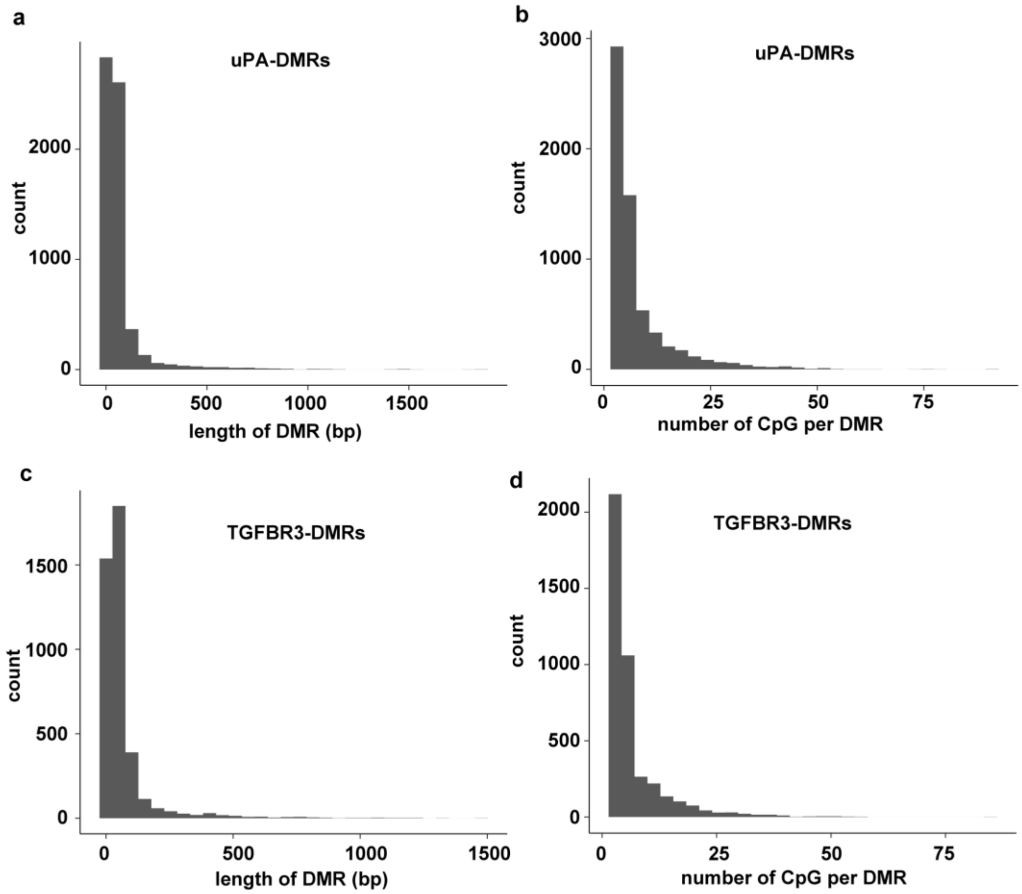

Supplementary Fig. 13 Histogram charts of the distribution of DMR length (bp), and

number of CpGs per DMR. (a-b) Distribution of DMR length and CpG number for the uPA-DMRs. (c-d) Distribution of DMR length and CpG number for the TGFBR3-DMRs

Fig. S14

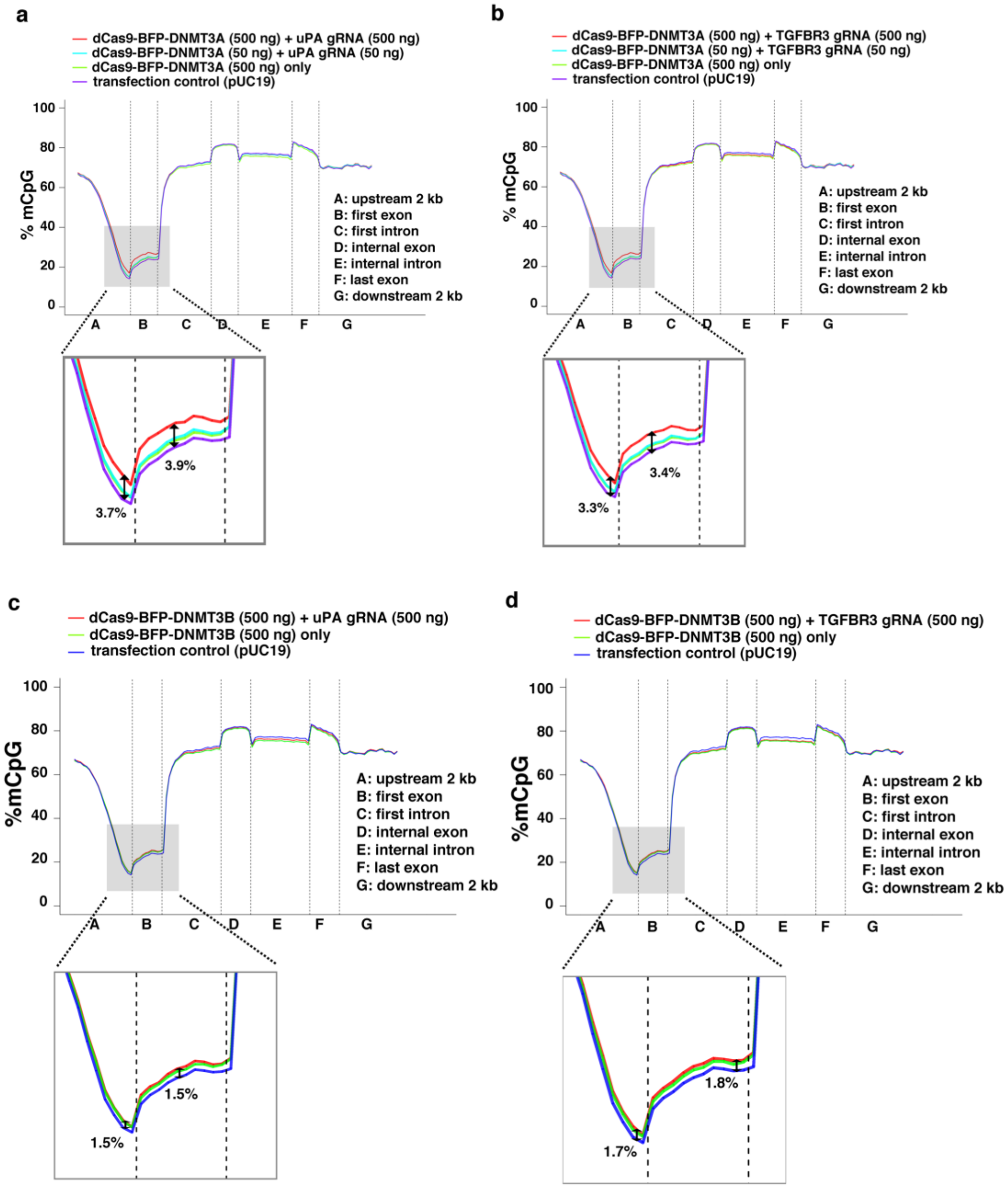

Supplementary Fig. 14 Average methylation levels of seven genomic regions in all annotated genes (hg19). (**a-d**) Each line indicates the genome-wide average methylation levels across seven genomic regions: upstream 2kb of the transcription start site, first exon, first intron, internal exons, internal introns, last exon, and downstream 2kb of the last exon.

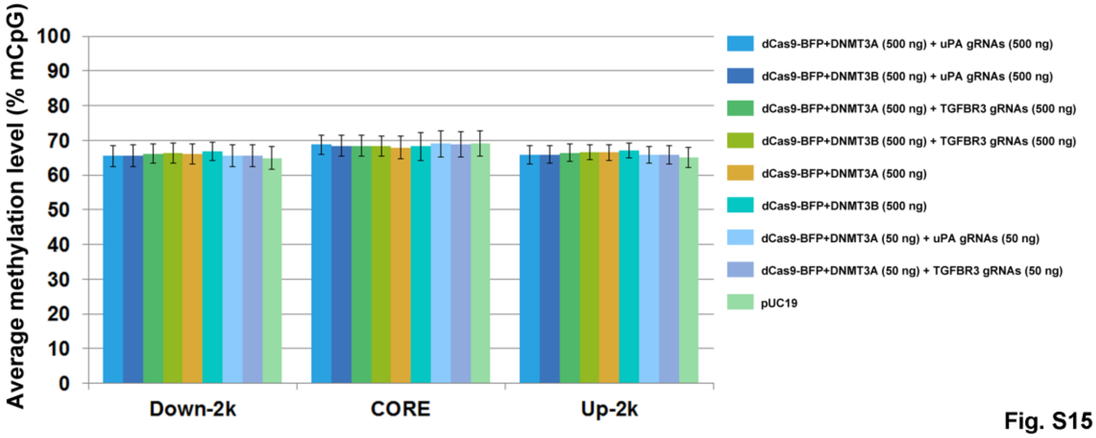

Fig. S15

Supplementary Fig. 15 The average methylation level in ChIP-peaks and flanking regions. Bar chat presents the average methylation level of all dCas9-BFP-DNMT3A and uPA gRNAoff-target binding sites (n = 7754) found by ChIP-seq, as well as the 2kb upstream and downstream region.

Supplementary Table 1 List of uPA-DMRs

Supplementary Table 2 List of TGFBR3-DMRs

Supplementary Table 3 List of ChIP peaks

Supplementary Table 4 List of plasmids deposited to Addgene, qPCR primers, CRISPRme gRNA sequences, bisulfite PCR primers, bisulfite pyrosequencing primers, and DNA regions analyzed for methylation.

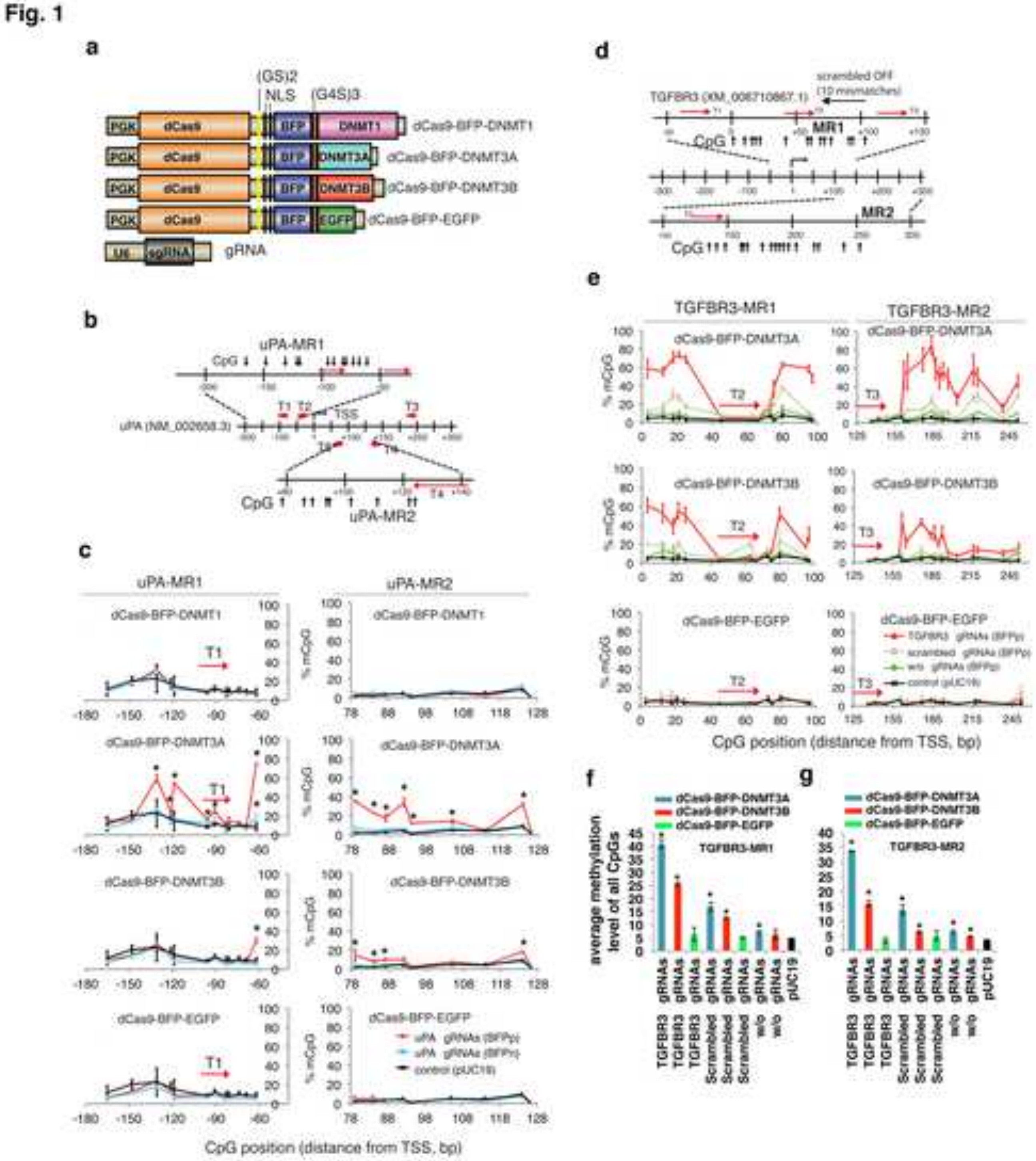

Fig. 1
a
(GS)2
NLS
(G4S)3
PGK
dCas9
BFP
DNMT1
dCas9-BFP-DNMT1
DNMT3A
dCas9-BFP-DNMT3A
DNMT3B
dCas9-BFP-DNMT3B
EGFP
dCas9-BFP-EGFP
U6
sgRNA
gRNA
b
uPA-MR1
CpG
T1
T2
TSS
T3
T4
uPA-MR2
c
uPA-MR1
uPA-MR2
% mCpG
CpG position (distance from TSS, bp)
d
TGFBR3
MR1
MR2
CpG
e
TGFBR3-MR1
TGFBR3-MR2
% mCpG
CpG position (distance from TSS, bp)
f
g
average methylation level of all CpGs
TGFBR3-MR1
TGFBR3-MR2
TGFBR3 gRNAs
Scrambled gRNAs
w/o gRNAs
pUC19

 Click here to download Figure Fig 2 dpi300.tif

Fig 2

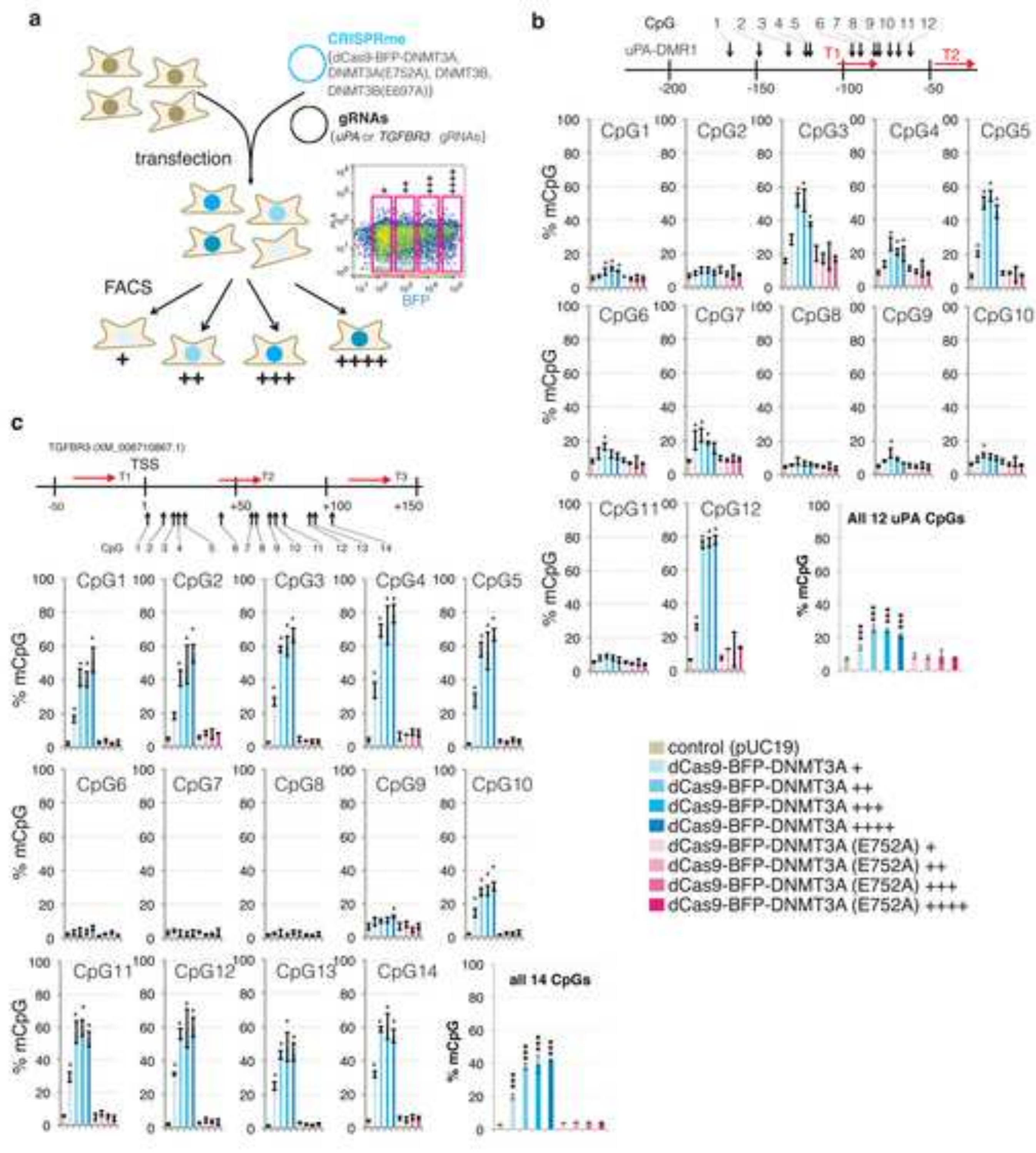

Figure 3

Click here to download Figure Fig 3 dpi300.tif

Fig. 3

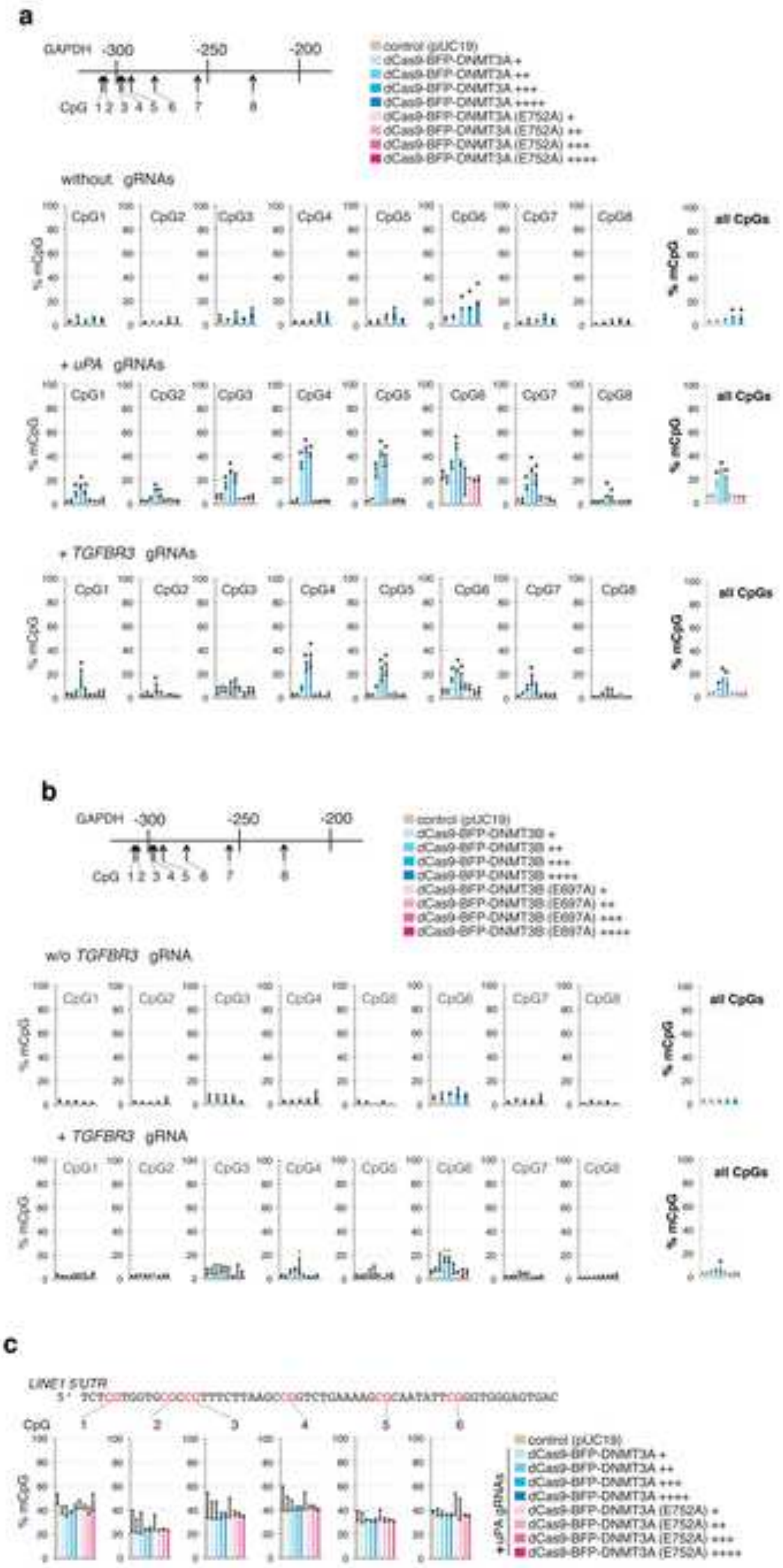

Figure 4 Click here to download Figure Fig 4 dpi300.tif

Fig 4

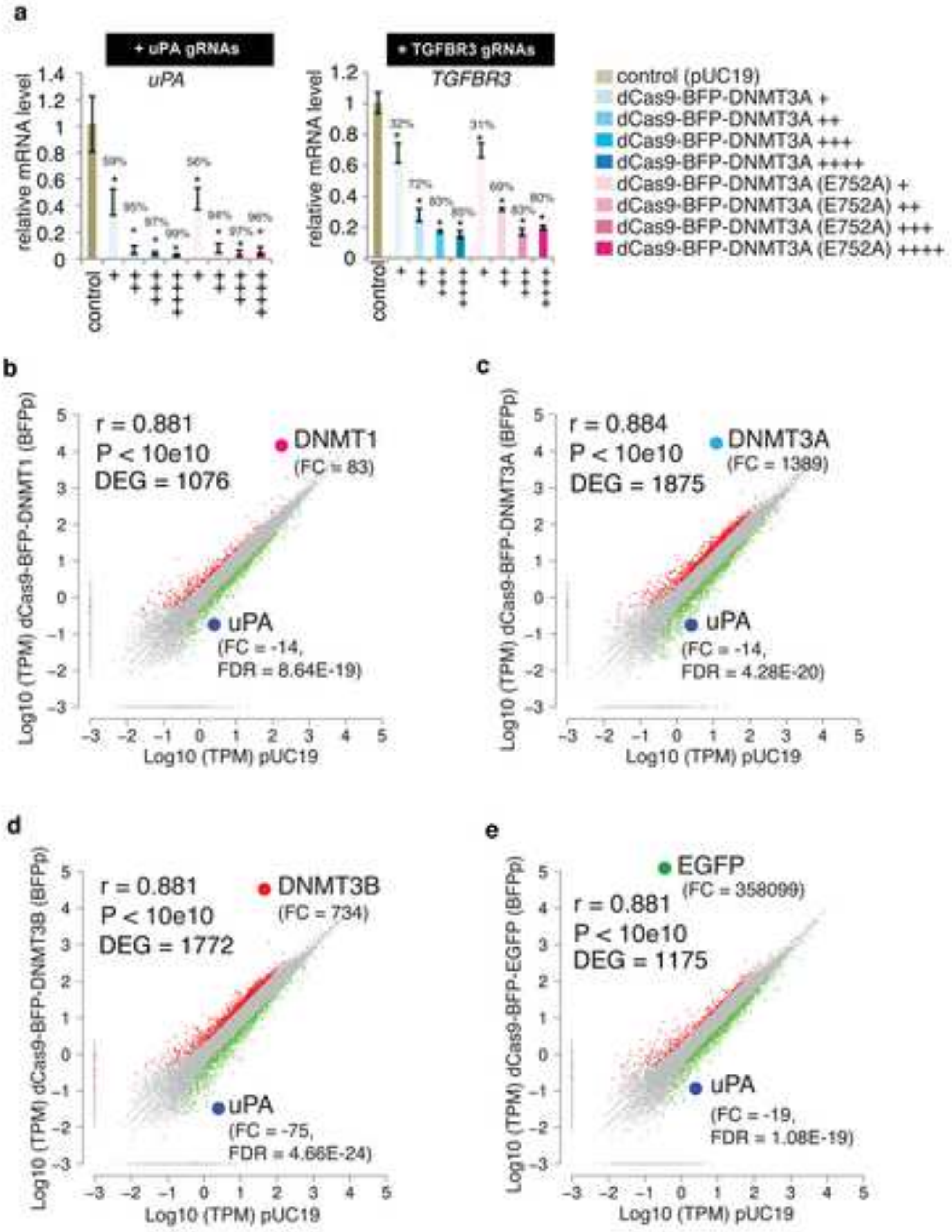

Figure 5

Click here to download Figure Fig 5 dpi300.tif

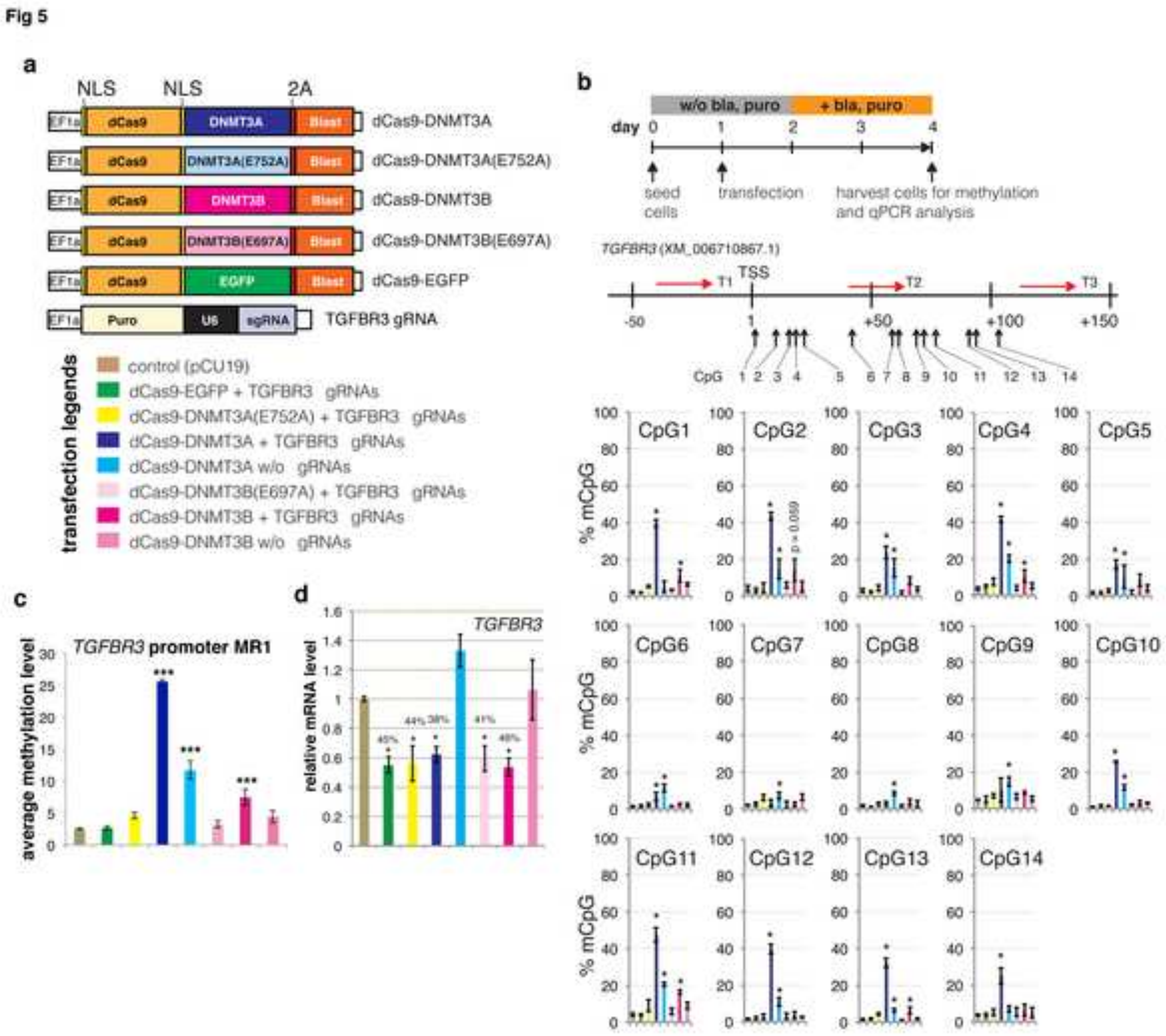

Figure 6

Click here to download Figure Fig 6 dpi300.tif

Fig 6

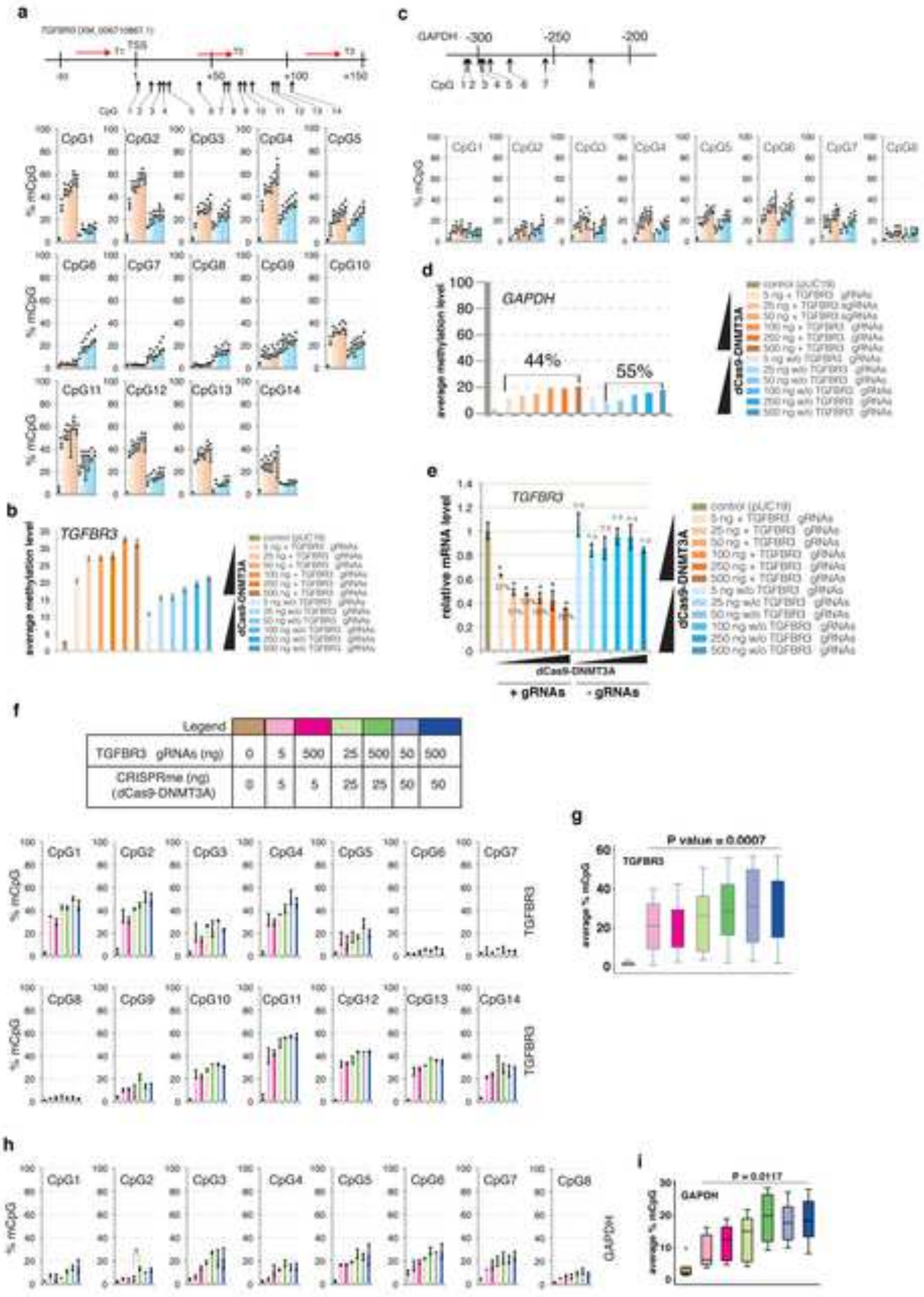

Fig. 7

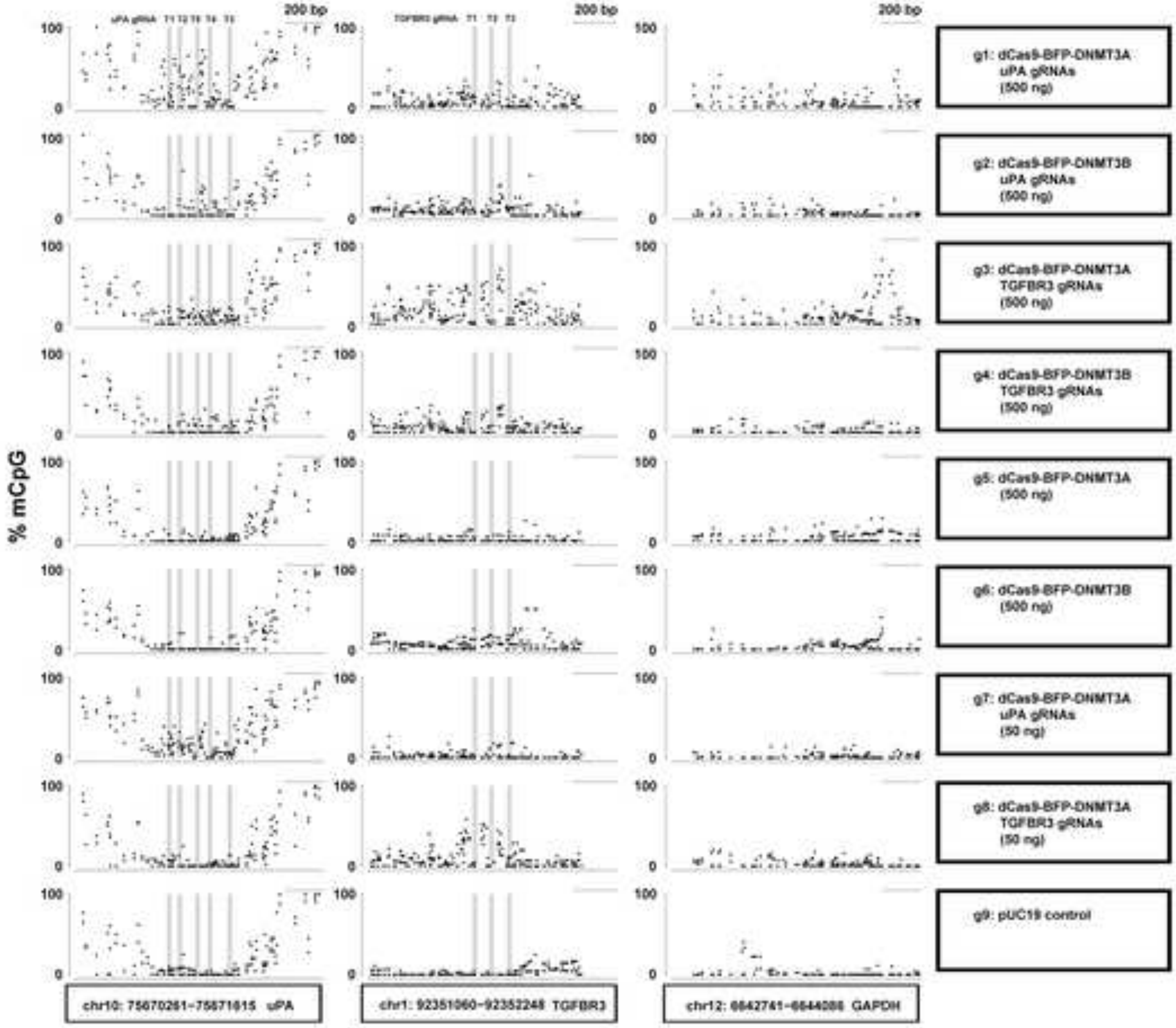
200 bp
% mCpG
100
0
g1: dCas9-BFP-DNMT3A uPA gRNAs (500 ng)
g2: dCas9-BFP-DNMT3B uPA gRNAs (500 ng)
g3: dCas9-BFP-DNMT3A TGFBR3 gRNAs (500 ng)
g4: dCas9-BFP-DNMT3B TGFBR3 gRNAs (500 ng)
g5: dCas9-BFP-DNMT3A (500 ng)
g6: dCas9-BFP-DNMT3B (500 ng)
g7: dCas9-BFP-DNMT3A uPA gRNAs (50 ng)
g8: dCas9-BFP-DNMT3A TGFBR3 gRNAs (50 ng)
g9: pUC19 control
chr10: 75670261-75671615 uPA
chr1: 92351060-92352248 TGFBR3

Figure 8

Click here to download Figure Fig 8 dpi300.tif

Fig. 8

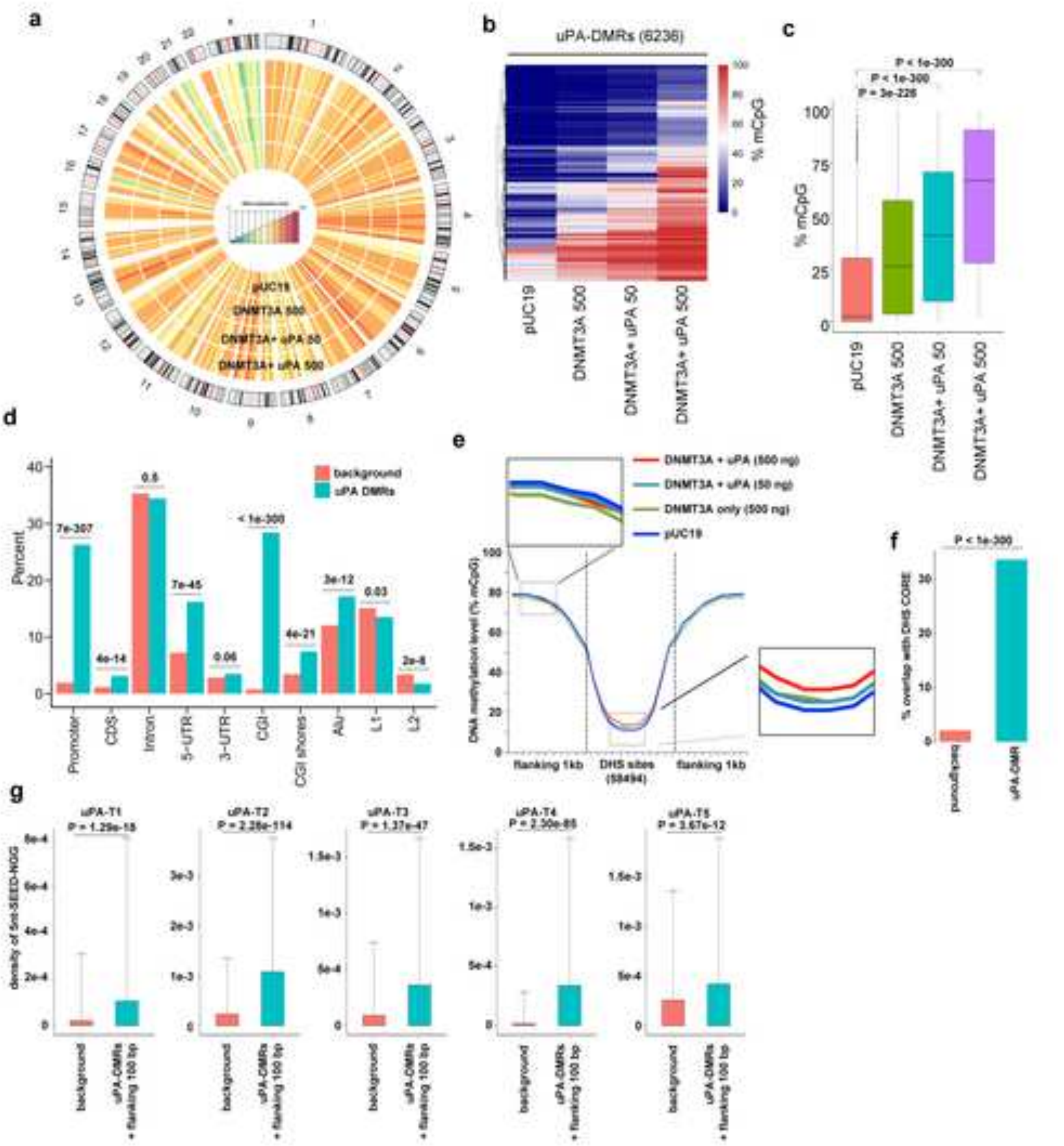

Figure 9

Click here to download Figure Fig 9 dpi300.tif

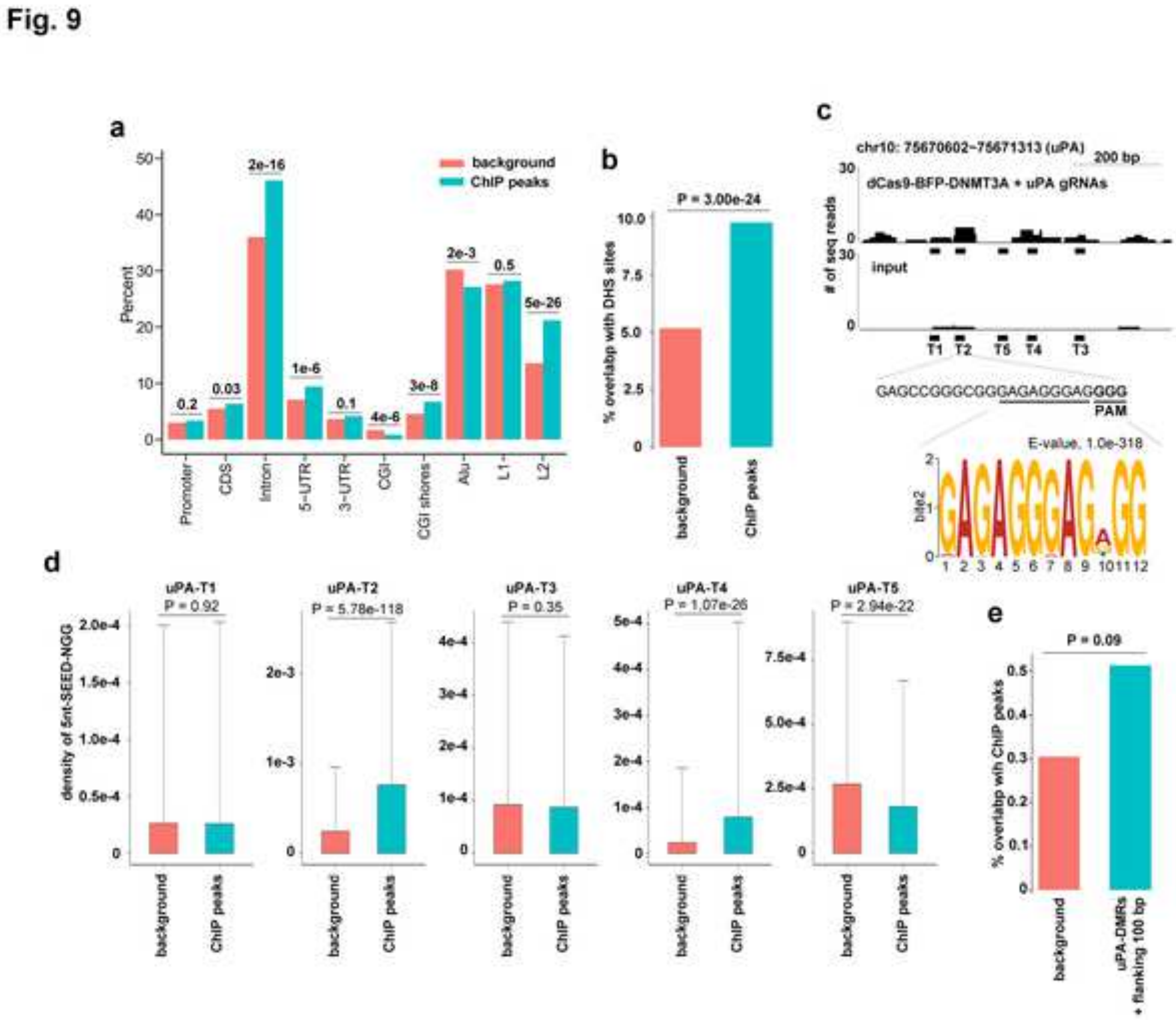

Figure S1

Click here to access/download
Supplementary Material
Fig.S1 dpi300.tif

Figure S2

Click here to access/download
Supplementary Material
Fig.S2 dpi300.tif

Figure S3

Click here to access/download
Supplementary Material
Fig.S3 dpi300.tif

Figure S4

Click here to access/download
Supplementary Material
Fig.S4 dpi300.tif

Figure S5

Click here to access/download
Supplementary Material
Fig.S5 dpi300.tif

Figure S6

Click here to access/download
Supplementary Material
Fig.S6 dpi300.tif

Figure S7

Click here to access/download

Supplementary Material

Fig.S7 dpi300.tif

Figure S8

Click here to access/download
Supplementary Material
Fig.S8 dpi300.tif

Figure S9

Click here to access/download
Supplementary Material
Fig.S9 dpi300.tif

Figure S10

Click here to access/download
Supplementary Material
Fig.S10 dpi300.tif

Figure S11

Click here to access/download
Supplementary Material
Fig.S11 dpi300.tif

Figure S12

Click here to access/download
Supplementary Material
Fig.S12 dpi300.tif

Figure S13

Click here to access/download
Supplementary Material
Fig.S13 dpi300.tif

Figure S14

Click here to access/download
Supplementary Material
Fig.S14 dpi300.tif

Figure S15

Click here to access/download
Supplementary Material
Fig.S15 dpi300.TIF

Supplementary Table 1

Click here to access/download
Supplementary Material
Supplementary Table 1. uPA-DMRs.xlsx

Supplementary Table 2

Click here to access/download
Supplementary Material
Supplementary Table 2. TGFBR3-DMRs.xlsx

Supplementary Table 3

Click here to access/download
Supplementary Material
Supplementary Table 3 ChIP peaks.xlsx

Supplementary Table 4

Click here to access/download
Supplementary Material
Supplementary Table 4.docx
