# Supplementary material for: Genome-wide determination of on-target and off-target characteristics for RNA-guided DNA methylation by dCas9 methyltransferases
Source: Gigascience. 2018 Feb 19;7(3):giy011. doi: 10.1093/gigascience/giy011 (PMC5888497; doi:10.1093/gigascience/giy011)
Supplement: GIGA-D-17-00040_Revision_2.pdf [file giy011_giga-d-17-00040_revision_2.pdf]

GigaScience

Genome-wide determination of on-target and off-target characteristics for RNA-guided DNA Methylation by dCas9 methyltransferases

--Manuscript Draft--

**Manuscript Number:**	GIGA-D-17-00040R2
**Full Title:**	Genome-wide determination of on-target and off-target characteristics for RNA-guided DNA Methylation by dCas9 methyltransferases
**Article Type:**	Research
**Funding Information:**	

Funder	Recipient
Teknologi og Produktion, Det Frie Forskningsråd (DFF–1337-00128)	Prof. Yonglun Luo
Teknologi og Produktion, Det Frie Forskningsråd (DFF-1335-00763A)	Prof. Yonglun Luo
Innovation Fund Denmark (BrainStem)	Prof. Yonglun Luo
Lundbeckfonden (R173-2014-1105)	Prof. Yonglun Luo
Lundbeckfonden (R151-2013-14439)	Prof. Lars Bolund
Lundbeckfonden (R219-2016-1375)	Dr. Lin Lin
Toyota Foundation (JP)	Prof. Anders Lade Nielsen
Lundbeckfonden	Prof. Anders Lade Nielsen

Abstract:

Background
Fusion of DNA methyltransferase domains to the nuclease-deficient clustered regularly interspaced short palindromic repeat (CRISPR) associated protein 9 (dCas9) has been used for epigenome editing, but the specificities of these dCas9 methyltransferases have not been fully investigated.

Findings
We generated CRISPR-guided DNA methyltransferases by fusing the catalytic domain of DNMT3A or DNMT3B to the C terminus of the dCas9 protein from S. pyogenes and validated its on-target and global off-target characteristics. Using targeted quantitative bisulfite pyrosequencing, we prove that dCas9-BFP-DNMT3A and dCas9-BFP-DNMT3B can efficiently methylate the CpG dinucleotides flanking its target sites at different genomic loci (uPA and TGFBR3) in human embryonic kidney cells (HEK293T). Furthermore, we conducted whole genome bisulfite sequencing (WGBS) to address the specificity of our dCas9 methyltransferases. WGBS revealed that although dCas9-BFP-DNMT3A and dCas9-BFP-DNMT3B did not cause global methylation changes, a substantial number (over 1000) of off-target differentially methylated regions (DMRs) were identified. The off-target DMRs, which were hypermethylated in cells expressing dCas9 methyltransferase and gRNAs, were predominantly found in promoter regions, 5' untranslated regions, CpG islands, and DNase I hypersensitivity sites, whereas, unexpected hypomethylated off-target DMRs were significantly enriched in repeated sequences. Through chromatin immunoprecipitation with massive parallel DNA sequencing analysis, we further revealed that these off-target DMRs were weakly correlated with dCas9 off-target binding sites. Using qPCR, RNA sequencing and fluorescence reporter cells, we also found that dCas9-BFP-DNMT3A and dCas9-BFP-DNMT3B can mediate transient inhibition of gene expression, which might be caused by dCas9-mediated de novo DNA methylation as well as interference with transcription.

Conclusion
Our results prove that dCas9 methyltransferases cause efficient RNA-guided methylation of specific endogenous CpGs. However, there is significant off-target methylation indicating that further improvements of the specificity of CRISPR-dCas9 based DNA methylation modifiers are required.

Powered by Editorial Manager® and ProduXion Manager® from Aries Systems Corporation

**Corresponding Author:**	Yonglun Luo<br>Aarhus Universitet Health<br>Aarhus, DENMARK
**Corresponding Author Secondary Information:**	
**Corresponding Author's Institution:**	Aarhus Universitet Health
**Corresponding Author's Secondary Institution:**	
**First Author:**	Lin Lin
**First Author Secondary Information:**	
**Order of Authors:**	Lin Lin
	Yong Liu
	Fengping Xu
	Jingrong Huang
	Tina Fuglsang Daugaard
	Trine Skov Petersen
	Bettina Hansen
	Lingfei ye
	Qing Zhou
	Fang Fang
	Ling Yang
	Shengting Li
	Lasse Fløe
	Kristopher Torp Jensen
	Ellen Shrock
	Fang Chen
	Huanming Yang
	Jian Wang
	Xin Liu
	Xun Xu
	Lars Bolund
	Anders Lade Nielsen
	Yonglun Luo
**Order of Authors Secondary Information:**	

**Response to Reviewers:**	Dear Editor and reviewers,<br><br>We really appreciate the reviewers' comments and concerns of the off-target effects we have discovered for dCas9 methyltransferases.<br><br>Of course, we fully aware of and understand that any finding of negative effects of the CRISPR technology should be carefully and thoroughly addressed before announcing it to the whole scientific and CRISPR community.<br><br>Unlike the original CRISPR/Cas9 technology, of which the endonuclease activity of Cas9 depends heavily on the base-pairing between the guide sequences and the

	target site (proto-spacer), the dead Cas9 (dCas9) derived CRISPR technology and applications are more depending on the physical interaction between dCas9/gRNA complex and the DNA loci, and more tolerance to mismatches. As already demonstrated in figure 2 and Supplementary Figure 4 of this study and several previous investigations by ChIP-seq, the criteria of defining off-target sites (based on mismatches) from wild type Cas9 is not suitable for the dCas9 methyltransferases. Although this study only evaluate the dCas9 methyltransferases, we speculate that this off-target effects are most likely to be the same for other kind of dCas9 based effectors.<br><br>We have conducted more WGBS validation experiment in the revision (Supplementary Figure 14). WGBS analyses are now conducted in HEK293T cells transfected with dCas9-BGP-DNMT3A and uPA gRNA (n = 3), and compared to transfection control (pUC19, n = 3). We validate that the hypermethylated DMRs found in our first WGBS experiments are significantly increased in cells expressing dCas9-BGP-DNMT3A and uPA gRNAs. Furthermore, we also validated DHS sites are prone to unspecific methylation. These results collectedly and consistently validate our finding that there is a certain degree of unspecific methylation causing by dCas9 methyltransferase and CRISPR gRNAs, and promoter region and open chromatin regions are prone to unspecific methylation.<br><br>Kr,<br>Yonglun Luo<br>Associate Professor, Department of Biomedicine, Aarhus University<br>Executive Director, Lars Bolund Institute of Regenerative Medicine, BGI-Qingdao, China
**Additional Information:**	
**Question**	**Response**
Are you submitting this manuscript to a special series or article collection?	No
**Experimental design and statistics**<br><br>Full details of the experimental design and statistical methods used should be given in the Methods section, as detailed in our Minimum Standards Reporting Checklist. Information essential to interpreting the data presented should be made available in the figure legends.<br><br>Have you included all the information requested in your manuscript?	Yes
**Resources**<br><br>A description of all resources used, including antibodies, cell lines, animals and software tools, with enough information to allow them to be uniquely identified, should be included in the Methods section. Authors are strongly encouraged to cite Research Resource Identifiers (RRIDs) for antibodies, model organisms and tools, where possible.	Yes

Powered by Editorial Manager® and ProduXion Manager® from Aries Systems Corporation

Have you included the information requested as detailed in our Minimum Standards Reporting Checklist?	
**Availability of data and materials**<br><br>All datasets and code on which the conclusions of the paper rely must be either included in your submission or deposited in publicly available repositories (where available and ethically appropriate), referencing such data using a unique identifier in the references and in the “Availability of Data and Materials” section of your manuscript.<br><br>Have you have met the above requirement as detailed in our Minimum Standards Reporting Checklist?	Yes

Powered by Editorial Manager® and ProduXion Manager® from Aries Systems Corporation

Manuscript

Click here to download Manuscript CRISPRme_R6 GigaScience.docx

Genome-wide determination of on-target and off-target characteristics for RNA-guided DNA Methylation by dCas9 methyltransferases

Lin Lin [1, 7, §], Yong Liu [1, §], Fengping Xu [2, 3, 4, §], Jinrong Huang [2, 3, 4,§], Tina Fuglsang Daugaard [1], Trine Skov Petersen [1], Bettina Hansen [1], Lingfei Ye [2], Qing Zhou [2, 3], Fang Fang [2, 3], Ling Yang [2, 3], Shengting Li [1, 2], Lasse Fløe [1], Kristopher Torp Jensen [1], Ellen Shrock [6], Fang Chen [2, 3, 4], Huanming Yang [2, 4, 5], Jian Wang [2, 4], Xin Liu [2, 3], Xun Xu [2, 3, *], Lars Bolund [1, 2, 3, 7, 9], Anders Lade Nielsen [1], Yonglun Luo [1, 2, 3, 7, 8, 9 *]

Initials for authors:

L.L., Y.Liu., F.X., J.H., T.F.D., T.S.P., B.H., L.Y., Q.Z., F.F., L.Y., S.L., L.F., K.T.J., E.S., F.C., H.Y., J.W., X.L., X.X., L.B., A.L.N., Y.L.

Affiliations for authors:

1. Department of Biomedicine, Aarhus University, Aarhus, Denmark
2. BGI-Shenzhen, Shenzhen 518083, China
3. China National GeneBank-Shenzhen, BGI-Research, Shenzhen 518083, China
4. Department of Biology, University of Copenhagen, Copenhagen, Denmark
5. James D. Watson Institute of Genome Sciences, Hangzhou 310058, China
6. Department of Genetics, Harvard Medical School, Boston, MA, USA.
7. Danish Regenerative Engineering Alliance for Medicine (DREAM), Department of Biomedicine, Aarhus University, Aarhus, Denmark
8. BrainStem - Stem Cell Center of Excellence in Neurology, Copenhagen, Denmark
9. BGI-Qingdao, 2877 Tuanjie Road, Sino-German Ecopark, Qingdao, 266000, China

§. These authors contributed equally to the study

*. All correspondence should be addressed to Xun Xu (XUXUN@GENOMICS.CN) and Yonglun Luo (ALUN@BIOMED.AU.DK)

Emails:

Lin Lin: lin.lin@biomed.au.dk

Yong Liu: liuyongbox@gmail.com

Jinrong Huang: huangjinrong@genomics.cn

Fengping Xu: xufengping@genomics.cn

Tina Fuglsang Daugaard: tfm@biomed.au.dk

Trine Skov Petersen: trinesp@biomed.au.dk

Bettina Hansen: bhansen@biomed.au.dk

Lingfei Ye: yelingfei@genomics.cn

Qing Zhou: zhouqing1@genomics.cn
Fang Fang: fangfang@genomics.org.cn
Ling Yang: yangling2@genomics.cn
Shengting Li: lishengting@gmail.com
Lasse Fløe: lassefloe@gmail.com
Kristopher Torp Jensen: kristopher.torp@gmail.com
Ellen Shrock: ellen_shrock@g.harvard.edu
Fang Chen: fangchen@genomics.cn
Huanming Yang: yanghm@genomics.cn
Jian Wang: wangjian@genomics.cn
Xin Liu: <liuxin@genomics.cn>
Xun Xu: xuxun@genomics.cn
Lars Bolund: bolund@biomed.au.dk
Anders Lade Nielsen: aln@biomed.au.dk
Yonglun Luo: alun@biomed.au.dk

1. Abstract

Background

Fusion of DNA methyltransferase domains to the nuclease-deficient clustered regularly interspaced short palindromic repeat (CRISPR) associated protein 9 (dCas9) has been used for epigenome editing, but the specificities of these dCas9 methyltransferases have not been fully investigated.

Findings

We generated CRISPR-guided DNA methyltransferases by fusing the catalytic domain of DNMT3A or DNMT3B to the C terminus of the dCas9 protein from *S. pyogenes* and validated its on-target and global off-target characteristics. Using targeted quantitative bisulfite pyrosequencing, we prove that dCas9-BFP-DNMT3A and dCas9-BFP-DNMT3B can efficiently methylate the CpG dinucleotides flanking its target sites at different genomic loci (*uPA* and *TGFBR3*) in human embryonic kidney cells (HEK293T). Furthermore, we conducted whole genome bisulfite sequencing (WGBS) to address the specificity of our dCas9 methyltransferases. WGBS revealed that although dCas9-BFP-DNMT3A and dCas9-BFP-DNMT3B did not cause global methylation changes, a substantial number (over 1000) of off-target differentially methylated regions (DMRs) were identified. The off-target DMRs, which were hypermethylated in cells expressing dCas9 methyltransferase and gRNAs, were predominantly found in promoter regions, 5' untranslated regions, CpG islands, and DNase I hypersensitivity sites, whereas, unexpected hypomethylated off-target DMRs were significantly enriched in repeated sequences. Through chromatin immunoprecipitation with massive parallel DNA sequencing analysis, we further revealed that these off-target DMRs were weakly correlated with dCas9 off-target binding sites. Using qPCR, RNA sequencing and fluorescence reporter cells, we also found that dCas9-BFP-DNMT3A and dCas9-BFP-DNMT3B can mediate transient inhibition of gene expression, which might be caused by dCas9-mediated *de novo* DNA methylation as well as interference with transcription.

Conclusion

Our results prove that dCas9 methyltransferases cause efficient RNA-guided methylation of specific endogenous CpGs. However, there is significant off-target methylation indicating that further improvements of the specificity of CRISPR-dCas9 based DNA methylation modifiers are required.

Key words

DNA methylation – CRISPR – Cas9 – DNMT3A – DNMT3B – dCas9 – specificity – off-targets – epigenome editing

2. Background

Owing to its simplicity, efficiency and potential for multiplicity, the type II Clustered Regularly Interspaced Short Palindromic Repeats (CRISPR) and CRISPR-associated protein 9 (Cas9) with engineered variants have been widely used for genome and epigenome editing in many species [1-5]. The Cas9 protein is guided to a specific genomic locus containing a protospacer adjacent motif (PAM) by a small single guide RNA (gRNA), which contains a conserved scaffold sequence and a programmable guide sequence (typically 20 nt) for base pairing with the taret [1]. By introducing double mutations (D10A and H840A) in the *S.pyogenes* Cas9 protein (dCas9) to inactivate its catalytic activity and fusing functional effectors to the C terminus of the dCas9, the applications of CRISPR/Cas9 are expanded to regulation of gene expression (CRISPRa and CRISPRi) [6-8], targeted DNA purification [9], visualization of specific gene regions [10], and acetylation or methylation of chromatin components [11, 12].

Genome-wide studies have revealed fundamental functional roles of DNA methylation as well as associations between aberrant DNA methylation and human diseases including cancer [13, 14]. Methylation of cytosine residues (5mC) in the mammalian genome mainly occurs at CpG dinucleotides. In promoter regions CpG methylation normally associated with repression of gene expression. Currently, insights into DNA methylation-associated biological processes are largely based on correlative data. Methods have been developed to methylate desired gene loci selectively by fusing programmable DNA binding proteins (zinc finger proteins (ZFs) or transcription-activator-like effectors (TALEs)) to DNA methyltransferases [3-9]. However, the laborious generation of ZFs- and TALEs hampers their broader applications. Engineered dCas9 has been harnessed for targeted DNA methylation by fusing dCas9 to the catalytic domain of mammalian DNA methyltransferases, thus providing an alternative tool for more easily programmable DNA methylation [15, 16].

Currently, genome-wide characterization of the specificity of dCas9-based epigenetic modifiers is lacking. To gain more insights into the efficiency and specificity of targeted DNA methylation by CRISPR gRNA-guided dCas9 methyltransferases, we used quantitative bisulfite pyrosequencing, whole genome bisulfite sequencing, and ChIP-seq to investigate the characteristics of dCas9 methyltransferase-mediated DNA methylation in human cells.

3. Methods

3.1 Cell Culture

Human embryonic kidney HEK293T cells (ATCC) were cultured in Dulbecco's modified Eagle's medium (DMEM, Life Technologies), 10% fetal bovine serum (Sigma), 1% penicillin-streptomycin (Sigma), 1X GlutaMAX (Life Technologies) at 37 °C, 5% CO2.

3.2 dCas9 methyltransferases plasmids

The dCas9 coding sequence was derived from pHR-SFFV-dCas9-BFP-KRAB (Addgene ID 46911) (a gift from Stanley Qi & Jonathan Weissman). The catalytic domains of DNMT1, DNMT3A and DNMT3B were PCR-amplified from pcDNA3/Myc-DNMT1 (Addgene ID 36939), pcDNA3/Myc-DNMT3A (Addgene ID 35521) and pcDNA3/Myc-DNMT3B1 (Addgene ID 35522) (a gift from Arthur Riggs), respectively. The DNMT3A (E752A) and DNMT3B (E697A) catalytically inactivating mutations were introduced by site-directed mutagenesis. All plasmids described in this study have been validated by Sanger sequencing and will be publically available through Addgene (https://www.addgene.org/Yonglun_Luo/) (**Supplementary Table S1).**

3.3 CRISPR gRNA design

Based on the observation that dCas9 methyltransferases could efficiently methylate the CpGs flanking the target sites, a web-based gRNA designing tool (dCas9 methyltransferases ***gRNA finder***, **http://luolab.au.dk/views/gRNA.cgi)** was developed to facilitate dCas9 methyltransferase-based gRNA design. All updates regarding the dCas9 methyltransferase protocol are available on the website (http://luolab.au.dk/). All gRNA sequences are listed in **Supplementary Table S1**.

3.4 Transfection and enrichment transfected cells

Unless stated elsewhere, cells were transfected with gRNAs (total 500 ng) and a dCas9 methyltransferase expression vector (500 ng) in six-well plates using X-tremeGENE 9 DNA transfection reagent (Roche). For single gRNA or pUC19 control transfections, the amount of plasmid added was equivalent to the total amount of plasmid added for multiple gRNA transfections. For BFP-based enrichment, cells were harvested 48 hours after transfection, and dCas9 methyltransferase-expressing cells were sorted by FACS. Briefly, transfected cells were harvested by trypsinization, washed twice with 2% FBS-PBS, and re-suspended in 500 µL 2% FBS-PBS. Cells were stained with Propidium Iodide (PI) before sorting. PI negative and BFP positive or negative cells were sorted with a 4 Laser BD Facs Aria III instrument. All transfections were performed in at least two independent experiments.

3.5 Quantitative PCR (qPCR)

Total RNA was extracted from cells with the RNeasy Plus Mini Kit (Qiagen, 74136) according to the manufacturer's instructions and quantified using a Nanodrop 1000 Spectrophotometer. The first strand cDNA was synthesized from 100-500 ng total RNA with the iScript cDNA synthesis kit (Bio-Rad, 170-8891) following the manufacturer's instructions. qPCR was performed in triplicate for each sample, using the Light Cycler 480 SYBR Green I Master mix (Roche Life Science, 04887352001) and a Light Cycler 480 qPCR machine. Each qPCR reaction contained 1 µL cDNA template (5 times diluted), 7.5 µL qPCR Master mix (2X), and 5 pmol of each qPCR primer in a total volume of 15 µL. The following qPCR program was used for *uPA, TGFBR3* and *GAPDH*: 1 cycle at 95 °C for 5 min; 45 cycles at 95 °C for 10s, 57 °C for 10s, and 72 °C for 10s during which the fluorescence signal was measured. The final product was subjected to melting curve analysis. Primers for qPCR are listed in **Supplementary Table S1**. Relative gene expression was calculated using the $2^{-\Delta\Delta CT}$ method by first normalizing to the internal control *GAPDH* (ΔCT) and then calibrating to the transfection control pUC19 (ΔΔCT) [17].

3.6 DNA methylation analysis by bisulfite pyrosequencing with PyroMark Q24

Genomic DNA was extracted using the DNeasy Blood & Tissue Kit (Qiagen, 69506) according to the manufacturer's instructions. A total of 200 ng of genomic DNA was bisulfite treated using the EpiTect Bisulfite Kit (Qiagen, 59104) according to the manufacturer's instructions. This converts unmethylated cytosines to uracils. The bisulfite converted DNA was eluted with 20 µL elution buffer provided by the kit. Bisulfite PCR reactions for all genes described in this study were performed in a 25 µl volume containing 0.15 µl Hotstar Taq polymerase (5U/µl) (New England Biolabs, M0495L), 2.5 µl 10xStandard buffer, 0.5 µl of 10 mM dNTPs, 1.0 µl of each primer (10 µM) and 1.5 µl bisulfite converted genomic DNA. PCR was performed under the following conditions: 95 ˚C for 5 min followed by 45 cycles of 94 ˚C for 30 sec, 58 ˚C for 1 min, and 72 ˚C for 45 sec, and, finally, by 72 ˚C for 7 min. 4 µL PCR product was checked by gel electrophoresis. Pyrosequencing was performed with the PyroMark Q24 Advanced Reagents (Qiagen, 970922) using 20 µL PCR product from the bisulfite treated DNA and 20 µL sequencing primer (0.375 µM) according to the PyroMark Q24 CpG protocol. The general degree of cytosine methylation was determined by pyrosequencing of the bisulfite converted genomic DNA, using the PyroMark Q24 Advanced system (Qiagen).

3.7 DNA methylation analysis by bisulfite Sanger sequencing

Bisulfite converted DNA was used as template for PCR amplifications with the BS specific PCR primers listed in **Supplementary Table S1**, using the DreamTaq DNA Polymerase (Life Technologies, EP0701). PCR products were gel purified, sub-cloned in a TA-cloning vector (Life Technologies, 450030) and transformed into chemically competent *E.coli* cells. Cell clones were

manually picked, sub-cultured in 250 ul LB medium overnight, lysed, subjected to Sanger sequencing and analyzed by BISMA [18].

3.8 Fluorescence reporter cell assay

Five stable fluorescence reporter cell clones were established by randomly inserting various copies of the CMV promoter-driven mCherry expression cassette into HEK293T (pLV-mCherry was a gift from Pantelis Tsoulfas, Addgene ID 36084). Cells were transfected separately with each dCas9 methyltransferase expression vector (50 ng) and gRNAs (total 50 ng) in 24-well plates. One-third of the transfected cells were seeded to a new plate every 2-3 days and the remainder used for flow cytometry analysis. Median mCherry intensity was measured with the BD LSRFortessa™ cell analyzer (FACS CORE facility, Aarhus University). Identical instrument settings and control beads were applied during the time course experiment to ensure valid comparison across different time points. 20,000 events were recorded for each sample. Flow cytometry data were analyzed using the Flowjo software.

3.9 Immunostaining

48 hours after transfection, cells were fixed with freshly-made 4% PFA for 15 min at room temperature, followed by three washes with DPBS. Cells were permeabilized in 0.3% Triton X-100 DPBS for 10 min and blocked in 5% goat serum-DPBS for 30 min. Cells were incubated with a primary rabbit anti-HA-tag antibody (C29F4, Cell Signaling 3724, 1:1000) overnight, followed by secondary antibody staining with Alexa Fluor 555 donkey anti-rabbit IgG (A-31572, Life technologies) at room temperature for 2 hours. Images were obtained with a confocal microscope (LSM710, Carl Zeiss).

3.10 Southern blot analysis

Genomic DNA (15 µg) was digested with *EcoRI* restriction enzyme overnight and then analyzed by gel electrophoresis with vacuum blotting. Primers for generating the mCherry probe are listed in **Supplementary Table S1**. Probe labelling was performed using the Prime-It II Random Primer Labeling Kit according to the manufacturer’s instructions. Pre-hybridization and hybridization steps were carried out at 42 °C. Excess probe was washed from the membrane with SSC buffer, and the hybridization pattern was visualized on X-ray film by autoradiography.

3.11 RNA sequencing

Integrity and quantity of extracted RNA was evaluated with an Agilent 2100 Bioanalyzer according to the manufacturer’s instructions. After DNase I treatment, mRNA was isolated with Oligo (dT) magnetic beads. Fragmentation buffer was added to generate short fragments of mRNA. cDNA was synthesized using the mRNA fragments as templates, resolved with EB buffer

for end repair and ligated with adaptors. After size selection and purification by agarose gel electrophoresis, cDNA with sizes of approximately 240 bp were used for PCR amplification (12 cycles) and library construction. Libraries were sequenced on an Ion Proton platform (>30 million reads per sample). Sequencing reads that contained low quality, adaptor, and/or short (< 30nt) read sequences were filtered out before mapping. tmap was used to align the clean reads to the hg19 UCSC RefSeq (RNA sequences, GRCh37). No more than 3 mismatches were allowed in the alignment. Gene expression levels were calculated by transforming uniquely mapped transcript reads to TPM (transcript per million) [19]. Differentially expressed genes were defined as genes with a Benjamini-Hochberg–adjusted P value (FDR) ≤ 0.001 and fold change ≥ 2 compared to pUC19 control.

3.12 ChIP-seq

HEK293T cells were transfected with dCas9 methyltransferase and five *uPA* gRNAs (triplicates). 48 hours after transfection, transfected cells were subjected to ChIP with a commercially available kit ChIP-IT Express Enzymatic (53009-AF, ActivMotif, distributed by Nordic Biolabs) and an anti-HA tag antibody (C29F4, Cell Signaling) according to the manufacturer's instructions. Next generation sequencing libraries were prepared for Chip and input samples. SE50 sequencing was performed on Illumina HiSeq2500. Clean reads were mapped to human genome hg19 using SOAP2 with the parameter "-p 4 -v 2 -s 35". Unique mapping reads was sampled randomly and equally (62723057 reads). Peaks were called using MACS with P value 1e-3 compared to the input samples. Common peaks found in the triplicates were selected. Furthermore, ChIP peaks loated in repeat sequences and rDNA were removed. Sequence motifs enriched within 70 bp of peak summits were identified using MEME-ChIP.

3.13 WGBS library preparation and sequencing

Genomic DNA was fragmented by sonication to a mean size of 250bp using a Bioruptor (Diagenode, Belgium), followed by the blunt-ending, dA addition to 3'-end, and adaptor ligation using the TruSeq Sample Preparation kit (Illumina Inc.) according to the manufacturer's instructions. Then, bisulfite conversion was conducted with the EZ DNA Methylation-Gold kit (ZYMO). The fragments with different insert size were excised from the same lane of a 2% TAE agarose gel. Products were purified by using QIAquick Gel Extraction kit (Qiagen) and amplified by 18 PCR cycles. The library quality was monitored using the Agilent 2100 BioAnalyzer (Agilent) and the concentration of the library was determined by quantitative PCR. Finally, the WGBS libraries were paired-end sequenced on Illumina HiSeq X Ten.

After filtering out adaptor and low-quality reads, a total of 953.7Gb 150bp paired-end clean data was generated. An average of 106Gb clean data was obtained for each sample. Clean reads were aligned to the human reference genome (hg19) by BSMAP(v2.74) with the parameter "-u -v

5 -z 33 -p 6 -n 0 -w 20 -s 16 -r 0 -f 10 -L 140” [20]. Only the CpG sites with read depths >=4 were taken into consideration for DNA methylation level calculation. The 48502 bp lambda DNA genome was used as an extra reference for calculating the bisulphite conversion rate. Nearly complete (>99%) bisulfite conversion was documented in all libraries. For repeat WGBS experiment, HEK293T cells were transfected with pUC19 as controls or transfected with dCas9-BFP-DNMT3A and uPA gRNAs. Transfections were conducted in triplicates. Genomic DNA were purified from all cells 48 hours after transfection without BFP-based FACS enrichment of transfected cells. WGBS library construction and sequencing were conducted as above but sequenced with less depth, of 10-15X coverage.

3.14 Identification of differentially methylated regions (DMRs) and attemps to exclude stochastic DMRs unrelated to the dCas9 methyltransferase treatment

The bioconductor package DSS was used to identify DMRs with the parameter "delta >=0.1, pvalue <= 0.01, CpG sites >= 3, DMR lenth >= 10 bp, smoothing window 100 bp". Since expressing high amount of dCas9-BFP-DNMT3A and either uPA or TGFBR3 gRNAs caused the highest *de novo* on-target methylation, we reasoned that the authentic off-target DMRs should be detected in these two comparisons. We first compared group 1 (dCas9-BFP-DNMT3A (500 ng) + uPA gRNAs (500 ng)) or group 3 (dCas9-BFP-DNMT3A (500 ng) + TGFBR3 gRNAs (500 ng)) to group 9 (pUC19 control).

Based on the observation of (1) dose- and gRNA-dependent *de novo* methylation of *uPA*, *TGFBR3 and GAPDH* by dCas9 methyltransferases and (2) dCas9-BGP-DNMT3A being more efficient than dCas9-BFP-DNMT3B, we reasoned that the authentic DMRs causes by dCas9 methyltransferases and uPA gRNAs should have a methylation pattern as described below:

Hypermethylated DMRs by dCas9 methyltransferases and uPA gRNAs should meet:

<u>% mCpG</u>:

(1) group 9 (pUC19) =< **group 5** (dCas9-DNMT-3A only (500 ng)) =< **group 7** (dCas9-DNMT-3A (50 ng) + uPA gRNAs (50 ng)) =< **group 1** (dCas9-DNMT-3A (500 ng) + uPA gRNAs (500 ng)).

(2) group 2 (dCas9-DNMT-3B (500 ng) + uPA gRNAs (500 ng)) =< **group 1** (dCas9-DNMT-3A (500 ng) + uPA gRNAs (500 ng))

(3) group 6 (dCas9-DNMT-3B (500 ng)) =< **group 2** (dCas9-DNMT-3B (500 ng) + uPA gRNAs (500 ng))

Hypomethylated DMRs by dCas9 methyltransferases and uPA gRNAs should meet:

<u>% mCpG</u>:

(1) group 9 (pUC19) >= **group 5** (dCas9-DNMT-3A only (500 ng)) >= **group 7** (dCas9-DNMT-3A (50 ng) + uPA gRNAs (50 ng)) >= **group 1** (dCas9-DNMT-3A (500 ng) + uPA gRNAs (500 ng)).

(2) group 2 (dCas9-DNMT-3B (500 ng) + uPA gRNAs (500 ng)) >= **group 1** (dCas9-DNMT-3A (500 ng) + uPA gRNAs (500 ng))

(3) group 6 (dCas9-DNMT-3B (500 ng)) >= **group 2** (dCas9-DNMT-3B (500 ng) + uPA gRNAs (500 ng))

Similarly, the authentic DMRs caused by dCas9 methyltransferases and *TGFBR3* gRNAs should have a methylation pattern as described below:

Hypermethylated DMRs by dCas9 methyltransferases and TGFBR3 gRNAs should meet:

<u>% mCpG</u>:

(1) group 9 (pUC19) =< **group 5** (dCas9-DNMT-3A only (500 ng)) =< **group 8** (dCas9-DNMT-3A (50 ng) + TGFBR3 gRNAs (50 ng)) =< **group 3** (dCas9-DNMT-3A (500 ng) + TGFBR3 gRNAs (500 ng)).

(2) group 4 (dCas9-DNMT-3B (500 ng) + TGFBR3 gRNAs (500 ng)) =< **group 3** (dCas9-DNMT-3A (500 ng) + TGFBR3 gRNAs (500 ng))

(3) group 6 (dCas9-DNMT-3B (500 ng)) =< **group 4** (dCas9-DNMT-3B (500 ng) + TGFBR3 gRNAs (500 ng))

Hypomethylated DMRs by dCas9 methyltransferases and TGFBR3 gRNAs should meet:

<u>% mCpG</u>:

(1) group 9 (pUC19) >= **group 5** (dCas9-DNMT-3A only (500 ng)) >= **group 8** (dCas9-DNMT-3A (50 ng) + TGFBR3 gRNAs (50 ng)) >= **group 3** (dCas9-DNMT-3A (500 ng) + TGFBR3 gRNAs (500 ng)).

(2) group 4 (dCas9-DNMT-3B (500 ng) + TGFBR3 gRNAs (500 ng)) >= **group 3** (dCas9-DNMT-3A (500 ng) + TGFBR3 gRNAs (500 ng))

(3) group 6 (dCas9-DNMT-3B (500 ng)) >= **group 4** (dCas9-DNMT-3B (500 ng) + TGFBR3 gRNAs (500 ng))

We applied this methylation level-based filtering criteria to further remove potential stochastic DMRs. The remaining DMRs were subjected to all analyses as described in this study.

3.15 Analysis of 5nt-SEED-NGG motif density

The 5nt-SEED-NGG density was calculated by counting the frequency of the sequence containing the 5 nt SEED sequences preceding a NGG site on either DNA strand. The PAM density was calculated by counting the frequency of PAM sites (NGG) on either DNA strand. The

median density with standard deviation is shown in the plots. Fisher's exact test was conducted to compare densities between different sequence datasets.

3.16 Statistics

All values in this study were presented as mean ± standard deviation. The one-way Analysis of Variance (ANOVA) with Bonferroni multiple testing, linear regression, Wilcoxon matched-pairs signed-rank test, Fisher's exact test and Benjamini-Hochberg–adjusted P value were used for statistical analysis. A p-value < 0.05 was considered statistically significant.

4 Results

4.1 On-target DNA methylation by dCas9 methyltransferases: dCas9-BFP-DNMT3A and dCas9-BFP-DNMT3B

In mammalian cells, DNA methylation is established by *de novo* DNA methyltransferases (DNMT3A and DNMT3B), and maintained upon replication by DNMT1 [21]. Using a similar approach as Vojta *et al.* and McDonald *et al.* [15, 16]., we fused DNMT1 catalytic domain, DNMT3A catalytic domain, DNMT3B catalytic domain or EGFP to the C-terminal end of dCas9 with a blue fluorescent protein (BFP) and a triple tandem repeated flexible linker (3XG4S, Gly-Gly-Gly-Gly-Ser) (**Fig. 1a and Supplementary Fig. S1a**). Enrichment of cells expressing the fusion dCas9 methyltransferases were validated by BFP-based Fluorescence Activated Cell Sorting (FACS) (**Supplementary Fig. S1b**) and immunofluorescence staining using anti-HA tag antibody (**Supplementary Fig. S1c**).

To validate that dCas9 methyltransferases can methylate endogenous CpGs, the dCas9 methyltransferases were first targeted by five gRNAs (*uPA* gRNA T1 to T5, **Fig. 1b**) to the *uPA* promoter, which contains a dense CpG island that is hypomethylated in human cancer cells [22]. HEK293T cells were transfected with *uPA* gRNAs and individual dCas9 fusion expression vectors. Following BFP-based FACS enrichment of transfected cells, the percentage of methylated CpGs (mCpGs) at individual CpG sites in the *uPA* promoter (uPA-MR1 and uPA-MR2 genomic regions) was quantified by bisulfite pyrosequencing (**Fig. 1c**). Compared to the pUC19 control, cells expressing *uPA* gRNAs and dCas9-BFP-DNMT3A or dCas9-BFP-DNMT3B, but not dCas9-BFP-DNMT1 or dCas9-BFP-EGFP, had significantly higher mCpG levels (*P* value < 0.01, ANOVA test). This is consistent with previous reports showing that the C-terminal catalytic domains of DNMT3A and DNMT3B, but not DNMT1, are active [23, 24]. The CpGs most efficiently de *novo* methylated were located 10-50 bp upstream and downstream of the gRNA target sites. CpGs located in the gRNA binding sites were not methylated by the dCas9 methyltransferases, most likely because CRISPR/dCas9 binding blocks the interaction of the methyltransferase domain with the CpGs (**Fig. 1c**). *De novo* methylation by dCas9-BFP-DNMT3A and gRNAs was further validated by bisulfite Sanger sequencing (**Supplementary Fig. S1d**).

To investigate dCas9 methyltransferase-mediated methylation of another genomic locus, we generated three gRNAs targeting the transforming growth factor beta receptor 3 (*TGFBR3*) promoter. Similar *de novo* methylation effects were observed for dCas9-BFP-DNMT3A or dCas9-BFP-DNMT3B with *TGFBR3* gRNAs (**Fig. 1d-g; Supplementary Fig. S2**). Our results collectively reveal that fusion of dCas9 to the catalytic domain of DNMT3A/3B can mediate targeted *de novo* DNA methylation.

4.2 Off-target methylation by dCas9 methyltransferases

Since high frequency off-target mutagenesis has been observed in previous applications of CRISPR-Cas9 [25], we investigated the specificity of dCas9 methyltransferases. For this purpose, we repeated the experiment with two additional controls: (1) cells expressing dCas9-BFP-DNMT3A or dCas9-BFP-DNMT3B only; (2) cells expressing dCas9-BFP-DNMT3A or dCas9-BFP-DNMT3B and three scrambled gRNAs (gRNAs targeting the CMV promoter). We found that expression of dCas9 methyltransferases and scrambled gRNAs could cause some unspecific *de novo* methylation of the *uPA* promoter, but at much lower levels compared to that obtained for *uPA* gRNAs (**Supplementary Fig. S3**). A slightly increased *uPA* promoter methylation, although not significant, was also observed in cells expressing dCas9 methyltransferase only (**Supplementary Fig. S3**).

To further assess the off-target methylation, we investigated three genomic regions with various sequence similarities to the *uPA* gRNA target sites: *SH2D3C* (3 mismatches**, Supplementary Fig. S4a**), *FAM221A* (3 mismatches, **Supplementary Fig. S4b**), and *GAPDH* promoter (9 mismatches, **Fig. 2a**). We did not observe significant changes in CpG methylation at *SH2D3C* and *FAM221A* genomic sites. Surprisingly, several CpG sites in the *GAPDH* promoter were significantly methylated in cells expressing dCas9-BFP-DNMT3A and *uPA, TGFBR3,* or scrambled (CMV) gRNAs (**Fig. 2b-c**). The same was observed, but to a lesser extent, in cells expressing dCas9-BFP-DNMT3B (**Fig. 2d-e**). This effect was less prominent in cells expressing dCas9 methyltransferase only, indicating that unspecific methylation of the *GAPDH* promoter is RNA-guided. Our results collectively reveal the existence of site dependent off-target methylation by dCas9 methyltransferases.

4.3 Effects of DNMT3A/3B catalytic activity and dCas9 methyltransferase expression level on on-target and off-target DNA methylation

De novo methylation by dCas9 methyltransferases could be mediated either by the catalytic activity of DNMT3A and DNMT3B, or by the recruitment of additional DNA methylation enzymes to the binding sites facilitated by protein interactions. To elucidate the mechanism of on-target and off-target DNA methylation, we introduced the E752A and E697A catalytically inactivating mutations [26] in the DNMT3A and DNMT3B catalytic domains, respectively. To investigate the effect of dCas9 methyltransferase expression levels on on-target and off-target DNA methylation, cells were sorted into four populations based on BFP signal intensity, a marker of dCas9 methyltransferase expression level: 1. very low: +; 2. low: ++; 3. medium: +++; and 4. high: ++++ (**Fig. 3a**). Bisulfite pyrosequencing analysis of the *uPA* (**Fig. 3b)** and *TGFBR3* (**Fig. 3c, Supplementary Fig. S5**) promoters revealed that only dCas9 methyltransferases but not dCas9 methyltransferase catalytic mutants cause dose-dependent *de novo* methylation, suggesting that

de novo on-target methylation by dCas9 methyltransferases is mediated by the catalytic activity of DNMT3A and DNMT3B.

We next investigated the effect of dCas9 methyltransferase expression level on off-target methylation by analyzing the *GAPDH* promoter methylation in the FACS-sorted cells with different BFP signal intensity (+, ++, +++, and ++++). Consistent with previous results, co-expression of dCas9-BFP-DNMT3A or dCas9-BFP-DNMT3B (**Fig. 4a, b**) with either *uPA* or *TGFBR3* gRNAs significantly increased *de novo* methylation of *GAPDH* promoter CpGs compared to cells expressing dCas9 methyltransferase without gRNAs or pUC19. Furthermore, titrating dCas9 methyltransferase expression levels decreased unspecific methylation of the *GAPDH* promoter (**Fig. 4a, b**). Similarly, methyltransferase catalytic mutants do not cause *de novo* methylation of GAPDH. Since *de novo* methylation of gRNA-targeted genes was also decreased by dCas9 methyltransferase titration (**Fig. 3**), our results collectively suggest that altering dCas9 methyltransferase expression levels cannot efficiently reduce unspecific methylation relative to targeted methylation.

To investigate global methylation levels, repetitive *LINE1* elements were investigated as they represent a surrogate marker for global DNA methylation [27]. We measured the *LINE1* 5'UTR methylation by bisulfite pyrosequencing which revealed that expression of dCas9-BFP-DNMT3A and *uPA* gRNAs did not result in significant *LINE1* methylation changes (**Fig. 4c**).

4.4 Genome-wide bisulfite sequencing revealed off-target methylation by dCas9 methyltransferases

Prompted by the unspecific methylation of *GAPDH* promoter by dCas9 methyltransferases, we investigated the genome-wide off-target methylation characteristics by CRISPR dCas9 methyltransferases using whole-genome bisulfite sequencing (WGBS). WGBS were conducted in HEK293T cells transfected with (i) pUC19 (control), (ii) dCas9-BFP-DNMT3A or dCas9-BFP-DNMT3B alone, and (iii) dCas9-BFP-DNMT3A or dCas9-BFP-DNMT3B with either *uPA* or *TGFBR3* gRNAs with two difference doses (50 ng or 500 ng) (**Supplementary Fig. S6a**). Using the Illumina HiSeq X platform, we generated over 100 giga bases (Gb) of clean data for each sample (more than 30X coverage with a 99.5% bisulfite conversion rate). This allowed us to analyze the methylation pattern at single-base pair resolution. Since mainly CpG dinucleotides are subject to methylation in HEK293T cells (**Supplementary Fig. S6b**), all following analyses are based on CpG methylation in the entire genome (approximately 40,000,000 CpG sites). We firstly examined *uPA, TGFBR3* and *GAPDH* promoter methylation as revealed by WGBS in all nine groups. WGBS confirmed that the *uPA* and *TGFBR3* gRNAs could target dCas9-BFP-DNMT3A or dCas9-BFP-DNMT3B to the *uPA* and *TGFBR3* loci and methylate CpGs flanking the

gRNA binding sites in a dose- and gRNA-dependent manner (**Fig. 5**). Furthermore, our WGBS data revealed that some dCas9 methyltransferase-mediated *de novo* methylation of *uPA, TGFBR3* and *GAPDH (off-target)* promoters occurred in a broad region surrounding the gRNA binding site.

Next, we analyzed the global DNA methylation profile. Consistent with the *LINE1* assay (**Fig. 3c**), expression of dCas9 methyltransferase alone or together with gRNAs was not associated with global methylation changes (**Supplementary Fig. S6c, d**). Since we have only one replicate per group and stochastic methylations frequently occur in cancer cells during cultivation [28], we analyzed the data with DSS-single (a method developed by Wu et al. for detecting differentially methylated regions (DMRs) from WGBS data without replicates [29]) to identify differentially methylated regions (DMRs) caused by dCas9 methyltransferase and gRNAs. Firstly, we compared cells transfected with dCas9 methyltransferases with or without gRNAs to control cells (transfected with pUC19 control plasmid). Over 10,000 hyper or hypo DMRs were identified by DSS-single (**Supplementary Fig. S7**). Secondly, based on the observation that: **(1)** there is dose- and gRNA-dependency of *uPA*, *TGFBR3* and *GAPDH* methylation by dCas9 methyltransferase and **(2)** dCas9-BFP-DNMT3A is more efficient than dCas9-BFP-DNMT3B, we applied a stringent filtering step to remove potentially stochastic DMRs. Following this filtering, we identified over 1000 DMRs resulting from dCas9 methyltransferase together with either *uPA* gRNAs (hypermethylated DMRs (hyper-DMRs) = 3671; hypomethylated DMRs (hypo-DMRs) = 1807) or TGFBR3 gRNAs (hyper-DMRs = 2267; hypo-DMRs = 1662) (**Supplementary Table S2-S5**). These DMRs were on average 63-81 bp and contained an average of 5-9 CpGs (**Supplementary Fig. S8**). The average methylation levels of these hyper/hypo-DMRs differ significantly between pUC19 control cells, cells expressing dCas9 methyltransferase only, cells expressing low amounts of dCas9 methyltransferase and gRNAs, and cells expressing high amounts of dCas9 methyltransferase and gRNAs (**Fig. 6a**, **Supplementary Fig. S9a**). Only a very small portion of the DMRs (hyper-DMRs = 192; hypo-DMRs = 81) were commonly found among DMRs caused by dCas9 methyltransferase and *uPA* compared to *TGFBR3* gRNAs (**Fig. 6b**), suggesting that the majority of the off-target DMRs are RNA-guided. Taken together, our WGBS result revealed that expression of dCas9 methyltransferases together with gRNAs can cause substantial off-target methylation.

4.5 Characteristics of dCas9 methyltransferase off-targets

To better describe the characteristics of dCas9 methyltransferase off-targets, we stratified hyper- and hypo-DMRs according to their localization in particular types of genomic regions, including promoters, coding sequences (CDS), introns, 5’ untranslated regions (5-UTR), 3-UTR, CpG islands (CGI), CGI shores, Alu sequences, LINE1 (L1) sequences, and LINE2 (L2) sequences.

Our results showed that hyper-DMRs were predominantly enriched in promoters, 5-UTR and CGI, whereas hypo-DMRs were enriched in repeated sequences Alu and LINE1 (**Fig. 6c-d**, **Supplementary Fig. S9b-c**). Consistent with this finding, a metaplot of average methylation levels for all genes before the DSS-single call also showed that transcription start site flanking regions (overlapping with promoters and 5'UTR) were hypermethylated in cells expressing dCas9 methyltransferase and gRNAs (**Supplementary Fig. 10**).

Since dCas9 preferentially binds open chromatin regions [30], we further analyzed DNase I hypersensitivity regions based on ENCODE data from HEK293T cells (GEO#: GSM1008573) and quantified the average methylation level in DNase I hypersensitivity sites (DHS) (as an indication of sites with an open chromatin state). The DHS flanking regions (1 kb upstream and downstream) were used as a control. Compared to cells transfected with pUC19, cells expressing dCas9 methyltransferase and gRNAs had significantly higher methylation levels in the DHS sites (P value < 0.05; Wilcoxon matched-pairs signed-rank test) (**Fig. 6e, Supplementary Fig. 9d**). Furthermore, only hyper-DMRs but not hypo-DMRs were significantly enriched in DHS (P value < 1e-300, Fisher's exact test, **Fig. 6f-g** and **Supplementary Fig. 9e-f**), which collectively suggests that open chromatin regions are prone to unspecific methylation by dCas9 methyltransferase and gRNAs.

Previous studies have discovered that complementary base pairing between gRNA guide sequences and the PAM-proximal 5nt region (5ntSEED-PAM) is crucial for off-target binding [30, 31]. We also assessed the density of individual gRNA 5ntSEED-PAM sequence (5'-**NNNNN**NGG-3') in the hyper- and hypo-DMRs. For each DMR, we included the 100-bp flanking sequences when calculating the presence of 5ntSEED-PAM sequence density. This is based on the previous observation that dCas9 methyltransferases methylate CpGs flanking the gRNA binding site. We consistently observed significant enrichment of 5ntSEED-PAM sequences for all gRNAs in the hyper-DMRs but not hypo-DMRs (**Fig. 6h, Supplementary Fig. 9g**). Taken together, this shows that, if guided by gRNAs, dCas9 methyltransferases can cause substantial off-target methylation of genomic regions with open chromatin accessibility i.e. promoters and 5'UTR, as well as CpG islands. Our finding between the off-target methylation and the chromatin accessibility is also consistent with our recent discovery that CRISPR/Cas9 cleaves more efficiently in euchromatin than heterochromatin regions [32].

4.6 dCas9 methyltransferase-mediated hypermethylated DMRs are weakly correlated with off-target binding

To further investigate the association between dCas9 methyltransferase off-target methylation and dCas9 off-target DNA binding, we studied off-target binding sites in HEK293T cells

expressing dCas9 methyltransferase and *uPA* gRNAs using ChIP-seq. Using pair-wise comparison as previous approach for dCas9 [31], 805 enriched peaks (P value < 0.001, **Supplementary Table S6**) were identified. These ChIP peaks were scattered throughout the genome and significantly enriched in DHS genomic regions (**Fig. 7a, b**). Using MEME motif scanning of ChIP peaks [33], we identified the most significant motif GGGAGAGGGAGNGG (P = 1.0e-593). This motif is identical to the 11-bp seed sequences of *uPA* gRNA T2 (GAGCCGGGC**GGGAGAGGGAG(GGG)**) and the PAM (NGG) site (**Fig. 7c**), suggesting that T2 is dominant compared to other *uPA* gRNAs in mediating off-target binding. Analysis of 5ntSEED-PAM sequence density further confirmed that uPA T2 binding sites were over-represented in the ChIP peaks (**Fig. 7d**). A previous study has shown that the choice of gRNAs has a great effect on dCas9 off-target binding [31]. The *uPA* gRNA T2 is highly G-rich or AG-rich in the seed region. This can potentially be the cause of most of the off-target activities. This could be the explanation of why we have found 40 times more off-target binding sites compared to the study by Liu et al. [34].

We next analyzed the correlation between the ChIP peaks and the uPA DMRs (including the flanking 100 bp of each DMR). There is a significantly increased overlap between ChIP peaks and *uPA* hyper-DMRs (p = 0.006, Fisher's exact test) but not *uPA* hypo-DMRs (p = 1, Fisher's exact test) (**Fig. 7e**). However, the percentage of *uPA* hyper-DMRs overlaps with ChIP peaks is still very low (11 out of 3671 hyper DMRs, 0.3%). Since the average methylation level of all ChIP peak regions exceeds 60% (**Supplementary Fig. 11**), and this may partially explain why there is a low correlation between ChIP peaks and DMRs given potential functional difficulty in further increasing the methylation level. Furthermore, ChIP-seq only identified sites to which the dCas9 methyltransferase binds strongly.

4.7 Effects of dCas9 methyltransferases on gene expression

Methylation of promoter DNA can be correlated with inhibition of gene transcription. To determine whether the dCas9 methyltransferase-mediated *uPA* and *TGFBR3* promoter methylation could inhibit gene expression, we measured *uPA* and *TGFBR3* mRNA levels by quantitative PCR (qPCR) in HEK293T cells. Compared to the pUC19 transfection control, both *uPA* and *TGFBR3* expression was significantly decreased in cells expressing dCas9-BFP-DNMT3A or dCas9-BFP-DNMT3B and either *uPA* or *TGFBR3* gRNAs (**Fig. 8a**). However, the reduced *uPA* and *TGFBR3* expression does not appear to be only associated with the *de novo* DNA methylation by dCas9 methyltransferases (**Fig. 8a**), as inactivating dCas9 methyltransferase mutants dCas9-BFP-DNMT3A(E752A) and dCas9-BFP-DNMT3B(E697A) also cause similar degrees of expression inhibition despite their lack of *de novo* DNA methylation activity.

To investigate whether the inhibition of gene expression is specific to the gRNA targeted genes, we conducted RNA sequencing in HEK293T cells expressing dCas9 methyltransferase and *uPA* gRNAs. A large number (> 1000) of differentially expressed genes (DEG) significantly (FDR *P* value < 0.001, fold change > 2) were found in cells expressing *uPA* gRNAs and either dCas9-BFP-DNMT3A or dCas9-BFP-DNMT3B (**Fig. 8b-c**). However, similar effects on the global transcription profile were observed in cells expressing *uPA* gRNAs with dCas9-BFP-DNMT1 or with dCas9-BFP-EGFP lacking *de novo* DNA methylation activity (**Fig. 8d-e**). We cross-compared DEGs among the four groups and 342 (18-32%) genes were commonly identified (**Fig. 8f**). For DEGs found in cells expressing dCas9-BFP-DNMT3A and uPA gRNAs, we also performed integrative analyses of the expression change, promoter methylation, and promoter binding intensity (**Fig. 8g**). Very weak but significant correlation was identified for a few clusters of DEGs. Taken together, these results suggest that the non-specific alteration of transcription is not merely caused by promoter methylation or binding of dCas9 methyltransferase. Since *uPA* is an important factor in regulating cell proliferation and inhibition of cell growth was found in cells expressing dCas9 methyltransferases and *uPA* gRNAs (**Supplementary Fig. S12**), the large number of differentially expressed genes might be a result of altered cellular functions. Taken together, our results clearly indicate that inhibition of *uPA* and *TGFBR3* expression by dCas9 methyltransferase and corresponding gRNAs is not merely due to *de novo* DNA methylation of their promoters.

To investigate whether longer term inhibition of gene expression can be facilitated by dCas9 methyltransferases, five HEK293T fluorescent reporter cell clones carrying different copies of a CMV-mCherry expression cassette (**Supplementary Fig. 13a, b**) were generated. We quantified mCherry level by FACS for two weeks after transfection. We observed that the number of dCas9 methyltransferase-expressing cells peaked on day 2 and decreased gradually (**Supplementary Fig. 13c**). Maximal inhibition of mCherry levels were observed on day 5 after transfection (**Supplementary Fig. 13d-h**). Compared to other dCas9 fusion proteins, the dCas-BFP-DNMT3A fusion resulted in the highest and longest inhibition of mCherry expression in the reporter cells (four out of five clones) (**Supplementary Fig. 13d-h**). The transient and prolonged inhibition efficacy varied among the five cell clones. For example, clone 2, which has the lowest copy number of transgene, showed the highest transient and longest inhibition by dCas-BFP-DNMT3A (**Supplementary Fig. 13e)**. However, expression of mCherry was, in all clones, not significantly different from the pUC19 control after two weeks, suggesting that inhibition of gene expression by dCas9 methyltransferases is not stably maintained.

Discussion

Since dCas9 methyltransferases are targeted to a specific genomic locus simply by a small gRNA, this system is more convenient than ZF- or TALE-based methyltransferases [26, 35, 36]. Recently, Vojta *et al.* and McDonald *et al.* reported that directly fusing DNMT3A to dCas9 could be used to induce DNA methylation at specific loci in HEK293T cells [15, 16]. Consistent with that, we show that dCas9-BFP-DNMT3A can methylate CpGs flanking the gRNA binding sites in genomic loci, further proving the general applicability of dCas9 methyltransferases for targeted DNA methylation in mammalian cells. In addition, our study shows for the first time that the fusion of dCas9 to DNMT3B is also capable of inducing specific DNA methylation, although the efficiency is lower than that of DNMT3A. Additionally, Peter *et al*. showed that the dCas9-DNMT3A-DNMT3L fusion can further improve *de novo* methylation efficiency compared to dCas9-DNMT3A [37]. Together with the reported systems, the dCas9 methyltransferases system reported in this study further broadens the availability and applicability of CRISPR-based reprogramming of DNA methylation. Based on the observation that dCas9 methyltransferases can efficiently methylate the flanking CpG sites from the gRNA binding site, we have developed an open-source web-based gRNA designing tool for dCas9 methyltransferase gRNAs (http://luolab.au.dk/views/gRNA.cgi).

On the basis of extensive gene-specific bisulfite pyrosequencing and whole-genome bisulfite sequencing (WGBS), we identified novel off-target methylation characteristics that appear to be predominantly enriched in promoter, 5'UTR, CGI, and open chromatin regions. Since most of these genomic regions are hypomethylated in HEK293T cells, it was expected that the off-target DMRs were enriched in such regions. In other genomic regions, which already have a high level of methylation, a further methylation by dCas9 methyltransferase is not achievable. We discovered that open chromatin regions are highly prone to off-target methylation by dCas9-methyltransferase. Since the *GAPDH* promoter is located a DHS region, this explains why this region is subjected to highly off-target methylation. To further confirm our finding, we repeat the WGBS experiment in triplicates (**Supplementary Fig. 14**). Our result confirmed that the hyper-methylated DMRs identified in previous WGBS experiment (**Fig. 6a**) are significantly increased in cells overexpressing dCas9-BFP-DNMT3A and *uPA* in the repeated experiments (**Supplementary Fig. 14a**). Consistently, DHS regions were significantly methylated in cells expressing dCas9-BFP-DNMT3A and *uPA* (**Supplementary Fig. 14b**).

Our study also revealed the gRNA-dependency of off-target methylation. This is consistent with the observations of McDonald *et al.* and Vojta *et al.* [15, 16]. Additionally, we have discovered that even in the absence of gRNAs, expression of the dCas9-BFP-DNMT3A or dCas9-BFP-DNMT3B alone can cause some unspecific DNA methylation. This gRNA-independent off-target methylation effect is even more pronounced when too many dCas9 methyltransferases, or the

DNMT3A catalytic domain, enter the nucleus. For example, increasing dCas9 methyltransferase expression level, fusing the catalytic domain of DNMT3A or DNMT3B directly to Cas9 without the BFP linker, or overexpressing the DNMT3A catalytic domain will cause increased gRNA-independent off-target methylation (see extended data and description in **Supplementary File 1**).

In this study, we found that expressing dCas9 methyltransferases and gRNAs could also cause significant demethylation of genomic regions enriched in repeated sequences. Repeated sequences, which make up more than half of the human genome, are generally highly methylated, and their dynamics, to some extent, are associated with normal development and tumorigenesis. A previous study of methylation in repeated sequences has shown that, with increasing age from adulthood, there is a global decrease in DNA methylation in repeated sequences and intergenic genome sequences [38]. We also observed that expression of dCas9 methyltransferase alone or together with gRNA can inhibit HEK293T cell growth (**Supplementary Fig. 12**). The hypo-methylated DMRs could potentially the result of inhibited cell proliferation by dCas9 methyltransferase and gRNAs. This should be investigated in future studies.

Improvement of dCas9 methyltransferase specificity, to minimize the gRNA-dependent and gRNA-independent off-target activity, is crucial for future applications of the technology. McDonald, et al., has observed significant reduction in off-target methylation using DOX inducible dCas9-DNMT3A. Consistent with these findings, we found that reducing the dCas9 methyltransferase and gRNA expression levels, as well as lowering the dCas9 methyltransferase nuclear entry efficiency (**Supplementary File 1**), can reduce off-target methylation. However, this approach also reduced on-target methylation levels accordingly. Thus, this may not represent a plausible way of increasing the specificity of the system. New approaches should be developed to reduce off-target methylation while maintaining sufficient on-target methylation efficiencies. The results presented in this study highlight the importance of inclusion of extensive controls in subsequent experiments, such as catalytically inactive dCas9 methyltransferase mutants, scrambled gRNAs, and gRNA free settings. This is necessary for reliable interpretations of correlations between specific DNA methylation events by dCas9 methyltransferase, gene expression regulation and phenotypic effects.

In this study, we have observed that dCas9 methyltransferases can efficiently inhibit expression of genes in human cells. However, the transient inhibition of gene expression could be resulted from both promoter methylation and blockage of transcription by dCas9 methyltransferases. A previous study reported that targeted DNA methylation by a zinc finger-based methyltransferase is not stably maintained [39]. Our time-course experiments to study the inhibition of gene expression is gradually decreased during *in vitro* expansion of the transfected cells. This could be

the result of removal of the *de novo* established epigenetic marks, dilution of the dCas9 methyltransferase expression plasmids, and/or negative selection of the cells expressing dCas9 methyltransferases. We also realize that DNA methylation and gene expression analyses were conducted in cells transiently transfected with dCas9 methyltransferase expression plasmids, which might lead to severe overexpression of the dCas9 methyltransferases. Thus, future studies could benefit from being conducted in cells stably or conditionally expressing low copy numbers of dCas9 methyltransferase to minimize off-target methylation. Taken together, our study is the first to reveal novel characteristics of the on-target and off-target DNA methylation by dCas9 methyltransferases on a genome-wide scale with single-base resolution and highlights the need for development of CRISPR DNA methylation editing systems with higher specificity.

Conclusions

The dCas9 methyltransferases presented here, and other dCas9 fusion protein systems described previously [11, 12, 15, 16], provide useful tools for targeted epigenome editing. Continued improvement of the specificities of these systems and combining tools to enable simultaneous modification of multiple histones and DNA loci will enable more precise and stable regulation of gene structure and function. Such CRISPR gRNA-guided programmable epigenetic modification tools will hopefully have broad research applications to delineate the association between specific epigenetic changes, gene-expression regulation, and phenotypes.

Availability of supporting data

RNA sequencing, WGBS, and ChIP-seq data are available from the publicly available repository (GEO).

RNA-seq: GSE74935

WGBS: GSE92310, GSE92311

ChIP-seq: GSE92261

Declarations

Competing Interests Statement

The authors declare no competing financial interests.

Author contributions

L.L., L.B. and Y.L., conceived the idea.

H.Y., J.W., L.B., X.X., A.L.N., and Y.L. planned and oversaw the study

L.L., Y.Liu., F.X., J.H., T.F.D., T.S.P., B.H., L.Y., Q.Z., F.F., S.L., K.T.J. L.F., E.S., and Y.L. performed experiments and analyzed the data.

L.L., J.H., and Y.L. prepared the figures.

L.L. and Y.L. drafted the manuscript and all authors revised the manuscript.

Acknowledgements

This work was partially supported by grants from Danish Research Council for Independent Research DFF–1337-00128 (Y.L.), the Sapere Aude Young Research Talent Prize DFF-1335-00763A (Y.L.), the Innovation Fund Denmark (BrainStem, Y.L.) and the Lundbeck Foundation: R173-2014-1105 (Y.L.); R151-2013-14439 (L.B.); R219-2016-1375 (L.L.) A.L.N. was supported by the Toyota-Foundation and the Lundbeck Foundation. FACS was performed with help from Charlotte Christie Petersen and Anni Skovbo at the FACS Core Facility, Aarhus University, Denmark.

Abbreviations

CRISPR: Clustered Regularly Interspaced Short Palindromic Repeats
Cas9: CRISPR-associated protein 9
dCas9: Nuclease deficient Cas9 or dead Cas9
FACS: Fluorescence-activated cell sorting
WGBS: whole-genome bisulfite sequencing
CGI: CpG island
UTR: Untranslated region
DHS: DNase I hypersensitivity sites
PAM: Protospacer adjacent motif
gRNA: guide RNA
ZF: zinc finger protein
TALE: transcription-activator-like effectors
qPCR: Quantitative PCR

Reference:

1. Jinek M, Chylinski K, Fonfara I, Hauer M, Doudna JA, Charpentier E: **A programmable dual-RNA-guided DNA endonuclease in adaptive bacterial immunity.** *Science* 2012, **337:**816-821.
2. Mali P, Yang L, Esvelt KM, Aach J, Guell M, Dicarlo JE, Norville JE, Church GM: **RNA-Guided Human Genome Engineering via Cas9.** *Science* 2013.
3. Jinek M, East A, Cheng A, Lin S, Ma E, Doudna J: **RNA-programmed genome editing in human cells.** *elife* 2013, **2:**e00471.
4. Cong L, Ran FA, Cox D, Lin S, Barretto R, Habib N, Hsu PD, Wu X, Jiang W, Marraffini LA, Zhang F: **Multiplex genome engineering using CRISPR/Cas systems.** *Science* 2013, **339:**819-823.
5. Johan Vad-Nielsen LL, Lars Bolund, Anders Lade Nielsen, Yonglun Luo **Golden-Gate assembly of CRISPR gRNA Expression Array for Simultaneously Targeting Multiple Genes.** *Cell Mol Life Sci* 2016.
6. Qi LS, Larson MH, Gilbert LA, Doudna JA, Weissman JS, Arkin AP, Lim WA: **Repurposing CRISPR as an RNA-Guided Platform for Sequence-Specific Control of Gene Expression.** *Cell* 2013, **152:**1173-1183.
7. Cheng AW, Wang H, Yang H, Shi L, Katz Y, Theunissen TW, Rangarajan S, Shivalila CS, Dadon DB, Jaenisch R: **Multiplexed activation of endogenous genes by CRISPR-on, an RNA-guided transcriptional activator system.** *Cell Res* 2013, **23:**1163-1171.
8. Gilbert LA, Larson MH, Morsut L, Liu Z, Brar GA, Torres SE, Stern-Ginossar N, Brandman O, Whitehead EH, Doudna JA, et al: **CRISPR-mediated modular RNA-guided regulation of transcription in eukaryotes.** *Cell* 2013, **154:**442-451.
9. Fujita T, Fujii H: **Efficient isolation of specific genomic regions and identification of associated proteins by engineered DNA-binding molecule-mediated chromatin immunoprecipitation (enChIP) using CRISPR.** *Biochem Biophys Res Commun* 2013, **439:**132-136.
10. Chen B, Gilbert LA, Cimini BA, Schnitzbauer J, Zhang W, Li GW, Park J, Blackburn EH, Weissman JS, Qi LS, Huang B: **Dynamic imaging of genomic**

loci in living human cells by an optimized CRISPR/Cas system. *Cell* 2013, **155:**1479-1491.

11. Kearns NA, Pham H, Tabak B, Genga RM, Silverstein NJ, Garber M, Maehr R: **Functional annotation of native enhancers with a Cas9-histone demethylase fusion.** *Nat Methods* 2015, **12:**401-403.

12. Hilton IB, D'Ippolito AM, Vockley CM, Thakore PI, Crawford GE, Reddy TE, Gersbach CA: **Epigenome editing by a CRISPR-Cas9-based acetyltransferase activates genes from promoters and enhancers.** *Nat Biotechnol* 2015, **33:**510-517.

13. Jones PA, Baylin SB: **The fundamental role of epigenetic events in cancer.** *Nat Rev Genet* 2002, **3:**415-428.

14. Brena RM, Costello JF: **Genome-epigenome interactions in cancer.** *Hum Mol Genet* 2007, **16 Spec No 1:**R96-105.

15. Vojta A, Dobrinic P, Tadic V, Bockor L, Korac P, Julg B, Klasic M, Zoldos V: **Repurposing the CRISPR-Cas9 system for targeted DNA methylation.** *Nucleic Acids Res* 2016.

16. McDonald JI, Celik H, Rois LE, Fishberger G, Fowler T, Rees R, Kramer A, Martens A, Edwards JR, Challen GA: **Reprogrammable CRISPR/Cas9-based system for inducing site-specific DNA methylation.** *Biol Open* 2016, **5:**866-874.

17. Livak KJ, Schmittgen TD: **Analysis of relative gene expression data using real-time quantitative PCR and the 2(-Delta Delta C(T)) Method.** *Methods* 2001, **25:**402-408.

18. Rohde C, Zhang Y, Reinhardt R, Jeltsch A: **BISMA--fast and accurate bisulfite sequencing data analysis of individual clones from unique and repetitive sequences.** *BMC Bioinformatics* 2010, **11:**230.

19. Wagner GP, Kin K, Lynch VJ: **Measurement of mRNA abundance using RNA-seq data: RPKM measure is inconsistent among samples.** *Theory Biosci* 2012, **131:**281-285.

20. Xi Y, Li W: **BSMAP: whole genome bisulfite sequence MAPping program.** *BMC Bioinformatics* 2009, **10:**232.

21. Law JA, Jacobsen SE: **Establishing, maintaining and modifying DNA methylation patterns in plants and animals.** *Nat Rev Genet* 2010, **11:**204-220.

22. Pakneshan P, Szyf M, Farias-Eisner R, Rabbani SA: **Reversal of the hypomethylation status of urokinase (uPA) promoter blocks breast cancer growth and metastasis.** *J Biol Chem* 2004, **279:**31735-31744.

23. Gowher H, Jeltsch A: **Molecular enzymology of the catalytic domains of the Dnmt3a and Dnmt3b DNA methyltransferases.** *J Biol Chem* 2002, **277:**20409-20414.

24. Margot JB, Aguirre-Arteta AM, Di Giacco BV, Pradhan S, Roberts RJ, Cardoso MC, Leonhardt H: **Structure and function of the mouse DNA methyltransferase gene: Dnmt1 shows a tripartite structure.** *J Mol Biol* 2000, **297:**293-300.

25. Fu Y, Foden JA, Khayter C, Maeder ML, Reyon D, Joung JK, Sander JD: **High-frequency off-target mutagenesis induced by CRISPR-Cas nucleases in human cells.** *Nat Biotechnol* 2013.

26. Rivenbark AG, Stolzenburg S, Beltran AS, Yuan X, Rots MG, Strahl BD, Blancafort P: **Epigenetic reprogramming of cancer cells via targeted DNA methylation.** *Epigenetics* 2012, **7:**350-360.

27. Yang AS, Estecio MR, Doshi K, Kondo Y, Tajara EH, Issa JP: **A simple method for estimating global DNA methylation using bisulfite PCR of repetitive DNA elements.** *Nucleic Acids Res* 2004, **32:**e38.

28. Landan G, Cohen NM, Mukamel Z, Bar A, Molchadsky A, Brosh R, Horn-Saban S, Zalcenstein DA, Goldfinger N, Zundelevich A, et al: **Epigenetic polymorphism and the stochastic formation of differentially methylated regions in normal and cancerous tissues.** *Nat Genet* 2012, **44:**1207-1214.

29. Wu H, Xu T, Feng H, Chen L, Li B, Yao B, Qin Z, Jin P, Conneely KN: **Detection of differentially methylated regions from whole-genome bisulfite sequencing data without replicates.** *Nucleic Acids Res* 2015, **43:**e141.

30. Kuscu C, Arslan S, Singh R, Thorpe J, Adli M: **Genome-wide analysis reveals characteristics of off-target sites bound by the Cas9 endonuclease.** *Nat Biotechnol* 2014, **32:**677-683.

31. Wu X, Scott DA, Kriz AJ, Chiu AC, Hsu PD, Dadon DB, Cheng AW, Trevino AE, Konermann S, Chen S, et al: **Genome-wide binding of the CRISPR endonuclease Cas9 in mammalian cells.** *Nat Biotechnol* 2014, **32:**670-676.

32. Jensen KT, Floe L, Petersen TS, Huang J, Xu F, Bolund L, Luo Y, Lin L: **Chromatin accessibility and guide sequence secondary structure affect CRISPR-Cas9 gene editing efficiency.** *FEBS Lett* 2017.

33. Bailey TL, Elkan C: **Fitting a mixture model by expectation maximization to discover motifs in biopolymers.** *Proc Int Conf Intell Syst Mol Biol* 1994, **2:**28-36.

34. Liu XS, Wu H, Ji X, Stelzer Y, Wu X, Czauderna S, Shu J, Dadon D, Young RA, Jaenisch R: **Editing DNA Methylation in the Mammalian Genome.** *Cell* 2016, **167:**233-247 e217.

35. Bernstein DL, Le Lay JE, Ruano EG, Kaestner KH: **TALE-mediated epigenetic suppression of CDKN2A increases replication in human fibroblasts.** *J Clin Invest* 2015, **125:**1998-2006.

36. Meister GE, Chandrasegaran S, Ostermeier M: **Heterodimeric DNA methyltransferases as a platform for creating designer zinc finger methyltransferases for targeted DNA methylation in cells.** *Nucleic Acids Res* 2010, **38:**1749-1759.

37. Stepper P, Kungulovski G, Jurkowska RZ, Chandra T, Krueger F, Reinhardt R, Reik W, Jeltsch A, Jurkowski TP: **Efficient targeted DNA methylation with chimeric dCas9-Dnmt3a-Dnmt3L methyltransferase.** *Nucleic Acids Res* 2016.

38. Suelves M, Carrio E, Nunez-Alvarez Y, Peinado MA: **DNA methylation dynamics in cellular commitment and differentiation.** *Brief Funct Genomics* 2016, **15:**443-453.

39. Kungulovski G, Nunna S, Thomas M, Zanger UM, Reinhardt R, Jeltsch A: **Targeted epigenome editing of an endogenous locus with chromatin modifiers is not stably maintained.** *Epigenetics Chromatin* 2015, **8:**12.

Figure captions

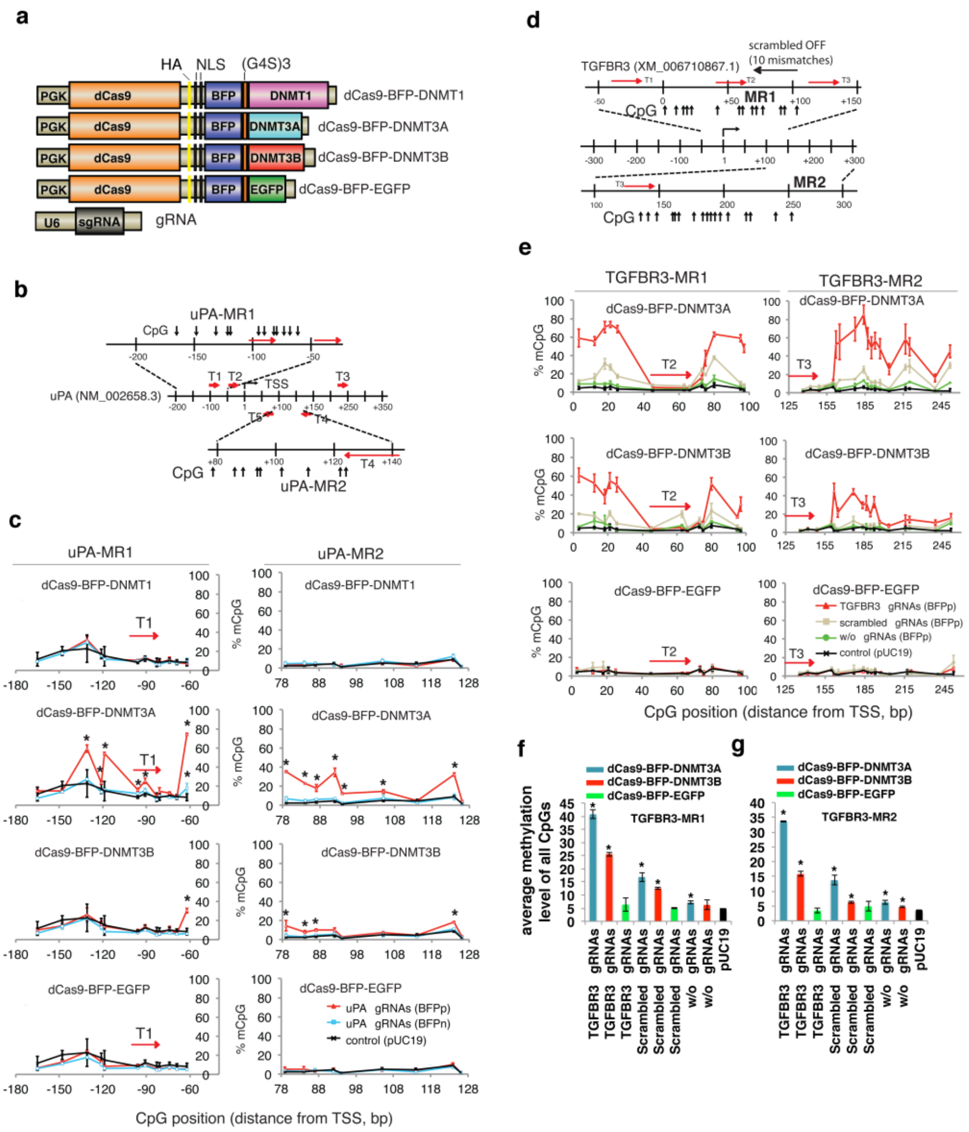

Fig. 1 *De novo uPA* and *TGFBR3* methylation by RNA-guided dCas9 methyltransferases

(**a**) Schematic illustration of the dCas9 methyltransferase expression vectors. PGK: phosphoglycerate kinase promoter; G4S: GGGGS linker; NLS: nuclear localization signal; U6: human U6 promoter.

(**b**) Schematic illustration of the *uPA* promoter and gRNA target sites (T1-T5), two *uPA* methylated regions (uPA-MR1, uPA-MR2) and CpGs analyzed by bisulfite pyrosequencing. TSS: Transcription start site. Numbers indicate distances in base pairs from TSS.

(**c**) Line plots of the percentage of methylated CpGs (mCpG). Red line: the BFP positive cells (BFPp). Light blue line: BFP negative cells (BFPn). Note that %mCpG in control cells transfected with pUC19 has been re-plotted as a reference (black line). BFPn cells include cells expressing

very low level of dCas9 methyltransferase. Each data point represents mean ± SD (n = 2-4). Asterisk (*) indicates statistical significance ($p < 0.05$) compared to the control after Bonferroni correction.

(**d**) Schematic illustration of the human *TGFBR3* promoter locus, *TGFBR3* gRNA binding sites (red arrows), potential off-target binding sites (black horizontal arrows) of the scrambled gRNA, and CpG sites.

(**e**) Line plots of % mCpG at the *TGFBR3* promoter in cells expressing dCas9 methyltransferase with (red line) or without (green line) *TGFBR3* gRNAs, or with the scrambled gRNAs (gray line). Note that %mCpG in control cells transfected with pUC19 has been re-plotted as a reference (black line). Each data point represents mean ± SD (n = 2-5).

(**f-g**) Bar chart of average methylation levels for TGFBR3-MR1 (**f**) and TGFBR3-MR2 (**g**) CpG sites. Values represent mean ± SD (n = 3). Asterisk (*) represents P value < 0.05 compared to pUC19 (ANOVA).

a

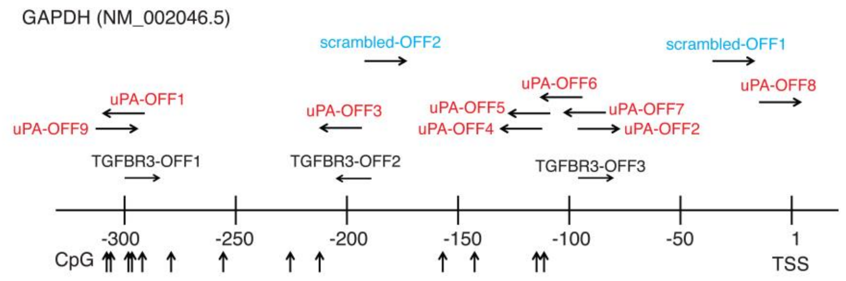

sgRNA	potential OFF sites	strand	guiding site	Sequence (5'-)	Gene	mismatches
uPA_T1	uPA-OFF1	-	chr12:6643261-6643283	GCACCGCTCGCGGCTGCACGCGG	GAPDH	9
uPA_T1	uPA-OFF2	+	chr12:6643477-6643499	GCGTGCCCAGTTGAACCAGGCGG	GAPDH	9
uPA_T2	uPA-OFF3	-	chr12:6643356-6643378	GACACTAGGGAGTCAAGGACGGG	GAPDH	10
uPA_T2	uPA-OFF4	-	chr12:6643442-6643464	GGCCCCGACGTAGGGGGGAAGGG	GAPDH	9
uPA_T2	uPA-OFF5	-	chr12:6643443-6643465	GGGCCCCGACGTAGGGGGGAAGG	GAPDH	10
uPA_T3	uPA-OFF6	-	chr12:6643451-6643473	GAGCGTGTGGGCCCCGACGTAGG	GAPDH	10
uPA_T3	uPA-OFF7	-	chr12:6643471-6643493	GGTTCAACTGGGCACGCACCGAG	GAPDH	10
uPA_T4	uPA-OFF8	+	chr12:6643556-6643578	GCGCACGTAGCTCAGGCCTCAAG	GAPDH	10
uPA_T5	uPA-OFF9	+	chr12:6643258-6643280	AGGCCGCGTGCAGCCGCGAGCGG	GAPDH	10
Scrambled_T1	Scrambled-OFF1	+	chr12:6643534-6643556	CCGGCTACTAGCGGTTTTACGGG	GAPDH	10
Scrambled_T2	Scrambled-OFF2	+	chr12:6643381-6643403	GCTGCCCACAGTCCAGTCCTGGG	GAPDH	10
TGFBR3_T1	TGFBR3-OFF1	+	chr12:6643271-6643293	CCGCGAGCGGTGCTGGGCTCCGG	GAPDH	10
TGFBR3_T2	TGFBR3-OFF2	-	chr12:6643367-6643389	GTGGGCAGCAGGACACTAGGGAG	GAPDH	9
TGFBR3_T3	TGFBR3-OFF3	+	chr12:6643477-6643499	GCGTGCCCAGTTGAACCAGGCGG	GAPDH	9

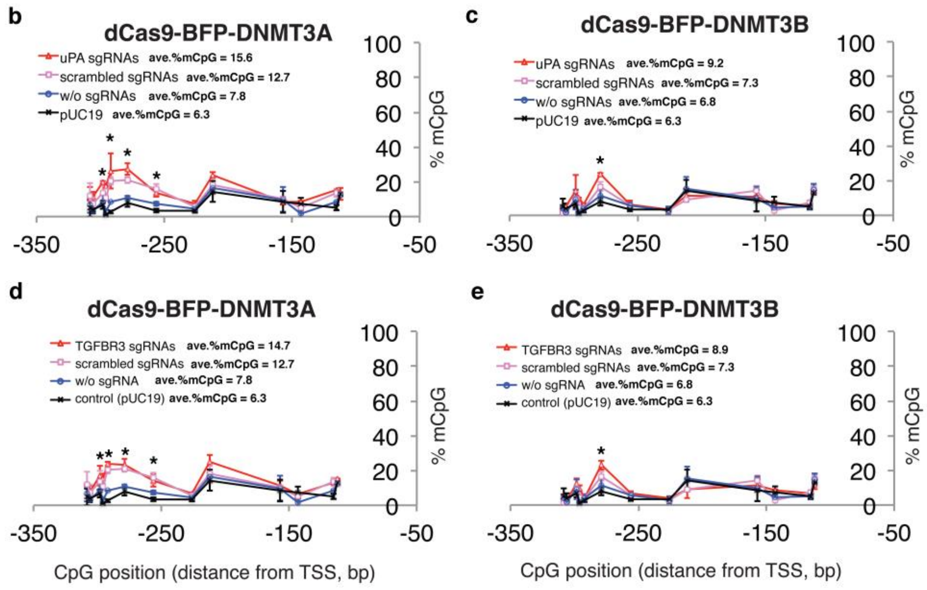

Fig. 2 Off-target methylation of *GAPDH* promoter by dCas9 methyltransferases and gRNAs

(**a**) Schematic illustration of the *GAPDH* promoter. Potential off target sites and CpGs analyzed by bisulfite pyrosequencing are indicated. Sequences of potential off-target binding sites by *uPA*, TGFBR3 and scrambled gRNAs with maximum 10 mismatches are listed.

(**b-d**) Line plots of *GAPDH* promoter methylation in FACS-sorted HEK293T cells 48 hours after transfection with dCas9 methyltransferases and gRNAs. The methylation profiles from the pUC19-transfected samples were re-plotted as reference. Each data point in the graph represents the mean ± SD (n = 2 independent transfections). Average methylation levels for all CpGs analyzed are presented next to line legends. Asterisks (*) represent P value < 0.05 compared to pUC19 (ANOVA).

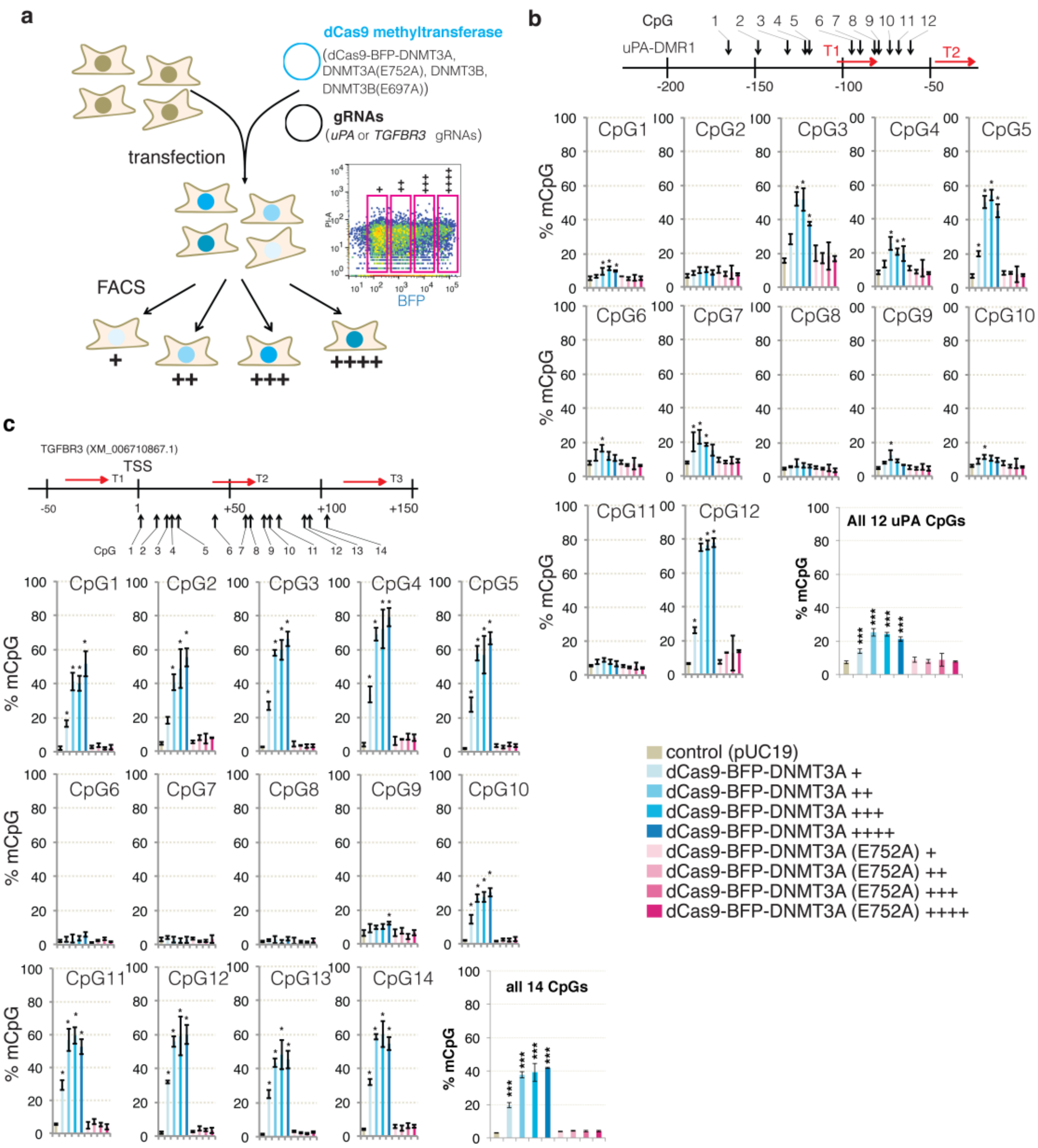

Fig. 3 On-target methylation by dCas9 methyltransferases

(**a**) Schematic illustration of the experiment. dCas9 methyltransferase-expressing cells were enriched by FACS 48 hours after transfection and sorted according to the BPF signal: +, ++, +++, ++++. Right: Representative FACS plot and gating.

(**b-c**) Bar charts indicating % mCpG for individual CpG and average values of all CpG sites in the *uPA* (**b**) and *TGFBR3* (**c**) target regions. The schematic illustrations above the bar graphs show gRNA binding sites and CpG sites analyzed. Value represents mean ± SD (n = 3). Asterisk (*) indicates statistical significance ($p < 0.05$, ANOVA) compared to the control after Bonferroni correction. Figure legend for bar graphs in (**b**) and (**c**) is presented at bottom-right.

927

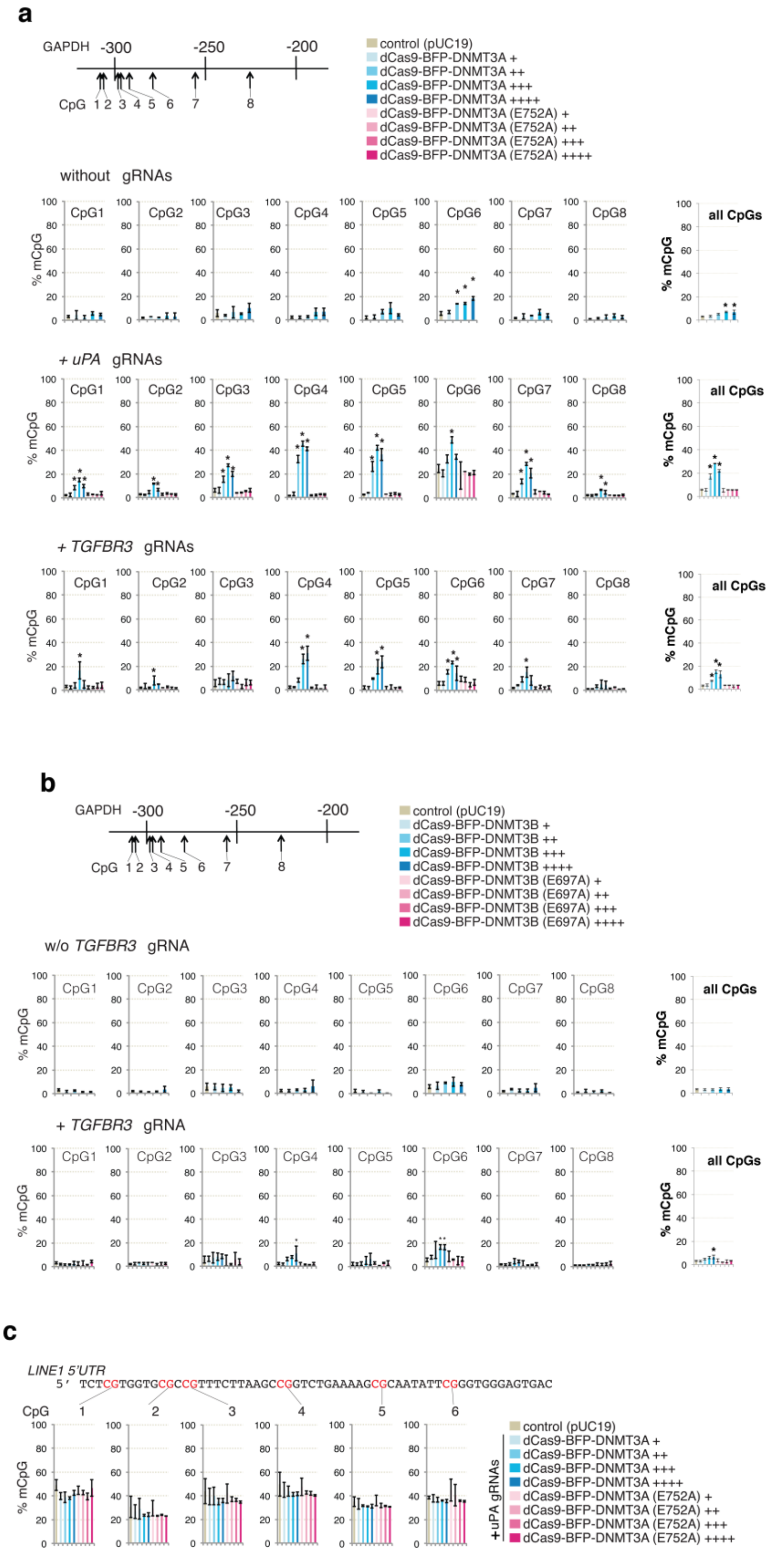
a
GAPDH -300 -250 -200
CpG 1 2 3 4 5 6 7 8
control (pUC19)
dCas9-BFP-DNMT3A +
dCas9-BFP-DNMT3A ++
dCas9-BFP-DNMT3A +++
dCas9-BFP-DNMT3A ++++
dCas9-BFP-DNMT3A (E752A) +
dCas9-BFP-DNMT3A (E752A) ++
dCas9-BFP-DNMT3A (E752A) +++
dCas9-BFP-DNMT3A (E752A) ++++
without gRNAs
+ uPA gRNAs
+ TGFBR3 gRNAs
CpG1 CpG2 CpG3 CpG4 CpG5 CpG6 CpG7 CpG8
all CpGs
% mCpG
b
GAPDH -300 -250 -200
CpG 1 2 3 4 5 6 7 8
control (pUC19)
dCas9-BFP-DNMT3B +
dCas9-BFP-DNMT3B ++
dCas9-BFP-DNMT3B +++
dCas9-BFP-DNMT3B ++++
dCas9-BFP-DNMT3B (E697A) +
dCas9-BFP-DNMT3B (E697A) ++
dCas9-BFP-DNMT3B (E697A) +++
dCas9-BFP-DNMT3B (E697A) ++++
w/o TGFBR3 gRNA
+ TGFBR3 gRNA
c
LINE1 5'UTR
5' TCTCGTGGTGCGCCGTTTCTTAAGCCGGTCTGAAAAGCGCAATATTCGGTGGGAGTGAC
CpG 1 2 3 4 5 6
+uPA gRNAs
control (pUC19)
dCas9-BFP-DNMT3A +
dCas9-BFP-DNMT3A ++
dCas9-BFP-DNMT3A +++
dCas9-BFP-DNMT3A ++++
dCas9-BFP-DNMT3A (E752A) +
dCas9-BFP-DNMT3A (E752A) ++
dCas9-BFP-DNMT3A (E752A) +++
dCas9-BFP-DNMT3A (E752A) ++++

Fig. 4 Off-target methylation by dCas9 methyltransferases

(a) Bar charts indicating % mCpG at individual CpGs and total % mCpG (8 CpG sites) for the *GAPDH* promoter in cells expressing different levels (BFP signal: +, ++, +++, ++++) of dCas9-BFP-DNMT3A or dCas9-BFP-DNMT3A(E752A) alone or together with either *uPA* or *TGFBR3* gRNAs.

(b) Bar charts indicating % mCpG in the *GAPDH* promote in cells expressing different levels (BFP signal: +, ++, +++, ++++) of dCas9-BFP-DNMT3B or dCas9-BFP-DNMT3B(E697A) alone or with *TGFBR3* gRNAs.

(**c**) *LINE1* 5'UTR methylation in cells expressing *uPA* gRNAs with different levels of either dCas9-BFP-DNMT3A or dCas9-BFP-DNMT3A(E752A). Cells transfected with pUC19 were used as controls. Values represent mean ± SD (n = 3). Asterisks (*) represent P value < 0.05 (ANOVA) compared to pUC19.

Fig. 5

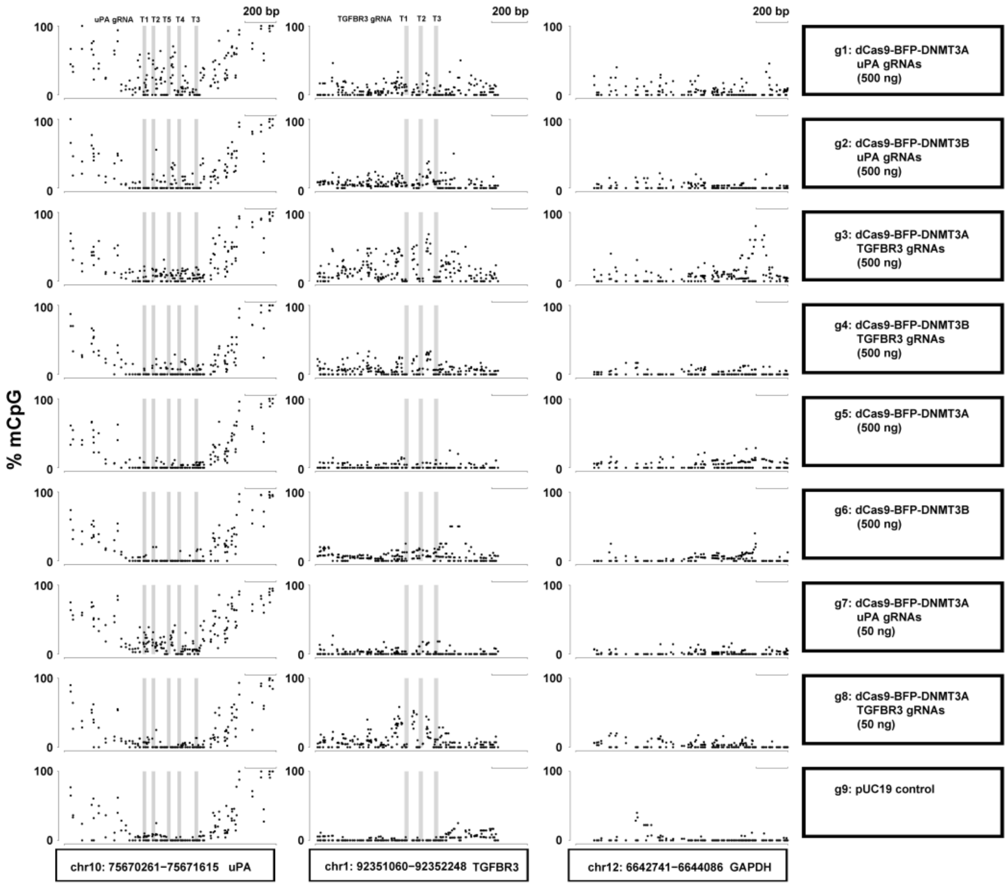

Fig. 5 De novo methylation of *uPA, TGFBR3 and GAPDH* promoters by dCas9 methyltransferase measured with WGBS. Dot plots of % mCpG for individual CpG sites in the

uPA, TGFBR3 and GAPDH promoter regions. Each dot represents one CpG site. Right panel indicates the transfected plasmids. mCpG levels were quantified by WGBS. Scale bar, 200 bp.

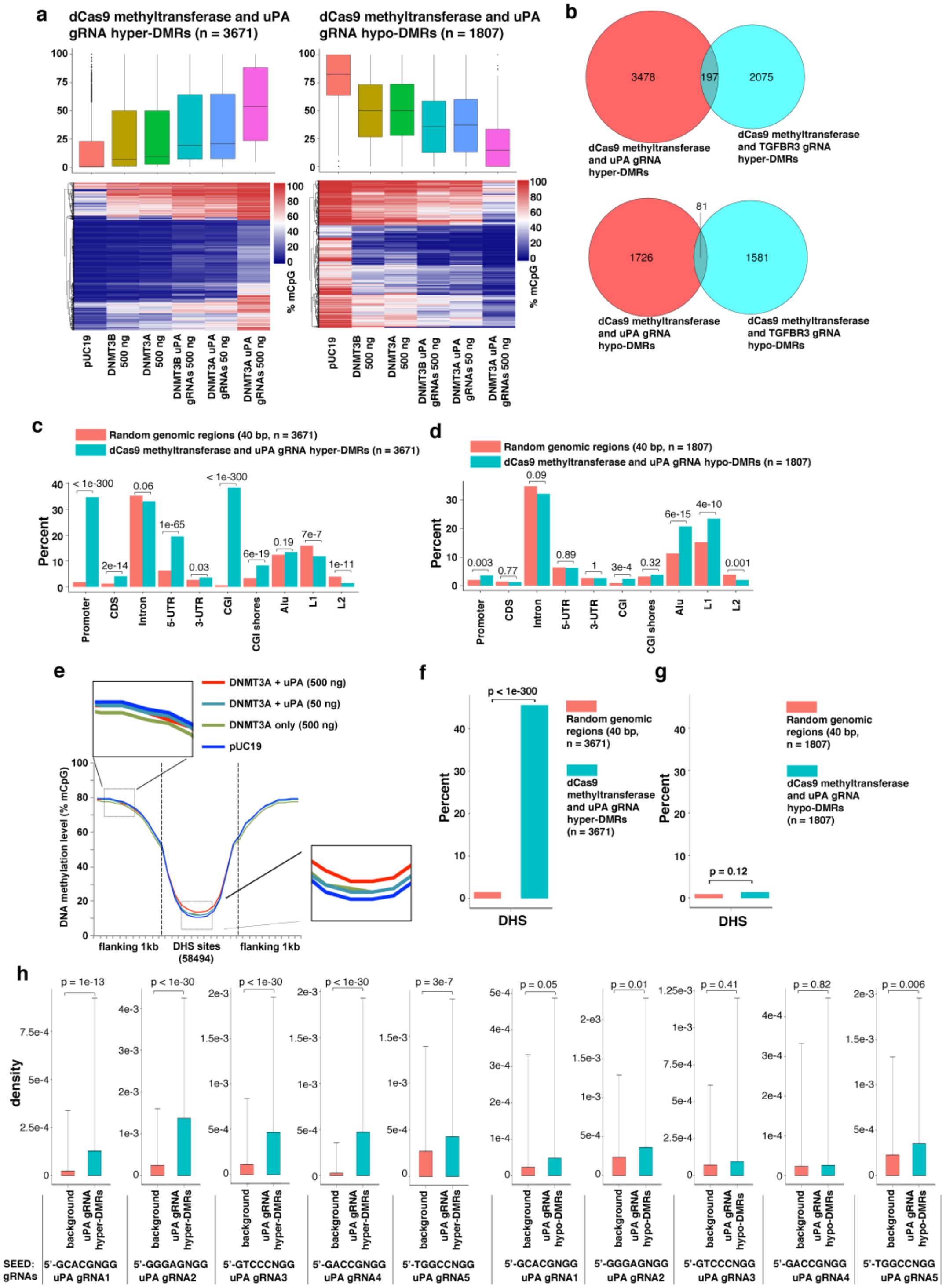

Fig. 6 Genomic characteristics of off-target DMRs caused by dCas9 methyltransferases and *uPA* gRNAs

(**a**) Box plot (top) and heatmap clustering (bottom) of the hypermethylated (left) and hypomethylated (right) DMRs resulting from dCas9 methyltransferases and *uPA* gRNAs.

(**b**) Venn diagram presentation of hypermethylated (top) or hypomethylated (bottom) DMRs caused by dCas9 methyltransferases and *uPA* gRNAs compared to *TGFBR3* gRNAs.

(**c-d**) Bar chart illustrating the percentage of the identified uPA hypermethylated (**c**) or hypomethylated (**d**) DMRs that fall into the different types of genomic regions indicated. Background represents a random sample of the same number of similar sized genomic windows that fall into the categories indicated. Values above bars are P values between background and uPA-DMRs (Fisher's exact test).

(**e**) Metaplot of average CpG methylation levels in 58,494 DNase I hypersensitive sites (DHS) and 1 kb upstream and downstream flanking regions.

(**f-g**) Bar chart of % uPA hypermethylated (**f**) or hypomethylated (**g**) DMRs falling into DHS core regions.

(**h**) Density of 5nt-SEED-NGG for uPA gRNAs (T1 to T5) in background genomic windows and uPA DMRs + flanking 100 bp. Values represent median density with one standard deviation. P values (t-test) are given above the bar charts.

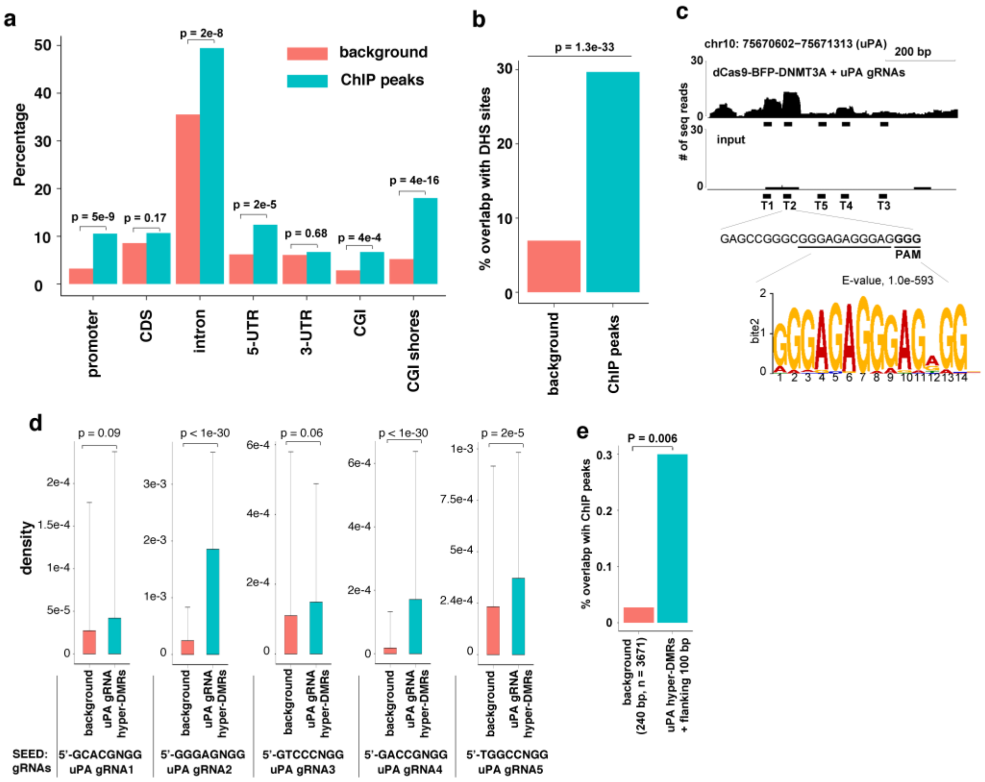

Fig. 7 Correlation between dCas9-BFP-DNMT3A off-target binding and off-target methylation

(**a**) Bar chart illustrating the percentage of ChIP peaks from cells expressing dCas9-BFP-DNMT3A and uPA gRNAs or background control regions (random sampling of the same number of similar sized genomic windows as the ChIP peaks) falling into the different types of genomic regions indicated. P-values between background and ChIP peaks indicated above bars, Fisher's exact test.

(**b**) Bar chart of % ChIP-peaks falling into DHS core regions.

(**c**) Representative plot of ChIP-seq reads in the *uPA* promoter, uPA gRNA T2 sequences, and the top motif identified by MEME-ChIP.

(**d**) Density of 5nt-SEED-NGG for uPA gRNAs (T1 to T5) ChIP peaks. Background is a random sample of the same number of similar sized genomic windows as ChIP peaks. Values represent median density with one standard deviation. P values are given for the indicated comparisons (t-test).

(**e**) Bar plot of % ChIP peaks overlapping with hypermethylated DMRs caused by dCas9 methyltransferase and *uPA* gRNAs. Background is a random sample of the same number of similar sized genomic windows as DMRs.

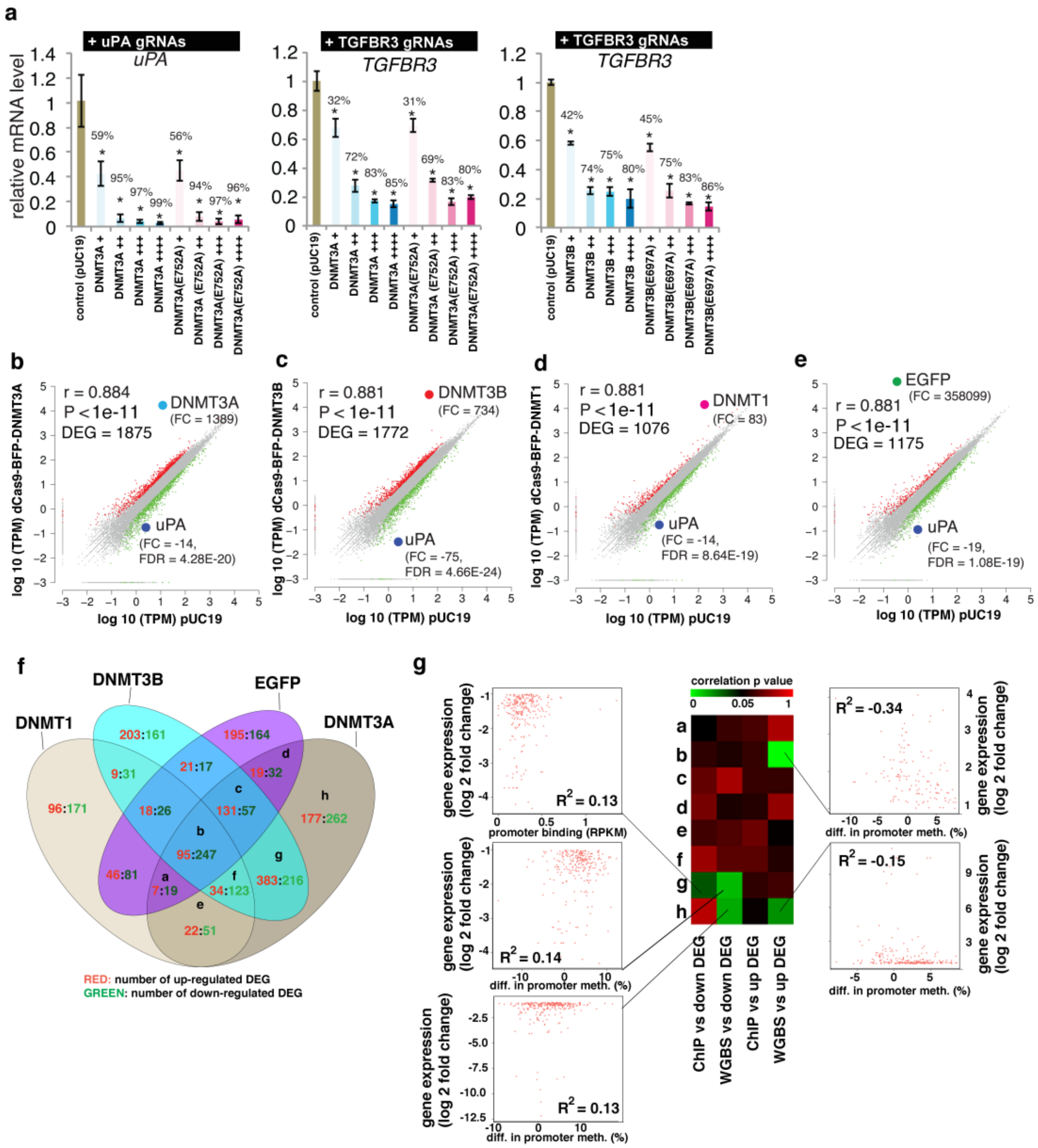

Fig. 8 Effect of dCas9 methyltransferases on gene expression

(**a**) Relative gene expression levels of *uPA* and *TGFBR3* in cells expressing different levels of dCas9-BFP-DNMT3A, dCas9-BFP-DNMT3B, dCas9-BFP-DNMT3A(E752A), or dCas9-BFP-DNMT3B(E697A). mRNA expression was measured by qPCR and quantified as fold change compared to control cells transfected with pUC19. Bar charts depict mean change in mRNA level compared to pUC19 controls. Data represent mean ± SD (n = 3 independent transfections). Mean percentage decrease in mRNA level compared to pUC19 is presented on top of bars. Asterisks (*) represent P value < 0.05 compared to pUC19.

(**b-e**) Dot plots of log10 (transcripts per million (TPM)) for all genes expressed in the BFP positive (BFPp) cells expressing *uPA* gRNAs (T1-T5) and dCas9-BFP-DNMT3A (**b**), dCas9-BFP-DNMT3B (**c**), dCas9-BFP-DNMT1 (**d**), or dCas9-BFP-EGFP (**e**) plotted against log10 (TPM) in a pUC19 control group. Differentially expressed genes (DEG) are marked in red (up-regulated) and green (down-regulated) (fold change >= 2, FDR < 0.001). Fold changes compared to pUC19 and FDR p-values for DNMT1, *DNMT3A, DNMT3B, EGFP*, and *uPA* are shown.

(**f**) Venn diagram representation of cross-comparison of DEGs.

(**g**) Integrative analysis of gene expression change, promoter methylation and promoter binding caused by dCas9-BFP-DNMT3A and *uPA* gRNAs for the different clusters of DEGs. Heatmap represents linear regression p values. Dot plots were given for significant correlations ($p < 0.05$).

Supplementary Figure Legends

Supplementary Fig. S1 Validation of dCas9 methyltransferase expression and *uPA* promoter methylation

(**a**) Schematic overview of the human DNA methyltransferases (DNMT1, DNMT3A and DNMT3B) with the N-terminal regulatory region, a C-terminal catalytic domain (CD), and the cytosine C5-DNA methyltransferase motifs highlighted. The first amino acid (a.a) residue of the C-terminal catalytic domain, which was fused to the dCas9, is indicated by an arrow.

(**b**) Representative FACS sorting and Re-analysis of HEK293T cells 48 hours after transfection. Gating for BFP positive (BFPp) and negative (BFPn) cells are indicated.

(**c**) Laser scanning microscopy of **dCas9 methyltransferase** expression in HEK293T cells, 48 hours after transfection. The BFP signal from the dCas9-BFP-DNMT1 transfected cells was enhanced since the BFP signal from the dCas9-BFP-DNMT1 fusion was initially weaker compared to that from the other three fusion proteins. Scale bar: 20 µm.

(**d**) Validation of RNA-guided *uPA* methylation (uPA-MR1) by dCas9-BFP-DNMT3A using bisulfite Sanger sequencing.

Supplementary Fig. S2 Validation of dCas9 methyltransferase-mediated *TGFBR3* methylation in HEK293T cells by bisulfite Sanger sequencing

TGFBR3 methylation by dCas9 methyltransferase and gRNAs was validated by bisulfite Sanger sequencing. CpG methylation status is indicated according to the absolute nucleotide position and color-coded as red, methylated; blue, unmethylated; or white, unknown methylation state based on the sequencing signal.

Supplementary Fig. S3 Validation of *de novo* methylation of *uPA* by dCas9 methyltransferase and *uPA* gRNAs

Line plots of uPA-MR2 methylation in cells transfected with pUC19 (control), dCas9-BFP-DNMT3A or dCas9-BFP-DNMT3B only, and dCas9-BFP-DNMT3A or dCas9-BFP-DNMT3B together with either *uPA* gRNAs or scrambled gRNAs.

Supplementary Fig. S4 Effect of dCas9 methyltransferases on two potential off-target sites (*SH2D3C* and *FAM221A*).

(**a-b**) Schematic illustration of the *SH2D3C* (**b**) and *FAM221A* (**c**) off-target loci, with off-target sites indicated by red arrows. Sequences of *uPA* gRNA (T2), *SH2D3C*, and *FAM221A* off-target sites are given above, with the PAM (red letters) and mismatches (green letters) indicated. CpGs analyzed are indicated by black arrows; numbers indicate distances (in bp) from the transcription start site (TSS) of the gene (SH2D3C, NM_001252334.1) or (FAM221A, XM_011515369.1). Y-

axis represents % mCpG level for each CpG site and X-axis represents distance (in bp) from TSS. The CpG methylation level from the control samples (pUC19 transfection) was re-plotted as a reference. Each data point in the graph represents the mean percentage of CpGs methylated ± SD (n = 2, independent transfections).

Supplementary Fig. S5 Effects of DNMT3B catalytic activity and expression level on *de novo TGFBR3* methylation

Bar charts of % mCpG level for individual CpG sites of the *TGFBR3* targeted regions in dCas9 methyltransferase-expressing cells. Cells were enriched by FACS 48 hours after transfection and sorted according to the BPF signal: +, ++, +++, ++++. The schematic illustrations above the bar charts show gRNA binding sites and CpG sites analyzed. Asterisk (*) indicates statistical significance ($p < 0.05$, ANOVA) compared to the pUC19 control group after Bonferroni correction. Percentage values represent % decrease of *TGFBR3* expression compared to pUC19.

Supplementary Fig. S6 WGBS analysis of cells expressing dCas9 methyltransferase and gRNAs

(**a**) Summary of WGBS including clean data, clean reads, clean rate, mapped reads, uniquely mapped reads and rate, and bisulfite conversion rate, for each experimental group and control (pUC19).

(**b**) Average percentage of methylated cytosine (% mC) for whole-genome CpG sites, CHG sites, and CHH sites. "H" represents A, C, and T.

(**c-d**) Average mCpG level (percentage) stratified according to individual chromosome or whole genome for all samples measured by WGBS.

Supplementary Fig. S7 Differentially methylated regions (DMRs) identified by DSS-single method. DMRs were categorized as hypermethylated or hypomethylated compared to control sample (pUC19 transfection).

Supplementary Fig. S8 Histogram charts of the distribution of DMR length (bp), and number of CpGs per DMR. DMRs included in this figure are those remaining after the stringent filtering step (see methods). Mean DMRs length (in bp) and mean number of CpG per DMR were given for each chart.

Supplementary Fig. S9 Genomic characteristics of off-target DMRs caused by dCas9 methyltransferases and *TGFBR3* gRNAs

(**a**) Box plot (top) and heatmap clustering (bottom) of the hypermethylated (left) and hypomethylated (right) DMRs caused by dCas9 methyltransferases and *TGFBR3* gRNAs.

(**b-c**) Bar chart illustrating the percentage of the identified *TGFBR3* hypermethylated (**c**) or hypomethylated (**d**) DMRs that fall into the different types of genomic regions indicated. Background represents of a random sample of the same number of similar sized genomic windows that fall into the categories indicated. Values above bars are P values between background and *TGFBR3* DMRs, Fisher's exact test.

(**d**) Metaplot of average CpG methylation levels in 58,494 DNase I hypersensitive sites (DHS) and 1 kb upstream and downstream flanking regions.

(**e-f**) Bar chart of % *TGFBR3* hypermethylated (**f**) or hypomethylated (**g**) DMRs falling into DHS core regions.

(**h**) Density of 5nt-SEED-NGG for TGFBR3 gRNAs (T1 to T3) in background genomic windows and *TGFBR3* DMRs + flanking 100 bp. Values represent median density with one standard deviation. P values (t-test) are given above the bar charts.

Supplementary Fig. S10 Average methylation levels of seven genomic regions in all annotated genes (hg19). (**a-d**) Each line indicates the genome-wide average methylation levels across seven genomic regions: upstream 2kb of the transcription start site, first exon, first intron, internal exons, internal introns, last exon, and downstream 2kb of the last exon.

Supplementary Fig. S11 The average methylation level in ChIP-peaks and flanking regions. Bar chat presents the average methylation level of all dCas9-BFP-DNMT3A and uPA gRNA off-target binding sites (n = 7754) found by ChIPseq, as well as the 2kb upstream and downstream region.

Supplementary Fig. S12 Effect of dCas9 methyltransferases and uPA gRNAs on cell growth. Cell growth was determined by counting the number of cell clones derived from 1,000 BFP positive cells after transfection. Values represent mean and one standard deviation from 6 experimental repeats. Asterisks represent a p value < 0.05 (ANOVA) compared to pUC19 transfection control.

Supplementary Fig. S13 Effects of dCas9 methyltransferases on mCherry expression in fluorescence reporter cell lines

(**a**) Schematic illustration of the mCherry fluorescence transgene expression cassette. The target sites of the gRNAs within the CMV promoter are indicated by red arrows (5'-3', targeting sense or antisense strands).

(**b**) Southern blot analysis of five cell clones with the transgene cassette randomly and stably integrated into the genome.

(**c**) Flow cytometry-based analysis of the percentage of BFP positive cells in the fluorescence reporter cells at 2, 5, 8 and 14 days after transient transfection with CMV gRNAs (T1-T3) and dCas9-BFP-DNMT1, dCas9-BFP-DNMT3A, dCas9-BFP-DNMT3B, or dCas9-BFP-EGFP.

(**d-h**) % mCherry fluorescence median intensity in these five clones at day 2, 5, 8, and 14 days following transient transfection with CMV gRNAs (T1-T3) and dCas9-BFP-DNMT1, dCas9-BFP-DNMT3A, dCas9-BFP-DNMT3B, or dCas9-BFP-EGFP. Control cells were transfected with pUC19. Percent inhibition of mCherry expression was calculated by normalizing the median mCherry fluorescence intensity to that from the pUC19 transfected cells at each time point. Figures are plotted using the mean % mCherry median ± SD (n = 3, independent transfections). ANOVA with Bonferroni comparison was performed for cell clone 2. “a”, “b”, “c”, and “d,” indicates a p-value < 0.05 compared to the pUC19 control for the corresponding transfection group.

Supplementary Fig. S14 Validation of hypermethylated DMRs and DHS methylation caused by dCas9-BFP-DNMT3A and *uPA* gRNAs by WGBS

(**a**) Box plot of % methylation level of hypermethylated DMRs dCas9 found in previous WGBS experiment. The WGBS in the repeat experiment was conducted as described in the methods sections, but cells were not FACS enriched and sequenced in lower depth than previous experiment.

(**b**) Metaplot of average CpG methylation levels in 58,494 DNase I hypersensitive sites (DHS) and 1 kb upstream and downstream flanking regions. P value represents Wilcoxon matched pairs signed rank test between treated and control groups.

Supplementary Table S1 List of plasmids deposited to Addgene, qPCR primers, gRNA sequences, bisulfite PCR primers, bisulfite pyrosequencing primers, and DNA regions analyzed for methylation.

Supplementary Table S2 List of hypermethylated DMRs caused by dCas9 methyltransferases and uPA gRNAs

Supplementary Table S3 List of hypomethylated DMRs caused by dCas9 methyltransferases and uPA gRNAs

Supplementary Table S4 List of hypermethylated DMRs caused by dCas9 methyltransferases and TGFBR3 gRNAs

Supplementary Table S5 List of hypomethylated DMRs caused by dCas9 methyltransferases and TGFBR3 gRNAs

Supplementary Table S6 List of binding peaks caused by dCAs9-BFP-DNMT3A and uPA gRNAs

Supplementary File 1 Extended discussion and results

Figure 1 Click here to download Figure Fig 1.tif

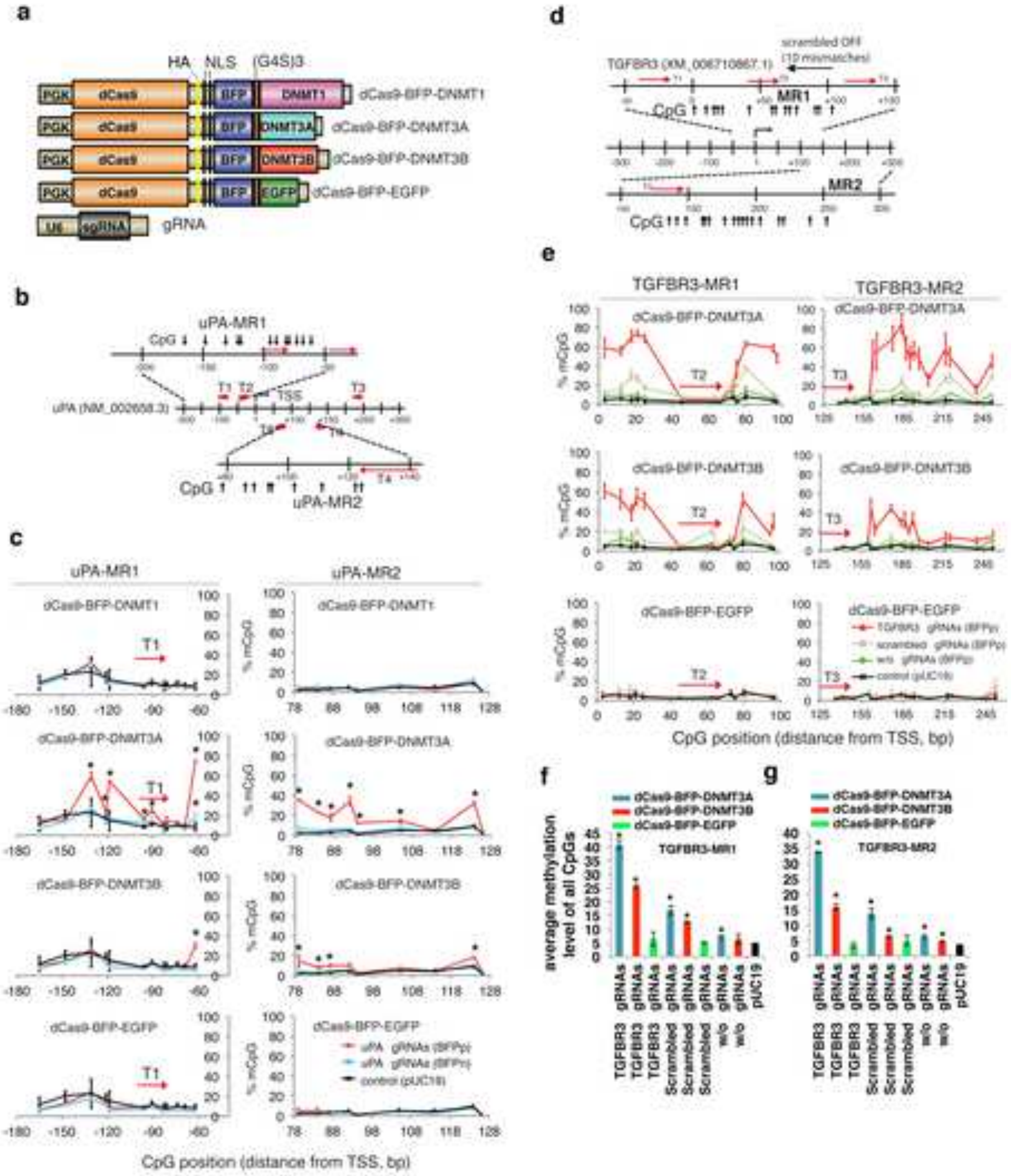

a

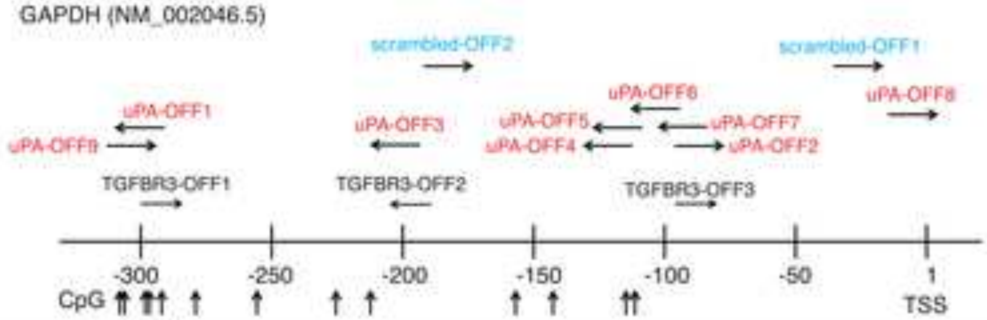

sgRNA	potential OFF sites	strand	guiding site	Sequence (5'-)	Gene	mismatches
uPA_T1	uPA-OFF1	-	chr12:6643261-6643283	GCACCGCTCGCGGCTGCACGCGG	GAPDH	9
uPA_T1	uPA-OFF2	+	chr12:6643477-6643499	GCGTGCCCAGTTGAACCAGGCGG	GAPDH	9
uPA_T2	uPA-OFF3	-	chr12:6643356-6643378	GACACTAGGGAGTCAAGGACGGG	GAPDH	10
uPA_T2	uPA-OFF4	-	chr12:6643442-6643464	GGCCCCGACGTAGGGGGGAAGGG	GAPDH	9
uPA_T2	uPA-OFF5	-	chr12:6643443-6643465	GGGCCCCGACGTAGGGGGGAAGG	GAPDH	10
uPA_T3	uPA-OFF6	-	chr12:6643451-6643473	GAGCGTGTGGGCCCCGACGTAGG	GAPDH	10
uPA_T3	uPA-OFF7	-	chr12:6643471-6643493	GGTTCAACTGGGCACGCACCGAG	GAPDH	10
uPA_T4	uPA-OFF8	+	chr12:6643556-6643578	GCGCACGTAGCTCAGGCCTCAAG	GAPDH	10
uPA_T5	uPA-OFF9	+	chr12:6643258-6643280	AGGCCGCGTGCAGCCGCGAGCGG	GAPDH	10
Scrambed_T1	Scrambled-OFF1	+	chr12:6643534-6643556	CCGGCTACTAGCGGTTTTACGGG	GAPDH	10
Scrambed_T2	Scrambled-OFF2	+	chr12:6643381-6643403	GCTGCCCACAGTCCAGTCCTGGG	GAPDH	10
TGFBR3_T1	TGFBR3-OFF1	+	chr12:6643271-6643293	CCGCGAGCGGTGCTGGGCTCCGG	GAPDH	10
TGFBR3_T2	TGFBR3-OFF2	-	chr12:6643367-6643389	GTGGGCAGCAGGACACTAGGGAG	GAPDH	9
TGFBR3_T3	TGFBR3-OFF3	+	chr12:6643477-6643499	GCGTGCCCAGTTGAACCAGGCGG	GAPDH	9

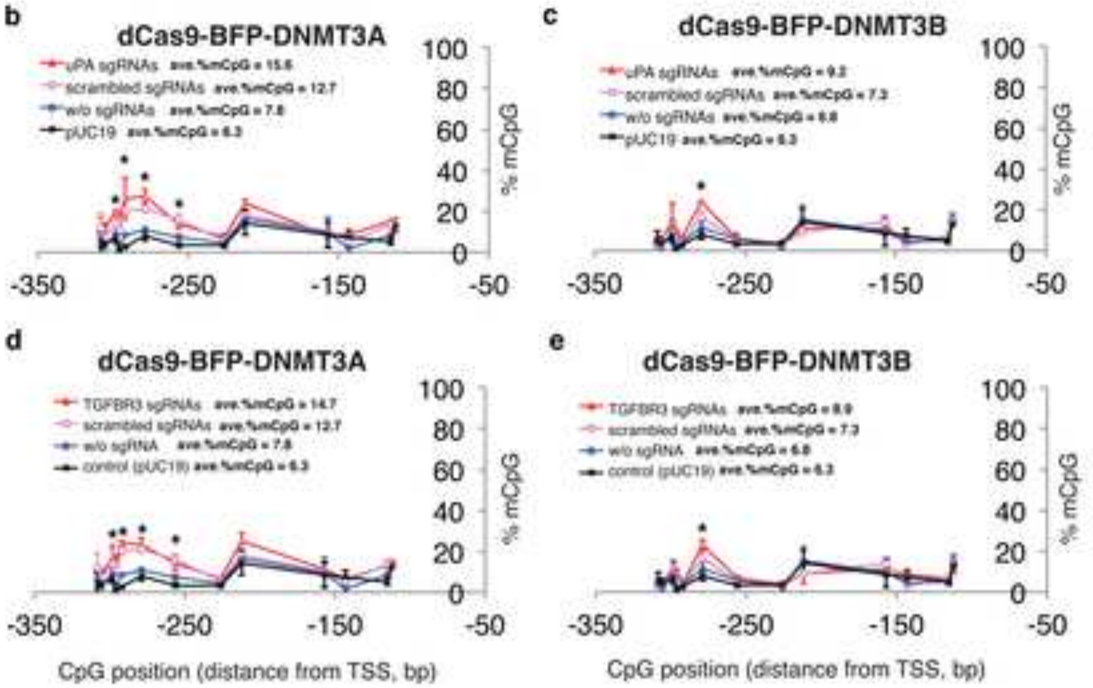

Figure 3

Click here to download Figure Fig 3.tif

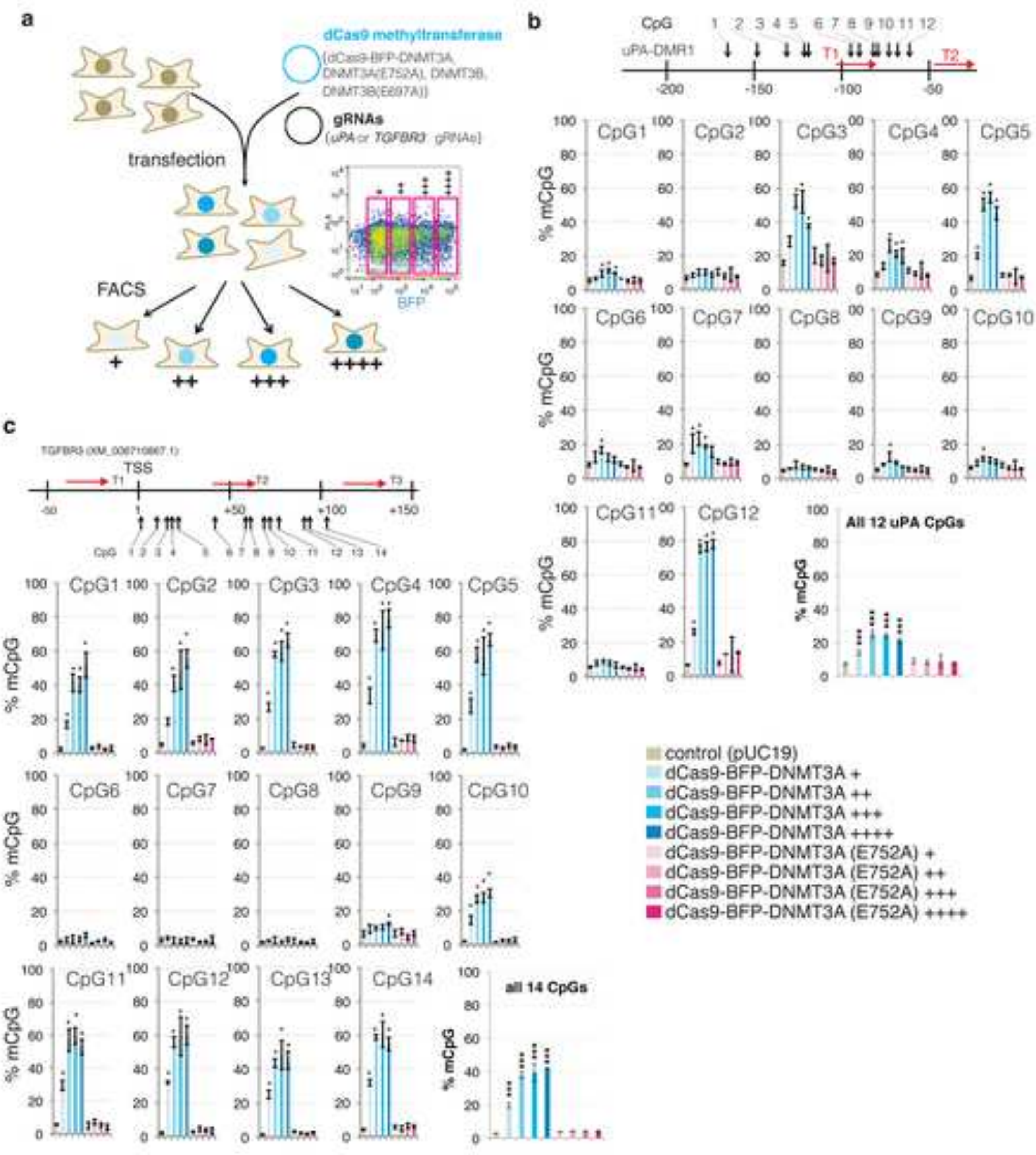

Figure 4

Click here to download Figure Fig 4.tif

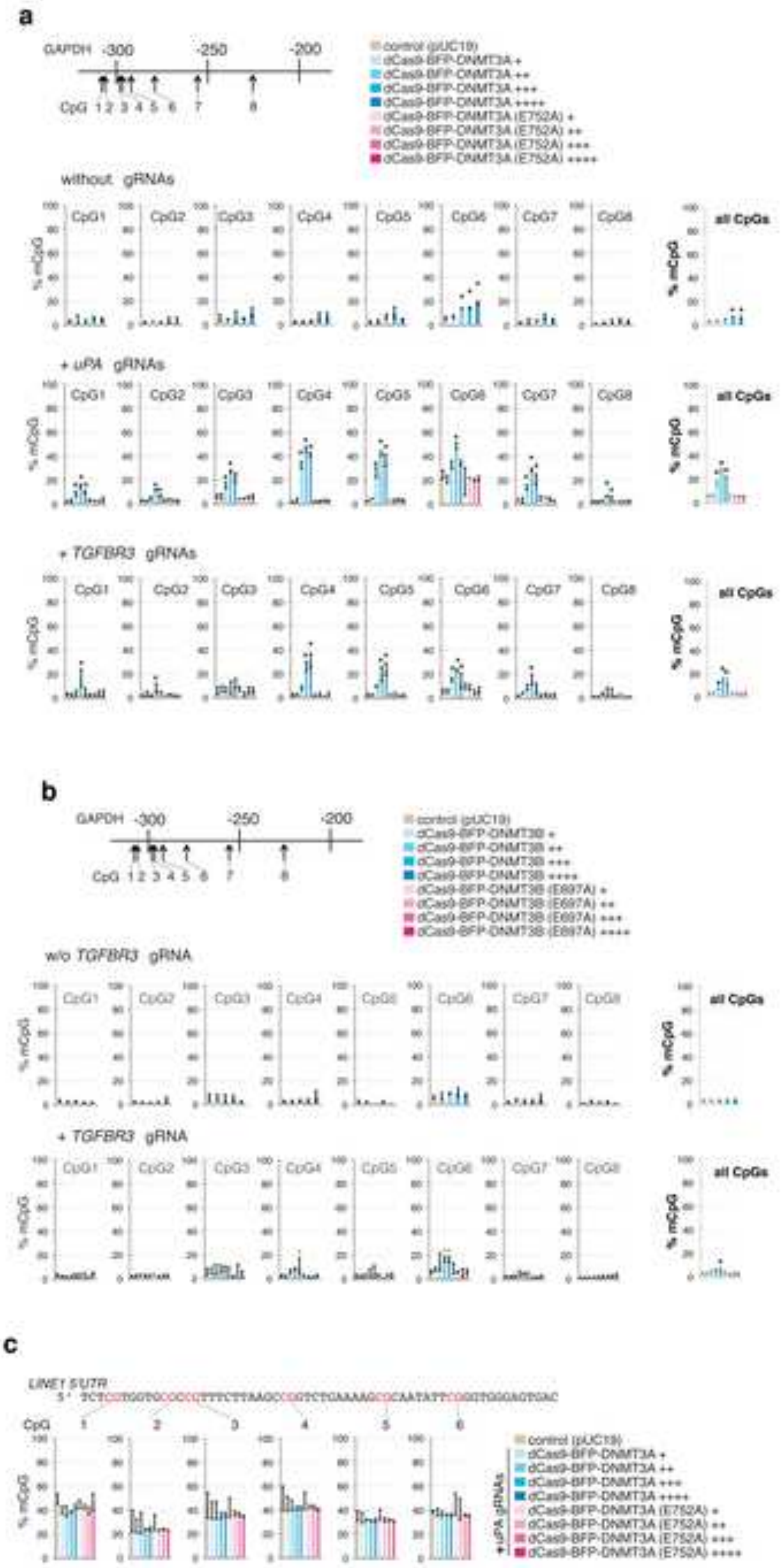

Figure 5

Click here to download Figure Fig 5.tif

Fig. 5

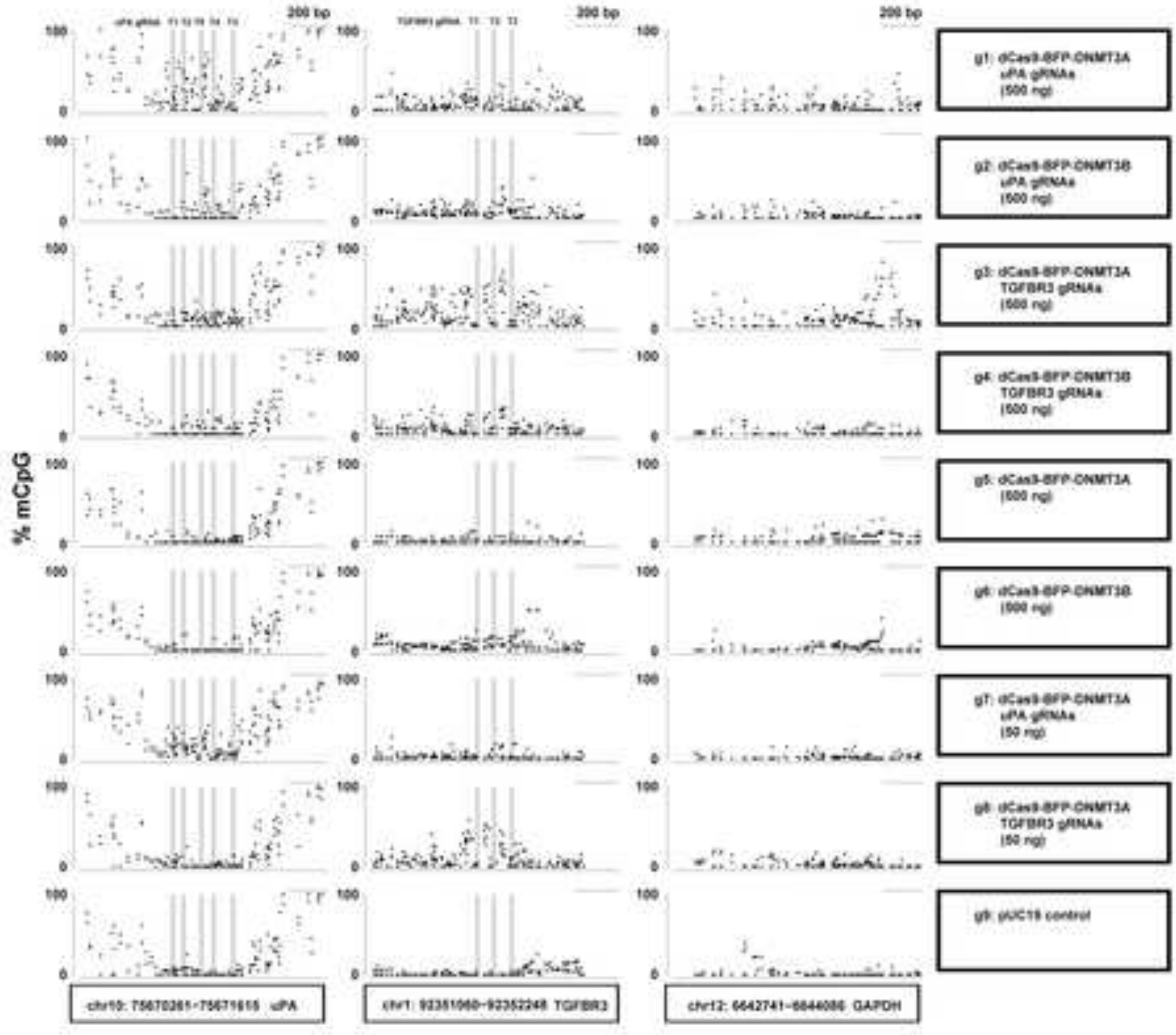

Figure 6

Click here to download Figure Fig 6.tif

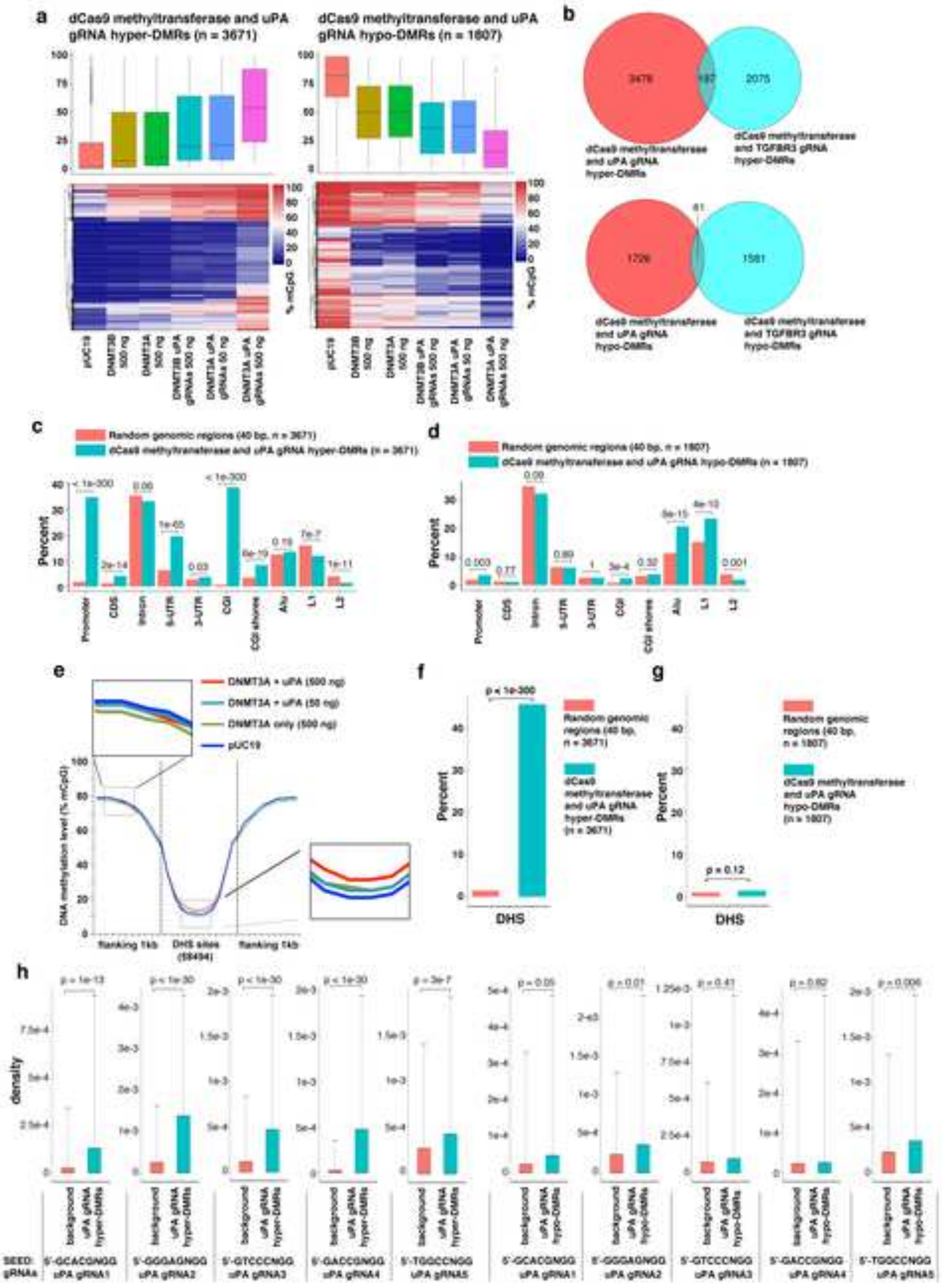

Figure 7

Click here to download Figure Fig 7.tif

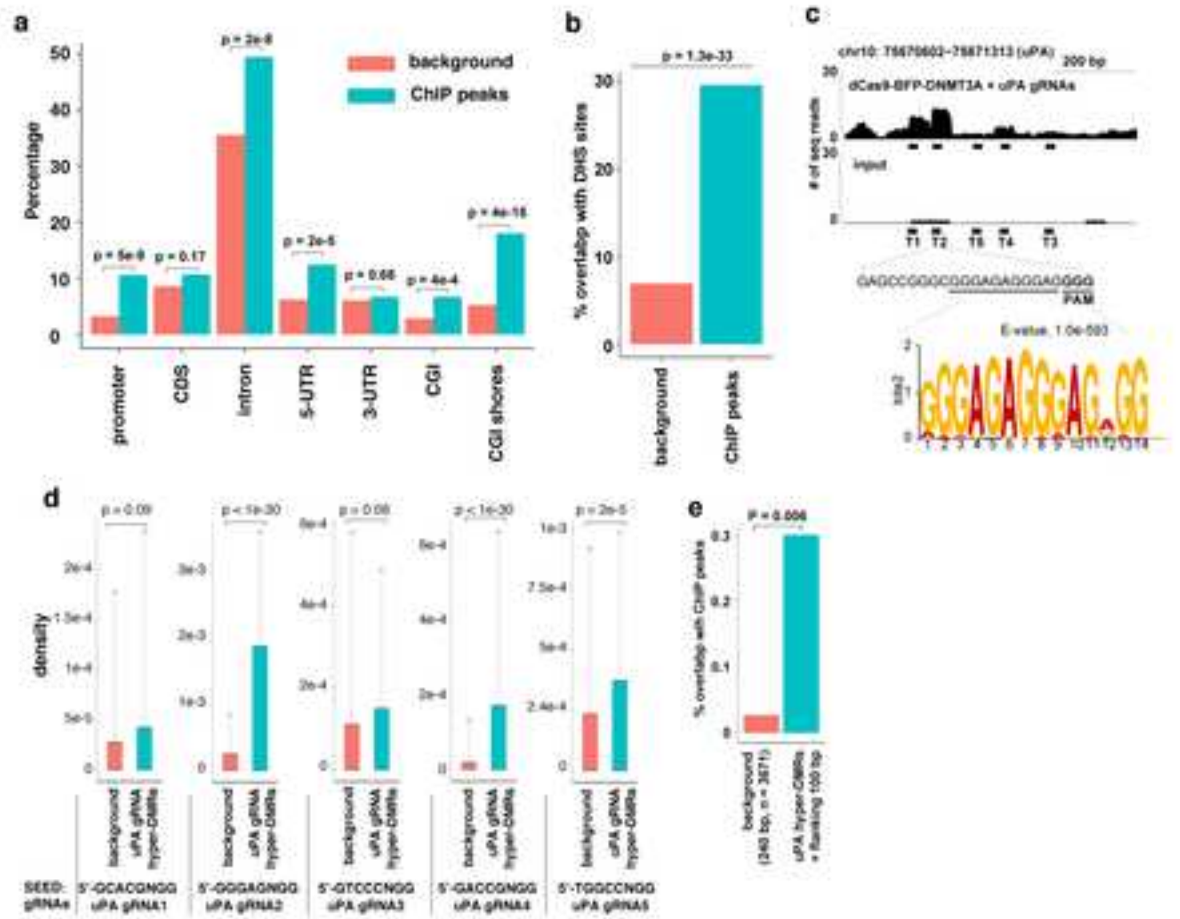

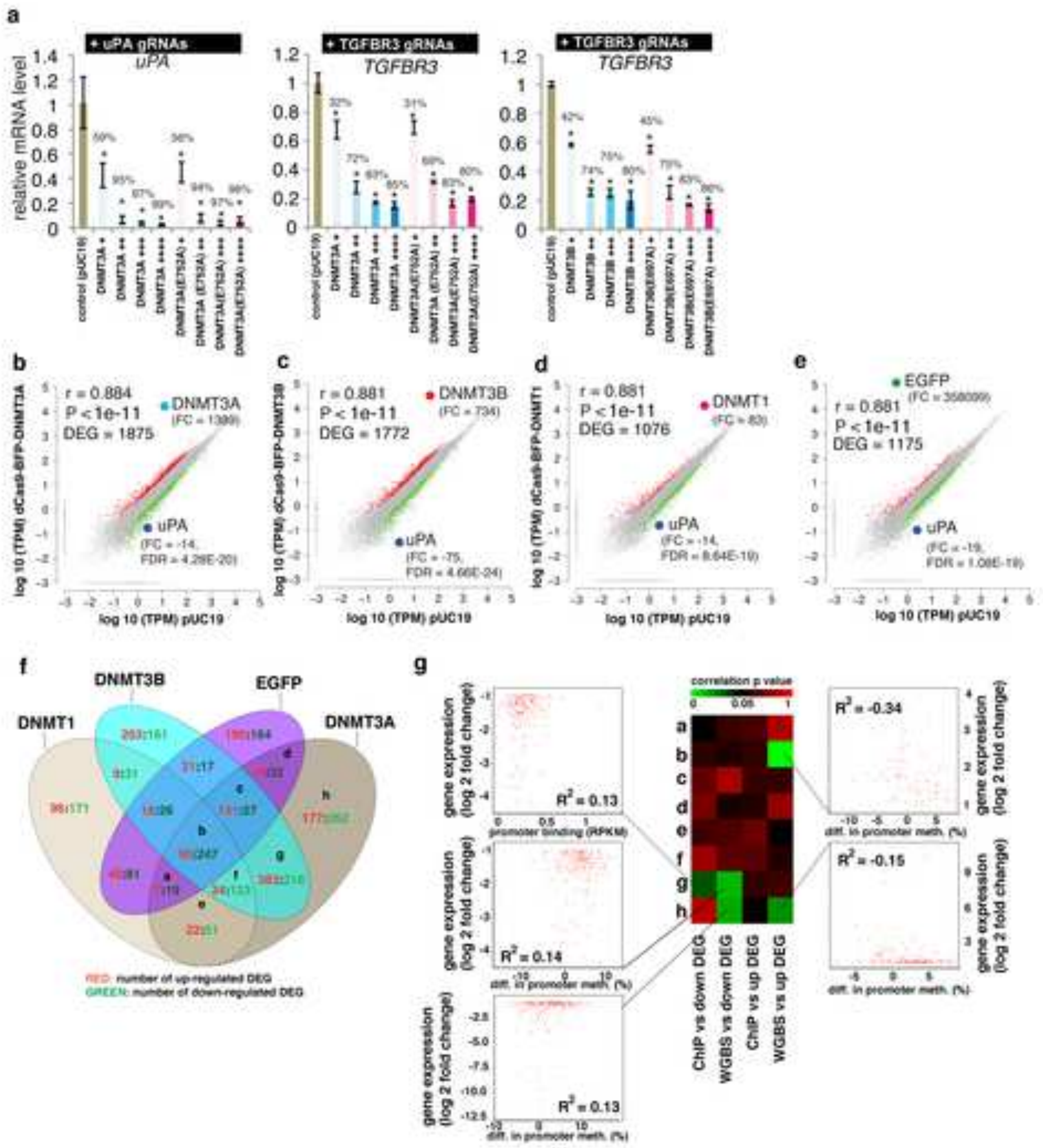
a
+ uPA gRNAs
uPA
+ TGFBR3 gRNAs
TGFBR3
relative mRNA level
b
r = 0.884
P < 1e-11
DEG = 1875
DNMT3A
c
r = 0.881
P < 1e-11
DEG = 1772
DNMT3B
d
r = 0.881
P < 1e-11
DEG = 1076
DNMT1
e
r = 0.881
P < 1e-11
DEG = 1175
EGFP
uPA
log 10 (TPM) pUC19
f
DNMT1
DNMT3B
EGFP
DNMT3A
RED: number of up-regulated DEG
GREEN: number of down-regulated DEG
g
correlation p value
gene expression (log 2 fold change)
promoter binding (RPKM)
diff. in promoter meth. (%)
ChIP vs down DEG
WGBS vs down DEG
ChIP vs up DEG
WGBS vs up DEG

Figure S1

Click here to download Figure Fig.S1.tif

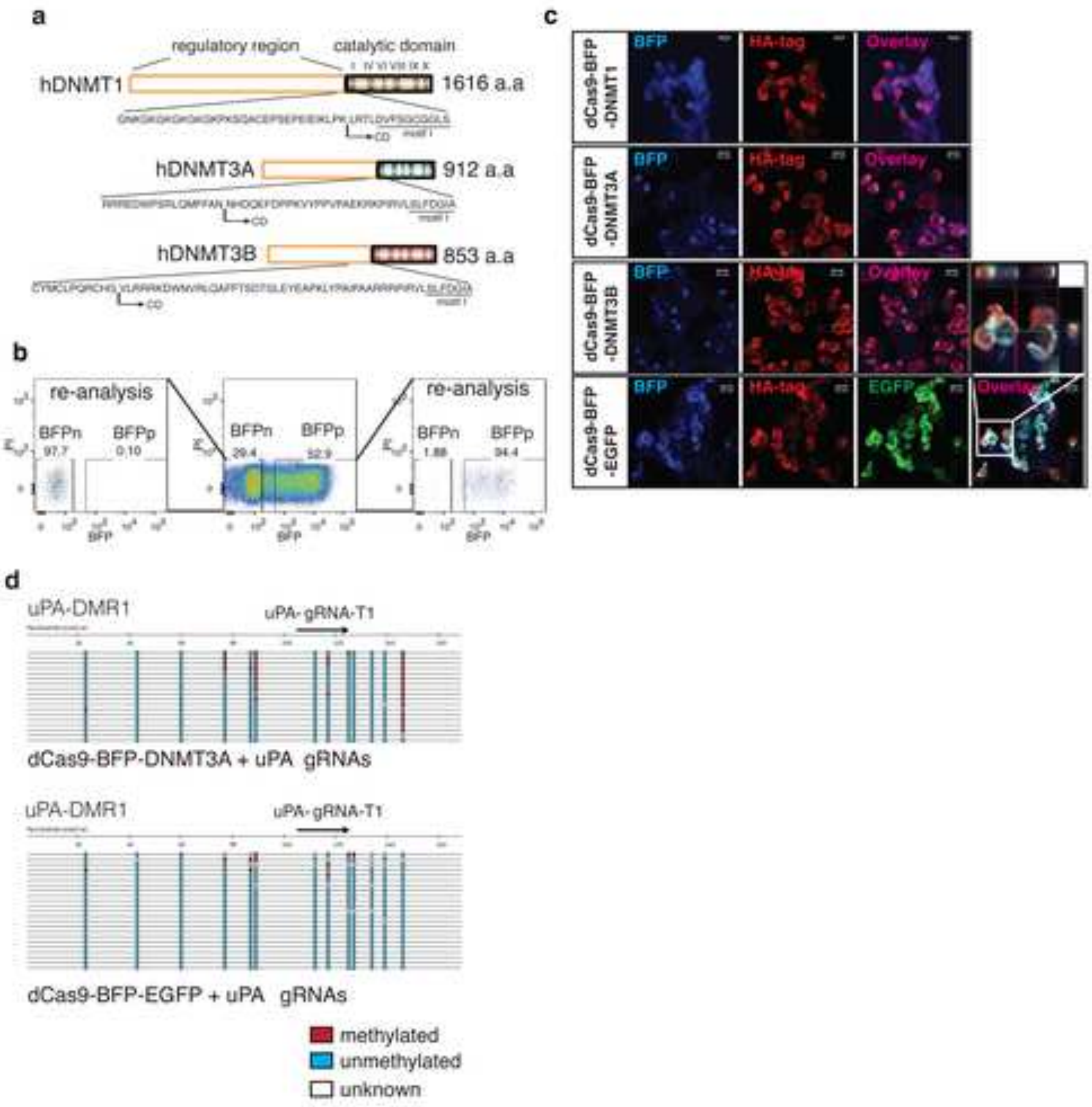

Figure S2

Click here to download Figure Fig.S2.tif

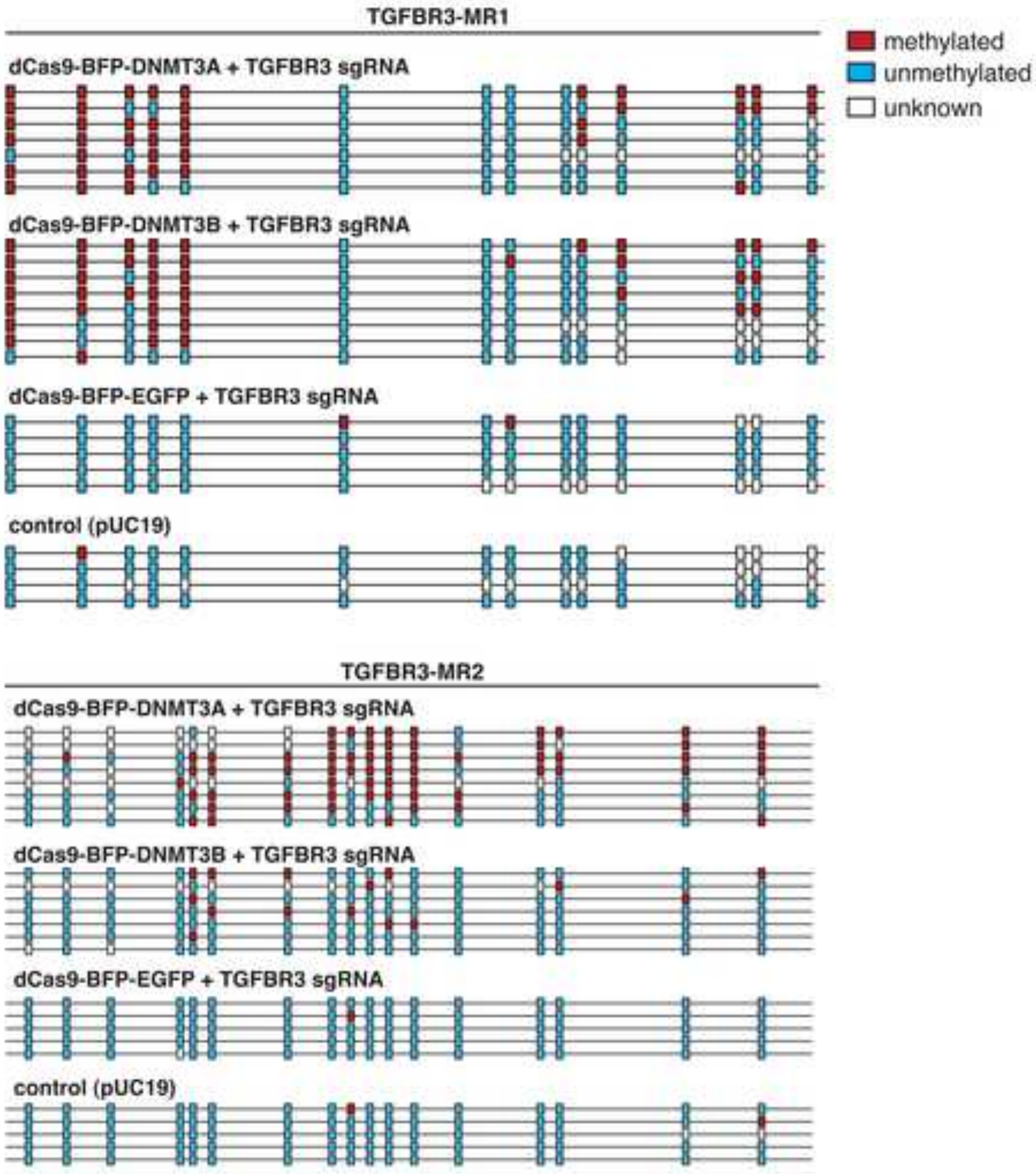

Figure S3

Click here to download Figure Fig.S3.tif

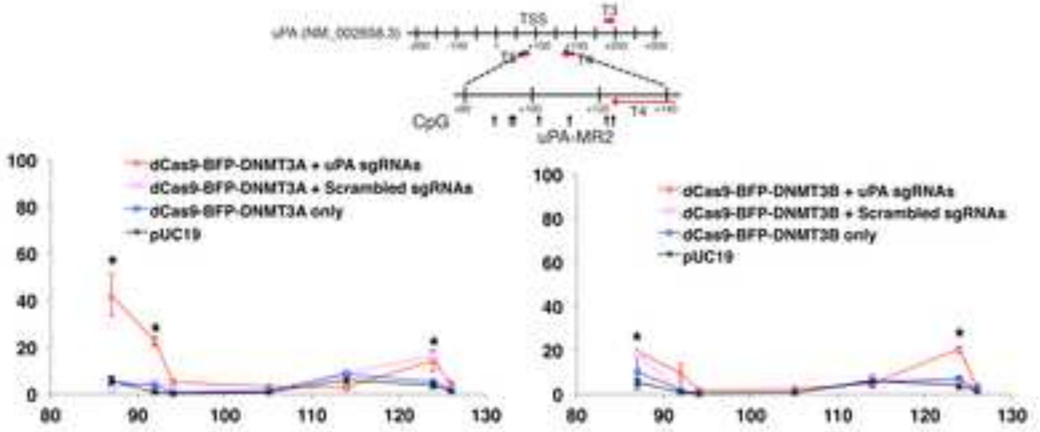

Figure S4

Click here to download Figure Fig.S4.tif

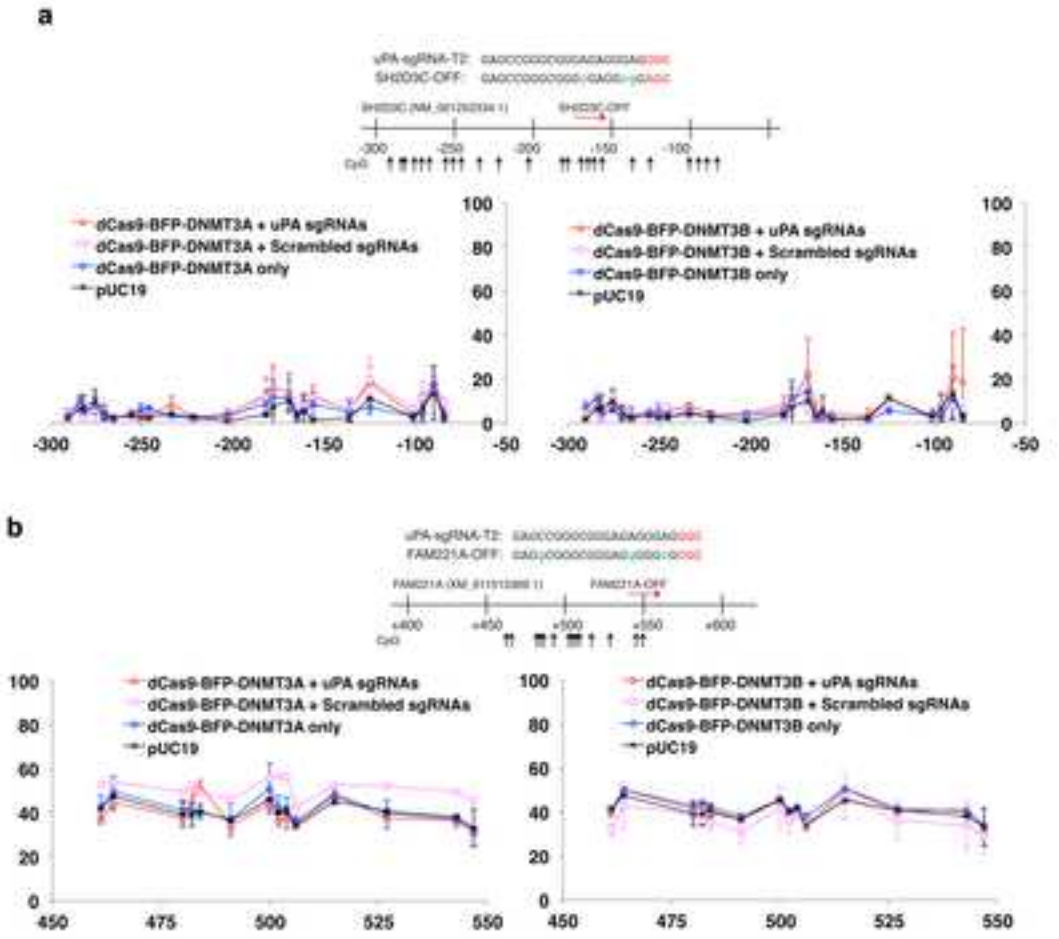

Figure S5

Click here to download Figure Fig.S5.tif

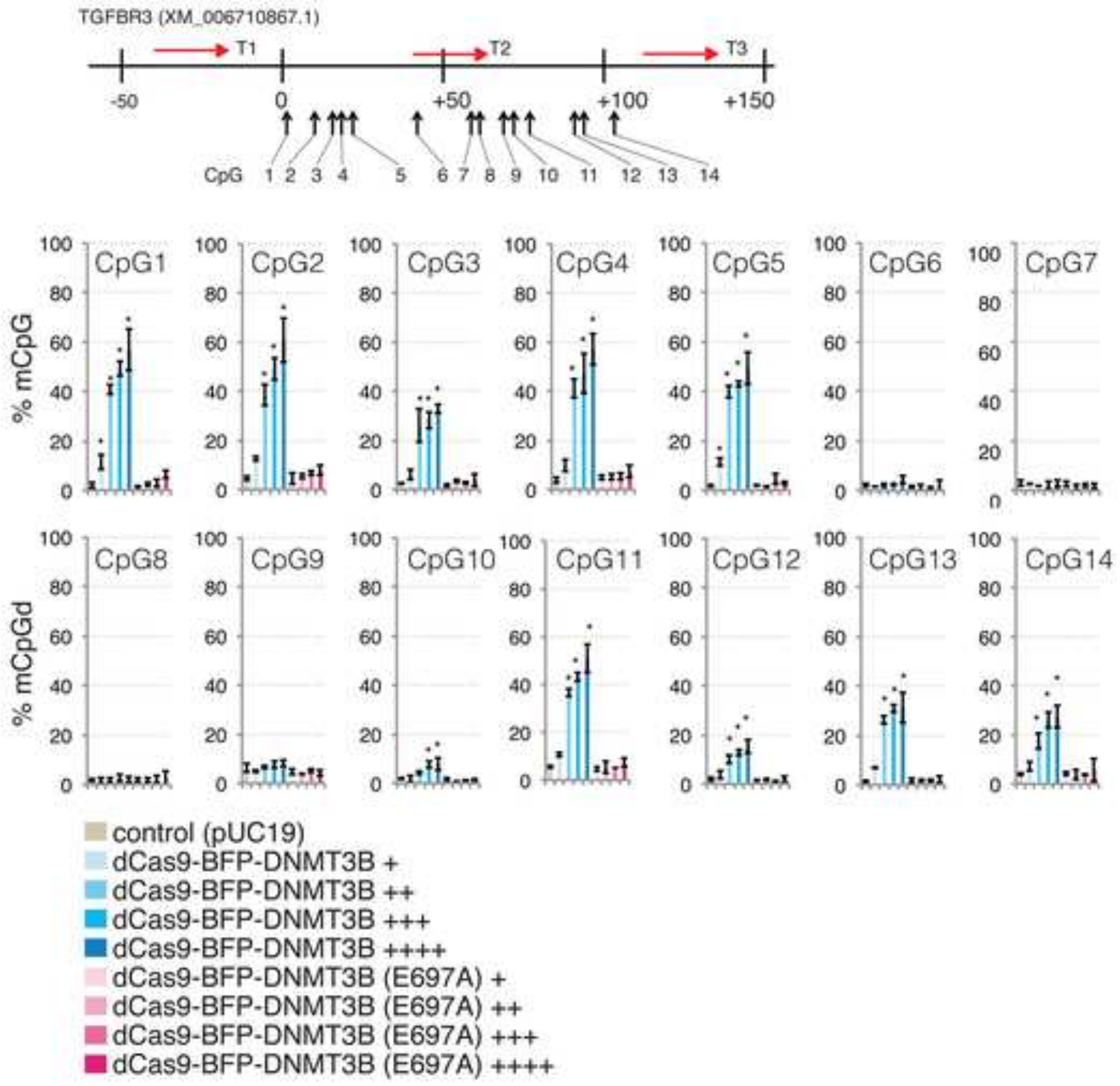

Figure S6

Click here to download Figure Fig.S6.tif

a

Sample ID	Description	Clean Data Size(bp)	Clean Reads Number	Clean Rate(%)	Mapped Reads	Mapping Rate (%)	Uniquely Mapped Reads	Uniquely Mapping Rate (%)	Bisulfite Conversion Rate (%)
g1	dCas9-BFP-DNMT3A (500 ng) + uPA gRNAs (500 ng)	100,601,409,600	670,676,064	95.06	558316453	83.25	558316453	83.25	99.63
g2	dCas9-BFP-DNMT3B (500 ng) + uPA gRNAs (500 ng)	103,778,694,600	691,857,964	95.64	575422550	83.17	575422550	83.17	99.52
g3	dCas9-BFP-DNMT3A (500 ng) + TGFBR3 gRNAs (500 ng)	117,505,796,100	783,371,974	94.06	651431832	83.16	651431832	83.16	99.65
g4	dCas9-BFP-DNMT3B (500 ng) + TGFBR3 gRNAs (500 ng)	109,001,895,600	726,679,304	90.65	604104613	83.13	604104613	83.13	99.63
g5	dCas9-BFP-DNMT3A (500 ng) only	105,265,839,000	701,772,260	90.36	582484794	83	582484794	83	99.62
g6	dCas9-BFP-DNMT3B (500 ng) only	118,394,522,400	789,296,816	95.6	660303371	83.66	660303371	83.66	99.6
g7	dCas9-BFP-DNMT3A (50 ng) + uPA gRNAs (50 ng)	117,366,362,100	782,442,414	87.18	645181340	82.46	645181340	82.46	99.6
g8	dCas9-BFP-DNMT3A (50 ng) + TGFBR3 gRNAs (50 ng)	101,372,416,500	675,816,110	85.25	561172295	83.04	561172295	83.04	99.49
g9	pUC19 control	80,429,413,800	536,196,092	90.08	444407297	82.88	444407297	82.88	99.46

b

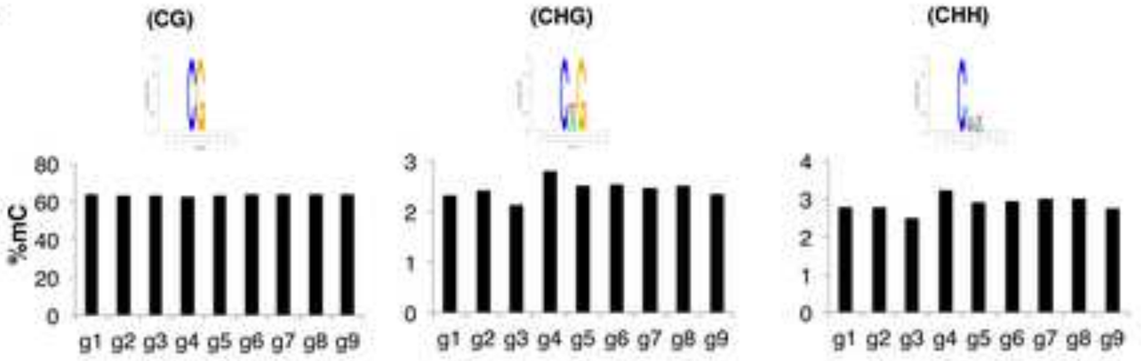

c

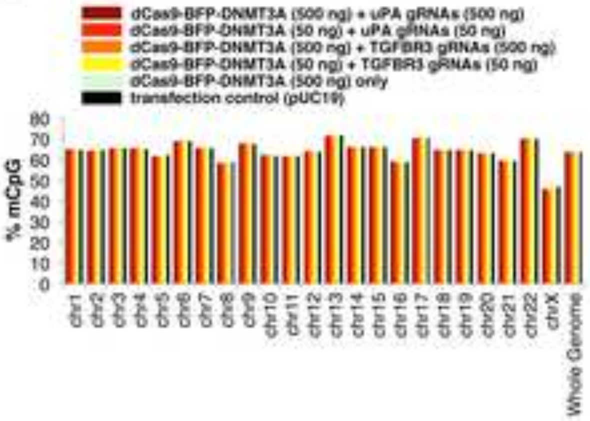

d

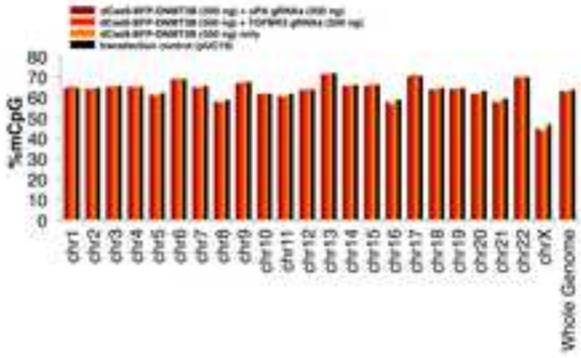

Click here to download Figure Fig.S7.tif

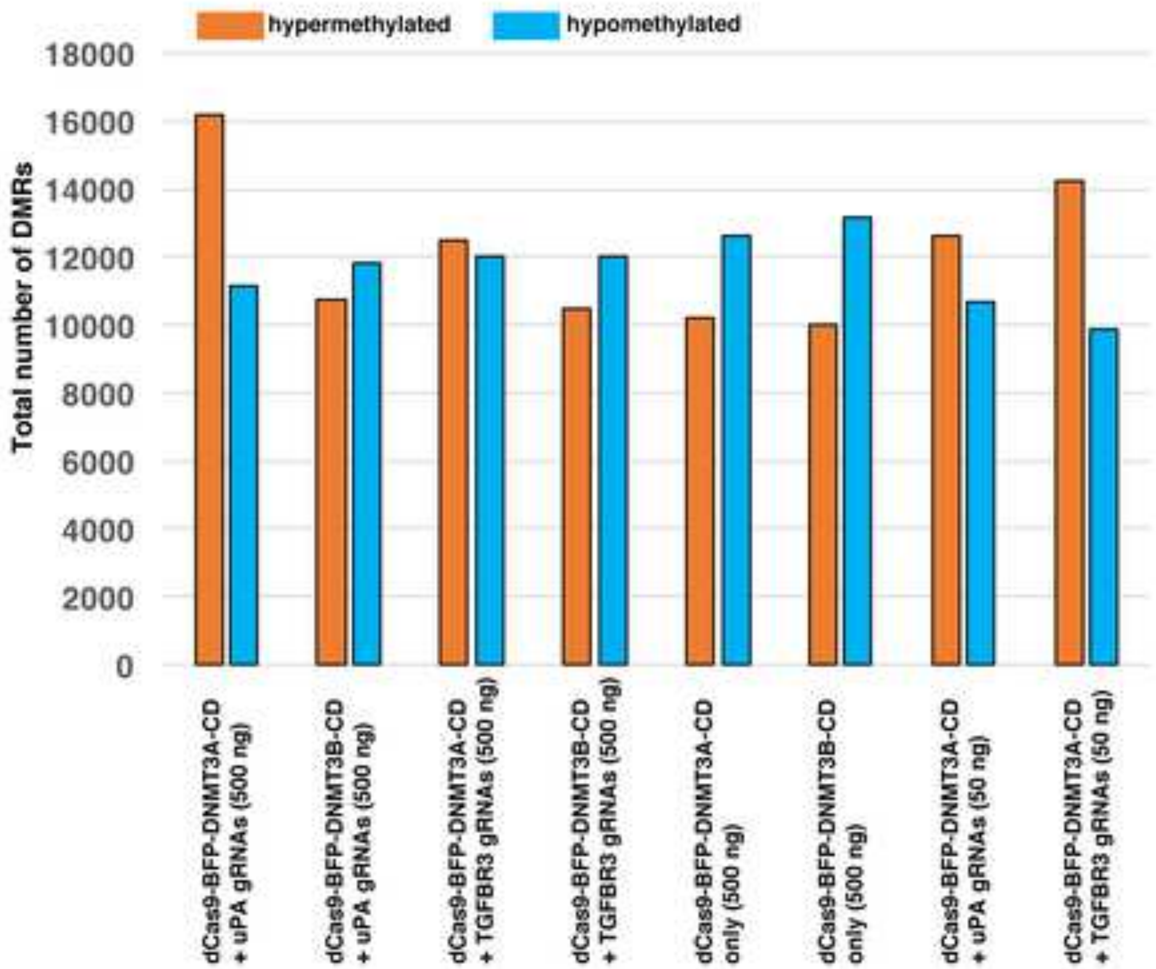

Figure S8

Click here to download Figure Fig.S8.tif

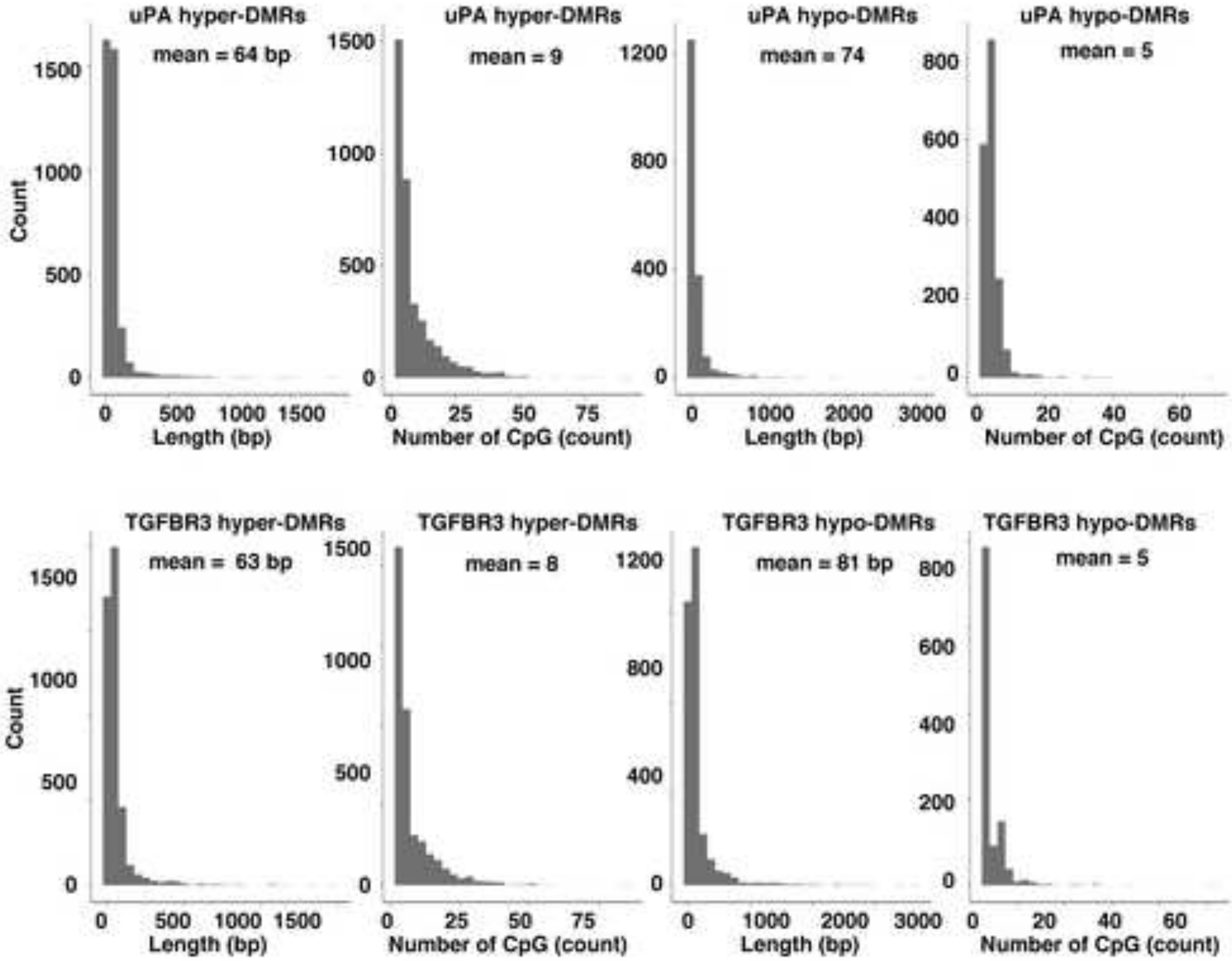

Figure S9

Click here to download Figure Fig.S9.tif

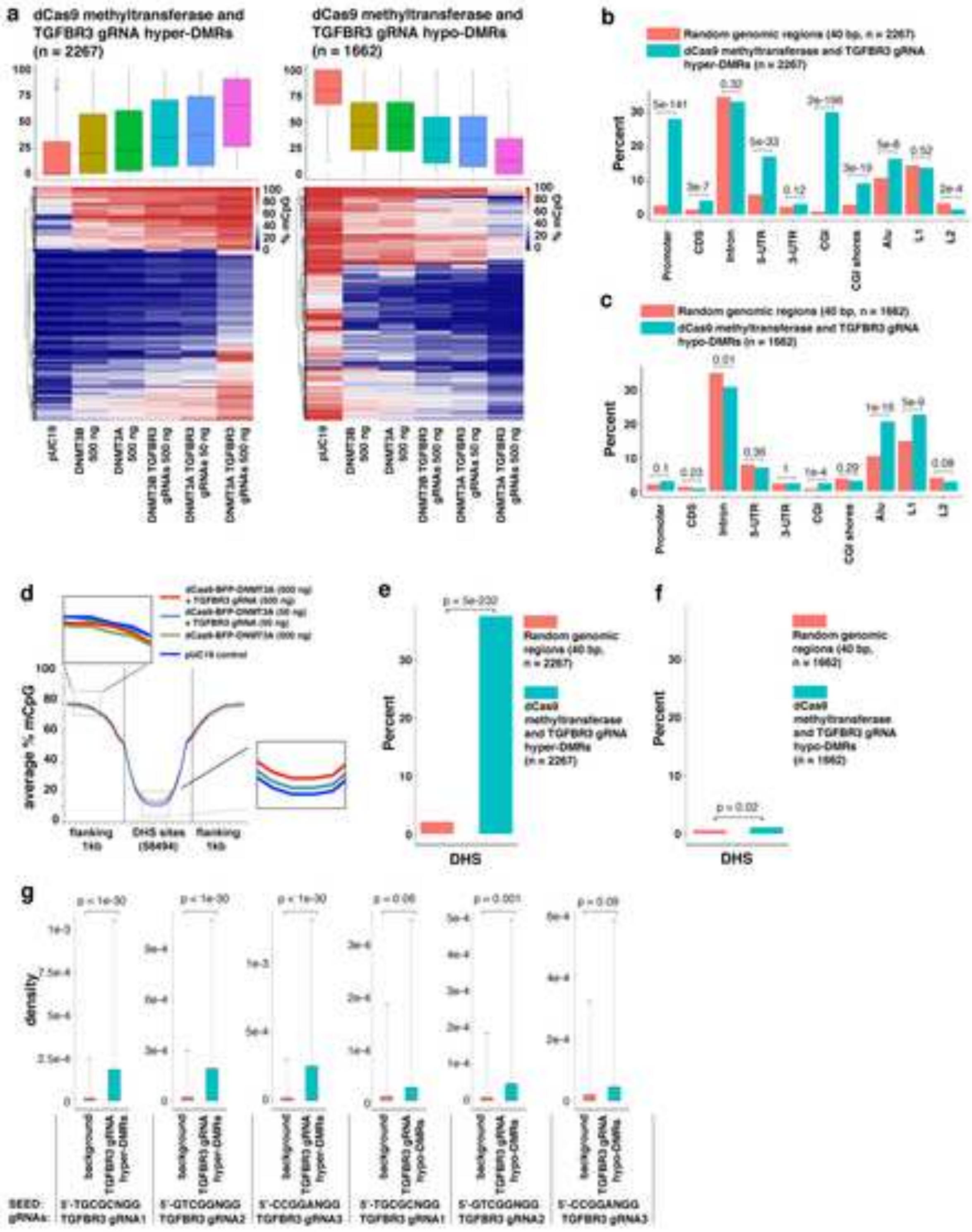

Figure S10

Click here to download Figure Fig.S10.tif

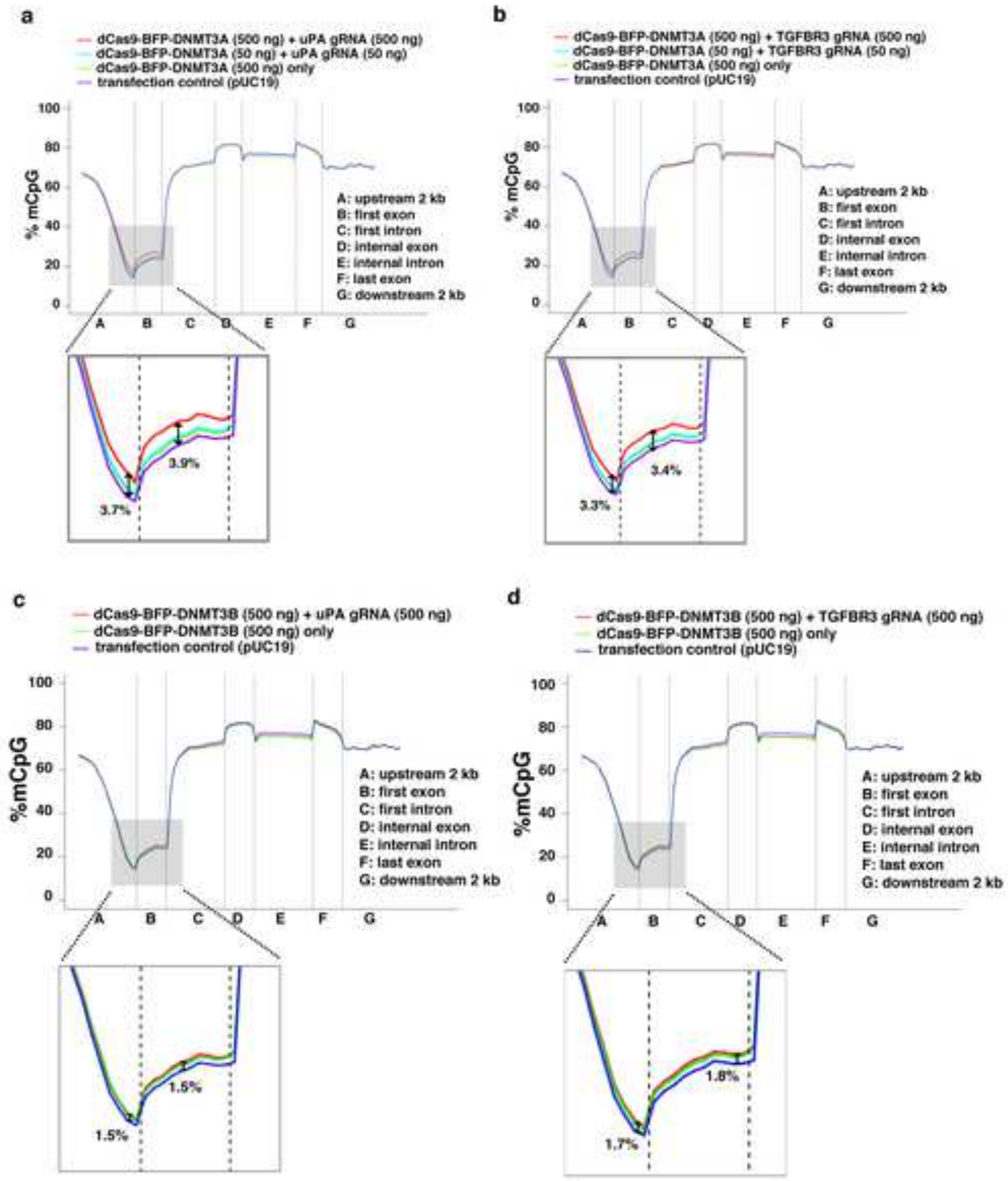

Figure S11

Click here to download Figure Fig.S11.tif

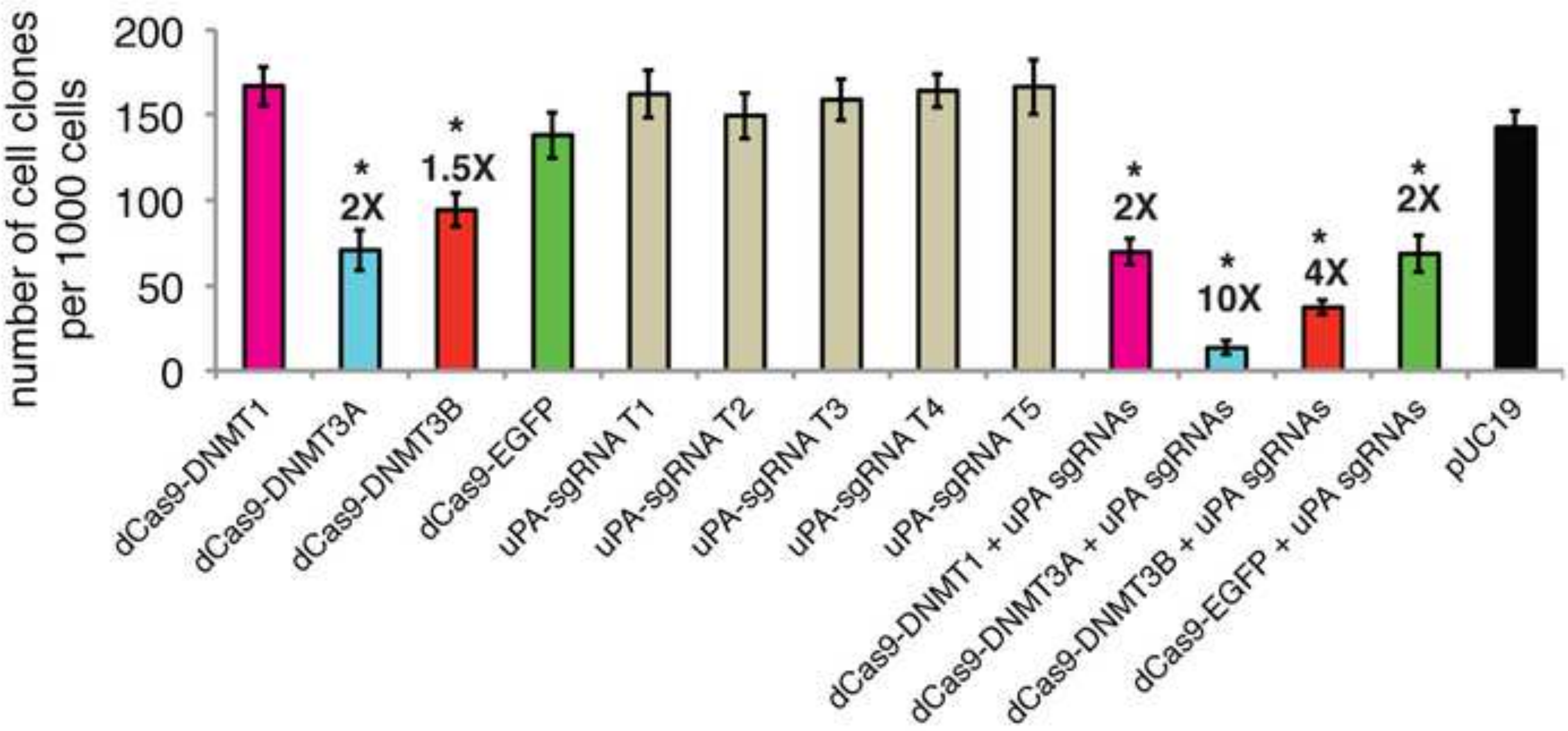

Figure S12

Click here to download Figure Fig.S12.tif

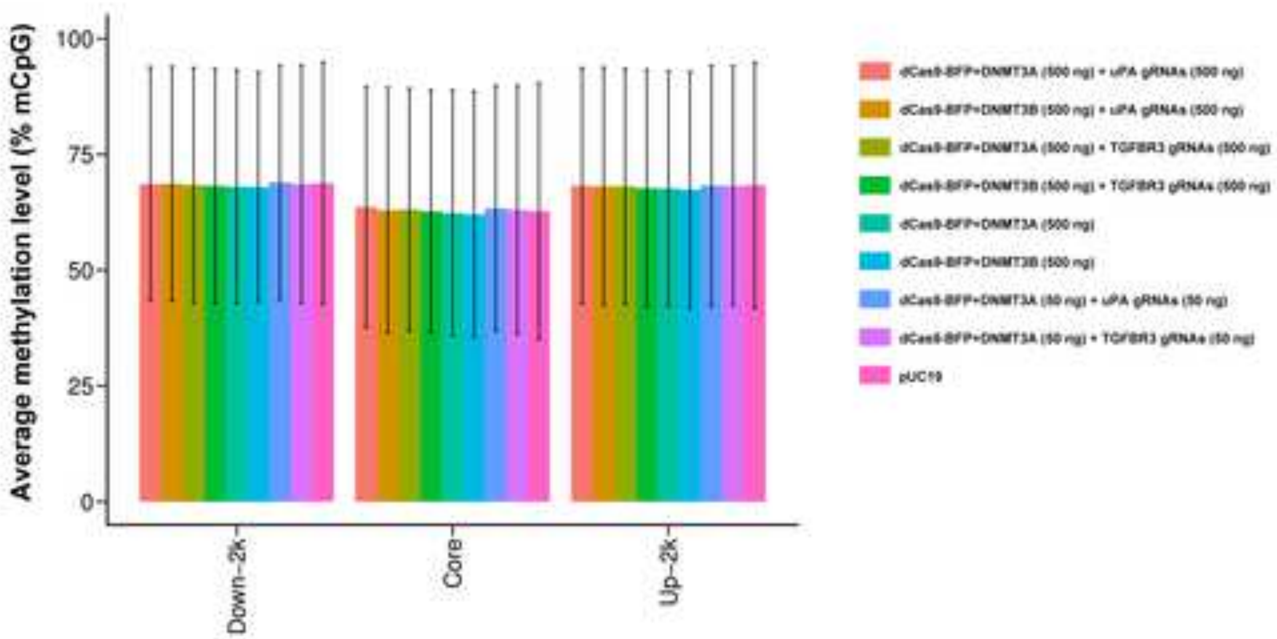

Figure S13 Click here to download Figure Fig.S13.tif

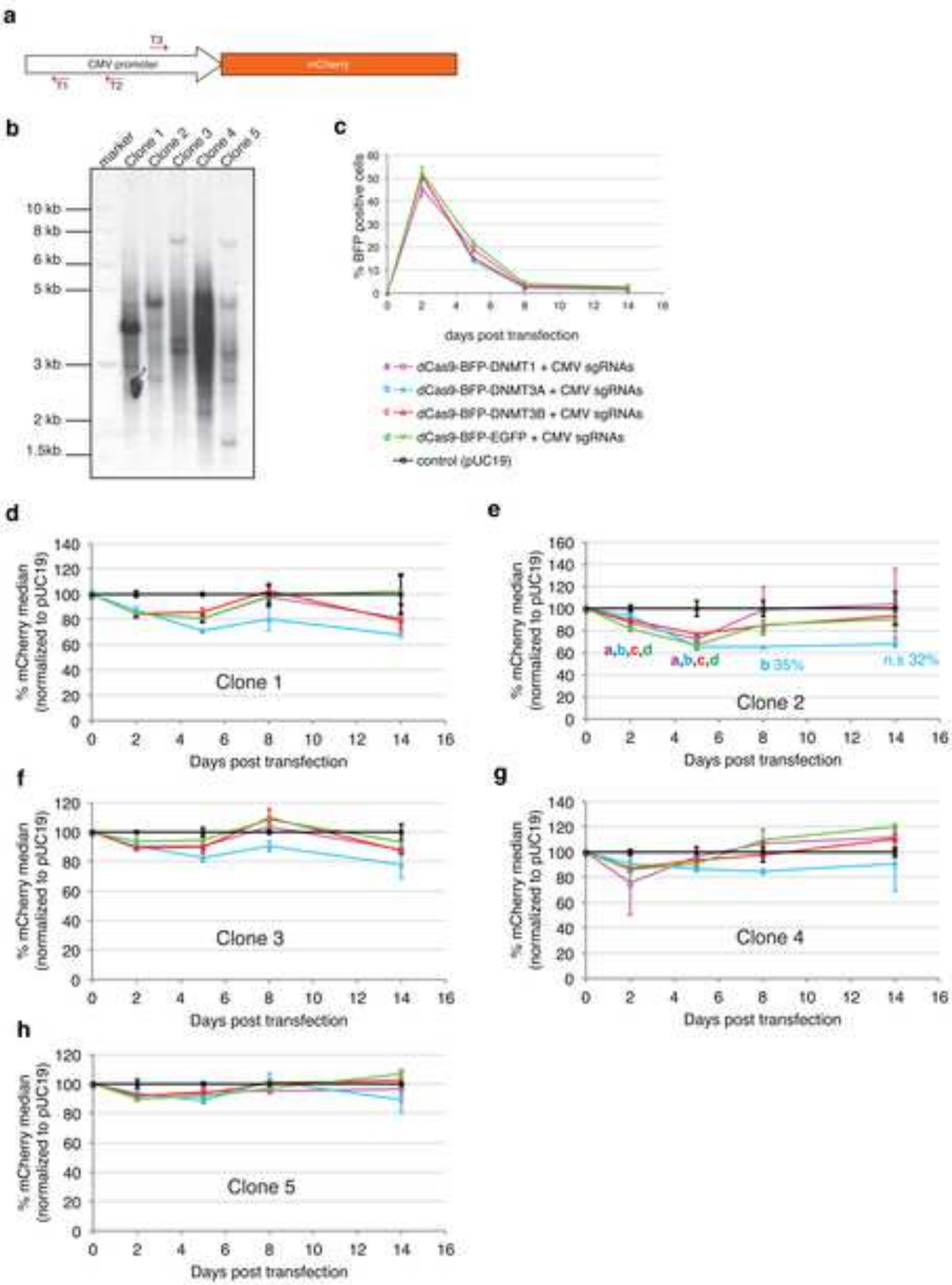

Figure S14

Click here to download Figure Fig.S14N.jpg

Supplementary Figure 14

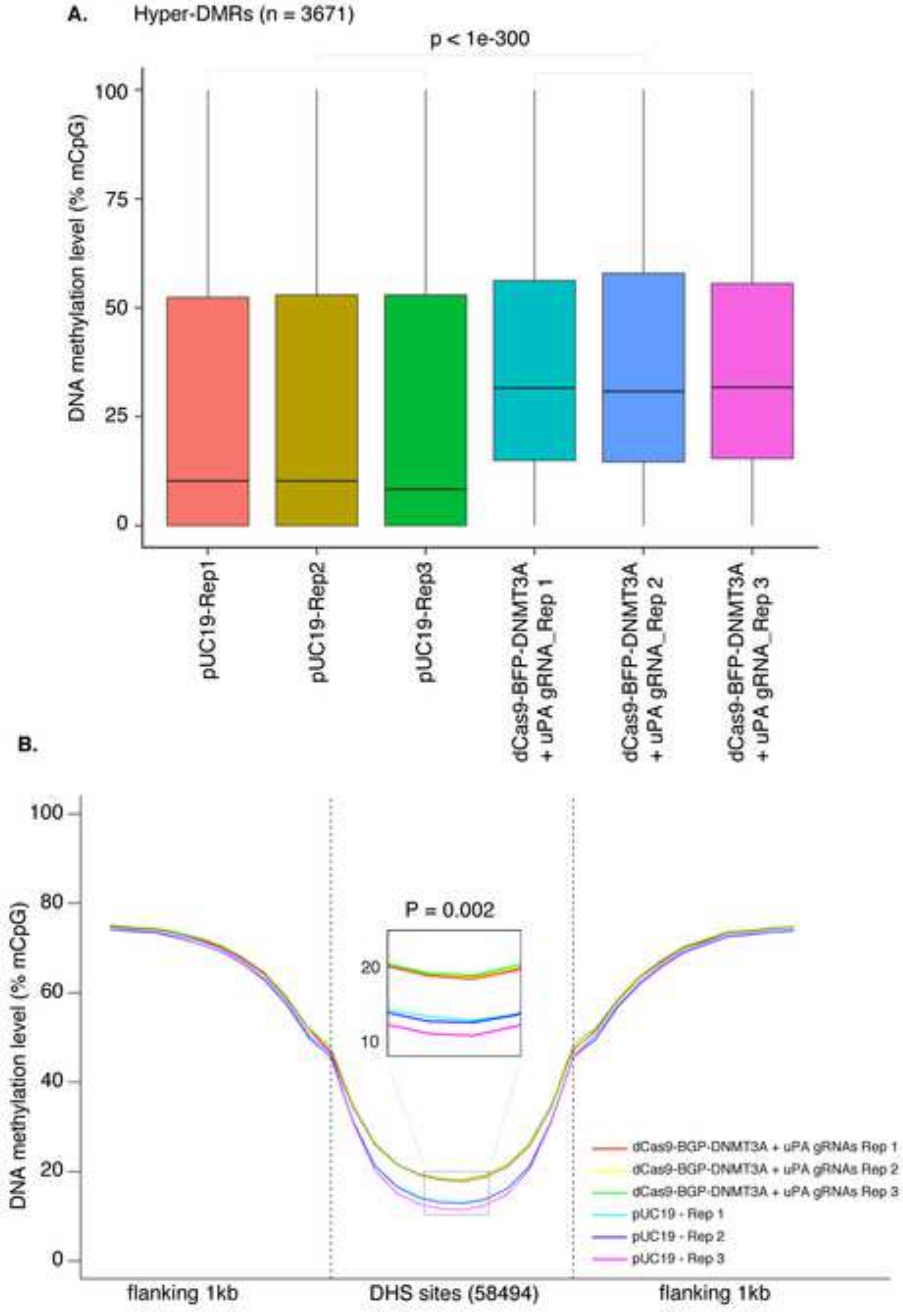

 Click here to download Figure Supplementary File 1- extended text and discussion.pdf

Supplementary File 1: extended text and discussion

DNMT3A and DNMT3B catalytic domain causes gRNA-independent off-target methylation

Lin Lin [1, 7, §], Yong Liu [1, §], Jinrong Huang [2, 3, §], Fengping Xu [2, 3, 4, §], Tina Fuglsang Daugaard [1], Trine Skov Petersen [1], Bettina Hansen [1], Lingfei Ye [2], Qing Zhou [2, 3], Fang Fang [2, 3], Shengting Li [1, 2], Lasse Fløe [1], Kristopher Torp Jensen [1], Ellen Shrock [6], Huanming Yang [2, 4], Jian Wang [2, 4], Xun Xu [2, 3, *], Lars Bolund [1, 2, 7], Anders Lade Nielsen [1], Yonglun Luo [1, 2, 7, 8 *]

Affiliations for authors:
1. Department of Biomedicine, Aarhus University, Aarhus, Denmark
2. BGI-Shenzhen, Shenzhen 518083, China
3. China National GeneBank-Shenzhen， BGI-Shenzhen， Shenzhen 518083, China
4. Department of Biology, University of Copenhagen, Copenhagen, Denmark
5. James D. Watson Institute of Genome Sciences, Hangzhou 310058, China
6. Department of Genetics, Harvard Medical School, Boston, MA, USA.
7. Danish Regenerative Engineering Alliance for Medicine (DREAM), Department of Biomedicine, Aarhus University, Aarhus, Denmark
8. BrainStem - Stem Cell Center of Excellence in Neurology, Copenhagen, Denmark
§. These authors contributed equally to the study
*. All correspondence should be addressed to Xun Xu (XUXUN@GENOMICS.CN) and Yonglun Luo (ALUN@BIOMED.AU.DK)

Main text:
Upon staining of the dCas9-BFP-DNMT3A and dCas9-BFP-DNMT3B fusion proteins in HEK293T cells, it is evident that most fusion protein is located in the cytoplasm rather than in the nucleus. This is probably due to the large size of the fusion proteins hindering efficient nuclear entry. We therefore reduced the size of the fusion proteins by removing the BFP domain in order to increase the nuclear entry efficiency of dCas9 methyltransferase. Furthermore, to enable enrichment of transfected cells, we introduce a blasticidin expression cassette.

Five expression vectors were constructed encoding the human-codon-optimized dCas9 protein flanked by two copies of an NLS and conjugated to the DNMT3A or DNMT3B catalytic domains, or DNMT3A (E752A), DNMT3B (E697A) or GFP, as well as a blasticidin resistance domain (**Fig. SF1a**). In the system utilized for the experiment, the blasticidin domain is cleaved from the fusion protein upon translation via the self-cleaving 2A peptide.

We first evaluated the *de novo* DNA methylation efficiency of dCas9-DNMT3A and dCas9-DNMT3B (**Fig. SF1b-c**). 10-fold and 3-fold increases of *de novo* methylation of the *TGFBR3* promoter were achieved in cells expressing *TGFBR3* gRNAs with dCas9-DNMT3A or dCas9-DNMT3B, respectively, compared to pUC19 or dCas9-EGFP controls (P value < 0.001, ANOVA). As expected, dCas9-DNMT3A(E752A) or dCas9-DNMT3B(E697A) lacked *de novo* methylation capacity. However, compared to the dCas9-BFP-DNMT3A and dCas9-BFP-DNMT3B fusions, the dCas9-DNMT3A and dCas9-DNMT3B fusions have higher gRNA-independent unspecific methylation (**Fig. SF1d**).

We speculated that the increased unspecific methylation by dCas9-DNMT3A and dCas9-DNMT3B was related to increased gene expression and/or nuclear localization efficiency. Thus, we used fluorescence

imaging to quantify the expression level and nuclear localization of the three EGFP control plasmids: dCas9-BFP-EGFP, dCas9-EGFP, and EF1a-EGFP, our results showed that both expression and nuclear entry levels of dCas9-EGFP is significantly higher (*P* value < 0.05, ANOVA) than dCas9-BFP-EGFP (**Fig. SF2a**). Furthermore, we analyzed *GAPDH* promoter methylation in cells expressing dCas9-BFP-DNMT3A, dCas9-DNMT3A, or EF1a-DNMT3A (a control plasmid expressing the DNMT3A catalytic domain under transcriptional control of the EF1a promoter). Our results showed that cells expressing dCas9-DMNT3A and EF1a-DNMT3A showed notably higher mCpG levels than cells transfected with pUC19 (mean diff. mCpG level = 11.7% and 38.5%, *P* value =0.012, Wilcoxon matched-pairs signed-rank test), while the difference between cells expressing dCas9-BFP-DNMT3A (CRISPRme1.0) and pUC19 was much smaller (mean diff. mCpG level = 2.3%, *P* value = 0.012, Wilcoxon matched-pairs signed-rank test) (**Fig. SF2b**).

We next measured *LINE1* 5'UTR methylation. There was no significant difference in *LINE1* 5'UTR DNA methylation between cells expressing dCas9-BFP-DNMT3A and dCas9-DNMT3A (**Fig. SF2c**). However, cells transfected with the expression vector EF1a-DNMT3A had significantly higher *LINE1* methylation levels compared to pUC19 control cells (mean diff. % mCpG = 3.1, P value = 0.028, Wilcoxon matched-pairs signed-rank test) (**Fig. SF2c**), suggesting that increased CRISPRme expression and nuclear entry is accompanied by increased gRNA-independent off-target methylation of DNMT3A and DNMT3B fusion proteins.

We also investigated whether titration of the dCas9-DNMT3A and dCas9-DNMT3B plasmids used for transfection could reduce gRNA-independent off-target methylation. To test this, we transfected HEK293T cells with a varying amount of dCas9-DNMT3A plasmid (5, 25, 50, 100, 250, and 500 ng) alone or together with *TGFBR3* gRNAs. Titration of dCas9-DNMT3A caused a concordant decrease in both *TGFBR3* and *GAPDH* methylation (**Fig. SF3a-d**). Next, we tested whether decreasing the gRNA expression level could minimize gRNA-dependent off-target effects. We transfected HEK293T cells with various combinations of the amounts of dCas9-DNMT3A plasmid (5, 25, and 50 ng) and *TGFBR3* gRNAs (5, 25, 50, and 500 ng). Bisulfite pyrosequencing results showed that titration of gRNA transfection quantities decreased both on-target (*TGFBR3*) and off-target (*GAPDH*) methylation concordantly (**Fig. SF3e-h**). Collectively, our results suggest that increasing the expression of dCas9-DNMT3A and gRNAs results in enhancement of both on-target and off-target DNA methylation.

In conclusion, we have shown that fusion of dCas9 to catalytic domains of DNA methyltransferases can achieved RNA-guided methylation of a targeted genomic regions. However, since these catalytic domains are active per se, cautions should be taken when delivering high amount of the fusion proteins/plasmids into cells, as these could cause unspecific methylation of genomic regions. Although GWBS was not conducted for the dCas9-DNMT3A and dCas9-DNMT3B fusions, the genomic regions prone to gRNA-independent methylation are most likely those located in open chromatin regions and CpG islands.

Figures and Figure Captions

Fig. SF1. Generation and validation of dCas9-DNMT3A and dCas9-DNMT3B methyltransferases

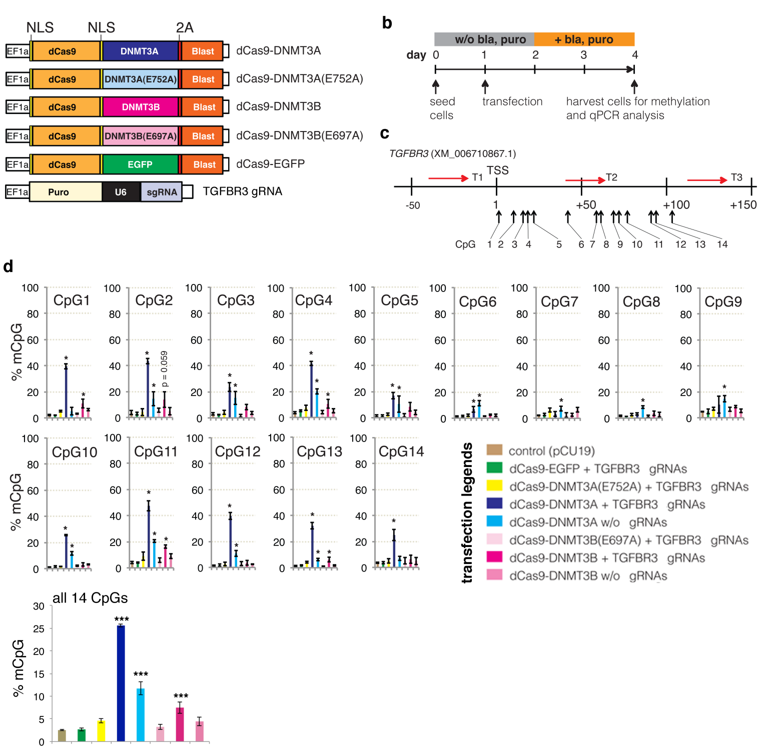

(**a**) Schematic illustration of CRISPRme 2.0 expression vectors encoding dCas9 flanked by nuclear localization signals (NLS) and fused to (i) a DNMT3A or DNMT3B catalytic domain, (ii) a DNMT3A (E752A) or DNMT3B (E697A) catalytically inactivated domain, or (iii) EGFP, and a blasticidin resistance gene. Expression of the fusion proteins was constitutively driven by the elongation factor-1 alpha (EF1a) promoter.

(**b**) Schematic illustration of the experiment. Transfected cells were enriched by blasticidin (10 ug/mL) and puromycin (10 ug/mL) antibiotic selection.

(**c**) *TGFBR3* promoter with gRNA target sites and CpGs used for subsequent analysis by pyrosequencing.

(**d**) Bar charts depicting % mCpG for individual and all *TGFBR3* CpG sites in cells expressing dCas9-DNMT3A, dCas9-DNMT3B, dCas9-DNMT3A(E752A), or dCas9-DNMT3B(E697A) with or without *TGFBR3* gRNAs. Cells transfected with pUC19 or a dCas9-EGFP fusion were used as negative controls. % mCpG and relative gene expression values are presented as mean ± SD (n = 3 independent transfections). Asterisks represent P value < 0.05 (*) and < 0.001 (***) (ANOVA). Percentage decrease in gene expression levels compared to pUC19 are presented on top of bars in (**d**).

Fig. SF2 Comparison of dCas9 methyltransferase expression, nuclear entry efficiency, and the effects on specificity

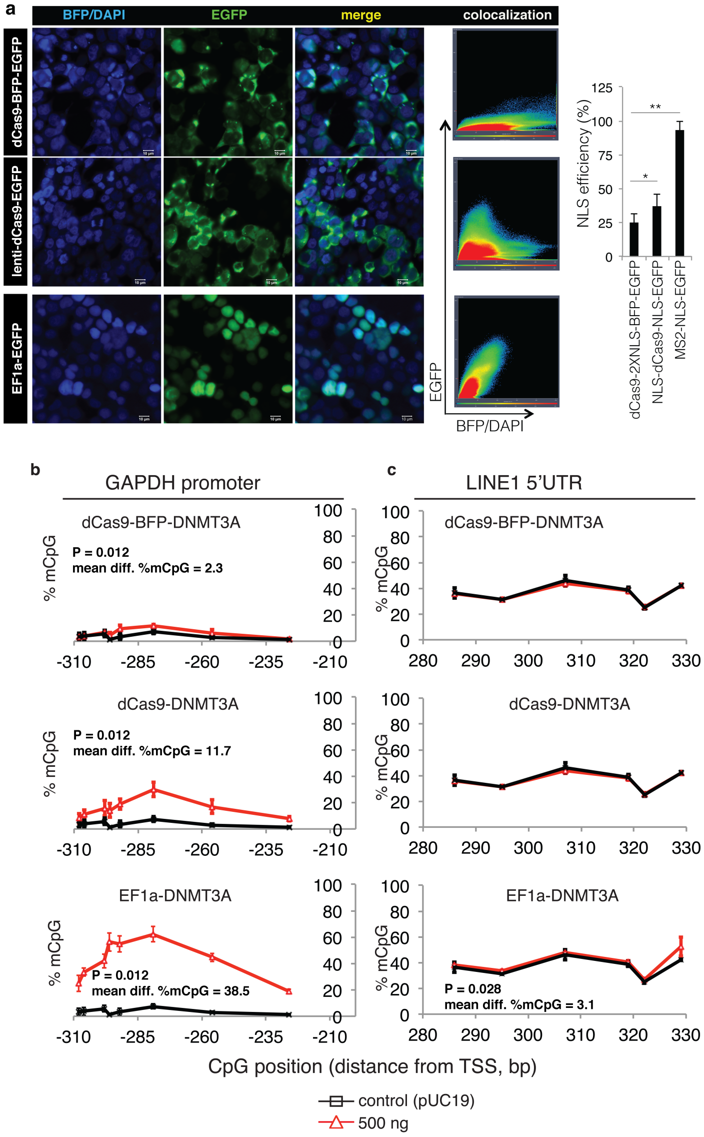

(**a**) Laser scanning fluorescent microscopy of HEK293T cells expressing three EGFP fusion proteins. NLS, nuclear localization signal; BFP, blue fluorescent protein; MS2, the MS2 bacteriophage coat protein. Co-localization of EGFP and BFP/DAPI is observed. Nuclear localization was estimated using Image J. *, p-value < 0.05; **, p-value < 0.01 by ANOVA.

(**b-c**) Percentage methylation of *GAPDH* (**b**) and *LINE1* 5'UTR (**c**). Each data point is presented as mean ± SD (n = 3, independent transfections). % mCpG levels were analyzed in cells 48 hours after transfection with dCas9-BFP-DNMT3A, lenti-dCas9-DNMT3A, or EF1a-DNMT3A plasmids (500 ng). Cells transfected with an identical amount of the pUC19 plasmid were used as control and re-plotted for the different treatments as reference. Average difference in % mCpG levels between experimental groups and controls are shown together with Wilcoxon matched-pairs signed-rank test P values.

Fig. SF3. Effects of titrating dCas9-DNMT3A, dCas9-DNMT3B and gRNA plasmids on on-target and off-target methylation

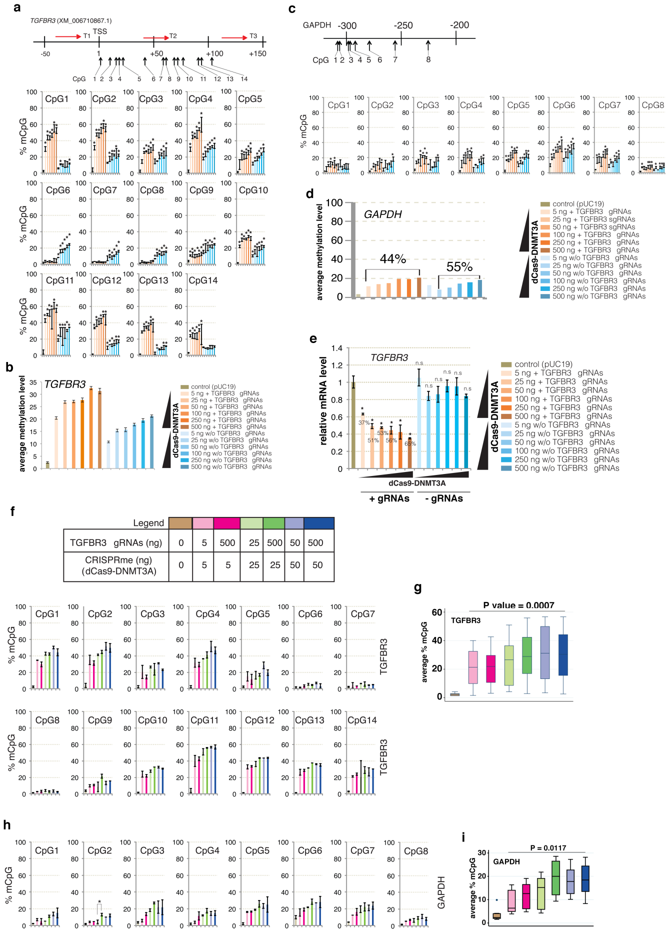

(**a-b**) Bar charts illustrating % mCpG levels for the 14 individual *TGFBR3* CpG sites (**a**) and the average methylation level for all 14 CpG sites. (**b**) in HEK293T cells expressing various amounts (5, 25, 50, 100, 250, and 500 ng) of the dCas9-DNMT3A plasmid with or without (w/o) *TGFBR3* gRNAs (500 ng).

(**c-d**) Bar charts illustrating % mCpG levels for individual CpG sites (**c**) or average methylation levels (**d**) in the *GAPDH* promoter in cells expressing different levels (5, 25, 50, 100, 250, and 500 ng) of dCas9-DNMT3A with or without *TGFBR3* gRNAs (500 ng). Cells transfected with pUC19 were used as controls. Asterisk (*) indicates statistical significance ($p < 0.05$, ANOVA) after Bonferroni correction.

(**e**) Bar charts of relative *TGFBR3* mRNA levels compared to control cells transfected with pUC19. Both CpG methylation and gene expression data represent mean ± SD (n = 3 independent transfections). Asterisk (*) indicates statistical significance (P value < 0.05, ANOVA) after Bonferroni correction.

(**f**) (**top**) Transfection legend. HEK293T cells were co-transfected with various amounts of dCas9-DNMT3A (5, 25, and 50 ng) and the *TGFBR3* gRNAs (5, 25, 50, and 500 ng). (**bottom**), bar charts of % mCpG for individual CpG sites in the *TGFBR3* promoter (TGFBR3-DMR1).

(**g**) Box plot of the average of % mCpG for all 14 CpG sites in the *TGFBR3* promoter for each experimental group.

(**h**) Bar charts illustrating % mCpG for individual CpG sites in the *GAPDH* promoter.

(**i**) Box plot of the average % mCpG for all 8 CpG sites in the *GAPDH* promoter for each experimental group.

Figures (**f, h**) are plotted as mean ± SD (n = 2, independent transfections). (**g, i**) P values are from Wilcoxon matched-pairs signed-rank tests between the 5 ng and 500 ng experimental groups.

Table S1

Click here to access/download
Supplementary Material
Supplementary Table S1.docx

Table S2

Click here to access/download
Supplementary Material
Supplementary Table S2.xls

Table S3

Click here to access/download
Supplementary Material
Supplementary Table S3.xls

Table S4

Click here to access/download
Supplementary Material
Supplementary Table S4.xls

Table S5

Click here to access/download
Supplementary Material
Supplementary Table S5.xls

Table S6

Click here to access/download
Supplementary Material
Supplementary Table S6.xls

Cover letter Click here to download Personal Cover Cover letter.doc

DEPARTMENT OF BIOMEDICINE
FACULTY OF HEALTH
AARHUS UNIVERSITY

To GigaScience editors

Dear *Editor Scott Edmunds*:

First of all, we would like to thank you and the reviewers constructive and valuable comments and suggestions for the further improvement of our study. It is very important that we bring the correct and significance finding, technology and knowledge to the broad readers of GigaScience and the scientific community. Secondly, we really appreciate the extended time for us to prepare a better revision.

Department of Biomedicine
Aarhus University

Yonglun Luo

PhD. Associate Professor

Date: June. 29, 2017

Direct Tel.: +45 8716 7761
Fax: +45 8612 3173
E-mail: alun@biomed.au.dk

In the revision, all important changes are highlighted in blue text. Just to highlight a few major revisions here for you:

1. We have conducted two more dCas9 methyltransferase and uPA gRNA ChIP-seq repeats. These NGS data will be deposited to the GigaScience database.
2. We have included both hyper-methylated and hypo-methylated DMRs into the analysis in the revision.
3. We have included a more stringent filtering step for the detection of DMRs.
4. The manuscript has been greatly shorten. We have prepared a separated section for the CRISPR2.0 study, and would like to present it as extended supplementary results.
5. We have carefully revised our conclusion on DNA methylation and gene expression.

Thanks again for giving us the opportunity to submit our revised study for consideration of publication in your journal.

Sincerely,

Yonglun Luo, on behalf of all authors.

29/06/2017, Iceland.

Department of Biomedicine
Aarhus University
Building 11240
Wilhelm Meyers Alle 4
8000 Aarhus C

E-mail: biomed@au.dk
http://www.biomed.au.dk
