# Supplementary material for: Genome-wide determination of on-target and off-target characteristics for RNA-guided DNA methylation by dCas9 methyltransferases
Source: Gigascience. 2018 Feb 19;7(3):giy011. doi: 10.1093/gigascience/giy011 (PMC5888497; doi:10.1093/gigascience/giy011)
Supplement: Supplemental material [file giy011_supp.zip › Supplementary Table S1.docx]

**Supplementary Table S1**

| **CRISPRme plasmids deposited to Addgene** | | | | | | | | | |
| --- | --- | --- | --- | --- | --- | --- | --- | --- | --- |
| **Plasmid name** | | **description** | | | **referred as in the article** | **Addgene ID** | | | **GenBank accession numbers** |
| pCCL-PGK-SPdCas9-BFP-DNMT1 | | dCas9 fused to BFP and the human DNMT1 catalytic domain | | | dCas9-BFP-DNMT1 | 66818 | | | MG840304 |
| pCCL-PGK-SPdCas9-BFP-DNMT3A | | dCas9 fused to BFP and the human DNMT3A catalytic domain | | | dCas9-BFP-DNMT3A | 66819 | | | MG840305 |
| pCCL-PGK-SPdCas9-BFP-DNMT3B | | dCas9 fused to BFP and the human DNMT3B catalytic domain | | | dCas9-BFP-DNMT3B | 66820 | | | MG840306 |
| pCCL-PGK-SPdCas9-BFP-EGFP | | dCas9 fused to BFP and EGFP | | | dCas9-BFP-EGFP | 66821 | | | MG840307 |
| pCCL-PGK-SPdCas9-BFP-DNMT3A(E752A) | | dCas9 fused to BFP and the human DNMT3A catalytically inactive domain | | | dCas9-BFP-DNMT3A(E752A) | 71213 | | | MG840308 |
| pCCL-PGK-SPdCas9-BFP-DNMT3B(E697A) | | dCas9 fused to BFP and the human DNMT3B catalytically inactive domain | | | dCas9-BFP-DNMT3B(E697A) | 71214 | | | MG840309 |
| pLenti-EF1a-SPdCas9-EGFP-2A-Blast | | dCas9 fused to EGFP | | | lenti-dCas9-EGFP | 71215 | | | MG840310 |
| pLenti-EF1a-SPdCas9-DNMT3A-2A-Blast | | dCas9 fused to human DNMT3A catalytic domain | | | lenti-dCas9-DNMT3A | 71216 | | | MG840311 |
| pLenti-EF1a-SPdCas9-DNMT3B-2A-Blast | | dCas9 fused to human DNMT3B catalytic domain | | | lenti-dCas9-DNMT3B | 71217 | | | MG840313 |
| pLenti-EF1a-SPdCas9-DNMT3A(E752A)-2A-Blast | | dCas9 fused to the human DNMT3A catalytically inactive domain | | | lenti-dCas9-DNMT3A(E752A) | 71218 | | | MG840312 |
| pLenti-EF1a-SPdCas9-DNMT3B(E697A)-2A-Blast | | dCas9 fused to the human DNMT3B catalytically inactive domain | | | lenti-dCas9-DNMT3B(E697A) | 71219 | | | MG840314 |
| **CRISPRme sgRNA guide sequences** | | | | | | | | | |
| **Oligo name** | **orientation** | | **Sequences 5’-3’** | | | | **Note** | | |
| uPA-T1 | + strand | | GTGAGCGTTGCGGAAGCACG | | | |  | | |
| uPA-T2 | + strand | | GAGCCGGGCGGGAGAGGGAG | | | |  | | |
| uPA-T3 | + strand | | GAGTCAAGGCGCCCCGTCCC | | | |  | | |
| uPA-T4 | - strand | | GCCCGGGGATCTCAGGACCG | | | |  | | |
| uPA-T5 | - strand | | GGGACAGGTGGACCCTGGCC | | | |  | | |
| CMV-T1 | - strand | | GTCCCTATTGGCGTTACTAT | | | | scrambled sgRNA | | |
| CMV-T2 | - strand | | GTCCCATAAGGTCATGTACT | | | | scrambled sgRNA | | |
| CMV-T3 | + strand | | GTGGATAGCGGTTTGACTCA | | | | scrambled sgRNA | | |
| TGFBR3-T1 | + strand | | GCGAGGGAGGGCGAGTGCGC | | | |  | | |
| TGFBR3-T2 | + strand | | GAGTGAAGGAGGGCAGTCGG | | | |  | | |
| TGFBR3-T3 | + strand | | GAGTCCCCAGCGGGTCCGGA | | | |  | | |
|  |  | |  | | | |  | | |
| **PCR primers** | | | | | | | | | |
| **Gene** | **orientation** | | **Sequences 5’-3’** | | | | **Note** | | |
| uPA | forward | | GGGAATGGTCACTTTTACCGAG | | | | qPCR | | |
| uPA | reverse | | GGGCATGGTACGTTTGCTG | | | | qPCR | | |
| TGFBR3 | forward | | ACTGGGGTCTCCAGACTG | | | | qPCR | | |
| TGFBR3 | reverse | | TCATTTCCATGGGGGAAG | | | | qPCR | | |
| GAPDH | forward | | TGGTATCGTGGAAGGACTCATGAC | | | | Reference gene for qPCR | | |
| GAPDH | reverse | | ATGCCAGTGAGCTTCCCGTTCAGC | | | | Reference gene for qPCR | | |
| mCherry | forward | | ATGGTGAGCAAGGGCGAGGAG | | | | Southern blot – probe PCR | | |
| mCherry | reverse | | CAGCTCGTCCATGCCGCCGGTGG | | | | Southern blot – probe PCR | | |
| **Bisulfite PCR and Pyrosequencing primers** | | | | | | | | | |
| **Gene** | **Primers** | | | | | | | **Note** | |
|  | **Name** | | | **Sequences 5’-3’ (Bio, biotin-labeled)** | | | |  | |
| uPA | uPA-MR1-F | | | GTTAGGTGTATGGGAGGAAGTA | | | | Primers for bisulfite PCR of uPA-MR1 | |
|  | uPA-MR1-R | | | **Bio-**ACTCCCTCCCCTATCTTACAAC | | | |  |  |
|  | uPA-MR1-pyro-seq | | | GGAGAATTTATAAGTT | | | | Pyrosequencing primer | |
|  | uPA-MR1 | | | TCTCGATTCCTCAGTCCAGACGCTGTTGGGTCCCCTCCGCTGGAGATCGCGCTTCCCCCAAATCTTTGTGAGCGTTGCGGAAGCACGCGGGGTCCGGGTCGCTGAGCGCTG | | | | sequences to be analyzed before bisulfite treatment | |
| uPA | uPA-MR2-F | | | GGGTTAGGGTTTATTTGTTTT | | | | Primers for bisulfite PCR of uPA-MR2 | |
|  | uPA-MR2-R | | | Bio-AACCAAACTCCCCAACTATCTCT | | | |  |  |
|  | uPA-MR2-pyro-seq | | | GGGTTAGGGTTTATT | | | | Pyrosequencing primer | |
|  | uPA-MR2 | | | TGTCCCCGCAGCGCCGGCTCGCGCCCTCCTGCCGCAGCCACCGGTGAGTGCCGCGG | | | | sequences to be analyzed before bisulfite treatment | |
| TGFBR3 | TGFBR3-MR1-F | | | TCGGTTTGATGGGGGTAAT | | | | Primers for bisulfite PCR of TGFBR3-MR1 | |
|  | TGFBR3-MR1-R | | | Bio-TCACCTCCTACAAAAAACTC | | | |  |  |
|  | TGFBR3-MR1-pyro-seq | | | TCGGTTTGATGGGGGTAAT | | | | Pyrosequencing primer | |
|  | TGFBR3-MR1 | | | CGAGGGTTTCGGGGACGCCGAGCGGCACTTTCCTCTTCCCAGCGAGTGAAGGAGGGCAGTCGGCGGCTCTCGCGCCCCGGCCACTTTCCCTGCGCGATTCCCGGAG | | | | sequences to be analyzed before bisulfite treatment | |
| TGFBR3 | TGFBR3-MR2-F | | | AGTTTTTTGTAGGAGGTGAGAG | | | | Primers for bisulfite PCR of TGFBR3-MR2 | |
|  | TGFBR3-MR2-R | | | Bio-CAACCTACAAAACCCACAAC | | | |  |  |
|  | TGFBR3-MR2-pyro-seq | | | AGTTTTTTGTAGGAGGTGAGAG | | | | Pyrosequencing primer | |
|  | TGFBR3-MR2 | | | TCCCCAGCGGGTCCGGATGGCGTAGTTTTGCCGCGGCGCAGCAGCTGCCGGAGCTCGCCGCCGCCGAGCGCTGGGCGGGGAAACTTGCCGCCGCTTTCCTCCAACTTGCTGCGGGTGGATCTCCGCTGGACACACCGCCTCCGAG | | | | sequences to be analyzed before bisulfite treatment | |
| SH2D3C | SH2D3C-PCR1-F | | | GGAGTTTGGTTTTTTTTATTTAGGGAAGT | | | | Primers for bisulfite PCR1 of SH2D3C | |
|  | SH2D3C-PCR1-R | | | Bio-CCCCTCCCCATCAATCAATATC | | | |  |  |
|  | SH2D3C-PCR2-F | | | GGAGAGTTGTTGGGGAAG | | | | Primers for bisulfite PCR2 of SH2D3C | |
|  | SH2D3C-PCR2-R | | | Bio-CCACTAAACTACAAAACTCAATACC | | | |  |  |
|  | SH2D3C-PCR1-pyro | | | GGAGTTTGGTTTTTTTTATTTAGGGAAGT | | | | Pyrosequencing primer for PCR1 | |
|  | SH2D3C-PCR2-pyro | | | GGAGAGTTGTTGGGGAAG | | | | Pyrosequencing primer for PCR1 and PCR2 | |
|  | SH2D3C-PCR1 | | | CGAGTTTCGCGGGAGCGGGGCGGGGCGGGGTGGGGCGAGGCGAGGCGGAGTTGAGGGCGTGTCTAGGGGCGTGGCCTACCCCAGGCTGCGC | | | | PCR1 sequences to be analyzed before bisulfite treatment | |
|  | SH2D3C-PCR2 | | | CGAGCGTTGGAGCCGGGCGGGCGAGGCGGAGGGGTGTGCTAGGGGGCGAGGCCTTCCCGACACTGACTGATGGGGAGGGGGCGGAGCGAGACCGCAGGCGGAG | | | | PCR2 sequences to be analyzed before bisulfite treatment | |
| FAM221A | FAM221A-MR-F | | | GGTAAATTGTATTTTATGTGG | | | | bisulfite PCR primers | |
|  | FAM221A-MR-R | | | Bio-ATAAAAAAACCAAAAACTTCCCTAC | | | |  |  |
|  | FAM221A-MR-pyro-seq | | | GGTTTTGGTGTTTGG | | | | Pyrosequencing primer | |
|  | FAM221A-MR | | | CGCCGGCAGCCAGTAGGGGCGCGCGTCCTGCGCAGTTGCCGCGCGCGGCCTCCTCGGGCTTCAGGCCGGGCCAATCAGGAGGCGGGCGG | | | | sequences to be analyzed before bisulfite treatment | |
| GAPDH | GAPDH-PCR1-F | | | TTTTTAAAGTTTTTTTGTTTTATTTAAG | | | | bisulfite PCR1 primers | |
|  | GAPDH-PCR1-R | | | Bio-CCCAAAATCTTAAAACCTAAACTAC | | | |  |  |
|  | GAPDH-PCR2-F | | | AAGAAAGGGGAGGGGGTAGGT | | | | bisulfite PCR2 primers | |
|  | GAPDH-PCR2-R | | | Bio-CAACAAAACACTAAAAAATCAAAAAC | | | |  |  |
|  | GAPDH-PCR1-pyro | | | TTTTTAAAGTTTTTTTGTTTTATTTAAG | | | | Pyrosequencing primer for PCR1 | |
|  | GAPDH-PCR2-pyro | | | AAGAAAGGGGAGGGGGTAGGT | | | | Pyrosequencing primer for PCR2 | |
|  | GAPDH-PCR1 | | | CGTGTAAGGGTCCCCGTCCTTGACTCCCTAGTGTCCTGCTGCCCACAGTCCAGTCCTGGGAACCAGCACCGATCACCTCCCATCGGGCCAATCTCAGTCCCTTCCCCCCTACGTCGGG | | | | sequences to be analyzed before bisulfite treatment | |
|  | GAPDH-PCR2 | | | CGCGTGCAGCCGCGAGCGGTGCTGGGCTCCGGCTCCAATTCCCCATCTCAGTCGTTCCCAAAGTCCTCCTGTTTCATCCAAGCGTGTAAGGGTCCCC | | | | sequences to be analyzed before bisulfite treatment | |
| LINE1  AS | LINE1-MR-F | | | TTTAGATTGTTGTGTTAGTAATTAG | | | | bisulfite PCR primers | |
|  | LINE1-MR-R | | | Bio-TTTCTACATTTCCATCTAAAATACC | | | |  |  |
|  | LINE1-MR-pyro-seq | | | GAGTTAGGTGTGGGATATAG | | | | LINE1 pyrosequencing primer | |
|  | LINE1-MR | | | TCTCGTGGTGCGCCGTTTCTTAAGCCGGTCTGAAAAGCGCAATATTCGGGTGGGAGTGAC | | | | sequences to be analyzed before bisulfite treatment | |
