# Supplementary material for: Genome-wide determination of on-target and off-target characteristics for RNA-guided DNA methylation by dCas9 methyltransferases
Source: Gigascience. 2018 Feb 19;7(3):giy011. doi: 10.1093/gigascience/giy011 (PMC5888497; doi:10.1093/gigascience/giy011)
Supplement: Response_to_Reviewer_Comments_Original_Submission.pdf [file giy011_response_to_reviewer_comments_original_submission.pdf]

Author's Response To Reviewer Comments

Responses to Reviewers:

Reviewer one:
Major comments
1. There seems to be a lack of integration for the analyses of the three genome-wide datasets, i.e. RNA-seq, bisulfite sequencing, and ChIP. An integrated analysis would potentially uncover the molecular mechanisms for off-target gene expression changes. The authors commented on the lack of strong correlation between ChIP signal and methylation changes, but it equally important to know whether gene expression changes can be explained by dCas9 binding and / or methylation changes at the promoter/enhancer.
Re: In the revision, we have conducted integrative analysis between gene expression and methylation changes in promoter, between gene expression and dCas9 binding. In general, the correlation between changes in gene expression and either promoter methylation or binding is very weak (Revised Fig. 8g).

2. It's been known ChIP signal can be highly biased towards open chromatins in a non-specific manner, and thus it is crucial to call peaks with a control IP sample. It is unclear whether this is what the authors have done. My previous experience with dCas9 ChIP is that a pairwise peak calling strategy helps remove the majority of non-specific peaks (Wu et al 2014 Nat Biotech). A cleaner set of peaks may reveal much stronger correlation between binding and methylation changes, and / or gene expression changes. Similar strategy may be used for calling differentially methylated regions.
Re: In the revision, we have conducted two more ChIP repeats. The peak call was conducted pairwise to the control input sample, which is now described in the revised manuscript. Only peaks that were found in the three independent ChIP repeats were considered as off-target binding peaks. Peaks in repeated sequences and rDNA regions were removed similar to Wu et al. For finding the differentially methylation regions, we in the revision used a more stringent parameter for filtering. First pairwise comparison was firstly conducted between control transfection and cells transfecting with higher amount of dCas9 fusion and gRNAs. These DMRs was then subjected to a dose- and gRNA-dependent methylation/demethylation filtering.

3. As the authors suggested, a single guide RNA, uPA T2, that is highly G-rich, or AG-rich in the seed region, can potentially be the cause of most off-target activities. Once the authors cleaned up the ChIP peaks using strategies recommend above, they can check the seed matches and see if peaks are dominated by this gRNA. If there is a strong correlation between binding and gene expression change, one can then also see if off-target binding of a particular gRNA is causing more gene expression changes. We previously showed the choice of gRNAs have huge effect on ChIP binding, it would be great to know whether similar design can help reduce off-targets in methylation and gene expression change.
Re: Thanks for the suggestions. We have included the discussion on why uPA T2 gRNA causes most off-target activity in the revision (Figure 8 and page 14-15).

4. The authors showed in Fig. 4 that thousands of genes changed expression upon transfecting uPA gRNAs and four fusion proteins (DNMT1, DNMT3A, DNMT3B, EGFP). However, it is

unclear if the same set of genes changed in the same direction in all four cases. If this is the case, it would be more direct support for the model that the changes are caused by CRISPRi-type of effect, as proposed by the authors (line 460-462).
Re: Crossed-comparison was performed for the four groups of DEGs (Figure 8f). Only 18-32% of the genes were commonly found, indicating the existence other factors on gene expression changes. We have revised our previous conclusion of the findings.

Minor comments
1. Line 109: correct citations
2. Cite and comment on a previous work (PMID: 27662091) that studied the same question using ChIP-seq
3. The off-target activity at the GAPDH locus shown in supplementary fig 3 and supplementary figure 5 is very interesting. The pattern looks almost identical between fig s3b and fig s5b. It suggests that the off-target activity depends on dCas9 to be loaded with some guide RNA but doesn't matter what guide RNA is loaded. This seems to be consistent with the idea that loading of a guide RNA stabilizes the dCas9 protein, and higher abundance of the dCas9 protein leads to off-target activity at the GAPDH locus. The presence of off-target activity at the GAPDH locus but not SH2D3C/FAM221A loci despite the other way predicted by gRNA mismatches, suggest the GAPDH loci may be highly accessible and facilitates dCas9 binding. Is this supported by DHS and ChIP data?
4. Fig. 4: define FC. P values should be 1e-11 not 10e10
Re: All above comments have been addressed in the revision.

Reviewer #2:
1) In general, this study should be presented in a shorter format. The authors have undoubtedly created a lot of data and invested time and money in the study, however, the manuscript is difficult to read and is not very cohesive. For example, the CRISPRme1 and CRISPRme2 should probably be compared side by side.
RE: We have significantly streamlined the revised manuscript. Instead of presenting CRISPRme1 and CRISPRme 2 side by side. In the revised manuscript, we have supplemented the CRISPRme2 study as extended discussion in the supplementary file and cite this in the discussion. The CRISPRme2 system mainly address the question of gRNA-independent off-target effects.

2) I don't understand the argument that hypomethylated regions are "likely stochastic DMRs resulted from in vitro cell cultivation and manipulations", while hypermethylated regions have to be the consequence of CRISPRme off-targeting. Hypomethylated regions should be used as a metric for noise in the experiments and to access false positive rates. I am very worried that the number of hypomethylated DMRs is in the same range as the number of hypermethylated DNA (group 1: hypermethylated DMR (hyper-DMR) = 16169, hypomethylated DMR (hypo-DMR) = 11172; group 3: hyper-DMR = 12500, hypo-DMR = 11996). To me this suggests that the off target effects the authors see are merely an expression of noise in the system. Unless the authors can rectify this relation, I am afraid their study remains inconclusive or underpowered for a majority of the claims.
RE: To ensure the DMRs are resulted from expressing dCas9 methyltransferases and gRNAs, we

have included a more stringent filtering steps for the identification of both hypermethylated and hypomethylated DMRs. There are clearly more hyper-methylated DMRs than hypomethylated ones. Furthermore, the genomic distributions of hyper-methylated DMRs and hypomethylated DMRs are different (Revised Fig. 6 and Supplementary Fig. S9). In the revised manuscript, we have included both types of DMRs into analysis.

3) The authors find significant de novo methylation in of the uPA promoter with scrambled gRNAs, although to a slightly lower extent than the uPA targeting gRNA. I am surprised that these off target effects the authors describe (there was another one on GAPDH, I think) sampling so few loci do not translate into genome-wide elevations of methylation levels.
Re: Both uPA and GAPDH promoter are hypomethylated in HEK293T cells and located in open chromatin regions. Our study discovers that these off target hypermethylated DMRs are highly enriched in promoters, 5'UTR and CpG islands. Furthermore, even in the same promoter, e.g. GAPDH promoter, not all CpG sites are equally un-specifically methylated. This also explain why previous published dCas9 methyltransferase studies could find identify this off-target effect, as they only study a few selected CpGs. Regarding the genome-wide elevations of methylation, since there are also sites which are demethylated due to expression of dCas9 and gRNAs, this will neutralize the general methylation level in cells.

In conclusion, I think the authors have done a significant amount of work, but I am wondering whether they are presenting the data in the best way possible and whether they are drawing the right conclusions. Maybe it would be best to concentrate on some core messages (inhibition not being methylation dependent, for example). I am especially worried about the off target effect conclusions, which in my opinion are not supported by the data.
Re: We have in the revision carefully re-analyzed our WGBS data, by including a more stringent filtering criteria, to ensure that the hypermethylated DMRs are off-target effects resulted from expressing dCas9 methyltransferase and gRNAs. Our data and results both WGBS and bisulfite pyrosequencing consistently support the finding that dCas9 methyltransferase can cause quite a number > 1,000 off-target DMRs, which is predominantly guided gRNAs. The off-target methylation effect is that robust as we had presented in the previous version. But the existence should definitely be aware, especially for those located in open chromatin regions.

Reviewer #3:
1) The methods part is describing in enough detail the experimental procedure, except for the WGBS sequencing section, where no protocol for library generation was provided. The authors should specify how the sequencing samples were prepared (library prep, which protocol PBAT or tagmentation or any other, number of amplification rounds that were used).
Re: Detail description of the WGBS protocol is now provided in the revision.

2) I have very serious doubts regarding the specificity analysis with WGBS. First, the authors performed only one biological repeat for WGBS per experimental sample, which does not allow to assess the natural variability (what they call as stochastic DMRs) in methylation state in the samples. Second, what, I consider even more troublesome a biased selection of the "valid" results is performed. As they write on pp.10 lines 275-277, the hypomethylated regions were omitted in the analysis and they argue that these are most likely caused by stochastic methylation changes.

Of course methylation changes are observed in a cell population as reported in many published reports, however these can be both gain and loss of DNA methylation at various loci, otherwise in a longer run the global DNA methylation levels would become higher if only gain is observed. Therefore, I find it unjustified to select only DMRs that gain methylation and conclude that methylation gain is observed. The
authors should show all the DMRs that are observed (with gain and loss of DNA). Because the second biological repeat for WGBS is missing, no conclusions about the stochasticity for the DMR can be made (even when using sophisticated statistical tests on a weak data basis).
Re: We really appreciate the comments and suggestions to the WGBS DMR analysis. In the revision, we have included both hypermethylated and hypomethylated DMRs into our results and discussion. Furthermore, since we have observed there is a dose-dependent and gRNA dependent methylation efficacy of the dCas9 methyltransferase, in addition to using the statistical test, we have applied a more stringent filtering step to identification of real DMRs.

3) Pp. 12, line 344: Regarding the repression results of the targeted genes. The authors observe a similar repression of the genes when targeted with wt or catalytically inactive MTase variants and conclude that the deposited DNA methylation does not repress transcription, but rather the sole dCas9-binding is responsible for this effect. However, the for both dCas9 binding and the location of DNA methylation regarding genes regulatory elements is critical, therefore these experiments by themselves do not allow to draw this conclusion.
Re: The previous conclusion has revised (page 15, last paragraph).

4) pp. 14, line 423-429: The authors note that reduced expression "does not appear to be due to de novo methylation" as similar repression effects are observed with dCas9 control fusions. However, it is known that dCas9 can interfere with binding RNA polymerase and/or other components in the TSS/gene body, yet the exact position of gRNA/dCas9 binding is important for blockage and in this case can have a major effect, nevertheless the effect of DNA methylation cannot be excluded. Especially, since targeted DNA methylation is not stable and gradually disappears with cell division, similarly as the expression of the targeting constructs.
Re: Our conclusion for methylation and expression changes have been carefully revised in the revision (section 4.7 and discussion).

5) Page 18, lines 575-578: I don't understand how hypomethylated DMRs are due to stochastic methylation, yet the hypermethylated DMRs are due to off-target methylation. This assumption made by the authors introduces a very strong bias in the data analysis and leads to wrong conclusions! Moreover, in the absence of a second biological replicate, it is impossible to discern which DMRs are due to stochastic or targeted methylation. Overall, for me, no valid conclusions can be drawn from this analysis in the current form.
Re: In the revision, we have included both hyper and hypo DMRs in the analysis. Furthermore, more carefully and detailed analyses of these DMRs were conducted.

Minor points:
1) pp. 12, line 376: Previous work of others, where Dnmt3a or Dnmt3a3L catalytic mutants were targeted using dCas9, showed that the cellular DNA methylation machinery is not recruited as there was no targeted methylation observed when targeting the mutants.
2) Page 16, line 495: It seems to me that the CRISPRme2.0 might suffer from protein folding

issues possibly due to shorter linker between the dCas9 and the MTase
Re: Above points have been addressed in the revision. For the CRISPRme2.0, we have seen that expressing the DNMT3A only cause dramatic off-target methylation of the GAPHD promoter. The shorter linker makes dCas9-DNMT3A more easily entering the nucleus compared to dCas9-BFP-DNMT3A. In addition, to streamline the manuscript as suggested by the 2nd reviewer. We have separated the CRISPRme2.0 system as one additional supplementary file.

Overall, I recognize that the authors performed lots of high-end experiments that attempt to investigate the specificity of targeted DNA methylation in the cells. However, in my opinion the work partially suffers from lack of novelty (for the dCas9 targeting constructs - despite having Dnmt3b), the WGBS data analysis and interpretation suffers from the lack of second repeat and strong bias in DMR analysis and in my opinion the conclusion that DNA methylation does not contribute to gene repression is not enough supported by experimental results presented and alternative explanations are possible.
Re: In the revision, we have carefully analyzed our WGBS data, repeated the ChIP experiments, and revised our conclusions on gene expression and DNA methylation.
