# Supplementary material for: Genome-wide determination of on-target and off-target characteristics for RNA-guided DNA methylation by dCas9 methyltransferases
Source: Gigascience. 2018 Feb 19;7(3):giy011. doi: 10.1093/gigascience/giy011 (PMC5888497; doi:10.1093/gigascience/giy011)
Supplement: Response_to_Reviewer_Comments_Revision_1.pdf [file giy011_response_to_reviewer_comments_revision_1.pdf]

Author's Response To Reviewer Comments

Dear Editor and reviewers,

We really appreciate the reviewers' comments and concerns of the off-target effects we have discovered for dCas9 methyltransferases.

Of course, we fully aware of and understand that any finding of negative effects of the CRISPR technology should be carefully and thoroughly addressed before announcing it to the whole scientific and CRISPR community.

Unlike the original CRISPR/Cas9 technology, of which the endonuclease activity of Cas9 depends heavily on the base-pairing between the guide sequences and the target site (proto-spacer), the dead Cas9 (dCas9) derived CRISPR technology and applications are more depending on the physical interaction between dCas9/gRNA complex and the DNA loci, and more tolerance to mismatches. As already demonstrated in figure 2 and Supplementary Figure 4 of this study and several previous investigations by ChIP-seq, the criteria of defining off-target sites (based on mismatches) from wild type Cas9 is not suitable for the dCas9 methyltransferases. Although this study only evaluate the dCas9 methyltransferases, we speculate that this off-target effects are most likely to be the same for other kind of dCas9 based effectors.

We have conducted more WGBS validation experiment in the revision (Supplementary Figure 14). WGBS analyses are now conducted in HEK293T cells transfected with dCas9-BGP-DNMT3A and uPA gRNA (n = 3), and compared to transfection control (pUC19, n = 3). We validate that the hypermethylated DMRs found in our first WGBS experiments are significantly increased in cells expressing dCas9-BGP-DNMT3A and uPA gRNAs. Furthermore, we also validated DHS sites are prone to unspecific methylation. These results collectedly and consistently validate our finding that there is a certain degree of unspecific methylation causing by dCas9 methyltransferase and CRISPR gRNAs, and promoter region and open chromatin regions are prone to unspecific methylation.

Kr,

Yonglun Luo
Associate Professor, Department of Biomedicine, Aarhus University
Executive Director, Lars Bolund Institute of Regenerative Medicine, BGI-Qingdao, China
