# Supplementary material for: Genome-wide determination of on-target and off-target characteristics for RNA-guided DNA methylation by dCas9 methyltransferases
Source: Gigascience. 2018 Feb 19;7(3):giy011. doi: 10.1093/gigascience/giy011 (PMC5888497; doi:10.1093/gigascience/giy011)
Supplement: Response_to_Reviewer_Comments_Revision_2.pdf [file giy011_response_to_reviewer_comments_revision_2.pdf]

Author's Response To Reviewer Comments

Dear Editor and Reviewers,

Thanks again for the constructive comments and the approval of our study. We have now include a paragraph in the discussion section describing the difference in off-target effects between Cas9 and dCas9 methyltransferases and effector proteins.

Kr
Alun
