# Supplementary material for: Genome-wide determination of on-target and off-target characteristics for RNA-guided DNA methylation by dCas9 methyltransferases
Source: Gigascience. 2018 Feb 19;7(3):giy011. doi: 10.1093/gigascience/giy011 (PMC5888497; doi:10.1093/gigascience/giy011)
Supplement: Response_to_Reviewer_Comments_Revision_3.pdf [file giy011_response_to_reviewer_comments_revision_3.pdf]

Author's Response To Reviewer Comments

Dear GigaScience Editorial team and reviewers,

Great thanks for all the constructive comments to our manuscript. We have included the addition discussion on the off-target effects of dCas9-based methyltransferases and other effector proteins. In addition, we have cited the GigaDB file of our study in the revised MS (highlighted in blue).

All supplementary files are now converted to the correct format.

Kr,
Alun
