# Supplementary material for: Genome-wide determination of on-target and off-target characteristics for RNA-guided DNA methylation by dCas9 methyltransferases
Source: Gigascience. 2018 Feb 19;7(3):giy011. doi: 10.1093/gigascience/giy011 (PMC5888497; doi:10.1093/gigascience/giy011)
Supplement: Reviewer_1_Report_(Original_Submission) -- Xuebing Wu — 28 Feb 2017 Reviewed [file giy011_reviewer_1_report_(original_submission).pdf]

Reviewer Report

Title: Genome-wide determination of on-target and off-target characteristics for RNA-guided DNA Methylation by dCas9 methyltransferases

Version: Original Submission **Date:** 28 Feb 2017

Reviewer name: Xuebing Wu

Reviewer Comments to Author:

Lin et al have generated an impressive amount of data to address the off-target effect of dCas9-based DNA methylation editors. An unbiased characterization of the off-target activities for epigenome editors is essential for understanding the limitation of such potentially powerful tools. The authors have combined detailed analyses of individual loci with several genome-wide assays to look at off-target binding, methylation, and gene expression changes. They first showed that dCas9-BFP-DNMT3A fusion was able to methylate two target loci directed by gRNAs, but also have strong off-target activity at the GAPDH locus, in a guide RNA dependent but sequence independent manner. Those off-target activity cannot be reduced without significantly affecting on-target activity. Expressing those fusion proteins changed the expression of thousands of genes, even when dCas9 is fused to GFP, suggesting off-targets were caused by transcription interference by dCas9 binding. Whole genome bisulfite sequencing identified thousands of differentially methylated regions that enrich for short seed match to the guide RNA. ChIP-seq also identified thousands of binding sites in the genome but with very weak overlap with hypermetylated regions.

The data together suggested that dCas9-based tools, including epigenome editor and CRISPRi/a, can have huge number of off-targets in the genome. Although this is consistent with previous Cas9 ChIP studies, such a result seems to contradict previous RNA-seq studies of dCas9 specificities that minimum off-target gene expression changes were observed (e.g. Gilbert et al 2013 Cell, Thakore et al 2015 Nature Methods). It is important to understand the difference between this study and previous works. For example, is it because the use of a guide RNA that has lots of partial matches in the genome? Things like this are important to know for improving the specificity of those dCas9 fusion proteins.

Major comments

1. There seems to be a lack of integration for the analyses of the three genome-wide datasets, i.e. RNA-seq, bisulfite sequencing, and ChIP. An integrated analysis would potentially uncover the molecular mechanisms for off-target gene expression changes. The authors commented on the lack of strong correlation between ChIP signal and methylation changes, but it equally important to know whether gene expression changes can be explained by dCas9 binding and / or methylation changes at the promoter/enhancer.

2. It's been known ChIP signal can be highly biased towards open chromatins in a non-specific manner, and thus it is crucial to call peaks with a control IP sample. It is unclear whether this is what the authors

have done. My previous experience with dCas9 ChIP is that a pairwise peak calling strategy helps remove the majority of non-specific peaks (Wu et al 2014 Nat Biotech). A cleaner set of peaks may reveal much stronger correlation between binding and methylation changes, and / or gene expression changes. Similar strategy may be used for calling differentially methylated regions.

3. As the authors suggested, a single guide RNA, uPA T2, that is highly G-rich, or AG-rich in the seed region, can potentially be the cause of most off-target activities. Once the authors cleaned up the ChIP peaks using strategies recommend above, they can check the seed matches and see if peaks are dominated by this gRNA. If there is a strong correlation between binding and gene expression change, one can then also see if off-target binding of a particular gRNA is causing more gene expression changes. We previously showed the choice of gRNAs have huge effect on ChIP binding, it would be great to know whether similar design can help reduce off-targets in methylation and gene expression change.

4. The authors showed in Fig. 4 that thousands of genes changed expression upon transfecting uPA gRNAs and four fusion proteins (DNMT1, DNMT3A, DNMT3B, EGFP). However, it is unclear if the same set of genes changed in the same direction in all four cases. If this is the case, it would be more direct support for the model that the changes are caused by CRISPRi-type of effect, as proposed by the authors (line 460-462).

Minor comments
1. Line 109: correct citations
2. Cite and comment on a previous work (PMID: 27662091) that studied the same question using ChIP-seq
3. The off-target activity at the GAPDH locus shown in supplementary fig 3 and supplementary figure 5 is very interesting. The pattern looks almost identical between fig s3b and fig s5b. It suggests that the off-target activity depends on dCas9 to be loaded with some guide RNA but doesn't matter what guide RNA is loaded. This seems to be consistent with the idea that loading of a guide RNA stabilizes the dCas9 protein, and higher abundance of the dCas9 protein leads to off-target activity at the GAPDH locus. The presence of off-target activity at the GAPDH locus but not SH2D3C/FAM221A loci despite the other way predicted by gRNA mismatches, suggest the GAPDH loci may be highly accessible and facilitates dCas9 binding. Is this supported by DHS and ChIP data?
4. Fig. 4: define FC. P values should be 1e-11 not 10e10

Methods

Are the methods appropriate to the aims of the study, are they well described, and are necessary controls included? Yes

Conclusions

Are the conclusions adequately supported by the data shown? Yes

Reporting Standards

Does the manuscript adhere to the journal's guidelines on minimum standards of reporting? Yes

Statistics

Are you able to assess all statistics in the manuscript, including the appropriateness of statistical tests used? Yes, and I have assessed the statistics in my report.

Quality of Written English

Please indicate the quality of language in the manuscript: Acceptable

Declaration of Competing Interests

Please complete a declaration of competing interests, considering the following questions:

- Have you in the past five years received reimbursements, fees, funding, or salary from an organisation that may in any way gain or lose financially from the publication of this manuscript, either now or in the future?
- Do you hold any stocks or shares in an organisation that may in any way gain or lose financially from the publication of this manuscript, either now or in the future?
- Do you hold or are you currently applying for any patents relating to the content of the manuscript?
- Have you received reimbursements, fees, funding, or salary from an organization that holds or has applied for patents relating to the content of the manuscript?
- Do you have any other financial competing interests?
- Do you have any non-financial competing interests in relation to this paper?

If you can answer no to all of the above, write 'I declare that I have no competing interests' below. If your reply is yes to any, please give details below.

I declare that I have no competing interests

I agree to the open peer review policy of the journal. I understand that my name will be included on my report to the authors and, if the manuscript is accepted for publication, my named report including any attachments I upload will be posted on the website along with the authors' responses. I agree for my report to be made available under an Open Access Creative Commons CC-BY license (http://creativecommons.org/licenses/by/4.0/). I understand that any comments which I do not wish to

be included in my named report can be included as confidential comments to the editors, which will not be published.

I agree to the open peer review policy of the journal

To further support our reviewers, we have joined with Publons, where you can gain additional credit to further highlight your hard work (see: https://publons.com/journal/530/gigascience). On publication of this paper, your review will be automatically added to Publons, you can then choose whether or not to claim your Publons credit. I understand this statement.

Yes
