# Supplementary material for: Genome-wide determination of on-target and off-target characteristics for RNA-guided DNA methylation by dCas9 methyltransferases
Source: Gigascience. 2018 Feb 19;7(3):giy011. doi: 10.1093/gigascience/giy011 (PMC5888497; doi:10.1093/gigascience/giy011)
Supplement: Reviewer_2_Report_(Original_Submission) -- Tamir Chandra — 14 Mar 2017 Reviewed [file giy011_reviewer_2_report_(original_submission).pdf]

Reviewer Report

Title: Genome-wide determination of on-target and off-target characteristics for RNA-guided DNA Methylation by dCas9 methyltransferases

Version: Original Submission **Date:** 14 Mar 2017

Reviewer name: Tamir Chandra

Reviewer Comments to Author:

In this study "Genome wide determination of on-target and off-target characteristics for RNA-guided DNA Methylation by dCas9 methyltransferases (CRISPRme)" Lin et al. generated CRISPR fusion proteins with DNMT3A/B proteins and tested them for their on/off target effects. As applications for CRISPR proteins are being presented on a weekly basis, also with potential clinical use, it is important to assess the specificity of these approaches, even if the results might be to a certain extent redundant and replicated in other studies.

While there is potentially interesting data presented by the authors, I found several aspects of the study inconclusive. My general and specific concerns are as follows:

1) In general, this study should be presented in a shorter format. The authors have undoubtedly created a lot of data and invested time and money in the study, however, the manuscript is difficult to read and is not very cohesive. For example, the CRISPRme1 and CRISPRme2 should probably be compared side by side.

2) I don't understand the argument that hypomethylated regions are "likely stochastic DMRs resulted from in vitro cell cultivation and manipulations", while hypermethylated regions have to be the consequence of CRISPRme off-targeting. Hypomethylated regions should be used as a metric for noise in the experiments and to access false positive rates. I am very worried that the number of hypomethylated DMRs is in the same range as the number of hypermethylated DNA (group 1: hypermethylated DMR (hyper-DMR) = 16169, hypomethylated DMR (hypo-DMR) = 11172; group 3: hyper-DMR = 12500, hypo-DMR = 11996). To me this suggests that the off target effects the authors see are merely an expression of noise in the system. Unless the authors can rectify this relation, I am afraid their study remains inconclusive or underpowered for a majority of the claims.

3) The authors find significant de novo methylation in of the uPA promoter with scrambled gRNAs, although to a slightly lower extent than the uPA targeting gRNA. I am surprised that these off target effects the authors describe (there was another one on GAPDH, I think) sampling so few loci do not translate into genome-wide elevations of methylation levels.

In conclusion, I think the authors have done a significant amount of work, but I am wondering whether they are presenting the data in the best way possible and whether they are drawing the right conclusions. Maybe it would be best to concentrate on some core messages (inhibition not being methylation dependent, for example). I am especially worried about the off target effect conclusions, which in my opinion are not supported by the data.

Methods

Are the methods appropriate to the aims of the study, are they well described, and are necessary controls included? No

Conclusions

Are the conclusions adequately supported by the data shown? No

Reporting Standards

Does the manuscript adhere to the journal's guidelines on minimum standards of reporting? Yes

Statistics

Are you able to assess all statistics in the manuscript, including the appropriateness of statistical tests used? Yes, and I have assessed the statistics in my report.

Quality of Written English

Please indicate the quality of language in the manuscript: Acceptable

Declaration of Competing Interests

Please complete a declaration of competing interests, considering the following questions:

- Have you in the past five years received reimbursements, fees, funding, or salary from an organisation that may in any way gain or lose financially from the publication of this manuscript, either now or in the future?
- Do you hold any stocks or shares in an organisation that may in any way gain or lose financially from the publication of this manuscript, either now or in the future?
- Do you hold or are you currently applying for any patents relating to the content of the manuscript?
- Have you received reimbursements, fees, funding, or salary from an organization that holds or has applied for patents relating to the content of the manuscript?
- Do you have any other financial competing interests?
- Do you have any non-financial competing interests in relation to this paper?

If you can answer no to all of the above, write 'I declare that I have no competing interests' below. If your reply is yes to any, please give details below.

I declare that I have no competing interests

I agree to the open peer review policy of the journal. I understand that my name will be included on my report to the authors and, if the manuscript is accepted for publication, my named report including any attachments I upload will be posted on the website along with the authors' responses. I agree for my report to be made available under an Open Access Creative Commons CC-BY license (http://creativecommons.org/licenses/by/4.0/). I understand that any comments which I do not wish to be included in my named report can be included as confidential comments to the editors, which will not be published.

I agree to the open peer review policy of the journal

To further support our reviewers, we have joined with Publons, where you can gain additional credit to further highlight your hard work (see: https://publons.com/journal/530/gigascience). On publication of this paper, your review will be automatically added to Publons, you can then choose whether or not to claim your Publons credit. I understand this statement.

Yes
