# Supplementary material for: Genome-wide determination of on-target and off-target characteristics for RNA-guided DNA methylation by dCas9 methyltransferases
Source: Gigascience. 2018 Feb 19;7(3):giy011. doi: 10.1093/gigascience/giy011 (PMC5888497; doi:10.1093/gigascience/giy011)
Supplement: Reviewer_2_Report_(Revision_1) -- Tamir Chandra — 07 Aug 2017 Reviewed [file giy011_reviewer_2_report_(revision_1).pdf]

Reviewer Report

Title: Genome-wide determination of on-target and off-target characteristics for RNA-guided DNA Methylation by dCas9 methyltransferases

Version: Revision 1 **Date:** 07 Aug 2017

Reviewer name: Tamir Chandra

Reviewer Comments to Author:

Ln 400:
I am still unsure as how representative selected loci to study off-target effects are. The authors could maybe calculate the number of loci in the genome that have similar number of mismatches as the selected loci and compare them in their genome-wide data.
Ln 469:
The authors mention fluctuations of methylation states in cancer cells. The study they are citing follows cells over many generations (up to 300) and is therefore not a suitable comparison. I am not clear how stochastic changes of methylation, as the authors claim, would be seen in a population of cells within the time scales the experiments were conducted. I still think we cannot exclude that the potential off-target effects are an expression of noise between experiments and with only one replicate, there is no way that the authors can back up their claims. Although the number of hypomethylated regions has gone down, it is still significant, at a similar level to the hypermethylated regions, weakening the argument that the hypermethylated regions observed are results of CRISPR targeting. I believe that the authors are looking at noise/technical variation between two data-sets.
495 onwards
Correlating gain of methylation to open chromatin regions is very descriptive and often subject to cofounding factors.

I believe that the study does not provide enough evidence nor is it conducted with the required statistical power to quantify the extent of off-target effects of CRISPR targeting.

Methods

Are the methods appropriate to the aims of the study, are they well described, and are necessary controls included? No

Conclusions

Are the conclusions adequately supported by the data shown? No

Reporting Standards

Does the manuscript adhere to the journal's guidelines on [minimum standards of reporting?]() Yes

Statistics

Are you able to assess all statistics in the manuscript, including the appropriateness of statistical tests used? No, I do not feel adequately qualified to assess the statistics.

Quality of Written English

Please indicate the quality of language in the manuscript: Acceptable

Declaration of Competing Interests

Please complete a declaration of competing interests, considering the following questions:

- Have you in the past five years received reimbursements, fees, funding, or salary from an organisation that may in any way gain or lose financially from the publication of this manuscript, either now or in the future?
- Do you hold any stocks or shares in an organisation that may in any way gain or lose financially from the publication of this manuscript, either now or in the future?
- Do you hold or are you currently applying for any patents relating to the content of the manuscript?
- Have you received reimbursements, fees, funding, or salary from an organization that holds or has applied for patents relating to the content of the manuscript?
- Do you have any other financial competing interests?
- Do you have any non-financial competing interests in relation to this paper?

If you can answer no to all of the above, write 'I declare that I have no competing interests' below. If your reply is yes to any, please give details below.

I declare that I have no competing interests'

I agree to the open peer review policy of the journal. I understand that my name will be included on my report to the authors and, if the manuscript is accepted for publication, my named report including any attachments I upload will be posted on the website along with the authors' responses. I agree for my report to be made available under an Open Access Creative Commons CC-BY license (http://creativecommons.org/licenses/by/4.0/). I understand that any comments which I do not wish to be included in my named report can be included as confidential comments to the editors, which will not be published.

I agree to the open peer review policy of the journal

To further support our reviewers, we have joined with Publons, where you can gain additional credit to further highlight your hard work (see: https://publons.com/journal/530/gigascience). On publication of

this paper, your review will be automatically added to Publons, you can then choose whether or not to claim your Publons credit. I understand this statement.

Yes
