# Supplementary material for: Genome-wide determination of on-target and off-target characteristics for RNA-guided DNA methylation by dCas9 methyltransferases
Source: Gigascience. 2018 Feb 19;7(3):giy011. doi: 10.1093/gigascience/giy011 (PMC5888497; doi:10.1093/gigascience/giy011)
Supplement: Reviewer_3_Report_(Original_Submission) -- Tomasz Jurkowski — 19 Mar 2017 Reviewed [file giy011_reviewer_3_report_(original_submission).pdf]

Reviewer Report

Title: Genome-wide determination of on-target and off-target characteristics for RNA-guided DNA Methylation by dCas9 methyltransferases

Version: Original Submission **Date:** 19 Mar 2017

Reviewer name: Tomasz Jurkowski

Reviewer Comments to Author:

The manuscript "Genome wide determination of on-target and off-target characteristics for RNA-guided DNA methylation by dCas9 methyltransferases (CRISPRme) by Lin and colleagues investigates the specificity and efficiency of targeted DNA methylation using WGBS method. The question of specificity of targeted DNA methylation deposition is of course of great importance for their future application. The manuscript is mostly well written and describes extensive number of experimental results which seem to be well performed.
However, there are numerous issues with the data interpretation and analysis that shed doubt on the conclusions emphasized in the manuscript. Regarding the novelty of the work, a number of the targeted DNA methylation tools were already described recently in literature (Vojta; McDonald; Stepper and others) which should be mentioned in the introduction. The dCas9-Dnmt3a and its mutants were already created and tested by others and the dCas9 fusion of Dnmt3b does not provide any advantage in terms of specificity or efficiency over the already published constructs.
In addition in the already published targeted DNA methylation paper at least basic specificity analysis was performed in each case, here in this work the analysis is further extended by using the genome-wide approach.
Major points:
1) The methods part is describing in enough detail the experimental procedure, except for the WGBS sequencing section, where no protocol for library generation was provided. The authors should specify how the sequencing samples were prepared (library prep, which protocol PBAT or tagmentation or any other, number of amplification rounds that were used).
2) I have very serious doubts regarding the specificity analysis with WGBS. First, the authors performed only one biological repeat for WGBS per experimental sample, which does not allow to assess the natural variability (what they call as stochastic DMRs) in methylation state in the samples. Second, what, I consider even more troublesome a biased selection of the "valid" results is performed. As they write on pp.10 lines 275-277, the hypomethylated regions were omitted in the analysis and they argue that these are most likely caused by stochastic methylation changes. Of course methylation changes are observed in a cell population as reported in many published reports, however these can be both gain and loss of DNA methylation at various loci, otherwise in a longer run the global DNA methylation levels would become higher if only gain is observed. Therefore, I find it unjustified to select only DMRs that gain methylation and conclude that methylation gain is observed. The authors should show all the DMRs that are observed (with gain and loss of DNA). Because the second biological repeat for WGBS is missing, no conclusions about the stochasticity for the DMR can be made (even when using sophisticated statistical

tests on a weak data basis).
3) Pp. 12, line 344: Regarding the repression results of the targeted genes. The authors observe a similar repression of the genes when targeted with wt or catalytically inactive MTase variants and conclude that the deposited DNA methylation does not repress transcription, but rather the sole dCas9-binding is responsible for this effect. However, the for both dCas9 binding and the location of DNA methylation regarding genes regulatory elements is critical, therefore these experiments by themselves do not allow to draw this conclusion.
4) pp. 14, line 423-429: The authors note that reduced expression "does not appear to be due to de novo methylation" as similar repression effects are observed with dCas9 control fusions. However, it is known that dCas9 can interfere with binding RNA polymerase and/or other components in the TSS/gene body, yet the exact position of gRNA/dCas9 binding is important for blockage and in this case can have a major effect, nevertheless the effect of DNA methylation cannot be excluded. Especially, since targeted DNA methylation is not stable and gradually disappears with cell division, similarly as the expression of the targeting constructs.
5) Page 18, lines 575-578: I don't understand how hypomethylated DMRs are due to stochastic methylation, yet the hypermethylated DMRs are due to off-target methylation. This assumption made by the authors introduces a very strong bias in the data analysis and leads to wrong conclusions! Moreover, in the absence of a second biological replicate, it is impossible to discern which DMRs are due to stochastic or targeted methylation. Overall, for me, no valid conclusions can be drawn from this analysis in the current form.

Minor points:
1) pp. 12, line 376: Previous work of others, where Dnmt3a or Dnmt3a3L catalytic mutants were targeted using dCas9, showed that the cellular DNA methylation machinery is not recruited as there was no targeted methylation observed when targeting the mutants.
2) Page 16, line 495: It seems to me that the CRISPRme2.0 might suffer from protein folding issues possibly due to shorter linker between the dCas9 and the MTase

Overall, I recognize that the authors performed lots of high-end experiments that attempt to investigate the specificity of targeted DNA methylation in the cells. However, in my opinion the work partially suffers from lack of novelty (for the dCas9 targeting constructs - despite having Dnmt3b), the WGBS data analysis and interpretation suffers from the lack of second repeat and strong bias in DMR analysis and in my opinion the conclusion that DNA methylation does not contribute to gene repression is not enough supported by experimental results presented and alternative explanations are possible.

Methods

Are the methods appropriate to the aims of the study, are they well described, and are necessary controls included? No

Conclusions

Are the conclusions adequately supported by the data shown? No

Reporting Standards

Does the manuscript adhere to the journal's guidelines on minimum standards of reporting? No

Statistics

Are you able to assess all statistics in the manuscript, including the appropriateness of statistical tests used? No, I do not feel adequately qualified to assess the statistics.

Quality of Written English

Please indicate the quality of language in the manuscript: Needs some language corrections before being published

Declaration of Competing Interests

Please complete a declaration of competing interests, considering the following questions:

- Have you in the past five years received reimbursements, fees, funding, or salary from an organisation that may in any way gain or lose financially from the publication of this manuscript, either now or in the future?
- Do you hold any stocks or shares in an organisation that may in any way gain or lose financially from the publication of this manuscript, either now or in the future?
- Do you hold or are you currently applying for any patents relating to the content of the manuscript?
- Have you received reimbursements, fees, funding, or salary from an organization that holds or has applied for patents relating to the content of the manuscript?
- Do you have any other financial competing interests?
- Do you have any non-financial competing interests in relation to this paper?

If you can answer no to all of the above, write 'I declare that I have no competing interests' below. If your reply is yes to any, please give details below.

I declare no competing interests

I agree to the open peer review policy of the journal. I understand that my name will be included on my report to the authors and, if the manuscript is accepted for publication, my named report including any attachments I upload will be posted on the website along with the authors' responses. I agree for my report to be made available under an Open Access Creative Commons CC-BY license (http://creativecommons.org/licenses/by/4.0/). I understand that any comments which I do not wish to

be included in my named report can be included as confidential comments to the editors, which will not be published.

I agree to the open peer review policy of the journal

To further support our reviewers, we have joined with Publons, where you can gain additional credit to further highlight your hard work (see: https://publons.com/journal/530/gigascience). On publication of this paper, your review will be automatically added to Publons, you can then choose whether or not to claim your Publons credit. I understand this statement.

Yes
